# Design, synthesis, and biological evaluation of novel pyrimidine–sulfonamide derivatives as multi-targeted COX-2/5-LOX/sEH inhibitors with potential antiproliferative properties

**DOI:** 10.1039/d6ra04113b

**Published:** 2026-08-27

**Authors:** Lamya H. Al-Wahaibi, Alshaimaa Abdelmoez, Stefan Bräse, Salah A. Abdel-Aziz, Bahaa G. M. Youssif

**Affiliations:** a Department of Chemistry, College of Sciences, Princess Nourah Bint Abdulrahman University Riyadh 11671 Saudi Arabia; b Department of Pharmaceutical Organic Chemistry, Faculty of Pharmacy, Assiut University Assiut 71526 Egypt bgyoussif2@gmail.com (002)-01098294419; c Institute of Biological and Chemical Systems, IBCS-FMS, Karlsruhe Institute of Technology 76131 Karlsruhe Germany braese@kit.edu; d Department of Pharmaceutical Medicinal Chemistry, Faculty of Pharmacy, Al-Azhar University Assiut 71524 Egypt

## Abstract

Cyclooxygenase-2 (COX-2) and lipoxygenases (LOXs), two enzymes involved in arachidonic acid metabolism, have been linked to various cancers. As a result, it was exciting to develop dual COX-2/5-LOX inhibitors for promoting the development of new treatments. This study examines the synthesis and *in vitro* pharmacological properties of pyrimidine–sulfonamide derivatives. Compounds 4c, 4g, 4j, 4m, and 4o were the most effective COX-2 inhibitors and showed substantial inhibitory activity against 5-LOX. Compound 4c demonstrated significant inhibition of COX-2 (IC_50_ = 0.11 ± 0.01 µM) and 5-LOX (IC_50_ = 0.46 ± 0.02 µM) with comparable efficacy to celecoxib (IC_50_ = 0.08 ± 0.0004 µM) and surpassing the standard Zileuton (IC_50_ = 0.69 ± 0.03 µM). Additionally, compound 4c had the highest potency as a soluble epoxide hydrolase (sEH) inhibitor, with an IC_50_ of 4.30 ± 0.19 µM, demonstrating 1.6-fold lower potency than the reference AUDA but 5-fold greater potency than celecoxib. Compounds 4c, 4g, and 4j showed promising antiproliferative activity, with compound 4c being the most potent against the pancreatic (Panc-1) cancer cell line, and compound 4j the most effective against the breast MCF-7 cancer cell line. Their efficacy against these two cell lines exceeded that of the reference compounds celecoxib, sorafenib, and doxorubicin. Docking analysis of compound 4c showed a good fit within the active sites of both COX-2 and human sEH.

## Abstract 2

Cyclooxygenase-2 (COX-2) and lipoxygenases (LOXs), two enzymes involved in arachidonic acid metabolism, have been linked to various cancers.

## Graphical abstract

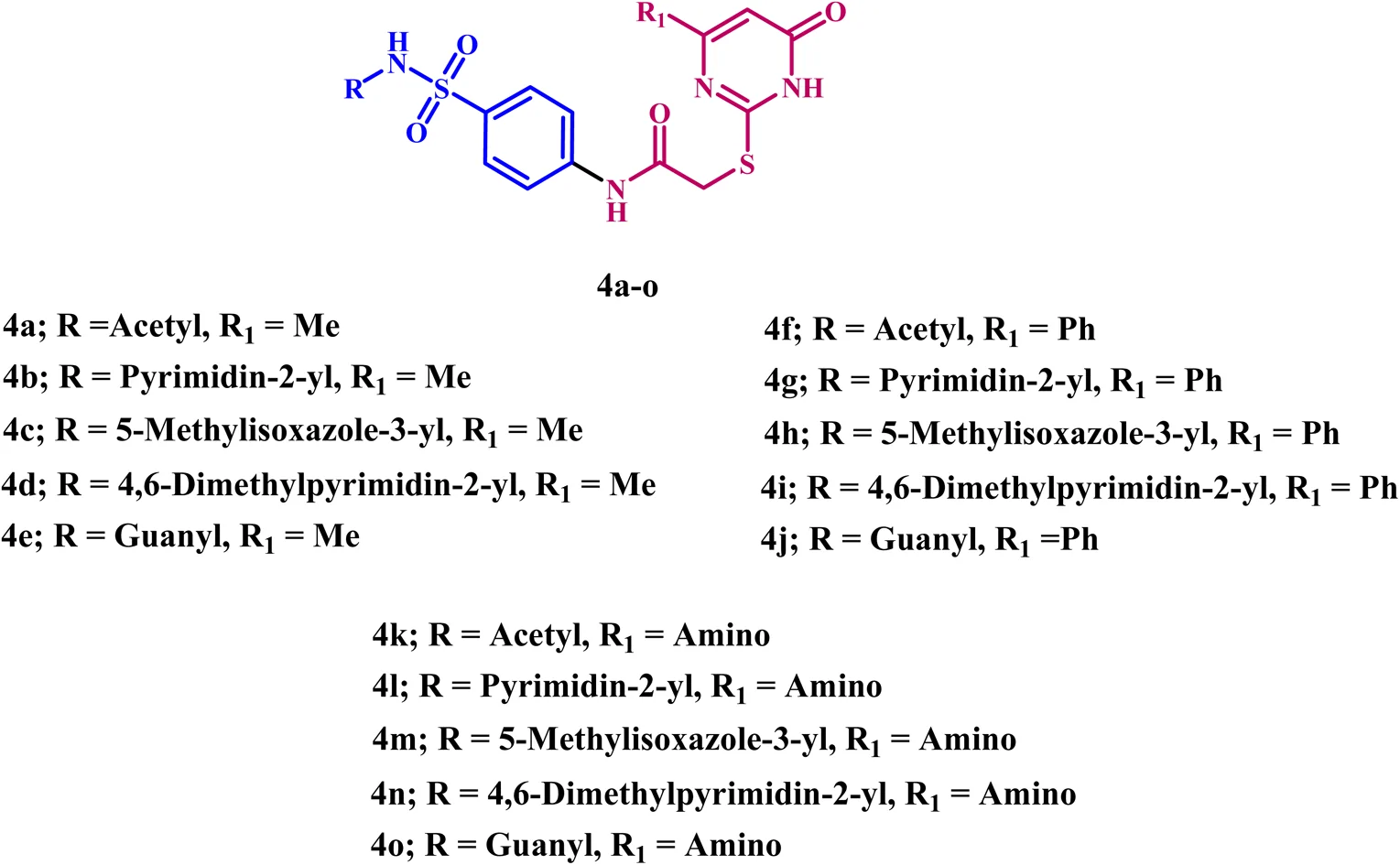

## 1. Introduction

Selective cyclooxygenase-2 (COX-2) inhibitors, also known as coxibs, are a class of nonsteroidal anti-inflammatory drugs (NSAIDs) that specifically block the COX-2 enzyme to alleviate pain and inflammation while attempting to minimize the gastrointestinal adverse effects associated with conventional NSAIDs.^1,2^ The analgesic and anti-inflammatory properties of NSAIDs arise from their inhibition of the synthesis of prostaglandins, which are hormone-like substances that facilitate pain and inflammation. There exist two major COX enzymes: COX-1 is a prevalent enzyme found in several tissues, responsible for synthesizing prostaglandins that safeguard the gastric mucosa and facilitate hemostasis. COX-2 is predominantly synthesised at locations of damage or inflammation.^3,4^ Selective COX-2 inhibitors predominantly inhibit the COX-2 enzyme, providing analgesic and anti-inflammatory benefits while minimizing interference with the protective role of COX-1 in the stomach, reducing the risk of gastrointestinal ulcers and hemorrhage.^5,6^

Currently, only one selective COX-2 inhibitor is available in the United States, while others are available in various countries or have been withdrawn from the market due to safety concerns.^7,8^ Celecoxib is the exclusive COX-2 inhibitor currently authorized and marketed in the United States. It is indicated for conditions such as osteoarthritis, rheumatoid arthritis, and acute pain. Rofecoxib was withdrawn from the market in 2004 due to an elevated risk of myocardial infarction and cerebrovascular accidents. Valdecoxib was retracted in 2005 owing to cardiovascular hazards and infrequent but severe dermatological reactions. Etoricoxib and Parecoxib are available in certain countries but are not approved in the United States.^9–11^

COX-2 produces prostacyclin (PGI2), an important vasodilator and inhibitor of platelet aggregation in the cardiovascular system. Selective COX-2 inhibitors (Coxibs) cause a pro-thrombotic condition, raise blood pressure, and increase the risk of myocardial infarctions and cerebrovascular accidents by selectively blocking PGI2 production *via* COX-2 while allowing thromboxane (TXA2) generation *via* COX-1.^12^

Soluble epoxide hydrolase (sEH) is essential for mitigating the cardiotoxic effects of Coxibs by preserving levels of essential cardioprotective lipid mediators (EETs) and re-establishing a healthy balance of factors that govern blood coagulation and vascular tone.^13,14^ Inhibition of soluble epoxide hydrolase mitigates these adverse cardiovascular effects through multiple pathways. sEH is the enzyme that catalyzes the conversion of profitable epoxyeicosatrienoic acids (EETs) into less active dihydroxyeicosatrienoic acids (DHETs).^15^ Inhibiting sEH increases the bioavailability of these endogenous EETs, which have various positive cardiovascular effects, including vasodilation and anti-thrombotic action. Furthermore, sEH inhibitors demonstrate anti-inflammatory properties by enabling EETs to block pro-inflammatory signalling pathways, such as NF-κB, thereby diminishing systemic and vascular inflammation linked to cardiovascular disease and organ impairment.^16^ Consequently, dual COX-2/sEH inhibitors represent an innovative, multi-target strategy in medicinal chemistry aimed at enhancing therapeutic efficacy while reducing systemic toxicity in various disorders, including inflammatory diseases.^6,17,18^ The most effective strategy has involved developing dual COX-2/sEH inhibitors (such as the investigational medication PTUPB) or the concurrent delivery of distinct sEH and COX-2 inhibitors. This strategy facilitates effective pain and inflammatory relief (*via* COX-2 inhibition) while offering organ protection and reducing cardiovascular risk (*via* sEH inhibition).^13,14,19^

Lipoxygenase (LOX), on the other hand, is an enzyme responsible for converting arachidonic acid (AA) from cell membranes into leukotriene.^20,21^ The combined inhibition of COX-2 and 5-LOX is expected to impede the synthesis of prostaglandins (PGs) *via* the COX pathway and leukotrienes (LTs) through the LOX pathway.^22^ These mediators of the inflammatory process are derived from the same arachidonic acid (AA) route. Thus, this could provide an appealing strategy for advancing novel anti-inflammatory medicines with a verified safety profile.

On the other hand, cancer has emerged as a predominant cause of premature mortality globally, with approximately 20 million new cases and 9.7 million cancer-related fatalities documented in 2022.^23^ Chronic inflammatory processes facilitate the initiation and progression of cancer by increasing the concentrations of eicosanoid intermediates, such as prostaglandins (PG) and leukotrienes (LT), produced by the metabolism of arachidonic acid (AA) catalyzed by COX-2 and 5-LOX.^20^ COX-2 and 5-LOX enzymes are overexpressed in various cancer types, including colon,^24^ ovarian,^25^ and lung^26^ cancers, among others. These intermediates yield substantial effects that facilitate tumor proliferation. The intermediates generated by 5-LOX (LTC4, LTD4, LTE4, and 5-HETE) enhance cell proliferation and reduce apoptosis in cancer cells,^27^ whereas prostaglandin E2 (PGE2), synthesized by COX-2, facilitates immunosuppression, promotes cell proliferation, metastasis, and inhibits apoptosis.^28^ Given these findings, the development of molecules with anti-inflammatory properties holds great promise for cancer prevention and treatment.^29–31^

**Fig. 1:**
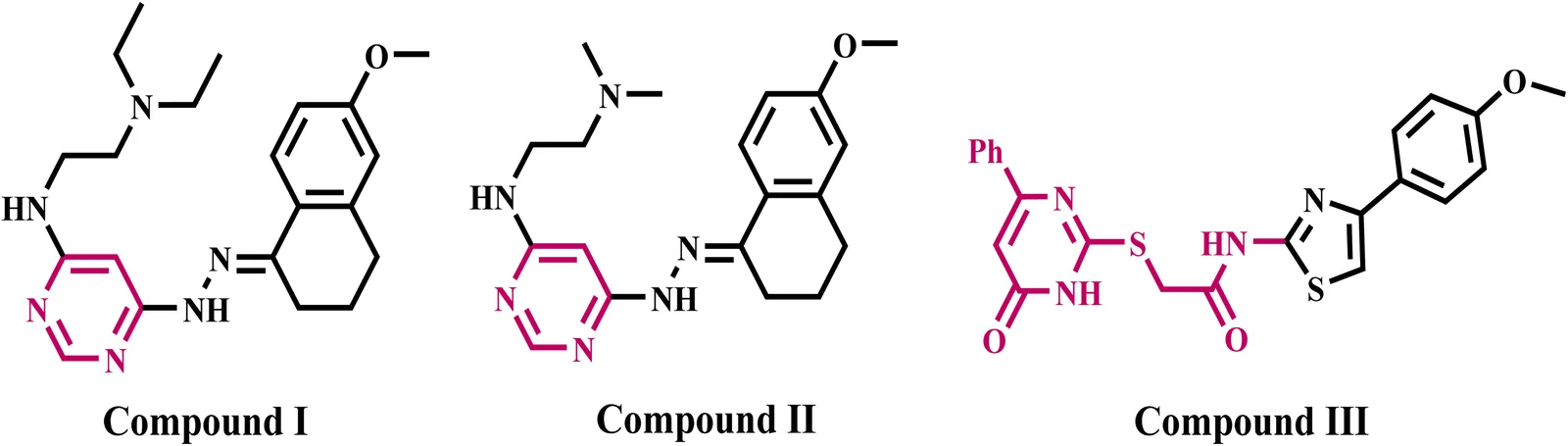
Structures of some reported pyrimidine-based compounds I–III.

The pyrimidine ring has been recognized as a significant scaffold for anti-inflammatory activity.^14,32,33^ Tylińska *et al.*^33^ described the synthesis of a novel class of pyrimidine-based derivatives that are selective COX-2 inhibitors with anticancer properties. Among the investigated pyrimidine derivatives, compounds I and II (Fig. 1) exhibited high selectivity towards COX-2, surpassing piroxicam and obtaining results comparable to meloxicam. In the sulforhodamine B (SRB) assay, compounds I and II exhibited dose-dependent inhibition of LPS-induced THP-1 cell growth. Furthermore, ROS studies indicated that these compounds reduced free radical levels, confirming their antioxidant properties.

Previously, we reported a novel class of pyrimidine-based selective COX-2/sEH inhibitors that exhibit analgesic and anti-inflammatory properties and reduce cardiotoxicity.^14^ Compound III (Fig. 1) had the highest COX-2 inhibitory activity and was identified as the most efficient combined COX-2/sEH inhibitor. *In vivo* assays demonstrated that compound III exhibited the highest efficacy as an analgesic and anti-inflammatory agent, along with enhanced ulcerogenic and cardioprotective attributes.

**Fig. 2:**
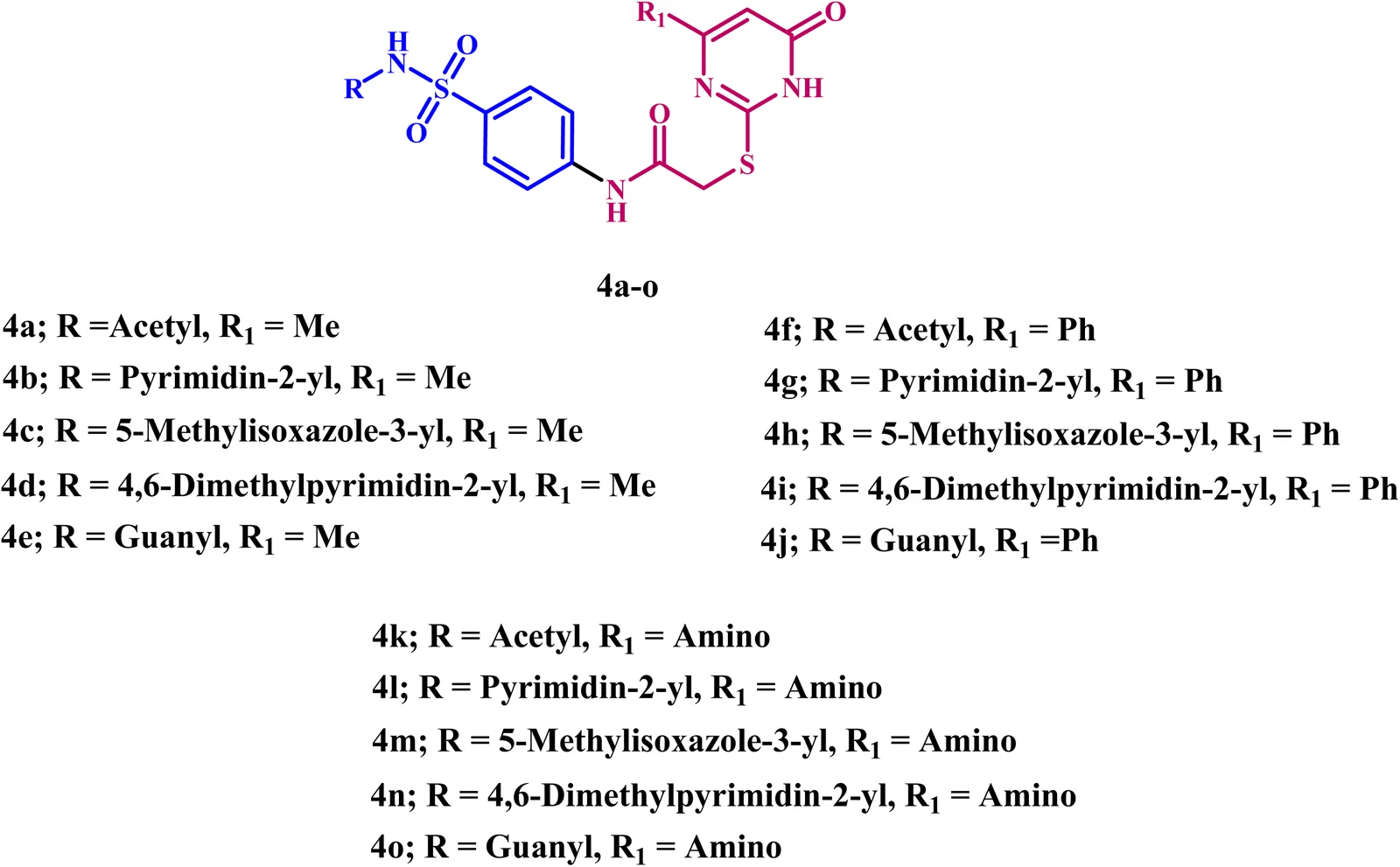
Structures of new compounds 4a–o.

Given the side effects of traditional NSAIDs and the need for new drugs suitable for polypharmacological approaches, the objective of this study was to develop a series of novel pyrimidine-based derivatives (4a–o) (Fig. 2) and investigate their multi-COX-2/5-LOX/sEH inhibition and anticancer proficiencies.

Development of multitarget inhibitors for the inflammatory cascade and as antiproliferative agents is a strategic advantage over giving combined single-target inhibitors. This is because it allows for synchronized modulation of the COX-2, 5-LOX, and sEH pathways while avoiding the pharmacokinetic problems and possible drug–drug interactions that come with polypharmacy.

### 1.1. Rational design

The multi-target compounds 4a–o (Fig. 2) were rationally designed to potentially impact the COX-2, 5-LOX, and sEH simultaneously in the arachidonic acid cascade. In order to systematically map the steric, electrical and lipophilic requirements within the extremely complex catalytic domains of these three enzymes, three structurally different R_1_ substituents, *i.e.* methyl, phenyl and amino groups were incorporated into the core framework of 4a–o. The small, aliphatic methyl group (compounds 4a–e) was chosen to investigate small, localized hydrophobic sub-pockets with minimum steric hindrance. However, the bulky, planar phenyl ring (compounds 4f–j) was added to investigate the scaffold's ability to engage in extended aromatic networks or pi–pi stacking interactions with conserved aromatic residues, such as tyrosine or phenylalanine, within the larger binding cavities. Finally, the hydrophilic amino group (compounds 4k–o) was introduced to investigate potential electrostatic interactions or hydrogen-bonding networks with polar amino acids, acting as a strong hydrogen-bond donor or acceptor to balance the scaffold's overall lipophilicity.

This comprehensive structural design permits compounds like 4c to be regarded as a multi-pharmacophoric hybrid, with a 5-methylisoxazole sulfonamide moiety coupled to a methyl substituted thiouracil core *via* a flexible thioether–acetamido linker. The benzenesulfonamide motif, particularly when incorporated with a heteroaryl ring such as 5-methylisoxazole, is a well-established pharmacophoric anchor designed to bind to COX-2's selective hydrophilic side-pocket.

The central thiouracil core possesses both structural and functional significance. The stiff pyrimidine-based structure determines the optimal spatial configuration and alignment of the adjacent pharmacophoric components. The inserted carbonyl and amide (NH) groups in the thiouracil framework act as bioisosteric substitutes for traditional urea or amide inhibitors that must accommodate the catalytic triad of sEH, while the molecule's extended volume is designed to obstruct access to the 5-LOX active site. The hybrid structure meets the topographical criteria for concurrent cascade inhibition, linking these particular modules to effectively regulate the clinical manifestations of the leukotriene shunt while mitigating cardiovascular side effects.

## 2. Experimental

### 2.1. General details

See Appendix A (SI file).

Compounds 2a–e (ref. 34) and 3a–f (ref. 35) were prepared according to reported procedures.

#### 2.1.1. General procedure for the synthesis of amino-benzenesulfonamide derivatives (2a–e)

A mixture of sulfanilamides 1a–e (10 mmol), chloroacetyl chloride (1.35 g, 12 mmol), and anhydrous potassium carbonate (3.30 g, 24 mmol) in dry DMF (20 mL) was stirred overnight at ambient temperature. The reaction mixture was transferred into 150 mL of distilled water and acidified with acetic acid. The resultant solid was filtered and subsequently washed with water (2 × 50 mL). The isolated solid was crystallized from ethanol.

#### 2.1.2. General procedure for the synthesis of 6-oxo-pyrimidinyl-2-yl-thioacetamido-benzenesulfonamides (4a–o)

A mixture consisting of compounds 2a–e (1 mmol), thiouracil derivatives 3a–b (1 mmol), and triethylamine (0.2 g, 2 mmol) in 5 mL of DMF, containing a few potassium iodide crystals, was heated at 80 °C for 2 hours. The reaction mixture was cooled, transferred into 30 mL of distilled water, and acidified with acetic acid. The resultant solid was filtered and subsequently washed with water (2 × 50 mL). The isolated solid was crystallized from ethanol.

##### 2.1.2.1 *N*-Acetyl-4-(1,6-dihydro-4-methyl-6-oxopyrimidin-2-yl-thio)acetamido-benzene-sulfonamide (4a)

Yellow crystals; yield, 78%; mp, 210–2.212 °C; ^1^H NMR (500 MHz, DMSO-*d*_6_) *δ* 12.00 (bs, 1H, Pyrm-NH), 10.69 (s, 1H, Ar-NH), 7.82–7.80 (d, *J* = 8.6 Hz, 2H, Ar–H), 7.74–7.72 (d, *J* = 8.7 Hz, 2H, Ar–H), 5.94 (s, 1H, Pyrm-C5-H), 4.06 (s, 2H, SCH_2_); 2.05 (s, 3H, CH_3_), 1.86 (s, 3H, CH_3_). ^13^C NMR (126 MHz, DMSO-*d*_6_) *δ* 171.70, 169.21, 167.36, 143.90, 133.72, 129.48, 119.19, 35.73, 23.72, 19.07. EI-MS (*m*/*z*) calcd for C_15_H_16_N_4_O_5_S_2_: 396.06. Found: 397.89 [M + 2H]^+^, anal. calcd % for C_15_H_16_N_4_O_5_S_2_: C, 45.44; H, 4.07; N, 14.13. Found %: C, 45.52; H, 4.00; N, 14.21.

##### 2.1.2.2 *N*-(Pyrimidin-2-yl)-4-(1,6-dihydro-4-methyl-6-oxopyrimidin-2-yl-thio)acetamido-benzenesulfonamide (4b)

Yellow crystals; yield, 65%; mp, 246 °C (decomposition); ^1^H NMR (500 MHz, DMSO-*d*_6_) *δ* 11.94 (bs, 1H, Pyrm-NH), 10.61 (s, 1H, Ar-NH), 8.46–8.45 (d, *J* = 4.8 Hz, 2H), 7.91–7.89 (d, *J* = 8.4 Hz, 2H), 7.71–7.69 (d, *J* = 8.4 Hz, 2H), 7.10–6.99 (s, 1H, Pyrm'-C5-H), 5.96 (s, 1H, Pyrm-C5-H), 4.05 (s, 2H, SCH_2_), 2.05 (s, 3H, CH_3_). ^13^C NMR (126 MHz, DMSO-*d*_6_) *δ* 167.26, 158.89, 158.76, 157.48, 143.29, 134.83, 129.48, 119.01, 116.30, 112.65, 35.45, 23.89. EI-MS (*m*/*z*) calcd for C_17_H_16_N_6_O_4_S_2_: 432.07. Found: 431.76 [M]^+^, anal. calcd % for C_17_H_16_N_6_O_4_S_2_: C, 47.21; H, 3.73; N, 19.43. Found %: C, 47.30; H, 3.65; N, 19.34.

##### 2.1.2.3 *N*-(5-Methylisoxazole-3-yl)-4-(1,6-dihydro-4-methyl-6-oxo-pyrimidin-2-yl-thio)acetamido benzenesulfonamide (4c)

Yellow crystals; yield, 68%; mp, 196–198 °C; ^1^H NMR (500 MHz, DMSO-*d*_6_) *δ* 12.16 (bs, 1H, Pyrm-NH), 10.65 (s, 1H, Ar-NH), 7.78–7.72 (dd, *J* = 24.5, 8.6 Hz, 4H, Ar–H), 6.09 (s, 1H, isoxazole-H), 5.96 (s, 1H, Pyrm-C5-H), 4.06 (s, 2H, SCH_2_), 2.25 (s, 3H, CH_3_), 2.06 (s, 3H, CH_3_). ^13^C NMR (126 MHz, DMSO-*d*_6_) *δ* 170.81, 167.35, 158.11, 143.65, 133.93, 128.67, 119.47, 95.93, 95.89, 35.70, 23.74, 12.57. Anal. calcd % for C_17_H_17_N_5_O_5_S_2_: C, 46.89; H, 3.93; N, 16.08. Found %: C, 46.80; H, 4.02; N, 16.17.

##### 2.1.2.4 *N*-(4,6-Dimethylpyrimidin-2-yl)-4-(1,6-dihydro-4-methyl-6-oxo-pyrimidin-2-yl-thio)acetamido-benzenesulfonamide (4d)

Brown crystals; yield, 80%; mp, 200–202 °C; ^1^H NMR (500 MHz, DMSO-*d*_6_) *δ* 12.17 (bs, 1H, Pyrm-NH), 10.57 (s, 1H, Ar-NH), 7.91–7.90 (d, *J* = 8.7 Hz, 2H, Ar–H), 7.69–7.67 (d, *J* = 8.7 Hz, 2H, Ar–H), 6.72 (s, 1H, Pyrm-C5-H), 5.96 (s, 1H, Pyrm-C5-H), 4.05 (s, 2H, SCH_2_), 2.21 (s, 6H, Pyrm-(CH_3_)_2_), 2.05 (s, 1H, CH_3_). ^13^C NMR (126 MHz, DMSO-*d*_6_) *δ* 167.20, 156.74, 143.04, 135.37, 129.88, 118.69, 114.34, 35.66, 23.85, 23.37. EI-MS (*m*/*z*) calcd for C_19_H_20_N_6_O_4_S_2_: 460.1. Found: 460.02 [M]^+^, anal. calcd % for C_19_H_20_N_6_O_4_S_2_: C, 49.55; H, 4.38; N, 18.25. Found %: C, 49.62; H, 4.30; N, 18.33.

##### 2.1.2.5 *N*-Guanyl-4-(1,6-dihydro-4-methyl-6-oxo-pyrimidin-2-yl-thio)acetamido-benzenesulfonamide (4e)

Red crystals; yield, 84%; mp, 248–250 °C; ^1^H NMR (500 MHz, DMSO-*d*_6_) *δ* 12.70 (bs, 1H, Pyrm-NH), 10.51 (s, 1H, Ar-NH), 7.67–7.63 (m, 4H, Ar–H), 6.75–6.63 (m, 4H, guanidine-NHs), 5.94 (s, 1H, Pyrm-C5-H), 4.06 (s, 2H, SCH_2_), 2.08 (s, 3H, CH_3_). ^13^C NMR (126 MHz, DMSO-*d*_6_) *δ* 166.99, 158.60, 141.77, 139.60, 127.24, 127.18, 119.10, 35.69, 23.72. Anal. calcd % for C_14_H_16_N_6_O_4_S_2_: C, 42.41; H, 4.07; N, 21.20. Found %: C, 42.33; H, 4.16; N, 21.13.

##### 2.1.2.6 *N*-Acetyl-4-(1,6-dihydro-4-phenyl-6-oxo-pyrimidin-2-yl-thio)acetamido-benzenesulfonamide (4f)

Yellow crystals; yield, 68%; mp, 242–248 °C; ^1^H NMR (500 MHz, DMSO-*d*_6_) *δ* 12.35 (bs, 1H, Pyrm-NH), 10.83 (s, 1H, Ar-NH), 7.95–7.93 (d, *J* = 8.0 Hz, 2H, Ar–H), 7.85–7.83 (d, *J* = 8.9 Hz, 2H, Ar–H), 7.80–7.78 (d, *J* = 8.9 Hz, 2H, Ar–H), 7.36–7.34 (t, *J* = 7.3 Hz, 1H, Ar–H), 7.21–7.18 (t, *J* = 7.7 Hz, 2H, Ar–H), 6.65 (s, 1H, Pyrm-C5-H), 4.16 (s, 2H, SCH_2_), 1.87 (s, 3H, CH_3_). ^13^C NMR (126 MHz, DMSO-*d*_6_) *δ* 169.32, 167.28, 144.03, 136.34, 133.79, 131.08, 129.53, 129.06, 127.42, 119.06, 35.89, 23.79. Anal. calcd % for C_20_H_18_N_4_O_5_S_2_: C, 52.39; H, 3.96; N, 12.22. Found %: C, 52.46; H, 4.02; N, 12.28.

##### 2.1.2.7 *N*-(Pyrimidin-2-yl)-4-(1,6-dihydro-4-phenyl-6-oxo-pyrimidin-2-yl-thio)acetamido-benzenesulfonamide (4g)

Yellow crystals; yield, 68%; mp, 256 °C (decomposition); ^1^H NMR (500 MHz, DMSO-*d*_6_) *δ* 12.51 (s, 1H, Pyrm-NH), 10.79 (s, 1H, Ar-NH), 8.46–8.45 (d, *J* = 4.4 Hz, 2H, Ar–H), 7.94–7.88 (dd, *J* = 19.6, 7.9 Hz, 4H, Ar–H), 7.78–7.77 (d, *J* = 8.3 Hz, 1H, Ar–H), 7.68–7.59 (dd, *J* = 34.4, 7.5, 1H, Ar–H), 7.50–7.45 (dd, *J* = 20.2, 6.8 Hz, 1H, Ar–H), 7.25–7.22 (m, 1H, Ar–H), 7.12–7.09 (m, 1H, Ar–H), 7.00–6.99 (d, *J* = 4.4 Hz, 1H, Pyrm-C5-H), 6.63–6.54 (m, 1H, Pyrm-C5-H), 4.14 (s, 2H, SCH_2_). ^13^C NMR (126 MHz, DMSO-*d*_6_) *δ* 167.19, 161.55, 158.87, 157.49, 153.49, 143.49, 136.37, 134.71, 129.94, 129.37, 128.94, 127.41, 118.85, 116.28, 112.71, 35.88. EI-MS (*m*/*z*) calcd C_22_H_18_N_6_O_4_S_2_: 494.08. Found: 494.04 [M]^+^, anal. calcd % for C_22_H_18_N_6_O_4_S_2_: C, 53.43; H, 3.67; N, 16.99. Found %: C, 53.50; H, 3.58; N, 16.90.

##### 2.1.2.8 *N*-(5-Methylisoxazole-3-yl)-4-(1,6-dihydro-4-phenyl-6-oxo-pyrimidin-2-ylthio)acetamido-benzenesulfonamide (4h)

Brown crystals; yield, 70%; mp, 244–246 °C; ^1^H NMR (500 MHz, DMSO-*d*_6_) *δ* 12.80 (s, 1H, Pyrm-NH), 11.31 (s, 1H, Ar-NH), 10.82 (s, 1H, sulphur-NH), 7.91–7.90 (d, *J* = 7.2 Hz, 2H), 7.79 (s, 4H, Ar–H), 7.31–7.29 (m, 1H, Ar–H), 7.15–7.14 (d, *J* = 6.7 Hz, 2H, Ar–H), 6.65 (s, 1H, Pyrm-C5-H), 6.09 (s, 1H, isoxazole-H), 4.15 (s, 2H, SCH_2_), 2.24 (s, 1H, CH_3_). ^13^C NMR (126 MHz, DMSO-*d*_6_) *δ* 170.77, 167.26, 158.09, 143.86, 136.35, 133.75, 131.01, 128.98, 128.74, 127.59, 127.42, 119.34, 95.92, 95.87, 35.89, 12.57. Anal. calcd % for C_22_H_19_N_5_O_5_S_2_: C, 53.11; H, 3.85; N, 14.08. Found %: C, 53.04; H, 3.80; N, 14.18.

##### 2.1.2.9 *N*-(4,6-Dimethylpyrimidin-2-yl)-4-(1,6-dihydro-4-phenyl-6-oxo-pyrimidin-2-ylthio)acetamido-benzenesulfonamide (4i)

Brown crystals; yield, 78%; mp, 236–238 °C; ^1^H NMR (500 MHz, DMSO-*d*_6_) *δ* 12.44 (s, 1H, Pyrm-NH), 10.83 (s, 1H, Ar-NH), 7.93–7.91 (dd, *J* = 5.9, 1.6 Hz, 4H, Ar–H), 7.77–7.70 (dd, *J* = 9.0, 5.0 Hz, 2H, Ar–H), 7.22–7.18 (d, *J* = 7.2 Hz, 1H, Ar–H), 7.12–7.08 (m, 2H, Ar–H), 6.71–6.70 (d, *J* = 4.3 Hz, 1H, Pyrm'-C5-H), 6.63 (s, 1H, Pyrm-C5-H); 4.15 (s, 2H, SCH_2_), 2.18 (d, *J* = 5.2 Hz, 6H, Pyrm-(CH_3_)_2_). ^13^C NMR (126 MHz, DMSO-*d*_6_) *δ* 167.88, 167.13, 156.73, 143.26, 136.33, 135.12, 131.05, 130.85, 129.92, 128.91, 127.45, 118.49, 35.87, 23.39. EI-MS (*m*/*z*) calcd C_24_H_22_N_6_O_4_S_2_: 522.11. Found: 521.98 [M]^+^, anal. calcd % for C_24_H_22_N_6_O_4_S_2_: C, 55.16; H, 4.24; N, 16.08. Found %: C, 55.22; H, 4.20; N, 16.00.

##### 2.1.2.10 *N*-Guanyl-4-(1,6-dihydro-4-phenyl-6-oxo-pyrimidin-2-ylthio)acetamido-benzenesulfonamide (4j)

Red crystals; yield, 70%; mp, 250−252 °C; ^1^H NMR (500 MHz, DMSO-*d*_6_) *δ* 12.72 (bs, 1H, Pyrm-NH), 10.67 (s, 1H, Ar-NH), 7.96–7.95 (d, *J* = 7.6 Hz, 2H, Ar–H), 7.69 (s, 4H, Ar–H), 7.39–7.36 (t, *J* = 7.0 Hz, 1H, Ar–H), 7.23–7.20 (t, *J* = 7.1 Hz, 2H, Ar–H), 6.65 (s, 5H, guanidine-NHs, Pyrm-C5-H), 4.15 (s, 2H, SCH_2_). ^13^C NMR (126 MHz, DMSO-*d*_6_) *δ* 166.91, 158.60, 141.97, 139.47, 136.34, 131.15, 129.07, 127.47, 127.22, 118.95, 35.57. EI-MS (*m*/*z*) calcd C_19_H_18_N_6_O_4_S_2_: 458.08. Found: 458.50 [M]^+^. Anal. calcd % for C_19_H_18_N_6_O_4_S_2_: C, 49.47; H, 3.96; N, 18.33. Found %: C, 49.41; H, 3.90; N, 18.26.

##### 2.1.2.11 *N*-Acetyl-4-(4-amino-1,6-dihydro-6-oxo-pyrimidin-2-yl-thio)acetamido-benzenesulfonamide (4k)

Brown crystals; yield, 64%; mp, 180–182 °C; ^1^H NMR (500 MHz, DMSO-*d*_6_) *δ* 11.97 (s, 1H, Pyrm-NH), 10.66 (s, 1H, Ar-NH), 7.83–7.81 (d, *J* = 9.3 Hz, 2H, Ar–H), 7.78–7.77 (d, *J* = 9.3 Hz, 2H, Ar–H), 6.51 (s, 1H, Pyrm-C5-H), 3.98 (s, 2H, SCH_2_), 1.87 (s, 3H, CH_3_). ^13^C NMR (126 MHz, DMSO-*d*_6_) *δ* 169.38, 169.24, 143.88, 133.79, 129.43, 119.37, 119.31, 82.05, 35.21, 23.72. EI-MS (*m*/*z*) calcd C_14_H_15_N_5_O_5_S_22_: 397.05. Found: 396.82 [M]^+^. Anal. calcd % for C_14_H_15_N_5_O_5_S_2_: C, 42.31; H, 3.80; N, 17.62. Found %: C, 42.39; H, 3.84; N, 17.55.

##### 2.1.2.12 *N*-(Pyrimidin-2-yl)-4-(4-amino-1,6-dihydro-6-oxo-pyrimidin-2-ylthio)acetamido-benzenesulfonamide (4l)

Yellow crystals; yield, 62%; mp, 238 °C (decomposition); ^1^H NMR (500 MHz, DMSO-*d*_6_) *δ* 11.58 (bs, 1H, Pyrm-NH), 10.49 (s, 1H, Ar-NH), 8.47–8.46 (d, *J* = 4.7 Hz, 2H, Ar–H), 7.90–7.89 (d, *J* = 8.2 Hz, 2H, Ar–H), 7.72–7.66 (dd, *J* = 27.8, 7.9 Hz, 2H, Ar–H), 7.00 (s, 1H, Pyrm'-C5-H), 6.60–6.35 (m, 1H, Pyrm-C5-H), 3.95 (s, 2H, SCH_2_). ^13^C NMR (126 MHz, DMSO-*d*_6_) *δ* 167.78, 164.33, 159.18, 158.88, 157.45, 143.24, 134.90, 13.00, 129.59, 129.41, 119.47, 119.16, 116.33, 82.04, 35.21. EI-MS (*m*/*z*) calcd C_16_H_15_N_7_O_4_S_2_: 433.06. Found: 433.40 [M]^+^. Anal. calcd % for C_16_H_15_N_7_O_4_S_2_: C, 44.33; H, 3.49; N, 22.62. Found %: C, 44.41; H, 3.45; N, 22.67.

##### 2.1.2.13 *N*-(5-Methylisoxazole-3-yl)-4-(4-amino-1,6-dihydro-6-oxo-pyrimidin-2-yl-thio)acetamido-benzenesulfonamide (4m)

Yellow crystals; yield, 66%; mp, 218–220 °C; ^1^H NMR (500 MHz, DMSO-*d*_6_) *δ* 11.56 (bs, 1H, Pyrm-NH), 10.53 (s, 1H, Ar-NH), 7.79–7.72 (ddd, *J* = 14.1, 8.8, 0.9 Hz, 4H, Ar–H), 6.61–6.43 (m, 1H, Pyrm-C5-H), 6.08 (s, 1H, isoxazole-H), 3.96 (s, 2H, SCH_2_), 2.25 (s, 3H. CH_3_). ^13^C NMR (126 MHz, DMSO-*d*_6_) *δ* 170.77, 167.85, 158.20, 143.55, 134.03, 129.37, 128.60, 119.58, 113.14, 95.96, 95.90, 82.02, 35.20, 12.58. EI-MS (*m*/*z*) calcd C_16_H_16_N_6_O_5_S_2_: 436.06. Found: 435.64 [M]^+^. Anal. calcd % for C_16_H_16_N_6_O_5_S_2_: C, 44.03; H, 3.69; N, 19.25. Found %: C, 44.11; H, 3.61; N, 19.30.

##### 2.1.2.14 *N*-(4,6-Dimethylpyrimidin-2-yl)-4-(4-amino-1,6-dihydro-6-oxo-pyrimidin-2-yl-thio)acetamido-benzenesulfonamide (4n)

Brown crystals; yield, 64%; mp, 220–222 °C; ^1^H NMR (500 MHz, DMSO-*d*_6_) *δ* 11.48 (bs, 1H, Pyrm-NH), 10.45 (s, 1H, Ar-NH), 7.91–7.89 (d, *J* = 7.1 Hz, 2H, Ar–H), 7.69–7.68 (d, *J* = 5.9 Hz, 2H, Ar–H), 6.72 (s, 1H, Pyrm'-C5-H), 6.47 (s, 1H, Pyrm-C5-H), 3.94 (s, 2H, SCH_2_), 2.21 (s, 6H, Pyrm-(CH_3_)_2_). ^13^C NMR (126 MHz, DMSO-*d*_6_) *δ* 167.72, 164.35, 156.75, 142.91, 135.38, 129.81, 118.76, 82.03, 35.19, 23.40. Anal. calcd % for C_18_H_19_N_7_O_4_S_2_: C, 46.84; H, 4.15; N, 21.24. Found %: C, 46.89; H, 4.12; N, 21.30.

##### 2.1.2.15 *N*-Guanyl-4-(4-amino-1,6-dihydro-6-oxo-pyrimidin-2-yl-thio)acetamido benzenesulfonamide (4o)

Yellow crystals; yield, 76%; mp, 206–208 °C; ^1^H NMR (500 MHz, DMSO-*d*_6_) *δ* 11.95 (bs, 1H, Pyrm-NH), 10.37 (s, 1H, Ar-NH), 7.66–7.65 (d, *J* = 4.2 Hz, 4H, Ar–H), 6.65–6.51 (m, 5H, guanide-NHs + Pyrm-C5-H), 3.96 (d, *J* = 6.3 Hz, 2H, SCH_2_). ^13^C NMR (126 MHz, DMSO-*d*_6_) *δ* 167.63, 164.30, 158.60, 141.80, 139.70, 127.30, 119.19, 82.04, 35.21. EI-MS (*m*/*z*) calcd C_13_H_15_N_7_O_4_S_2_: 397.06. Found: 395.97 [M − H]^+^. Anal. calcd % for C_13_H_15_N_7_O_4_S_2_: C, 39.29; H, 3.80; N, 24.67. Found %: C, 39.33; H, 3.72; N, 24.63.

### 2.2. Biology

#### 2.2.1. COX-1/COX-2 inhibitory assay

The COX-1/COX-2 (human) Inhibitor Assay Kit^14,19^ was employed to evaluate the *in vitro* COX-1/COX-2 inhibitory activity of 4a–o. Appendix A (SI file) provides more information.

#### 2.2.2. 5-LOX inhibitory assay

Compounds 4c, 4g, 4j, 4m, and 4o were evaluated for their capacity to inhibit the lipoxygenase (LOX) enzyme using the LOX enzyme assay kit,^36^ with Zileuton as the reference standard. Refer to Appendix A (SI file) for additional information.

#### 2.2.3. Human sEH inhibitory assay

The inhibitory efficacy of compounds 4c, 4j, and 4o against the sEH enzyme was evaluated *in vitro* utilizing a cell-based assay kit,^13^ yielding IC_50_ values. Refer to Appendix A (SI file).

#### 2.2.4. Cell viability assay

The impact of compounds 4c, 4g, 4j, 4m, and 4o on cell viability was evaluated utilizing the human mammary gland epithelial normal cell line (MCF-10A). The MTT assay was conducted to assess the cell viability of 4c, 4g, 4j, 4m, and 4o following four days of incubation with MCF-10A cells.^37^ Refer to Appendix A (SI file) for additional information.

#### 2.2.5. Antiproliferative assay

The MTT assay was employed to assess the antiproliferative effects of compounds 4c, 4g, 4j, 4m, and 4o on four human cancer cell lines: HCT-116 (colon), PC-3 (prostate), MCF-7 (breast), and MDAMB-231 (pancreatic). The cell lines (either normal or cancer cell lines) were obtained from ATCC (American Type Cell Culture) *via* Holding company for biological products and vaccines (VACSERA), Cairo, Egypt. Doxorubicin, sorafenib, and celecoxib served as reference agents.^38^ Dose–response assays were conducted to ascertain the IC_50_ values for the novel compounds. The reported data were derived from a minimum of two independent experiments, each comprising three repetitions per concentration. Experimental details are provided in Appendix A (SI file).

## 3. Results and discussion

### 3.1. Chemistry

**Scheme 1:**
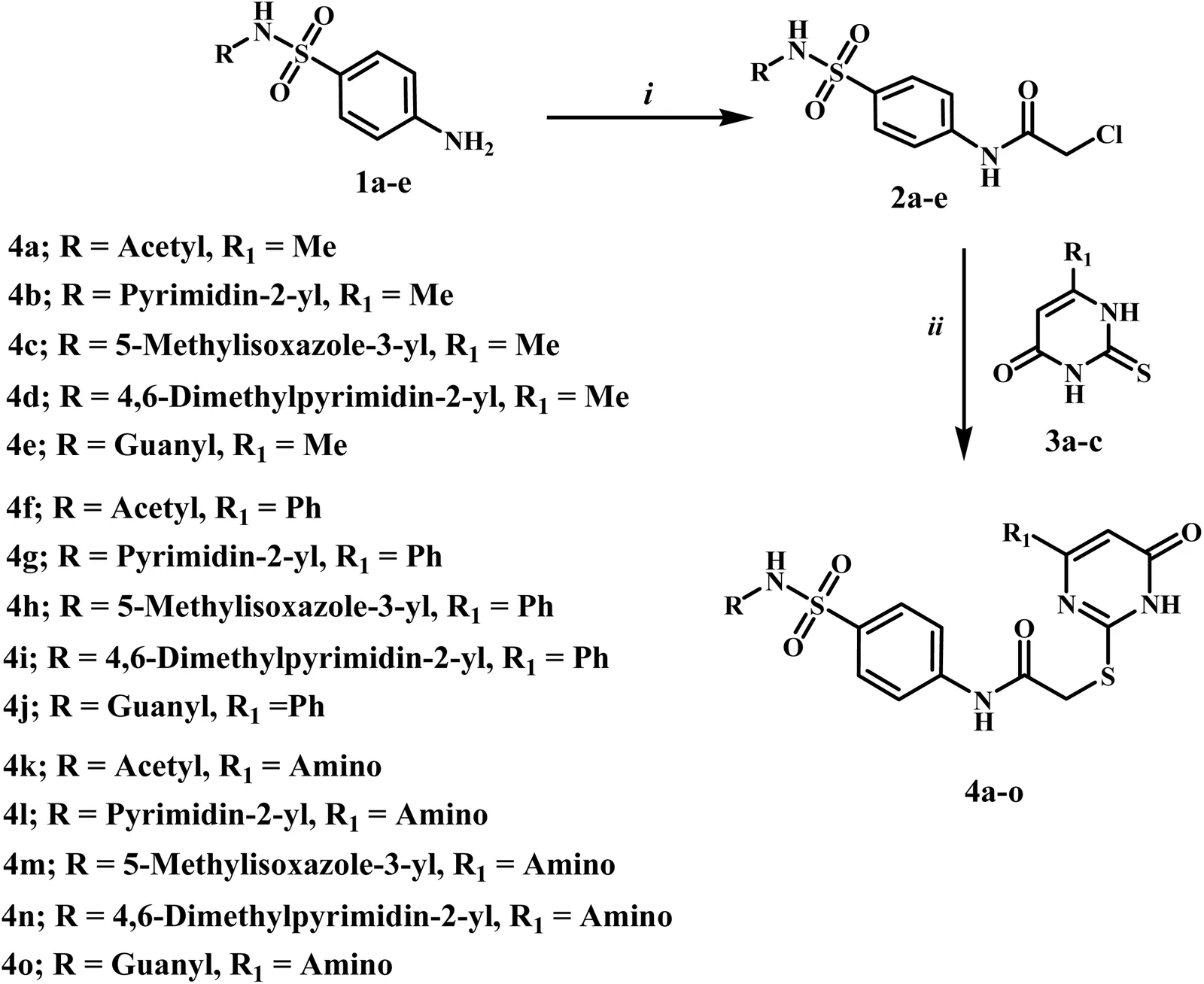
Synthesis of new compounds 4a–o. Reagents and conditions: (i) ClCH_2_COCl, DMF, K_2_CO_3_, ambient temperature, stirring overnight, 70–85% (ii) DMF, TEA, heat at 80 °C for 2 h, 62–84% yield.

Scheme 1 delineates the synthetic methodologies for the preparation of the key intermediates 2a–e and the final compounds 4a–o. The chloroacetyl sulfanilamides 2a–e were prepared from compounds 1a–e using a previously described method.^34^ The chloroacetylation of sulfanilamides 1a–e using 2-chloroacetyl chloride in anhydrous DMF with potassium carbonate as an acid scavenger yielded 2a–e in high yields (85%) along with confirmed melting points. Compounds 2a–e were then reacted with thiouracils 3a–c (ref. 35) using triethylamine and a catalytic amount of potassium iodide, yielding the new sulfanilamide–thiouracils 4a–o (60–80% yield).

The structures of compounds 4a–o were elucidated through ^1^H NMR, ^13^C NMR, EI-MS, and elemental microanalyses. The ^1^H NMR spectra of compounds 4a–o exhibited singlet signals (2H) within the range of *δ* 3.94–4.16 ppm, attributed to the CH_2_ groups, while the corresponding ^13^C NMR signals were observed at *δ* 35.13–35.87 ppm. Compounds 4a–e, which contain a methylthiouracil moiety, exhibited distinct singlet signals (3H) at approximately *δ* 2.05 ppm, attributed to the CH_3_ groups, alongside corresponding signals at *δ* 23.81 ppm in their ^13^C NMR spectra.

The ^1^H and ^13^C NMR spectra of 4a–e showed distinct signals at *δ* 5.94 ppm and *δ* 119 ppm, respectively, corresponding to the pyrimidine C5 proton and carbon. Compounds 4f–j, featuring a phenyl thiouracil moiety, and compounds 4k–o, which are derivatives based on amino thiouracil, exhibited distinct singlet signals near *δ* 6.5 ppm attributed to the pyrimidine–C5–H protons. Correspondingly, their ^13^C NMR signals were observed at approximately *δ* 116–119 ppm for 4f–j and nearly *δ* 82 ppm for the amino thiouracils 4k–o.

Compound 4c, representative of the series, features a 5-methylisoxazole moiety characterized by distinctive ^1^H NMR signals at 6.09 ppm (s, 1H, isoxazole-C4-H) and 2.25 ppm (s, 3H, isoxazole-CH_3_). The corresponding ^13^C NMR spectra displayed a doublet at *δ* 95.93–95.87 ppm and a signal at *δ* 12.8 ppm, assigned to the isoxazole C4^39^ and methyl carbons, respectively. Furthermore, EI-MS analysis of these compounds confirmed their structures, showing molecular ion peaks as [M]^+^, [M − H]^+^, or [M + 2H]^+^.

### 3.2. Biology

#### 3.2.1. *In vitro* cyclooxygenase (COX) inhibition assay

Compounds 4a–o were tested *in vitro* for their potential to inhibit COX-1 and COX-2 subtypes. The isozyme-specific inhibition was screened using a colorimetric enzyme immunoassay (EIA) kit.^14,19^ The potency of the tested compounds was determined by calculating the concentration required to achieve 50% enzyme inhibition (IC_50_). Furthermore, the COX-2 selectivity indexes (SI values), defined as IC_50_ (COX-1)/IC_50_ (COX-2), were calculated and compared with those of celecoxib, a reference drug. The findings are presented in Table 1.

**Table 1:** IC_50_ values of compounds 4a–o against COX-1, COX-2, 5-LOX, and sEH^a^

Compound	COX-1 (IC_50_ µM)	COX-2 (IC_50_ µM)	Selectivity index (SI)	5-LOX (IC_50_ µM)	sEH (IC_50_ µM)
4a	3.10 ± 0.13	7.40 ± 0.44	00.40	—	—
4b	2.90 ± 0.11	6.50 ± 0.40	00.45	—	—
4c	6.60 ± 0.31	0.11 ± 0.01	60.00	0.46 ± 0.02	4.30 ± 0.19
4d	16.50 ± 0.90	4.20 ± 0.17	03.92	—	—
4e	0.90 ± 0.04	4.30 ± 0.33	00.20	—	—
4f	5.91 ± 0.21	6.20 ± 0.37	00.95	—	—
4g	1.80 ± 0.01	0.90 ± 0.06	02.00	0.88 ± 0.04	—
4h	7.20 ± 0.28	5.92 ± 0.22	01.20	—	—
4i	11.30 ± 0.64	5.61 ± 0.29	02.00	—	—
4j	9.10 ± 0.41	0.28 ± 0.01	32.50	0.80 ± 0.03	7.40 ± 0.20
4k	4.90 ± 0.27	2.80 ± 0.12	01.75	—	—
4l	3.80 ± 0.19	2.50 ± 0.18	01.50	—	—
4m	1.30 ± 0.07	1.09 ± 0.07	01.20	0.93 ± 0.07	—
4n	12.70 ± 0.60	2.12 ± 0.10	06.00	—	—
4o	9.70 ± 0.50	1.38 ± 0.09	07.00	1.01 ± 0.07	8.40 ± 0.40
Indomethacin	1.11 ± 0.09	—	—	—	—
Celecoxib	7.32 ± 0.30	0.08 ± 0.004	91.50	—	19.70 ± 0.85
Zileuton	—	—		0.69 ± 0.03	—
AUDA	—	—	—	—	2.70 ± 0.08

a —: not determined, SI (selectivity index) = IC_50_ (COX-1)/IC_50_ (COX-2).

Table 1 demonstrates that compounds 4a–o exhibited moderate to weak inhibitory activity against COX-1, with IC_50_ values ranging from 0.90 to 16.50 µM. This contrasts with the reference drug, indomethacin, which has an IC_50_ of 1.11 µM. Except for compound 4e, all compounds were less potent than indomethacin as COX-1 inhibitors. Compound 4e (R = Guanyl, R_1_ = Me) exhibited the highest potency as a COX-1 inhibitor, with an IC_50_ value of 0.90 µM, comparable to the reference indomethacin (IC_50_ = 1.11 µM). Compound 4m (R = 5-methylisoxazole, R_1_ = NH_2_) exhibited the second highest COX-1 inhibitory activity, with an IC_50_ value of 1.30 µM, marginally less potent than the reference indomethacin. The remaining compounds demonstrated poor to moderate efficacy, being 3 to 15-fold less potent than indomethacin as COX-1 inhibitors.

Table 1 shows that compounds 4a–o exhibited significant inhibitory activity against the COX-2 isoform, demonstrating greater potency as COX-2 inhibitors (IC_50_ = 0.11–7.40 µM) than against COX-1 (IC_50_ = 0.90–16.50 µM), yielding COX-2 selectivity scores (SI) ranging from 0.20 to 60. In all instances, the recorded activities against COX-2 were inferior to that of celecoxib (IC_50_ = 0.08 µM). Compounds 4c, 4g, 4j, 4m, and 4o were the most effective COX-2 inhibitors, with IC_50_ values of 0.11, 0.90, 0.28, 1.09, and 1.38 µM, respectively. Compound 4c (R = 5-methylisoxazole, R_1_ = Me) has the highest potency as a COX-2 inhibitor, with an IC_50_ value of 0.11 µM and a selectivity index (SI) of 60. It is somewhat less efficient than the reference drug celecoxib, which has an IC_50_ value of 0.08 µM and a SI of 92.

The substitution pattern at the fourth position of the dihydropyrimidine moiety substantially influences the COX-2 inhibitory activity of compounds 4a–o (R_1_ group) as well as the substitution pattern on the nitrogen atom (R group) of the phenyl sulfamoyl moiety. For example, compounds 4a (R = acetyl, R_1_ = Me), 4b (R = dihydropyrimidine, R_1_ = Me), 4d (*R* = 4,6-dimethyl-dihydropyrimidine, R_1_ = Me), and 4e (R = guanyl, R_1_ = Me) exhibit identical structural characteristics to 4c but possess distinct substituents at the nitrogen atom of the phenyl sulfamoyl group. These compounds demonstrated a significant reduction in COX-2 inhibitory efficacy, with IC_50_ values of 7.40, 6.50, 4.20, and 4.30 µM, respectively, rendering them at least 38-fold less potent than 4c as COX-2 inhibitors.

Furthermore, compounds 4h (R = 5-methylisoxazole, R_1_ = Ph) and 4m (R = 5-methylisoxazole, R_1_ = NH_2_) differ from compound 4c by incorporating a phenyl and an amino group, respectively, in the fourth position of the dihydropyrimidine moiety, as opposed to the methyl group present in 4c. Compounds 4h and 4m demonstrated IC_50_ values of 5.92 and 1.09 µM, respectively, with a selectivity index of 1.02, compared to an IC_50_ value of 0.11 µM for 4c and a selectivity index of 60. The observations suggest that both methyl and amino groups at the fourth position of the dihydropyrimidine moiety are acceptable for COX-2 inhibitory activity.

Compound 4j (R = guanyl, R_1_ = Ph) had the second highest COX-2 inhibitory activity, with an IC_50_ value of 0.28 µM and a selectivity index of 32.50, demonstrating a potency that is 2.5-fold less than that of 4c and 3.5-fold less than celecoxib as a COX-2 inhibitor. Compounds 4g (R = dihydropyrimidine, R_1_ = Ph), 4m (*R* = 5-methylisoxazole, R_1_ = NH_2_), and 4o (R = guanyl, R_1_ = NH_2_) had significant COX-2 inhibitory activity with IC_50_ values of 0.90, 1.09, and 1.39 µM, respectively; nevertheless, they displayed poor selectivity index (SI) values of 2, 1.20, and 7, respectively.

*In vitro* COX-1 and COX-2 inhibitory assays indicate that compounds 4c and 4j are promising selective COX-2 inhibitors.

#### 3.2.2. *In vitro* 5-LOX assay

Compounds 4c, 4g, 4j, 4m, and 4o, identified as the most effective COX-2 inhibitors, were assessed for their potential to inhibit the lipoxygenase (LOX) enzyme utilizing the LOX enzyme assay kit, with Zileuton serving as ref. 36. The acquired data is presented in Table 1. The outcomes of this *in vitro* experiment correspond with the *in vitro* COX-1/COX-2 assays. Compounds 4c, 4g, 4j, 4m, and 4o demonstrated significant 5-LOX inhibitory activity, with IC_50_ values of 0.46, 0.88, 0.80, 0.93, and 1.01 µM, respectively, compared with the reference compound Zileuton, which has an IC_50_ of 0.69 µM.

Compound 4c, the most effective and selective COX-2 inhibitor, also exhibited the highest efficacy as a 5-LOX inhibitor, with an IC_50_ value of 0.46 µM, surpassing the reference Zileuton. Compound 4c exhibited 1.5-fold more potency than the standard Zileuton. Compound 4j rated second in efficacy as a 5-LOX inhibitor and as a COX-2 inhibitor. Compound 4j demonstrated an IC_50_ value of 0.80 µM, indicating it is 1.8 times less potent than 4c and 1.1 times less effective than Zileuton. These data indicated that compounds 4c and 4j are prospective dual inhibitors of COX-2 and 5-LOX.

#### 3.2.3. *In vitro* sEH assay

A cell-based assay experiment^13^ was employed to assess the inhibitory activity of compounds 4c, 4j, and 4o, the most selective COX-2 inhibitors, against the sEH enzyme *in vitro*. The IC_50_ values are presented in Table 1. Compared with the reference compound AUDA (IC_50_ = 2.70 µM), the evaluated compounds showed significant sEH suppression, with IC_50_ values ranging from 4.30 to 8.40 µM. The studied compounds were less potent than the standard AUDA but more effective than celecoxib as sEH inhibitors (IC_50_ = 19.70 µM).

Compound 4c, the most effective COX-2/5-LOX inhibitor, had the highest potency as a sEH inhibitor with an IC_50_ value of 4.30 µM, demonstrating 1.6-fold reduced potency compared to the reference AUDA, yet 5-fold greater potency than celecoxib as a sEH inhibitor. Compounds 4j and 4o had mild sEH inhibitory action with IC_50_ values of 7.40 and 8.40 µM, respectively; nonetheless, they outperformed celecoxib in sEH inhibition. These findings identify compound 4c as a selective and potent multi-targeted COX-2/5-LOX/sEH inhibitor.

#### 3.2.4. Cell viability assay

The effects of compounds 4c, 4g, 4j, 4m, and 4o on the viability of the human mammary gland epithelial cell line (MCF-10A) were evaluated to determine the safety of the synthesized compounds. The MTT assay was used to assess the cell viability of the new compounds after a four-day incubation with MCF-10A cells.^37^Table 2 shows that none of the evaluated compounds exhibited cytotoxicity in normal cells, with all maintaining cell viability above 89% at 50 µM.

**Table 2:** Cell viability% and antiproliferative assays of 4c, 4g, 4j, 4m, 4o, and celecoxib^a^

Compound	Cell viability%	*In vitro* cytotoxicity IC_50_ (µM)				
		HCT-116	MCF-7	Panc-1	PC-3	Mean IC_50_
4c	89	6.47 ± 0.4	5.05 ± 0.4	2.89 ± 0.1	12.95 ± 1.0	6.84
4g	90	8.67 ± 0.9	6.94 ± 0.4	4.80 ± 0.3	9.08 ± 0.8	7.37
4j	91	8.06 ± 0.7	3.78 ± 0.2	5.97 ± 0.3	7.48 ± 0.6	6.32
4m	91	17.31 ± 1.4	9.37 ± 0.7	8.51 ± 0.7	14.15 ± 1.2	12.33
4o	87	18.63 ± 1.5	9.68 ± 0.8	8.21 ± 0.7	16.22 ± 1.3	13.18
Celecoxib	—	9.25 ± 0.7	25.32 ± 1.6	30.06 ± 2.1	28.78 ± 1.8	23.35
Doxorubicin	—	5.23 ± 0.3	4.17 ± 0.2	3.18 ± 0.1	8.87 ± 0.6	5.36
Sorafenib	—	5.47 ± 0.3	7.26 ± 0.3	7.64 ± 0.4	11.53 ± 0.9	7.97

a —: not determined.

#### 3.2.5. Antiproliferative assay

The antiproliferative efficacy of compounds 4c, 4g, 4j, 4m, 4o, and celecoxib was assessed against four human cancer cell lines (colon – HCT-116, prostatic – PC-3, breast – MCF-7, and pancreatic – Panc-1) using the MTT assay.^38^ Doxorubicin and sorafenib were employed as controls in this investigation. Table 2 presents the median inhibitory concentration (IC_50_) and mean IC_50_ values for the four cancer cell lines.

The findings from this assay corresponded with those of the *in vitro* COX-2 and 5-LOX experiments. Compounds 4c, 4g, 4j, 4m, and 4o showed significant antiproliferative activity, with mean IC_50_ values ranging from 6.32 to 13.18 µM, compared with the mean IC_50_ of celecoxib at 23.35 µM. All investigated compounds were more potent than celecoxib but less potent than the reference doxorubicin, which had a mean IC_50_ value of 5.36 µM across the four cancer cell lines. Compounds 4c, 4g, and 4j exhibited greater potency than sorafenib, with mean IC_50_ values of 6.84, 7.37, and 6.32 µM, respectively, compared to sorafenib's mean IC_50_ value of 7.97 µM.

Compound 4c, the most effective derivative in all *in vitro* studies, had interesting antiproliferative activity with a mean IC_50_ value of 6.84 µM, surpassing the potency of celecoxib (mean IC_50_ = 23.35 µM) and sorafenib (mean IC_50_ = 7.97 µM). Compound 4c demonstrated 3.4-fold and 1.2-fold greater potency compared to celecoxib and sorafenib, respectively. Compound 4c exhibited the highest potency against the pancreatic (Panc-1) cancer cell line, with an IC_50_ value of 2.89 ± 0.1 µM, surpassing celecoxib by tenfold, sorafenib by 2.7-fold, and even exceeding the reference doxorubicin by 1.1-fold.

Compound 4j, positioned second in potency and selectivity against COX-2 and 5-LOX, had significant antiproliferative activity with a mean IC_50_ value of 6.32 µM, surpassing the potency of celecoxib (mean IC_50_ = 23.35 µM) and sorafenib (mean IC_50_ = 7.97 µM). Compound 4j had the highest potency against the MCF-7 breast cancer cell line, with an IC_50_ value of 3.78 ± 0.2 µM, surpassing celecoxib by 6.7-fold, sorafenib by 2-fold, and doxorubicin by 1.1-fold. Finally, compounds 4m and 4o had the lowest antiproliferative potency, with mean IC_50_ values of 12.33 and 13.18, respectively. They were more potent than celecoxib but less potent than doxorubicin and sorafenib.

These data identified compounds 4c and 4j as potent and selective inhibitors of COX-2, 5-LOX, and sEH, demonstrating antiproliferative effects superior to celecoxib and serving as potential lead compounds for future anticancer development targeting COX-2/5-LOX pathways with improved cardiovascular safety.

### 3.3. Docking studies

#### 3.3.1. Docking investigation within the active site of COX-2

Molecular docking studies were carried out using AutoDock 4.2 to evaluate hypothetical binding processes and structures of the target drugs in the COX-2 active region. To test the docking protocol's reliability and geometric fidelity, the native, co-crystallized ligand rofecoxib was re-docked into COX-2's active pocket. The self-redocked pose closely matched the orientation of the experimental crystal, with an RMSD of 0.198 Å and a binding energy score of −11.72 kcal mol^−1^. The extraordinarily low RMSD value significantly supports the prediction accuracy and conformational reliability of the docking parameters employed.

Compound 4c and the reference celecoxib were docked into the crystal structure of the COX-2 enzyme in association with the ligand inhibitor rofecoxib [PDB ID code 5KIR]^40^ at a resolution of 2.7 Å utilizing Autodock 4.2^41^ and Discovery Studio 2024.^42^ The most stable docking model was chosen based on the highest-scoring conformation. The docking results are presented in Table 3.

**Table 3:** Binding scores and amino acid interactions of 4c, celecoxib, and rofecoxib within the active COX-2

Compound	Binding score (*S* value) kcal mole^−1^	Interaction residues		
		H Bonding	Hydrophobic interaction	
			π–π, π–σ, π–sulfur, π–alkyl, alkyl interaction	van der Waals interaction
4c	−15.86	Arg513, His90, Tyr355, Gln192, Arg120, Ser353	His90, Leu531, Leu359, Met113, Val116, Val349, Ala527, Val523, Ala516, Ile517, Phe518, Leu352	Ile345, Leu117, Ser530, Gly519
Celecoxib	−13.42	Arg513, Ser353	Ser353, Leu352, Leu384, Trp387, Tyr385, Val523, Leu531, Ala527, Gly526, Val349, Leu359	Glyn192, Ala516, Ile517, Phe518, Met522, Ser530, Tyr348, Val116, Arg120, Tyr355, His90, Gly354, Phe381
Rofecoxib	−11.72	Arg513, His90, Ala527	Ser353, gly526, val349, Ala527, Leu352, Val523	Arg120, Tyr355, Leu359, Leu531, Ser530, Tyr348, Tyr385, Tyr387, Leu384, Met522, Ala516, Ile517, Phe518, Gly192

The literature indicates that the COX active binding site is divided into three regions: the carboxylate-binding region, the primary pocket region, and the side-pocket region. The carboxylate binding region contains three hydrophilic residues, Tyr355, Arg120, and Glu524, which form a hydrogen bond network at the entrance. Following the primary pocket, the entrance forms an elongated hydrophobic channel that extends further into the catalytic domain. The NSAID-binding site is composed of four amino acids: Tyr385, Trp87, Phe518, and Ser530. Val523 enlarges the lateral pocket above Arg120 and encompasses Arg513 and His90 residues, which are the principal residues accountable for COX-2 selectivity.^43^

**Fig. 3:**
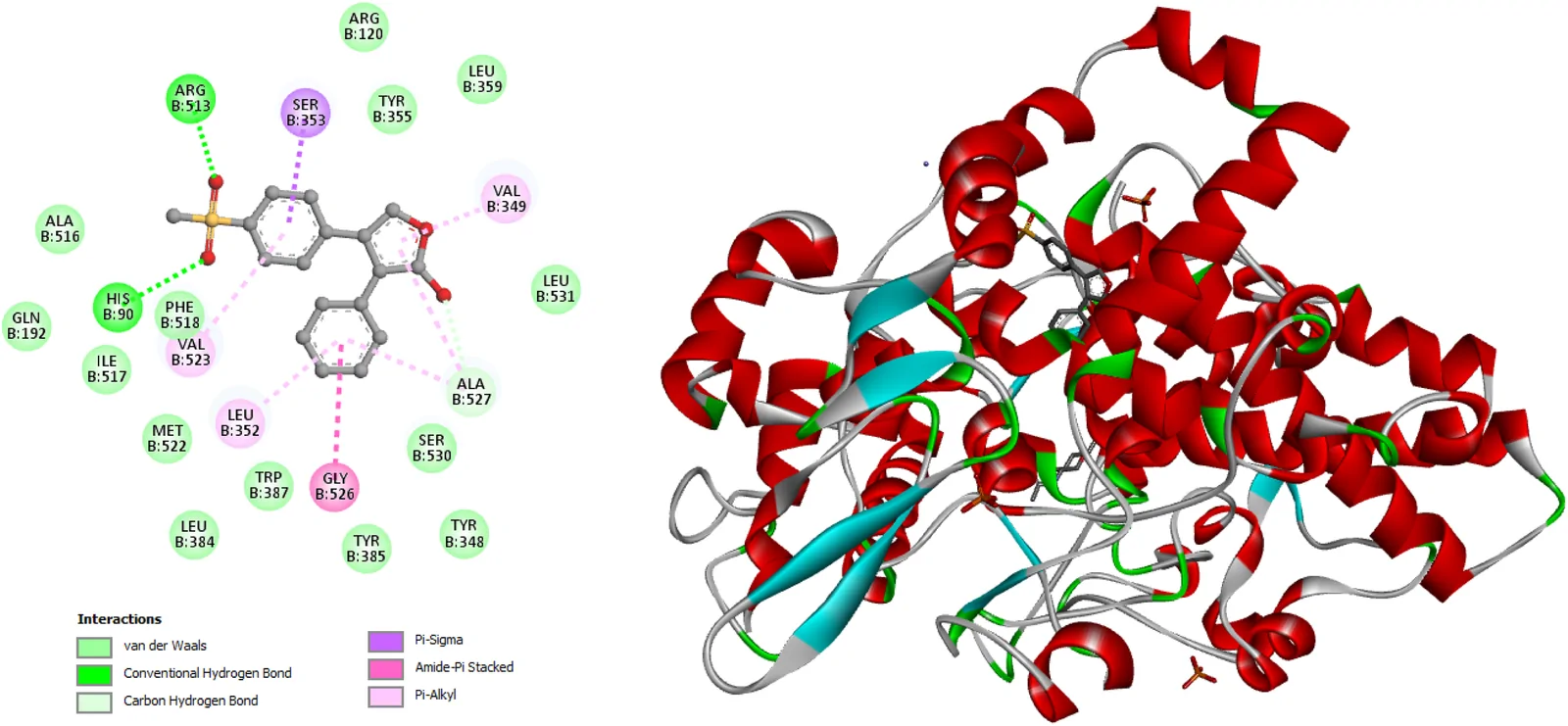
Docking models of rofecoxib within the COX-2 active site: 2D (left) and 3D (right).

Docking validation was performed by self-redocking co-crystallized rofecoxib into the COX-2 active site and comparing the docking pose to the initial pose using root-mean-square deviation (RMSD). Rofecoxib was docked in nearly the same position (RMSD = 0.198 Å) with a docking energy score of *S* = −11.72 kcal mol^−1^, exhibiting orientations similar to those previously documented.^44^ The methylsulfonyl moiety fits into the side pocket, where one methylsulfonyl oxygen atom forms a hydrogen bond with Arg513. In contrast, the other sulfonyl oxygen forms a separate hydrogen bond with His90, while the hydroxyl oxygen of 2-hydroxyfuran forms a carbon–hydrogen bond with Ala527. The remaining amino acids surrounding the pocket interact with the two phenyl and furan rings through a network of hydrophobic contacts, as shown in Table 3 and Fig. 3.

**Fig. 4:**
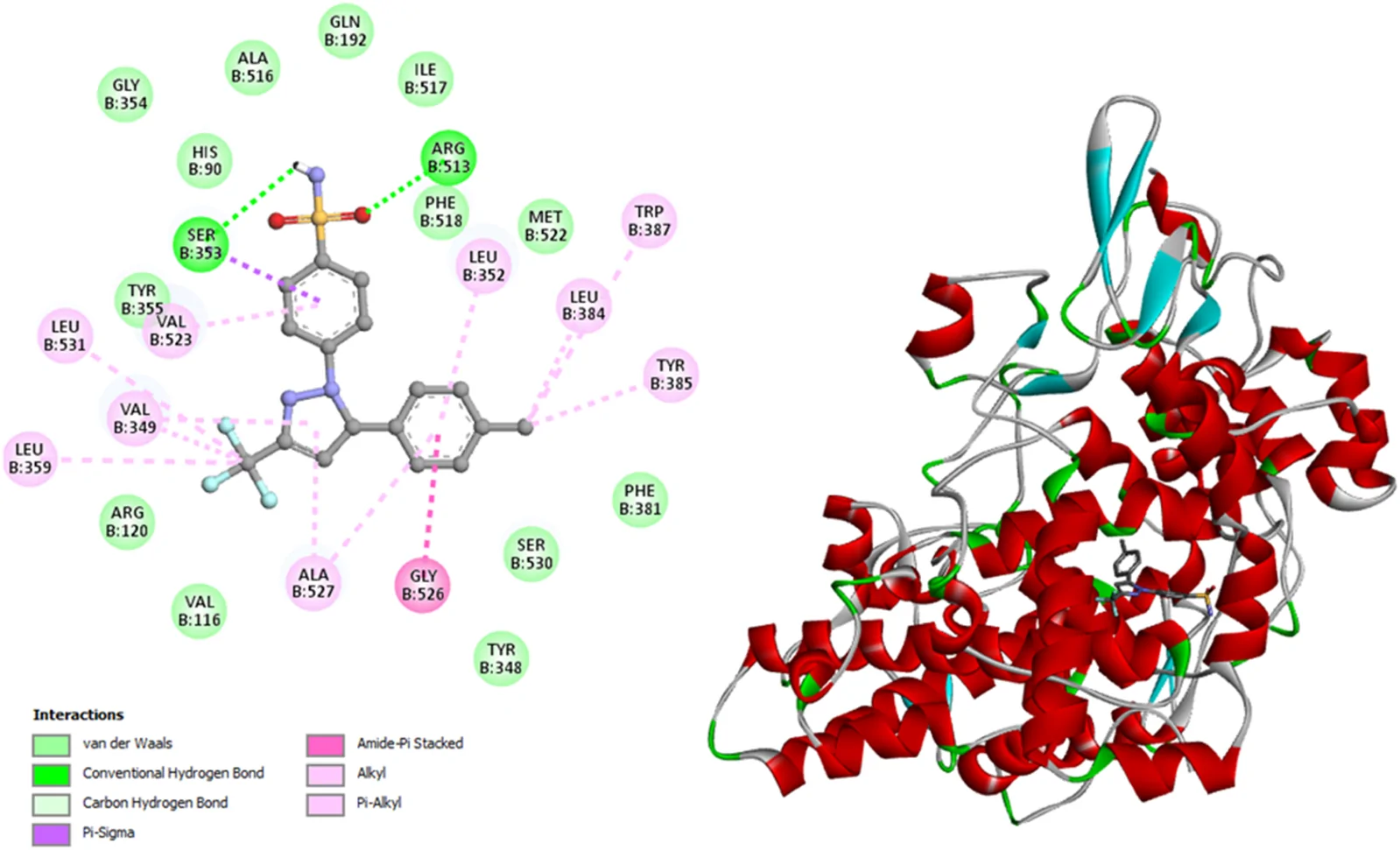
Docking models of celecoxib within the COX-2 active site: 2D (left) and 3D (right).

The molecular docking analysis of the reference drug, celecoxib, at the COX-2 binding site showed that it is docked and aligned parallel to rofecoxib, with a minimum binding energy of −13.42 kcal mol^−1^, compared to rofecoxib's −11.972 kcal mol^−1^. Both hydrophilic and hydrophobic interactions reinforced the binding interactions of celecoxib. Celecoxib forms two hydrogen interactions with Arg513 through one sulfonamide oxygen and with Ser353 *via* the sulfonamide amino group. The remaining amino acids around the pocket interact with the phenyl and alkyl substituents *via* a network of hydrophobic interactions, as shown in Table 2 and Fig. 4.

**Fig. 5:**
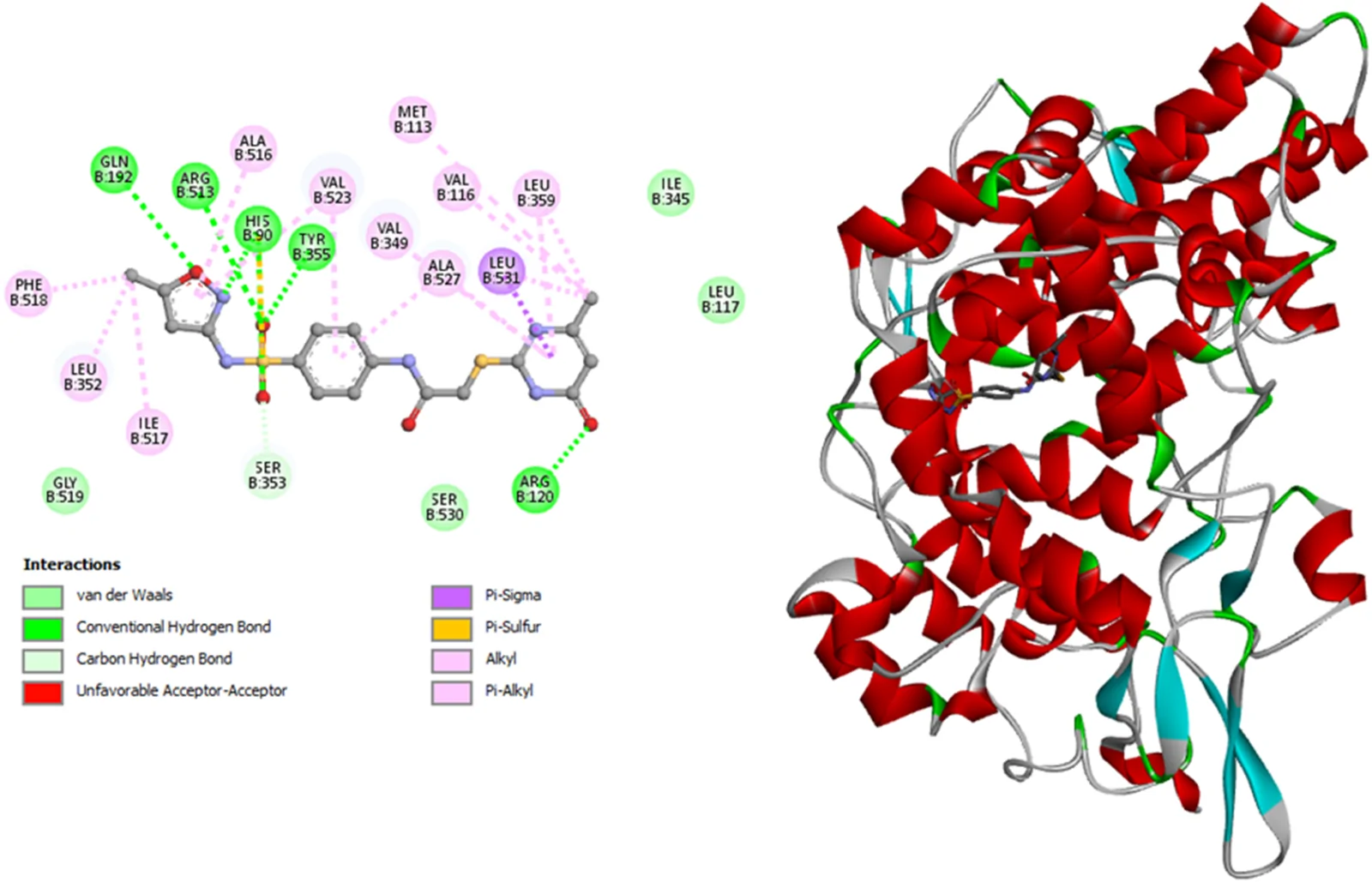
Docking models of 4c within the COX-2 active site: 2D (left) and 3D (right).

Docking experiments of compound 4c at the COX-2 binding site showed that 4c is docked and aligned parallel to rofecoxib, with a binding energy of −15.86 kcal mol^−1^, compared to the native inhibitor rofecoxib, which has a binding energy of −11.972 kcal mol^−1^. One sulfonyl oxygen of compounds 4c forms two hydrogen connections with Arg513 and Tyr355, while the other sulfonyl oxygen forms a hydrogen bond with His90 and a carbon–hydrogen bond with Ser353. Furthermore, the nitrogen of the isoxazole forms a hydrogen bond with His90, while the oxygen of the isoxazole forms a hydrogen bond with Gln192. Furthermore, the oxygen of 6-oxopyrimidine establishes a hydrogen bond with Arg120. The remaining amino acids around the pocket interact with the phenyl and alkyl substituents *via* a network of hydrophobic interactions, as shown in Table 2 and Fig. 5.

The favorable binding configurations align conceptually with the potent *in vitro* COX-2 inhibitory action exhibited by compound 4c. Nevertheless, a meticulous quantitative comparison of the absolute experimental (IC_50_) values of the newly synthesized hybrids with historical literature metrics for rofecoxib is rendered imprecise due to standard differences in independent assay settings, including enzyme supplies and incubation procedures. Nonetheless, the computational and experimental findings collectively affirm the structural feasibility of the hybrid framework to target the COX-2 enzyme.

#### 3.3.2. Docking analysis into the human sEH active site

A docking analysis was conducted to predict the potential interaction mode of the most active dual compound 4c within the active site of human sEH. The docking of compounds 4c and the reference sEH inhibitor AUDA into the crystal structure of the human sEH enzyme's catalytic domain, complexed with the ligand inhibitor 4-cyano-*N*-[(1*S*,2*R*)-2-phenylcyclopropyl] benzamide (PDB ID code 3ANS),^45^ was conducted. The docking results are presented in Table 4. The docking methodology was confirmed by self-redocking the co-crystallized ligand inhibitor into the sEH active site, and the docking pose was compared to the initial pose using RMSD. The ligand inhibitor was docked in nearly the same position (RMSD = 0.693 Å) and exhibited the same orientation as previously documented;^46^ the docking energy score was *S* = −12.84 kcal mol^−1^.

**Table 4:** Binding scores, amino acid interactions of 4c, AUDA, and ligand inhibitor within the active site of human sEH

Compound	Binding score (*S* value) kcal mole^−1^	Interaction residues		
		H Bonding	Hydrophobic interaction	
			π–π, π–σ, π–sulfur, π–alkyl, alkyl interaction	van der Waals interaction
4c	−13.22	Tyr383, Asp335, Tyr466 Gln384	Phe267, Leu499, Trp383, Met503, Ile363, Trp525, Met419, Val498	Phe381, His524, Leu408, Trp336, Thr360, Met469, Met339, Pro361
AUDA	−15.48	Tyr383, Asp335, Asn466, Ser374	Met469, Met339, Trp336, Leu408, Leu499, Trp525, Val498, His524	Phe381, Ile375, Pro371, Gln384, Thr360, Phe387, Met419, Leu428, Pro268, Phe267, Asn378
4-Cyano-*N*-[(1*S*,2*R*)-2-phenylcyclopropyl]benzamide ligand inhibitor	−12.84	Tyr383, Asp335, Tyr466	Tyr466, Phe387, Trp336, Val498, His524, Leu499	Phe381, Gln384, Met469, Trp525, Phe267, Pro268, Leu408, Leu428, Met419, Thr360, Met339

**Fig. 6:**
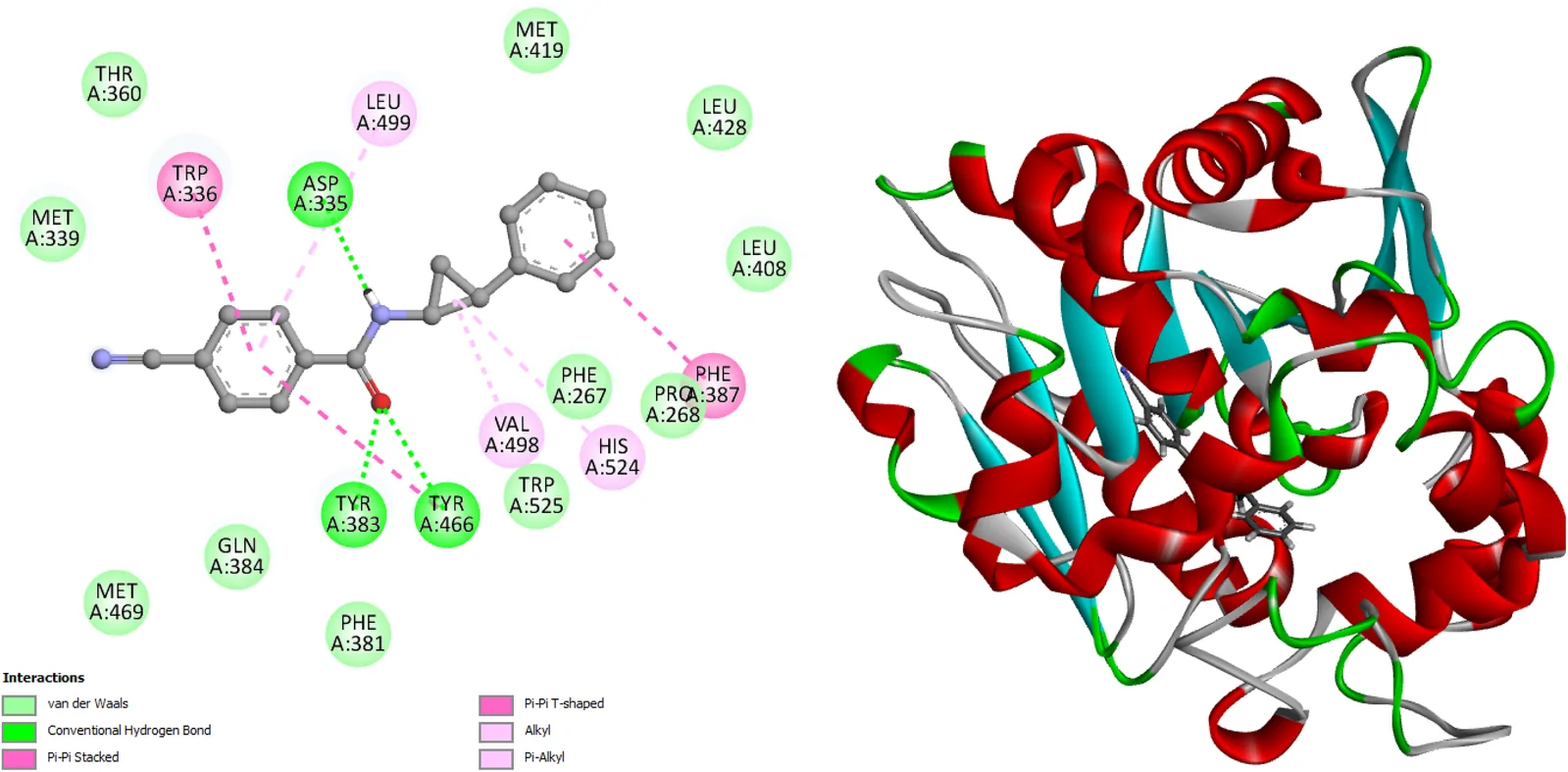
Docking models of the ligand inhibitor into the human sEH active site; 2D (left side) and 3D (right side).

The interactions between the ligand inhibitor and human sEH demonstrated that the amide oxygen forms two hydrogen bonds with Tyr466 and Tyr383, while the amide NH group establishes a hydrogen bond with Asp335. The remaining amino acids surrounding the pocket interact *via* a network of hydrophobic interactions, as illustrated in Table 4 and Fig. 6.

**Fig. 7:**
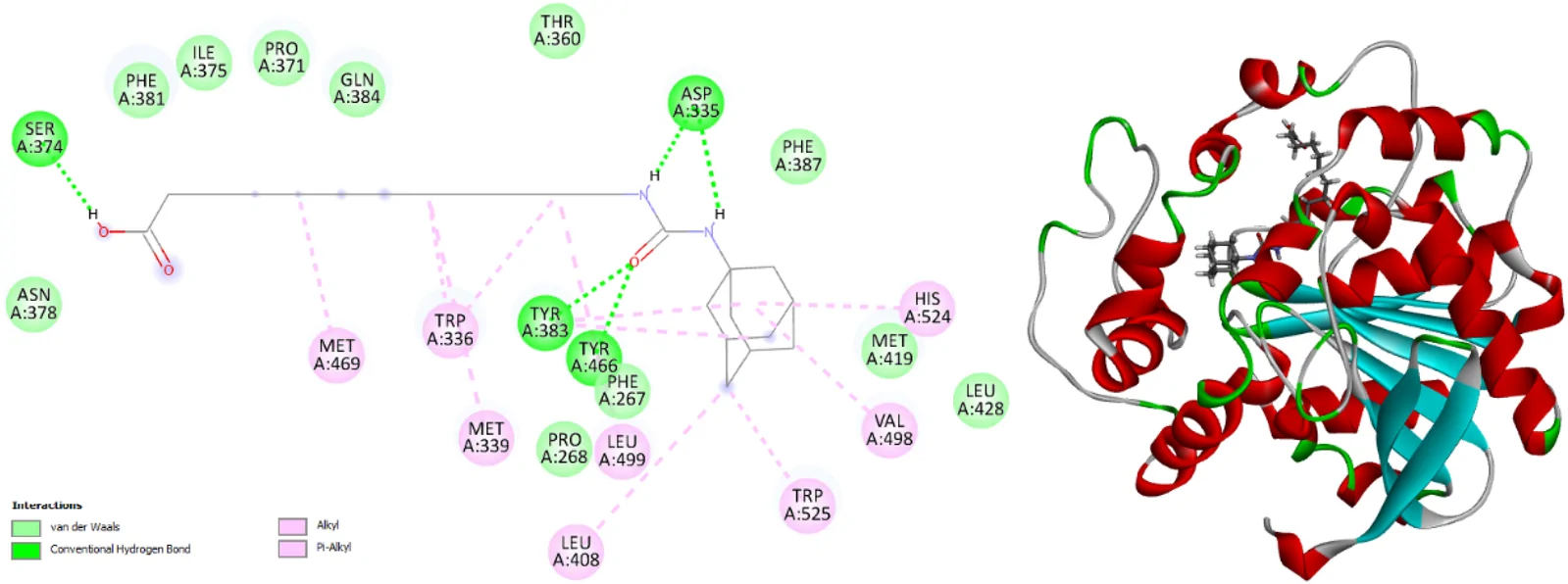
Docking models of AUDA within human sEH active site; 2D (left side) and 3D (right side).

The reference compound, AUDA, is well-suited to human sEH, with a docking score of *S* = −15.48 kcal mol^−1^. It demonstrates a similar orientation and binding affinity to the ligand inhibitor, in which the urea oxygen forms two hydrogen bonds with Tyr383 and Tyr466. In comparison, the urea amino groups form two hydrogen bonds with Asp335. The hydroxy carboxylate side chain also interacts with Ser374 *via* hydrogen bonding (Table 4 and Fig. 7).

**Fig. 8:**
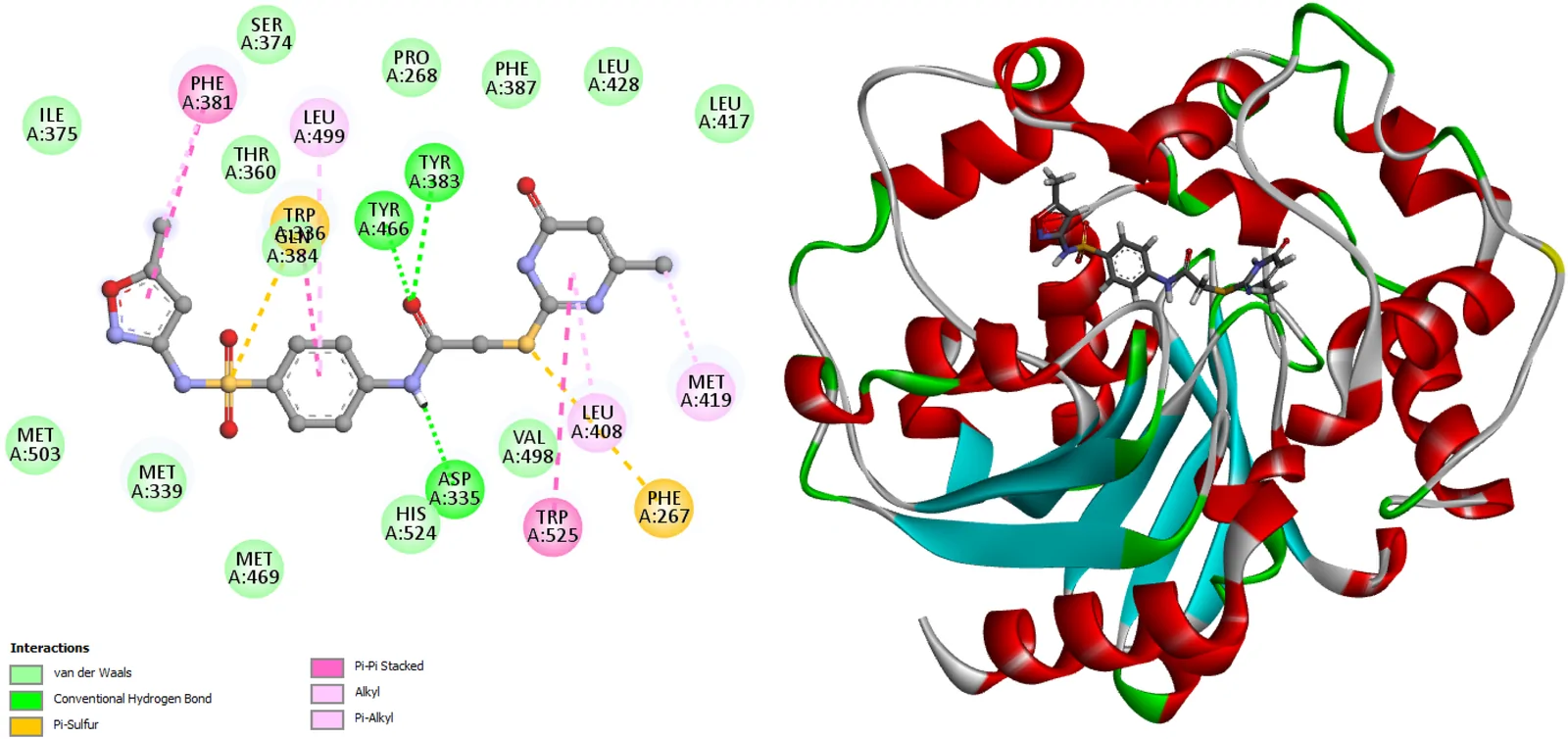
Docking models of 4c into human sEH active site; 2D (left side) and 3D (right side).

Compound 4c demonstrates a favorable compatibility with human sEH, with a docking score of *S* = −13.22 kcal mol^−1^. It demonstrates a similar orientation and binding affinity as the ligand inhibitor. The nitrogen atom of thioacetamide forms a hydrogen bond with Asp335, whereas the carbonyl group of thioacetamide engages in two hydrogen interactions with Tyr466 and Tyr383. One of the sulfonyl oxygens forms a hydrogen bond with Glyn384, as illustrated in Table 4 and Fig. 8.

#### 3.3.3. Docking study into 5-LOX active site

**Fig. 9:**
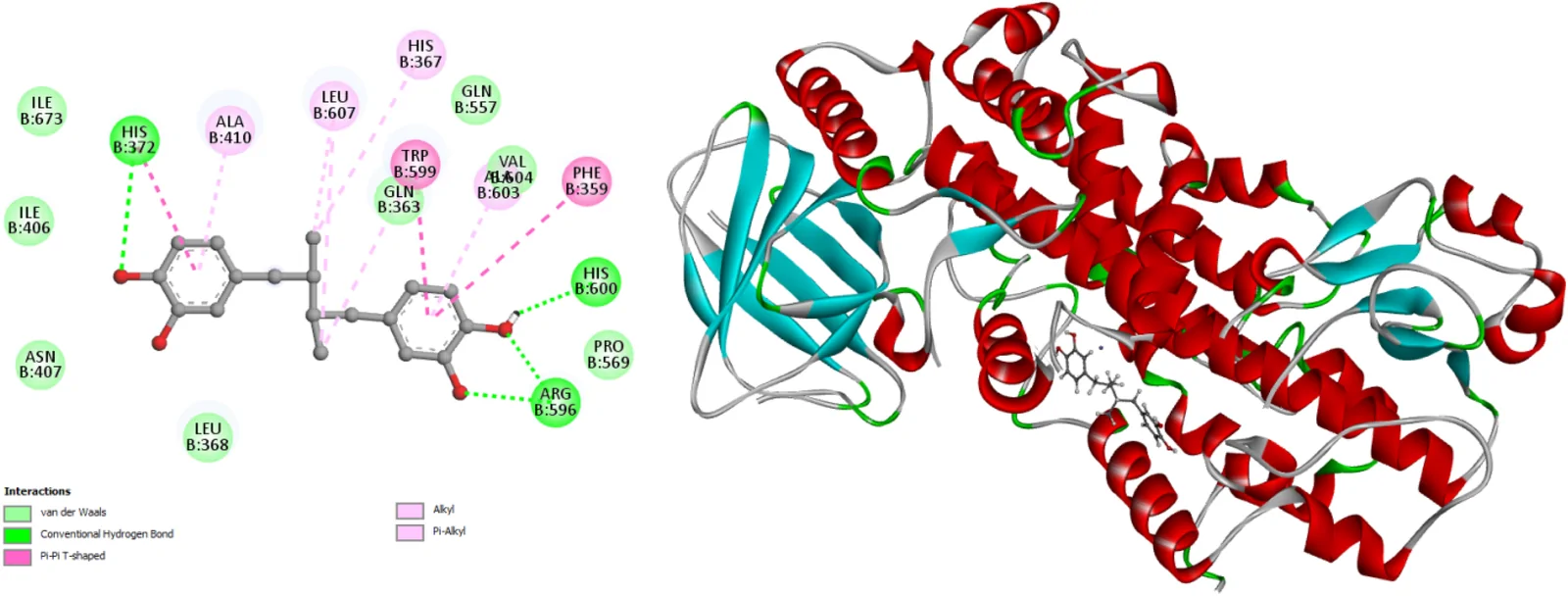
2D diagram representation of (left side) and 3D diagram representation (right side) of NDGA docked into human 5-LOX active site.

A docking study was performed to examine the potential binding interaction of the most active compound 4c within the active site of the 5-LOX enzyme, compared to the reference standard Zileuton. Docking of compound 4c into the crystal structure of the 5-LOX enzyme catalytic domain in association with the ligand inhibitor Nordihydroguaiaretic acid (NDGA) (PDB ID 6N2W).^47^ To validate the docking methodology, the NDGA co-crystallized conformer was redocked into the 5-LOX active site, and the docking pose was compared to the starting pose using root mean square deviation (RMSD). NDGA was docked nearly at the identical site (RMSD = 1.49 Å) and exhibited the similar orientations as previously documented;^47^ the docking energy score was *S* = −10.38 kcal mol^−1^. The interactions between NDGA and 5-LOX revealed that Arg596 and His600 both form two hydrogen bonds with the neighboring hydroxyl group proton and one oxygen in the catechol side chain, while His372 forms a hydrogen bond with an oxygen atom in the other catechol side chain. The alkyl moieties and phenyl rings interact with the remaining amino acids in the vicinity of the pocket through hydrophobic interactions, as seen in Fig. 9.

**Fig. 10:**
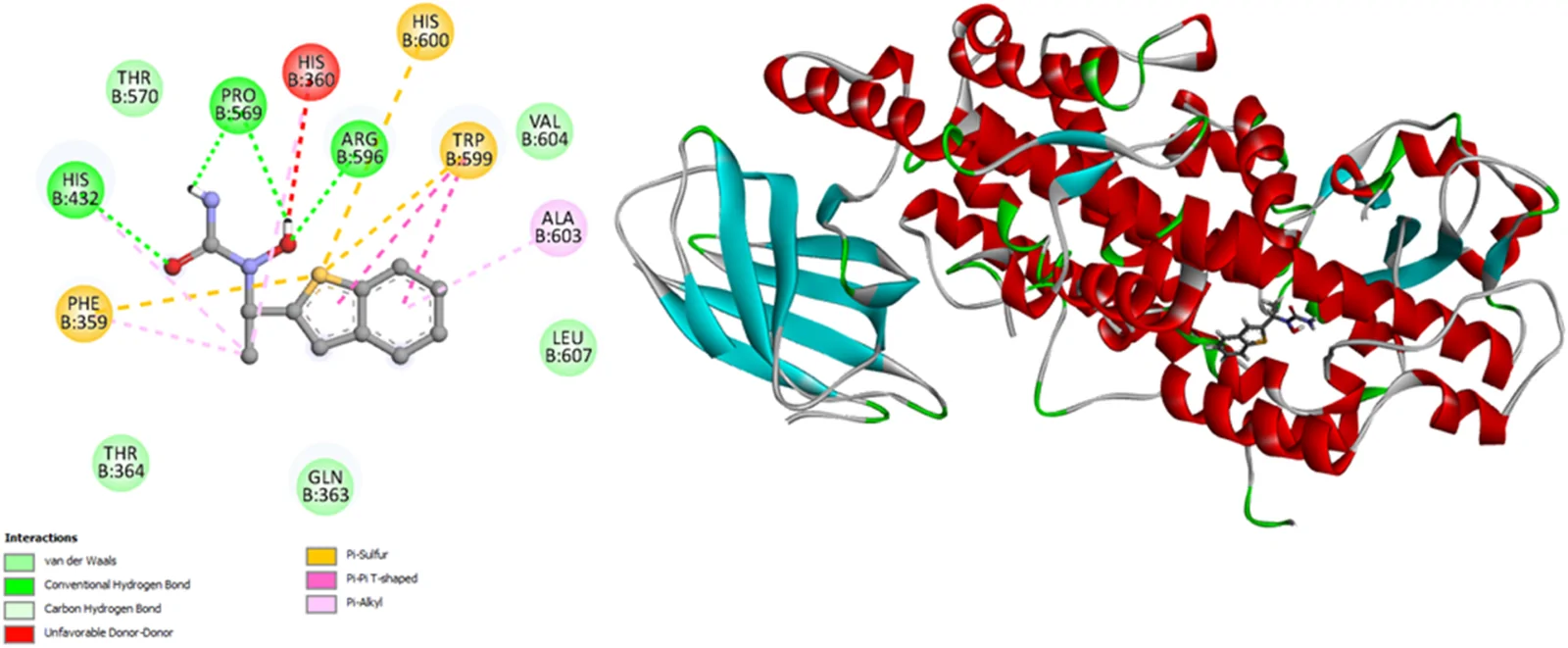
2D diagram representation of (left side) and 3D diagram representation (right side) of Zileuton docked into human 5-LOX active site.

The reference drug, Zileuton, demonstrates a favorable interaction with human 5-LOX, exhibiting a docking score of *S* = −8.78 kcal mol^−1^. It demonstrates a similar orientation and binding affinity as NDGA, wherein Arg596, Pro569, and His432 establish three hydrogen bonds with the hydroxyl proton of urea, the amino proton of urea, and the oxygen of urea, respectively. The remaining amino acids around the pocket engage in hydrophobic interactions with the alkyl moieties and benzothiophene ring, as illustrated in Fig. 10.

**Fig. 11:**
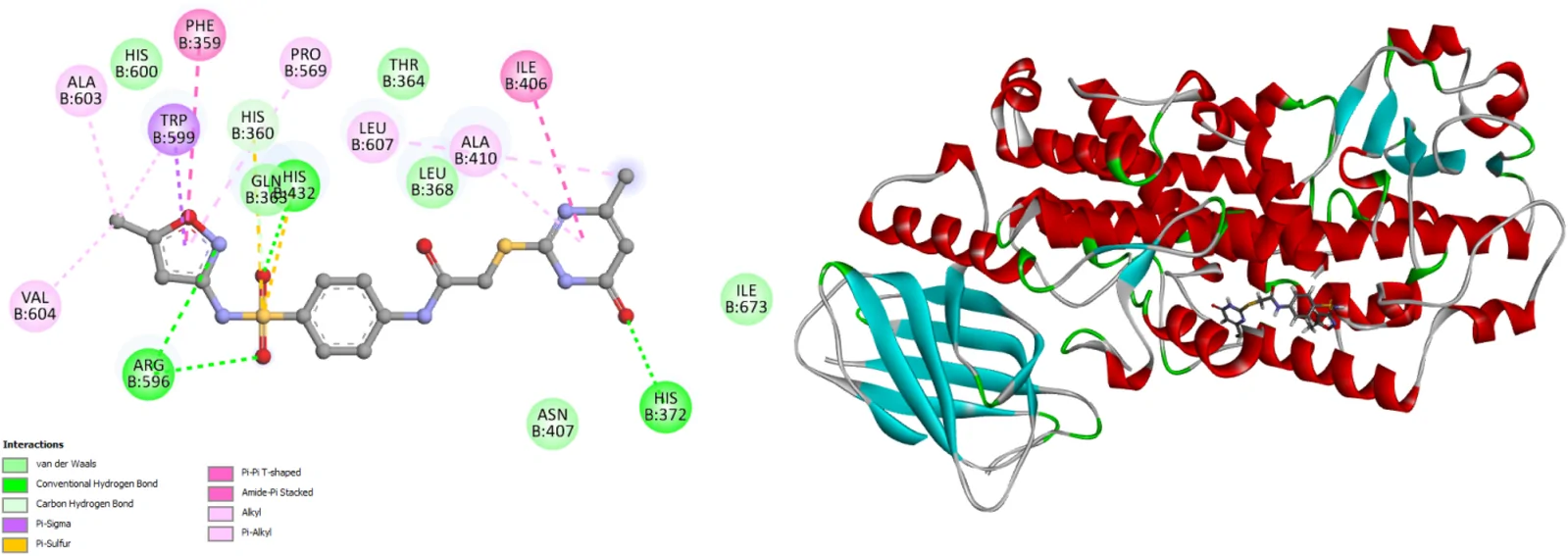
2D diagram representation of (left side) and 3D diagram representation (right side) of 4c docked into human 5-LOX active site.

Compound 4c exhibits a favorable compatibility with human 5-LOX, evidenced by a docking score of *S* = −11.22 kcal mol^−1^. It demonstrates a similar orientation and binding affinity as NDGA. The interactions of compound 4c with 5-LOX demonstrated that Arg596, His432, and His372 establish three hydrogen bonds with the isoxazole nitrogen, one sulfonamide oxygen, and 6-oxopyrimidine oxygen, respectively. The remaining amino acids surrounding the pocket engage in hydrophobic interactions with the alkyl and aryl substituents, as illustrated in Fig. 11.

### 3.4. Drug-likeness or ADME properties

The various criteria of Lipinski's and Veber's rules (drug-likeness or ADME properties)^48^ for the five most active tested compounds 4c, 4g, 4j, 4m, and 4o, were evaluated as potential drug candidates with enzyme inhibitory activity against COX-2, 5-LOX, and sEH. The assessed parameters comprised molecular weight (MW), number of hydrogen bond acceptors (HBA), number of hydrogen bond donors (HBD), number of rotatable bonds (*N*_rotb_), computed logarithm of the partition coefficient (*C* log *P*), and topological polar surface area (TPSA). The prediction of ADME properties may be valuable in anticipating the transport characteristics of the examined molecules across various membranes, including the blood–brain barrier and the gastrointestinal tract, where a molecule's failure to comply with two or more of the specified parameters would indicate a likelihood of poor bioavailability. As seen in Table 5, compounds 4c, 4g, 4j, and 4m adhere to Lipinski's Rule of Five, however compound 4o does not comply due to possessing more than 5 hydrogen bond donors (HBD) and more than 10 hydrogen bond acceptors (HBA). All five tested compounds do not comply with Veber's criterion due to possessing a TPSA greater than 140 Å^2^. The anticipated pharmacokinetic characteristics of the chosen drugs indicate minimal gastrointestinal absorption.

**Table 5:** Drug likeness examination illustrated through Lipinski's rule of five and Veber's rule for the tested compounds^a^

Compd.	MW (g mol^−1^)	HBA	HBD	Log Po/w (*C* log *P*)	Lipinski rule of 5 (Ro5)	*N*_rotb_	TPSA (Å^2^)	Veber role
4c	435.48	7	3	0.10	Yes; 0 violation	8	180.73	No, (TPSA > 140)
4g	494.55	7	3	0.59	Yes; 0 violation	9	180.48	No, (TPSA > 140)
4i	522.60	7	3	1.02	Yes; 1 violation: MW > 500	9	180.48	No, (TPSA > 140)
4m	436.47	7	4	−0.24	Yes; 0 violation	8	206.75	No, (TPSA > 140)
4o	397.43	6	6	−1.17	No; 2 violation: NorO > 10, NHorOH > 5	8	230.59	No, (TPSA > 140)

a MW: Molecular weight; HBA: H-bond acceptor; HBD: H-bond donor; *C* log *P*, calculated log of the partition coefficient; *N*_rotb_: number of rotatable bonds; TPSA, topological polar surface area.

Interestingly, the computed log *P* (1.104) of compound 4c indicates that the compound is reasonably hydrophilic, suggesting that its inadequate solubility in water is not attributable to excessive lipophilicity. The limited solubility is likely attributable to the elevated energy of the crystal lattice. The sulfonamide, amide, and pyrimidinone components of the molecule serve as robust hydrogen-bond donors and acceptors. This enables the solid state to possess dense intermolecular hydrogen-bonding networks. The structural stiffness and planarity of the aromatic systems frequently complicate their development, despite favorable log *P* values.

## 4. Conclusion

We developed a novel class of pyrimidine-derived compounds that selectively inhibit COX-2, 5-LOX, and sEH, demonstrating encouraging antiproliferative effects and anticipated enhancements in the safety profile, particularly regarding the cardiotoxicity associated with established COX-2 inhibitors. Compounds 4c and 4j were identified as the most potent and selective COX-2 inhibitors. Additionally, they were the most efficient derivatives as 5-LOX and sEH inhibitors. These compounds have the potential to be efficient antiproliferative agents with reduced cardiotoxicity, which is a major drawback of Coxibs. Molecular docking supported these findings by demonstrating favorable interactions with key residues within the COX-2, she, and 5-LOX active sites. Taken together, biological activity, mechanistic validation, and computational profiling strongly support compounds 4c and 4j as promising lead candidates for further optimization in the development of antiproliferative agents with potential anti-inflammatory activity and low cardiovascular side effects.

## Conflicts of interest

The authors declare no competing interests.

## Supplementary Material

RA-OLF-D6RA04113B-s001

## Data Availability

The authors declare that the data supporting the findings of this study are available within the supplementary information (SI). Supplementary information is available. See DOI: https://doi.org/10.1039/d6ra04113b.
