# Supplementary material for: Design, synthesis, and biological evaluation of novel pyrimidine–sulfonamide derivatives as multi-targeted COX-2/5-LOX/sEH inhibitors with potential antiproliferative properties
Source: RSC Adv. 2026 Aug 27;16(42):50806–21. doi: 10.1039/d6ra04113b (PMC13519979; doi:10.1039/d6ra04113b)
Supplement: Supplementary file 1 [file RA-016-D6RA04113B-s001.pdf]

Supplementary Information (SI) for RSC Advances.
This journal is © The Royal Society of Chemistry 2026

Design, synthesis and biological evaluation of novel pyrimidine-sulfonamide derivatives as multi-targeted COX-2/5-LOX/sEH inhibitors with potential antiproliferative properties

Lamya H. Al-Wahaibi[1], Alshaimaa Abdelmoez[2], Stefan Bräse[3]*, Salah A. Abdel-Aziz[4], Bahaa G.M. Youssif[2]*

[1]Department of Chemistry, College of Sciences, Princess Nourah bint Abdulrahman University, Riyadh 11671, Saudi Arabia; [2]Department of Pharmaceutical Organic Chemistry, Faculty of Pharmacy, Assiut University, Assiut 71526, Egypt; [3]Institute of Biological and Chemical Systems, IBCS-FMS, Karlsruhe Institute of Technology, 76131 Karlsruhe, Germany; [4]Department of Pharmaceutical medicinal Chemistry, Faculty of Pharmacy, Al-Azhar University, Assiut 71524, Egypt.

To whom correspondence should be addressed:

Bahaa G. M. Youssif, Ph.D. Pharmaceutical Organic Chemistry Department, Faculty of Pharmacy, Assiut University, Assiut 71526, Egypt.

Tel.: (002)-01098294419

E-mail address: bgyoussif2@gmail.com

Stefan Bräse

Institute of Biological and Chemical Systems, IBCS-FMS, Karlsruhe Institute of Technology, 76131 Karlsruhe, Germany. E-mail: braese@kit.edu

Supplementary Data

Fig. S1 : ^{1}H NMR of compound 4a

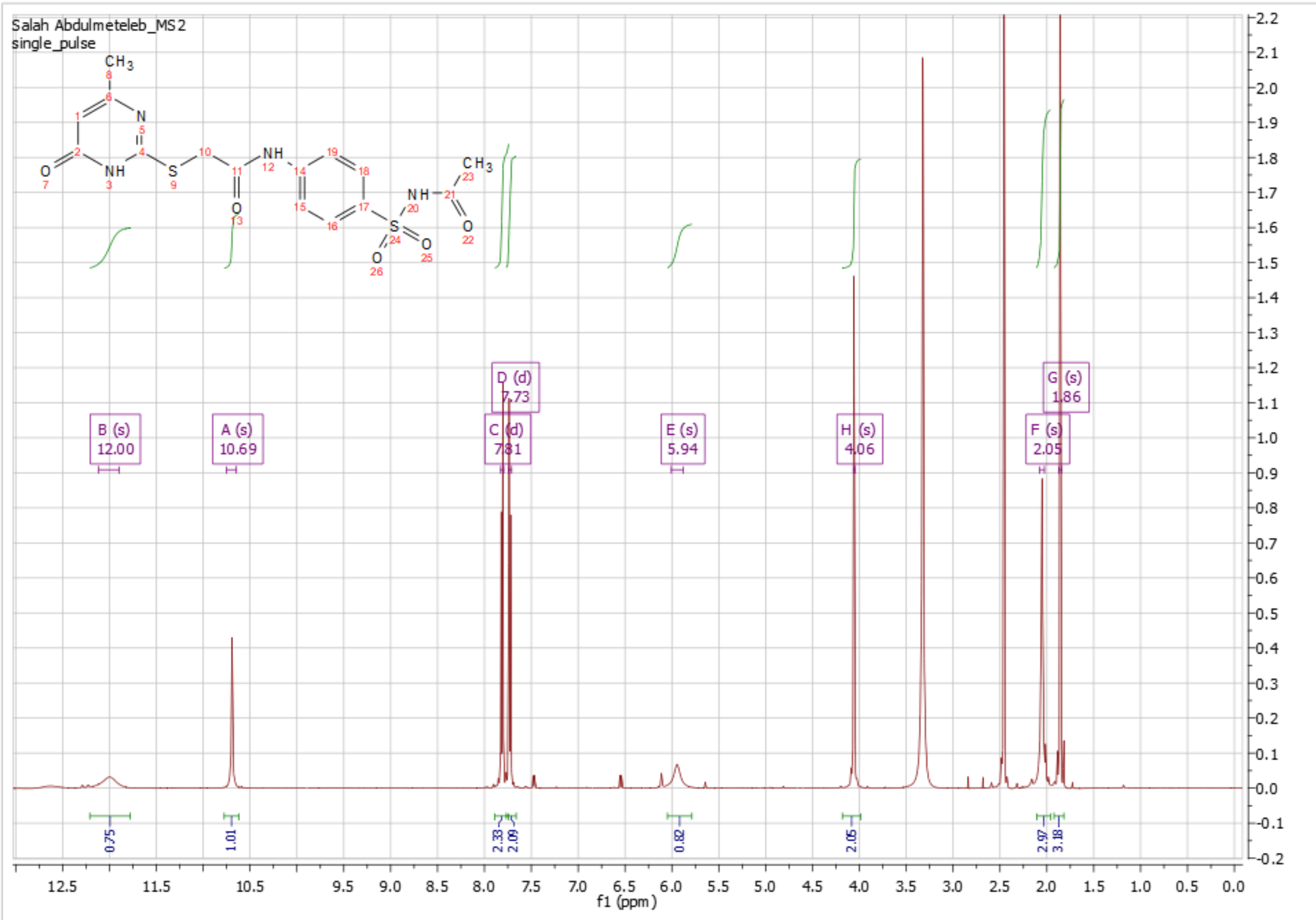

Fig. S2 : Expanded ^{1}H NMR of compound 4a

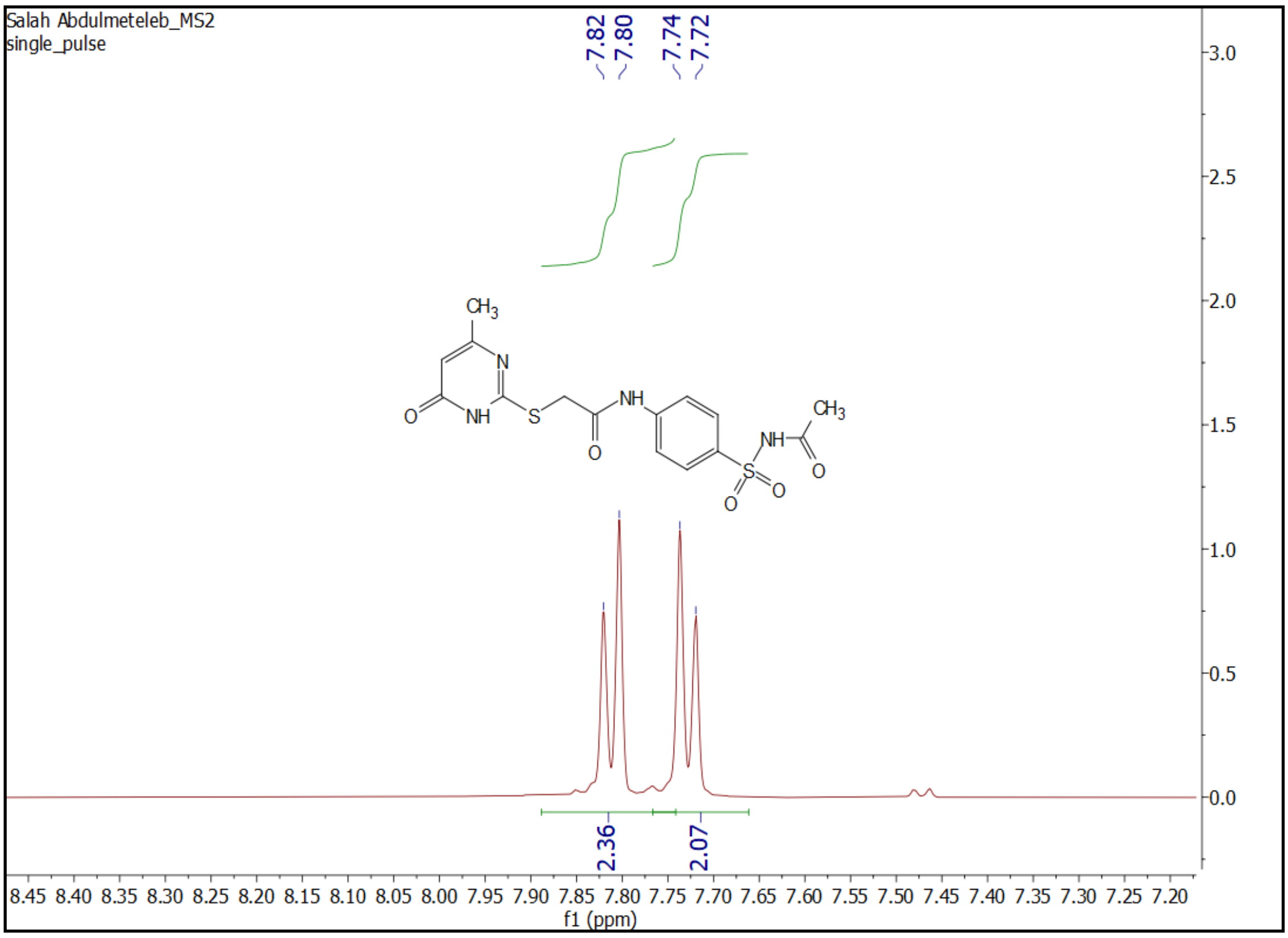

Fig. S3 : ^{13}C NMR of compound 4a

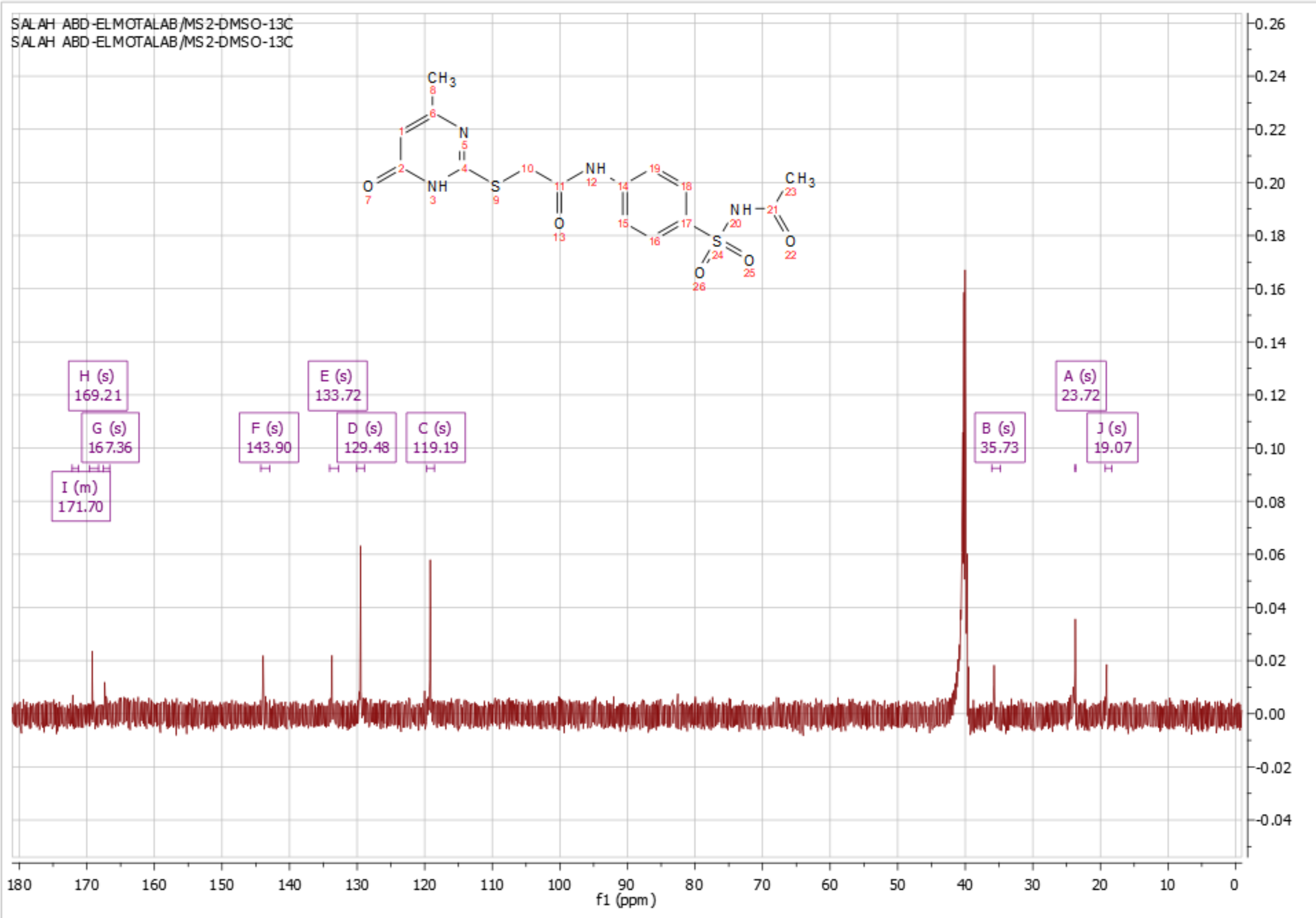

Figure S4: ^{1}H NMR of compound 4b

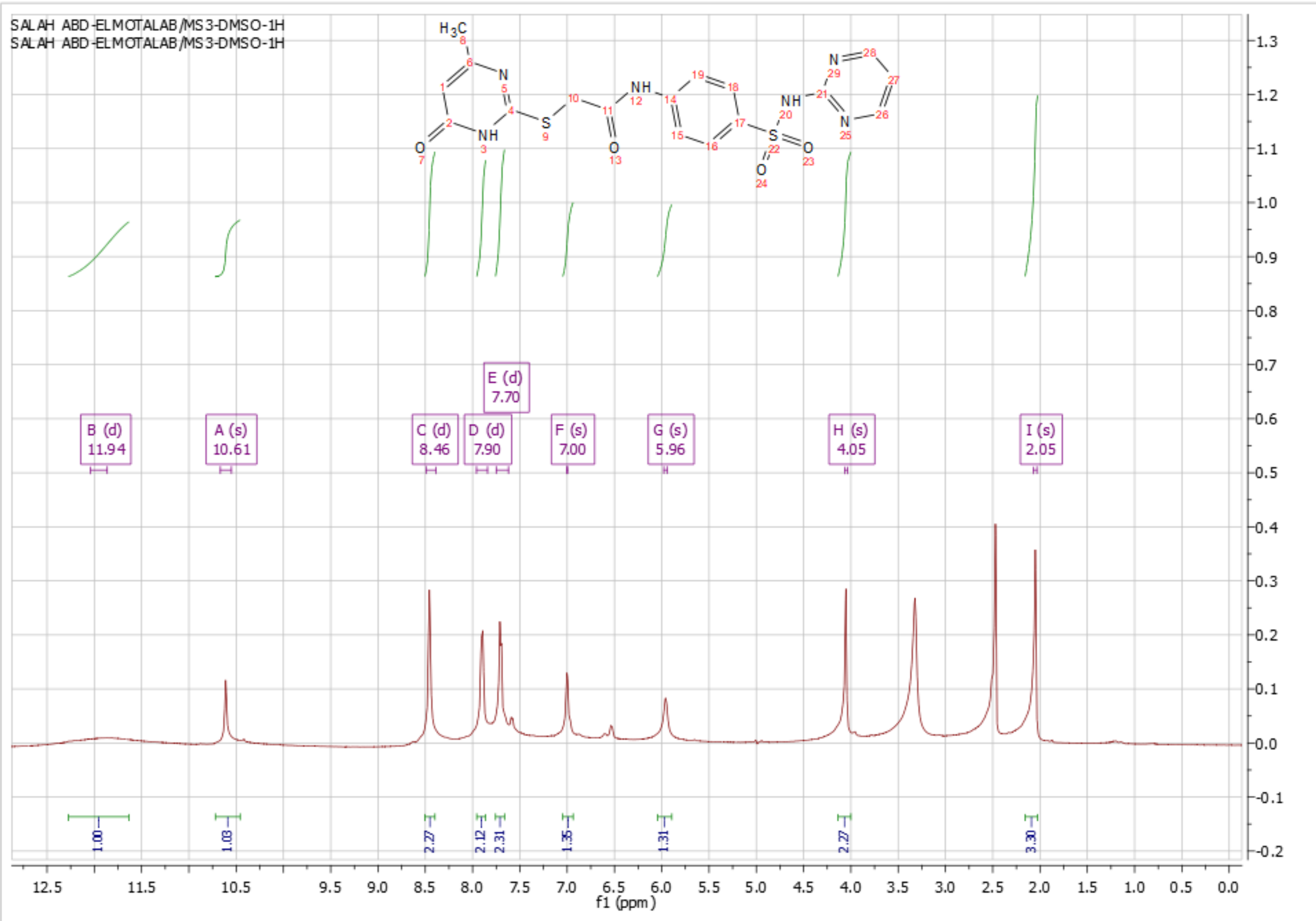

Figure S5: **Expanded ^{1}H NMR of compound 4b**

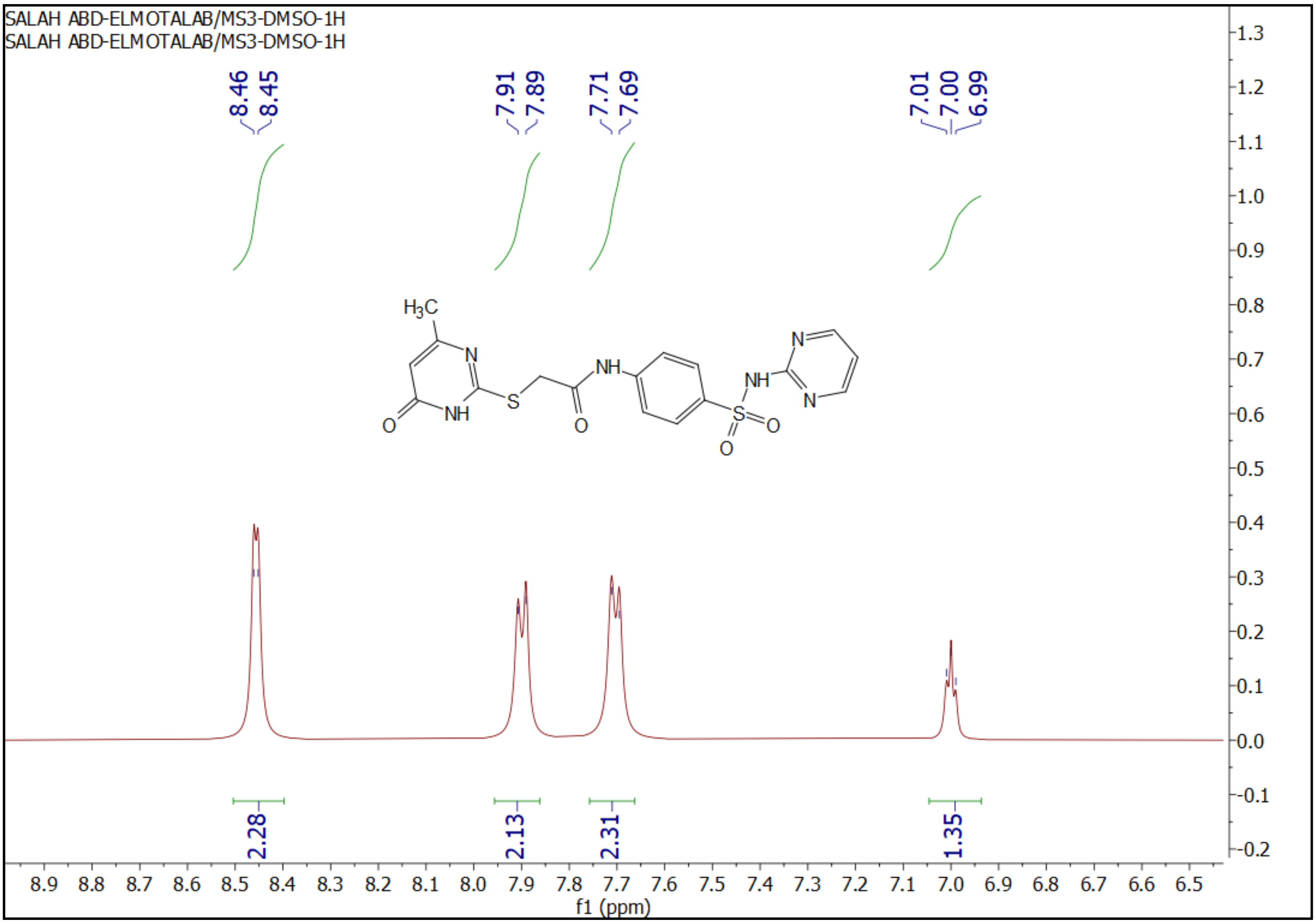

Figure S6: ^{13}C NMR of compound 4b

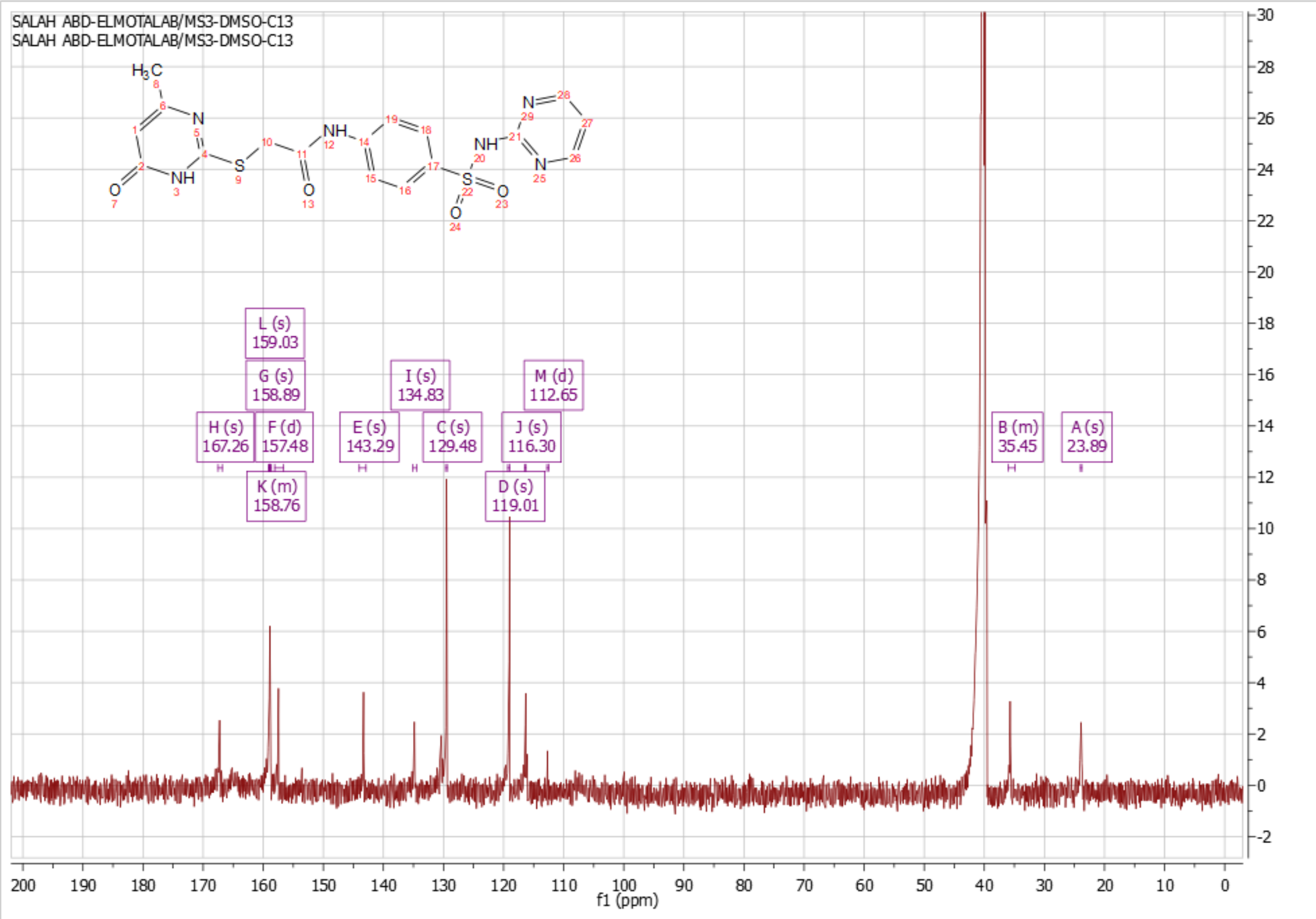

Figure S7: ^{1}H NMR of compound 4c

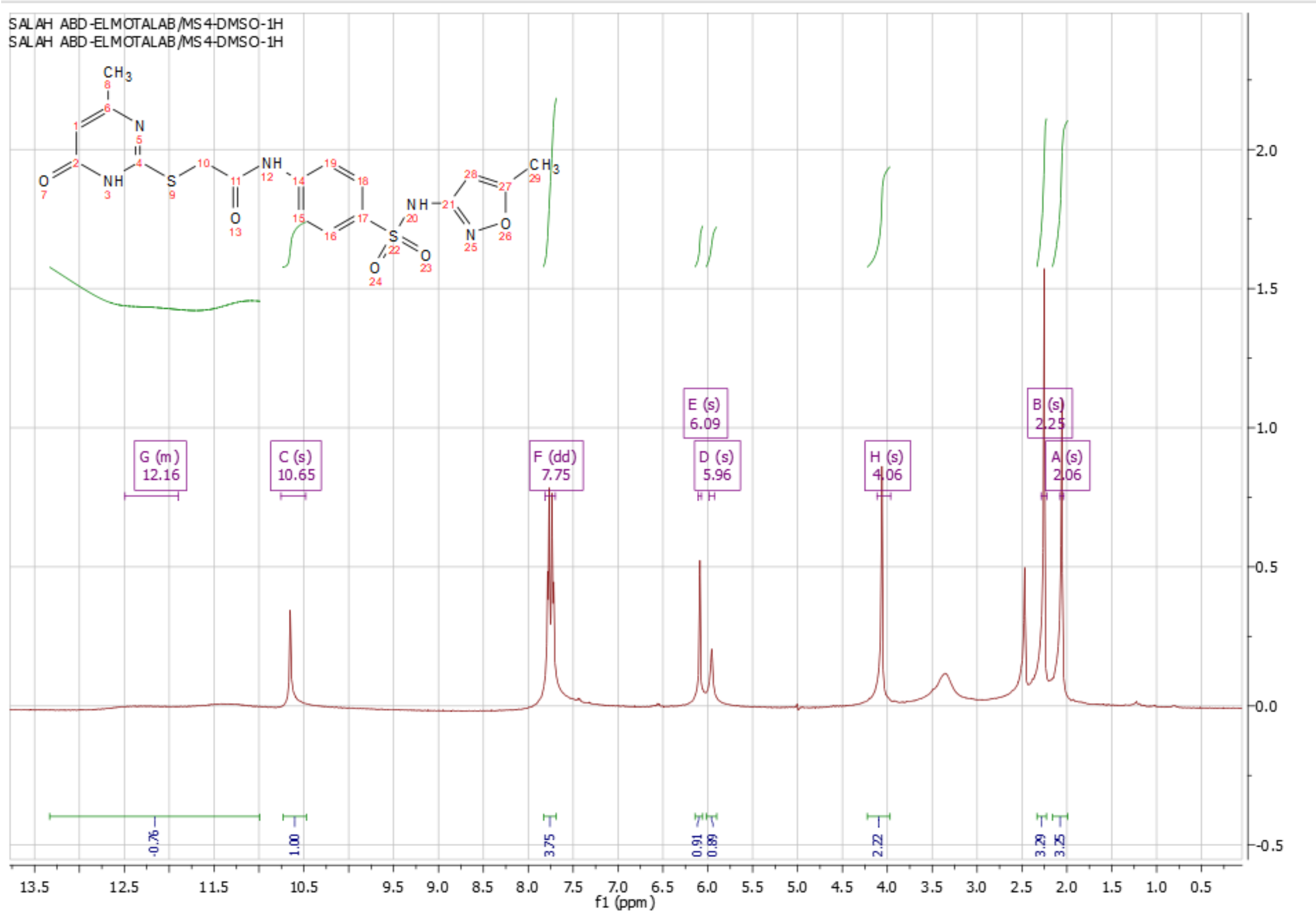

Figure S8: **Expanded ^{1}H NMR of compound 4c**

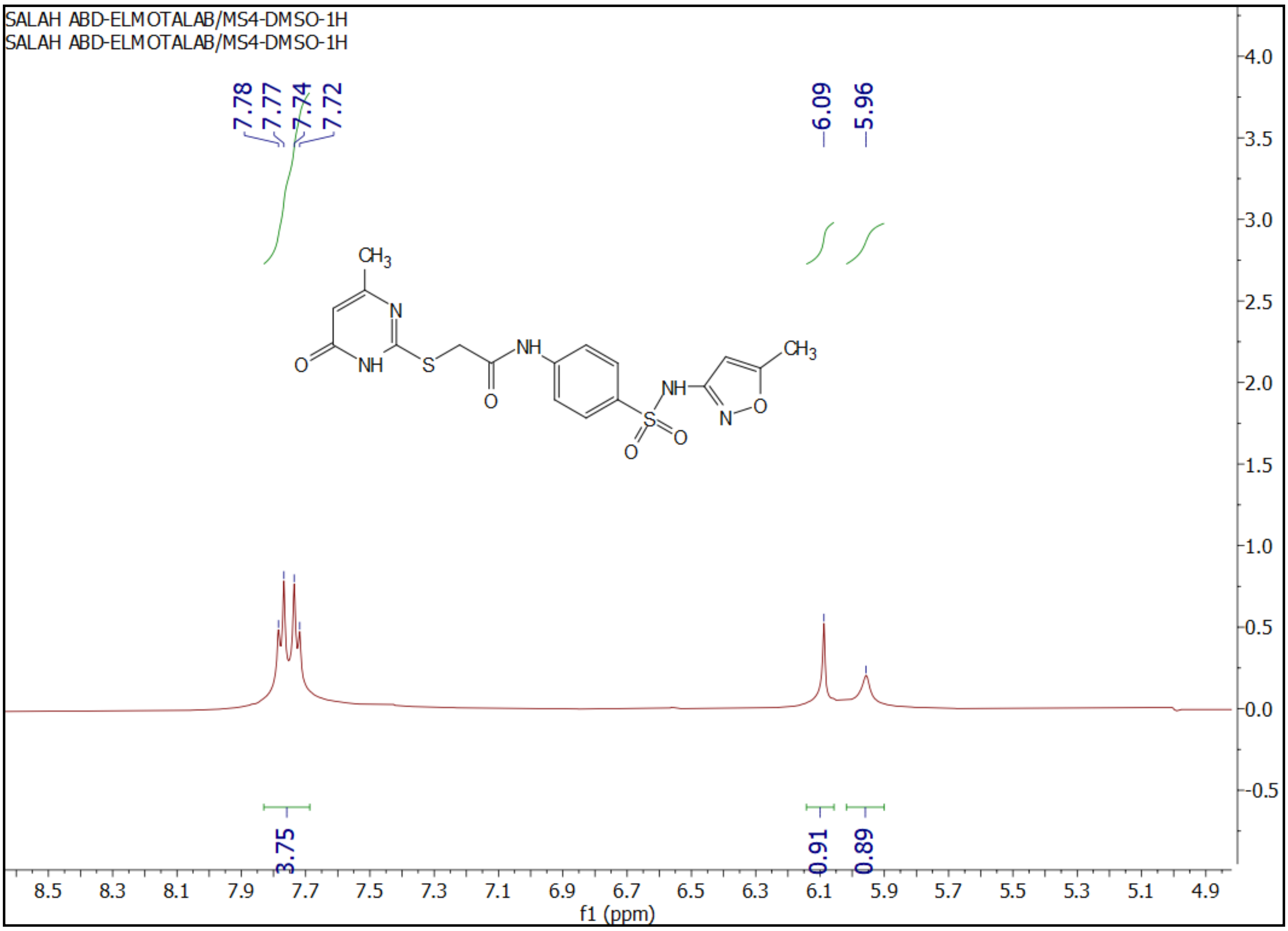

Figure S9: ^{13}C NMR of compound 4c

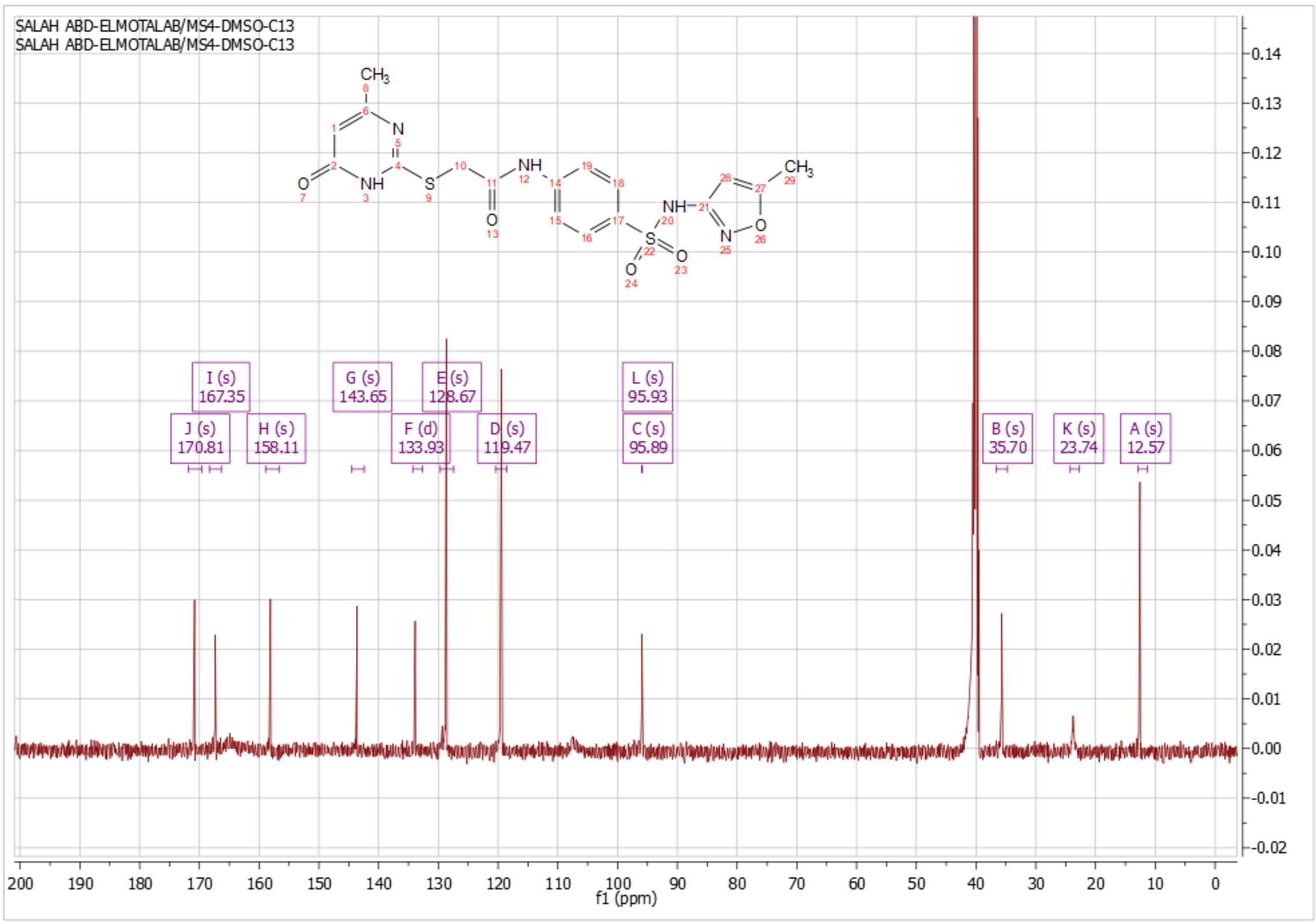

Figure S10: 1H NMR of compound 4d

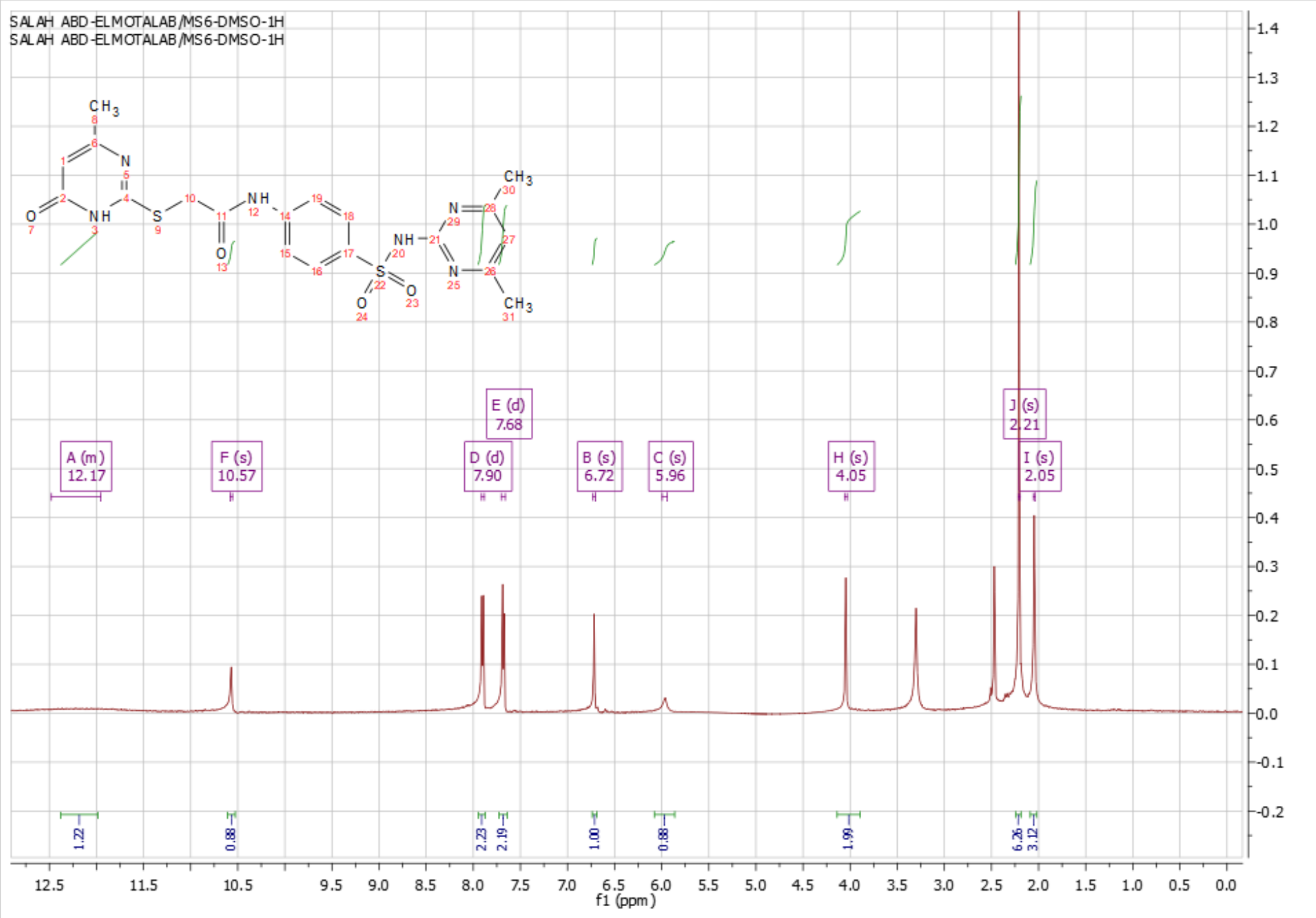

Figure S11: **Expanded ^{1}H NMR of compound 4d**

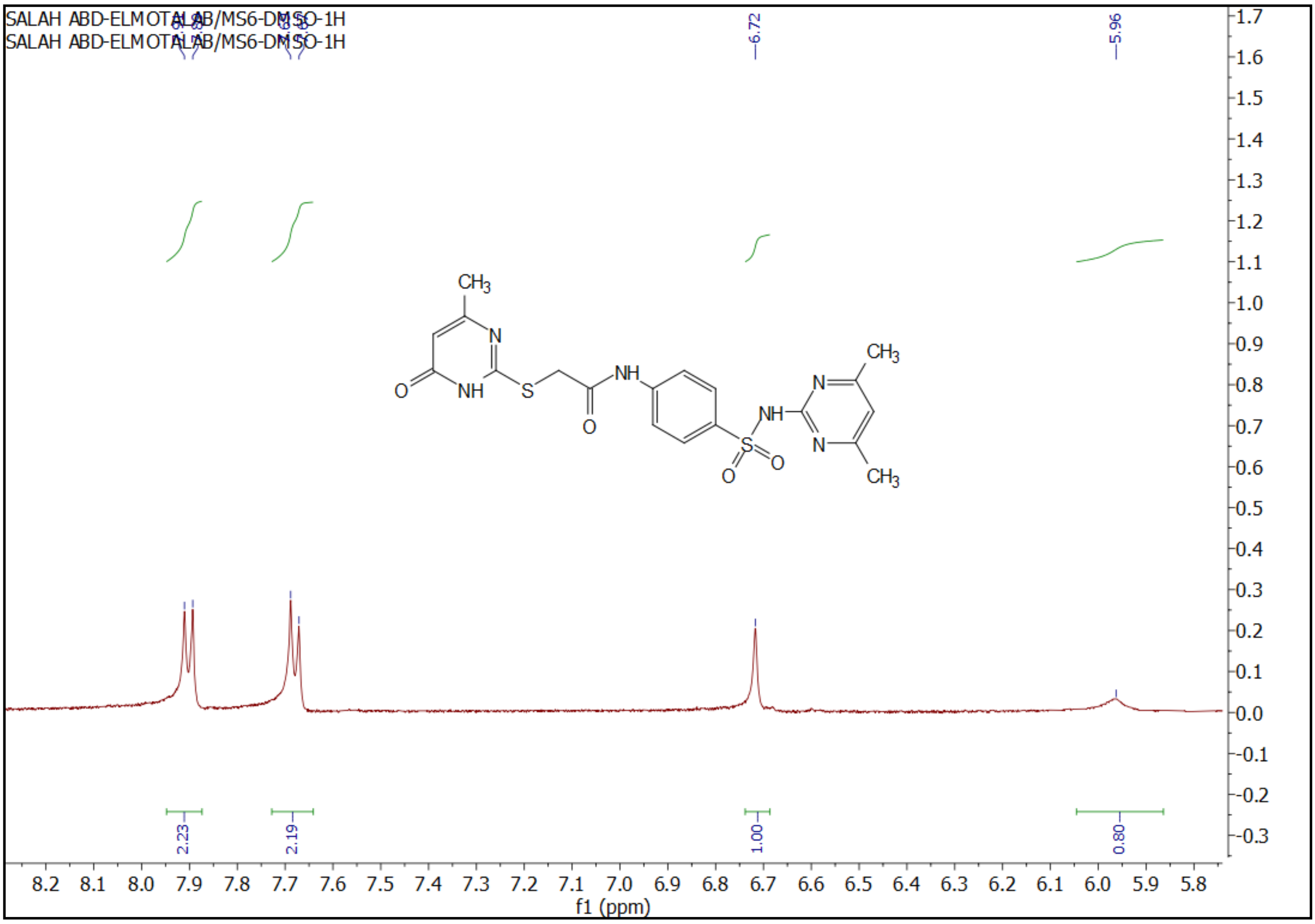

Figure S12: ^{13}C NMR of compound 4d

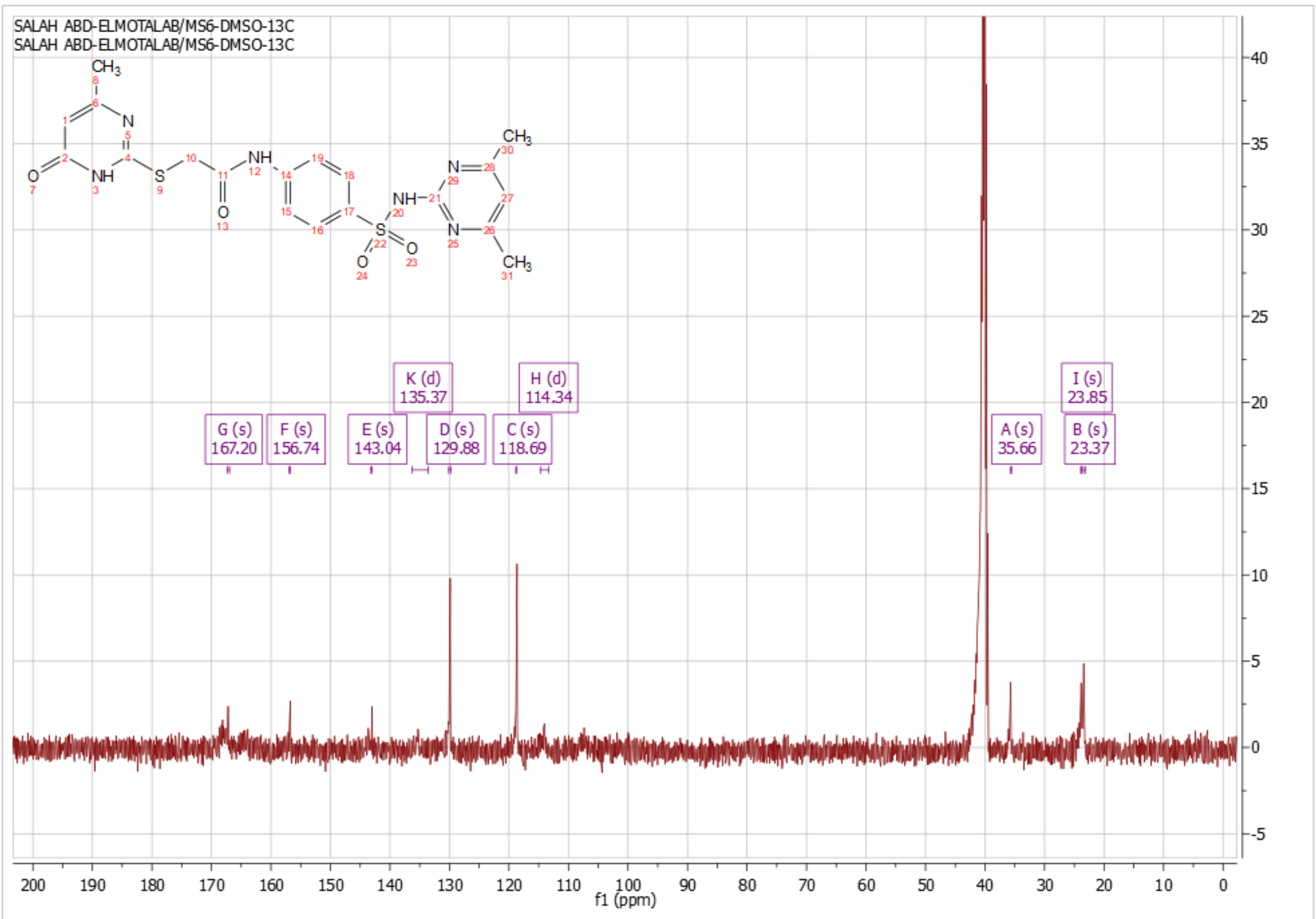

Figure S13: ^{1}H NMR of compound 4e

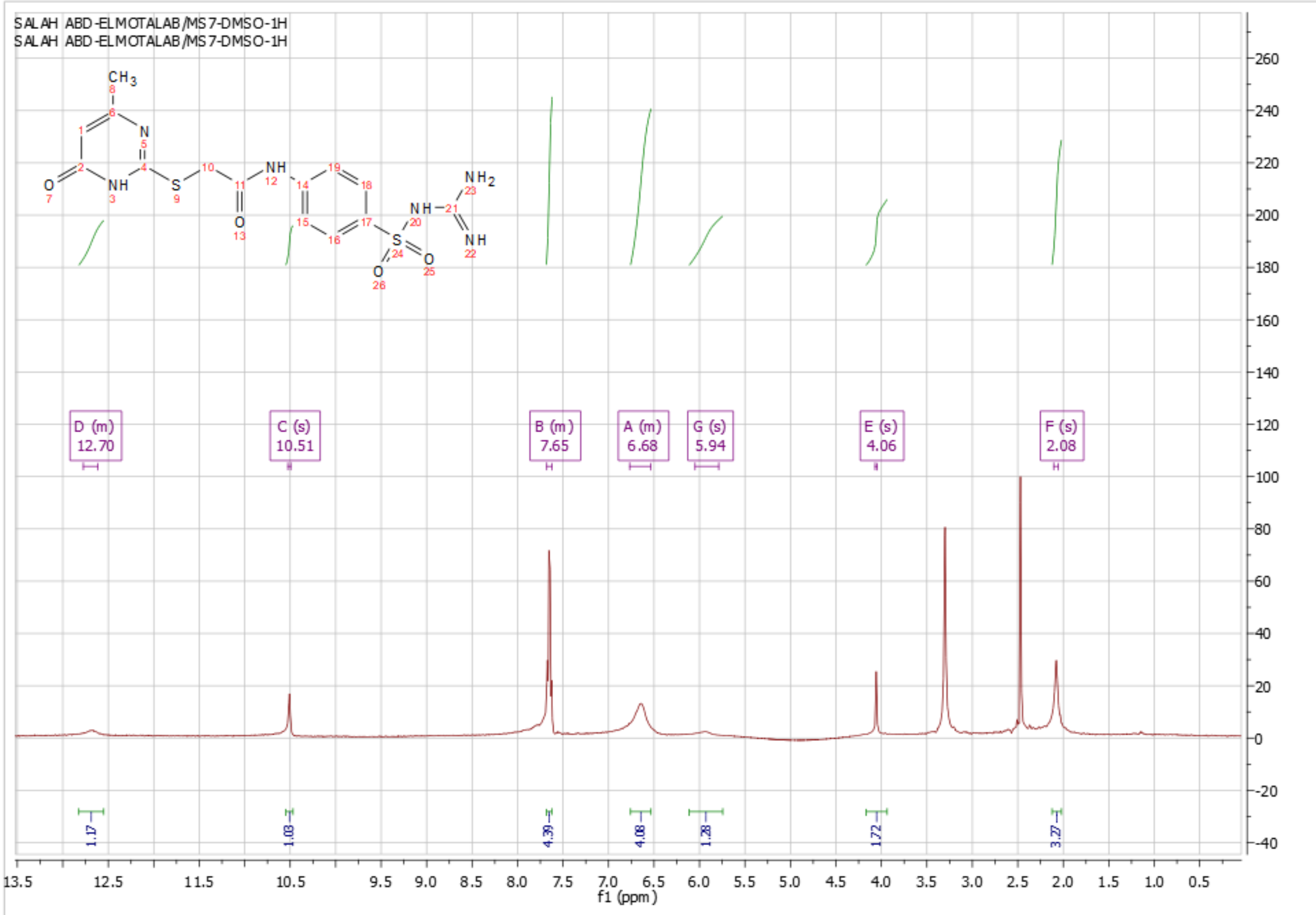

Figure S14: **Expanded ^{1}H NMR of compound 4e**

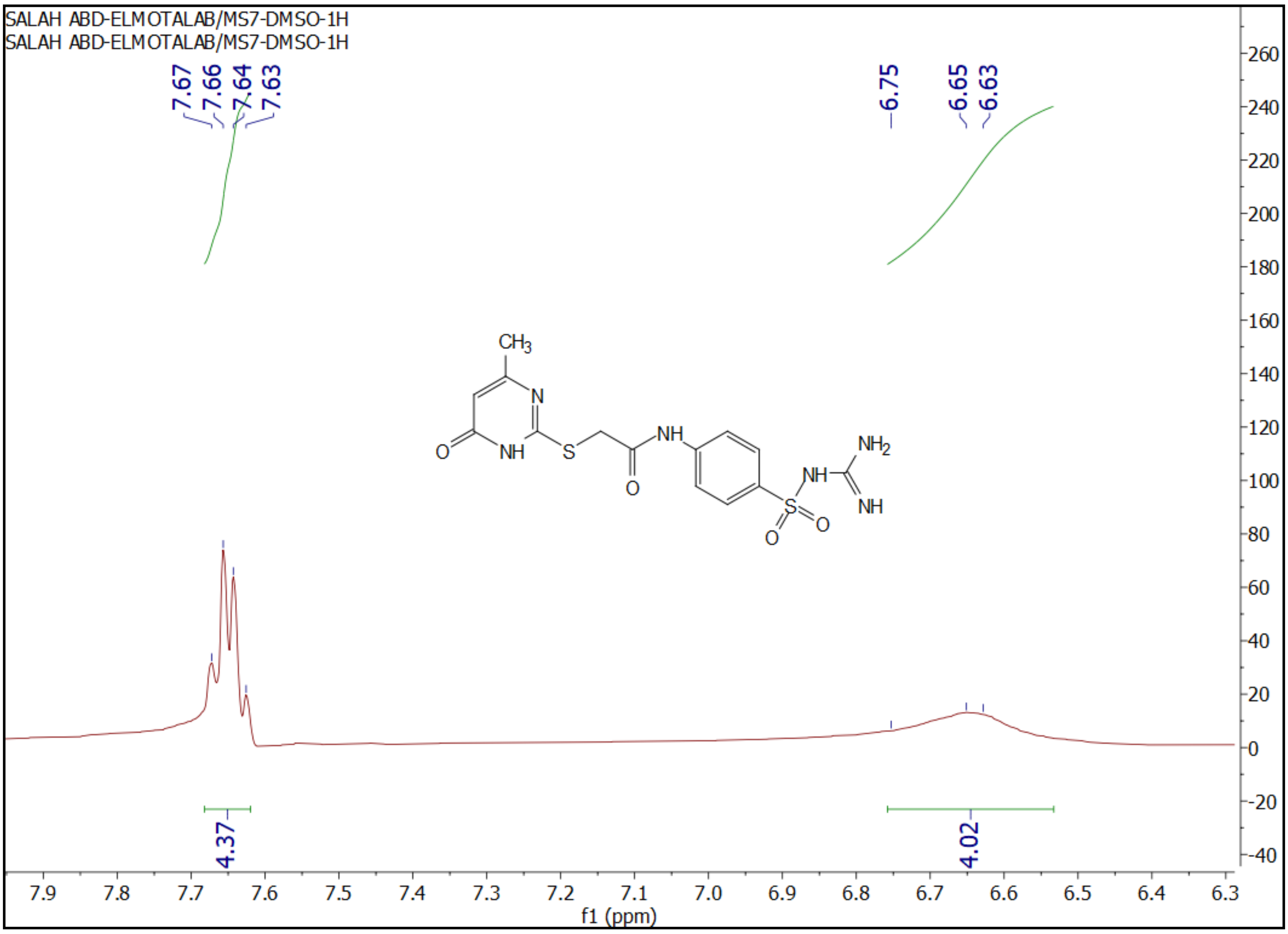

Figure S15: ^{13}C NMR of compound 4e

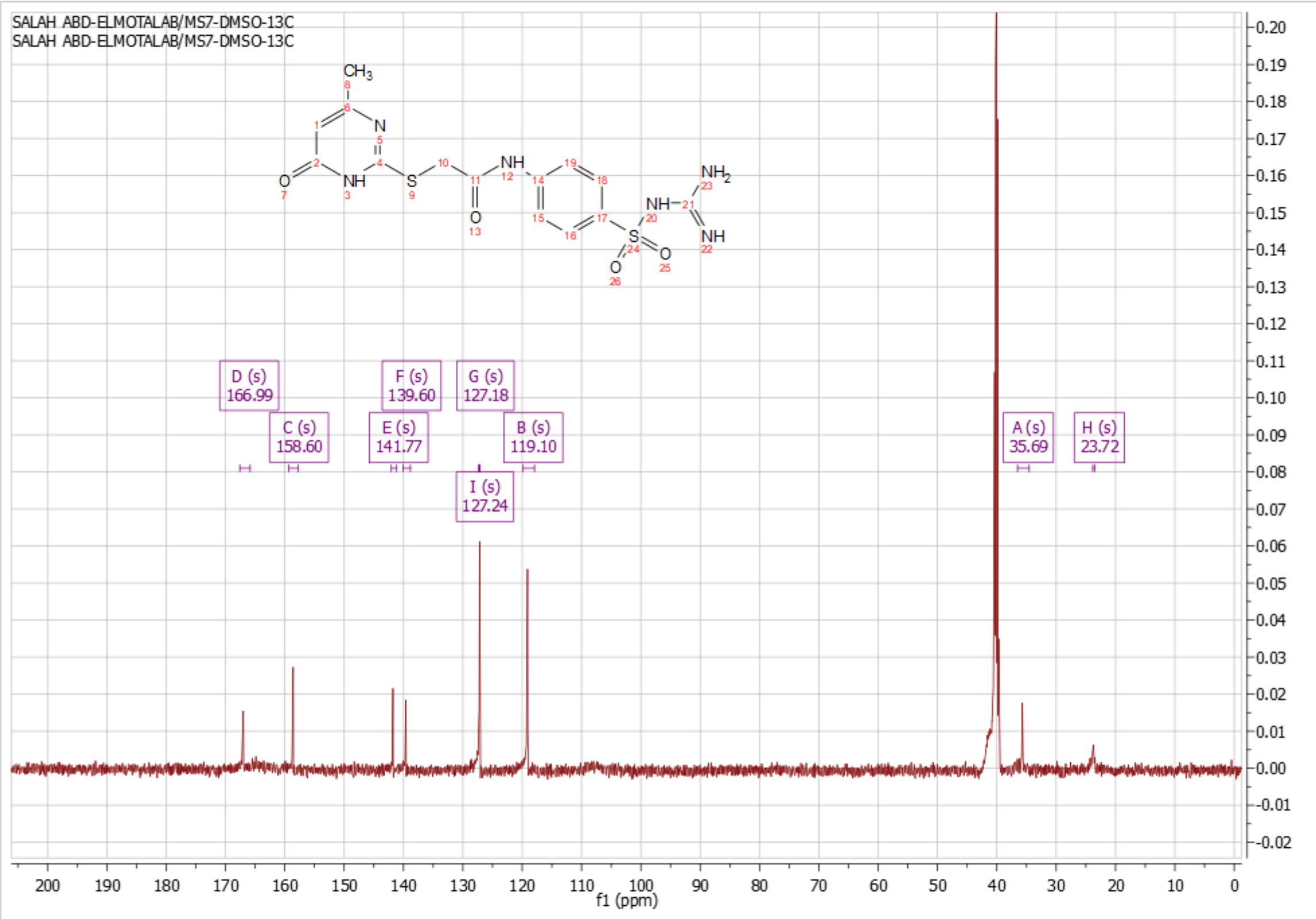

Figure S16: ^{1}H NMR of compound 4f

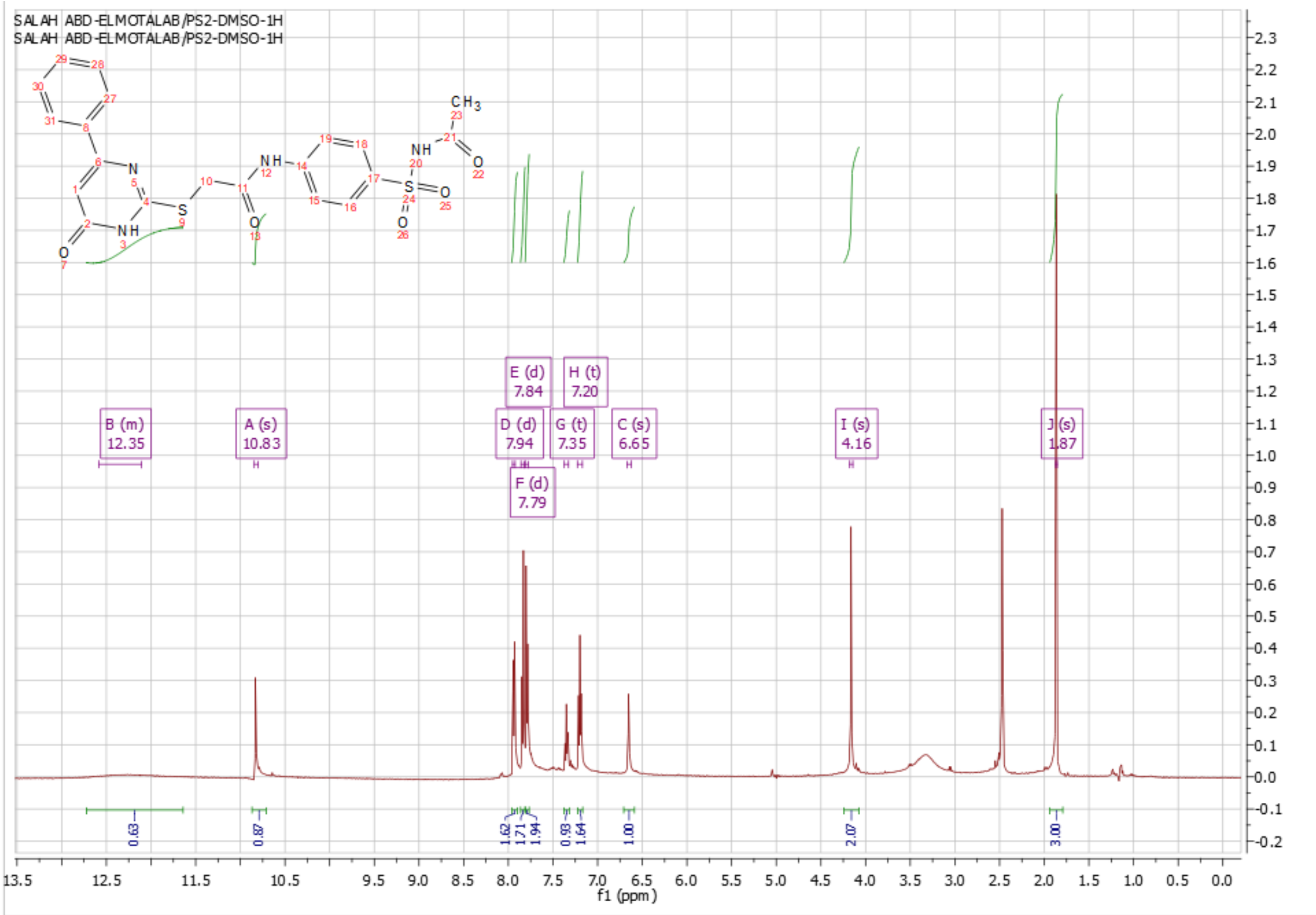

Figure S17: **Expanded ^{1}H NMR of compound 4f**

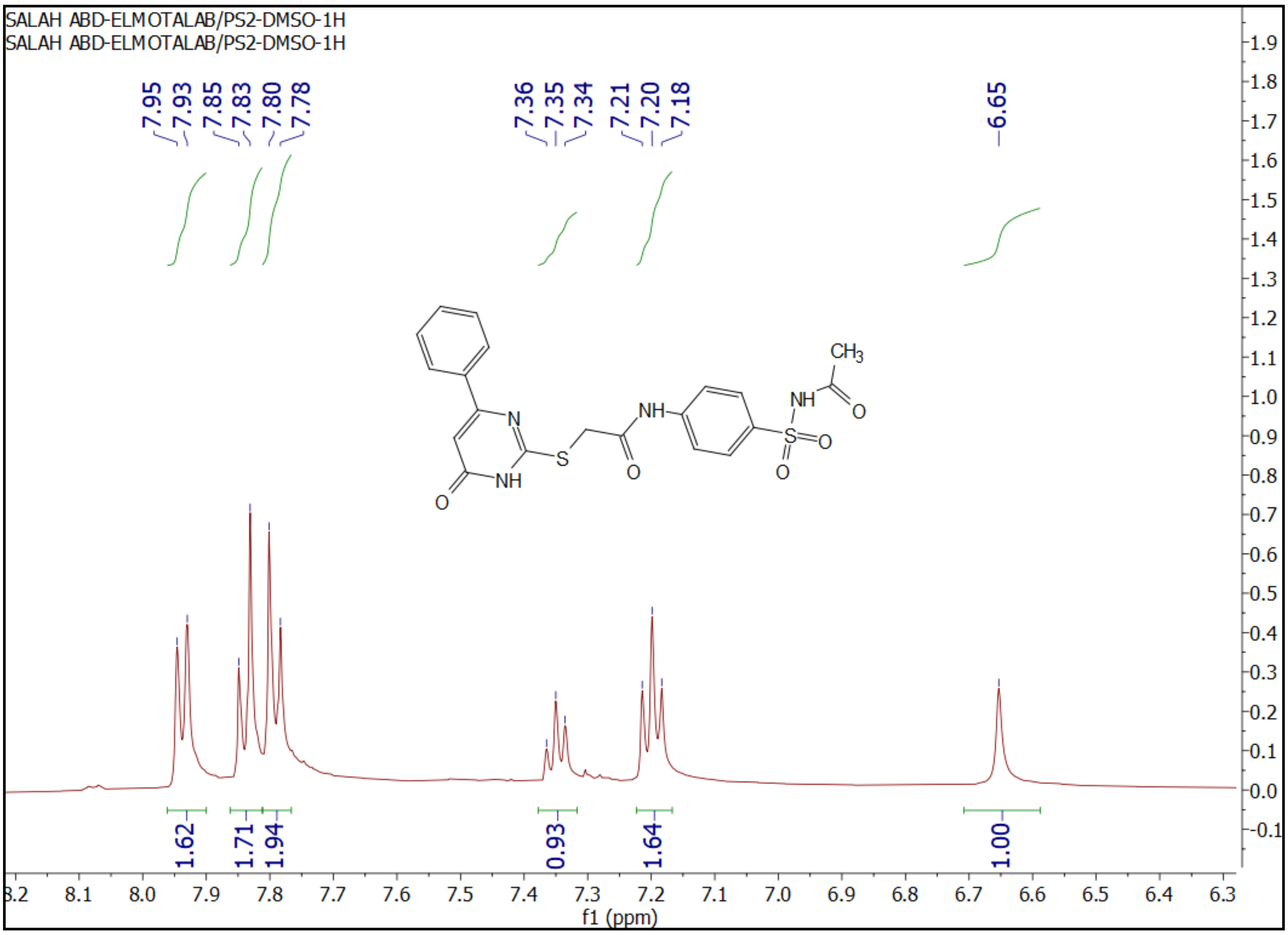

Figure S18: ^{13}C NMR of compound 4f

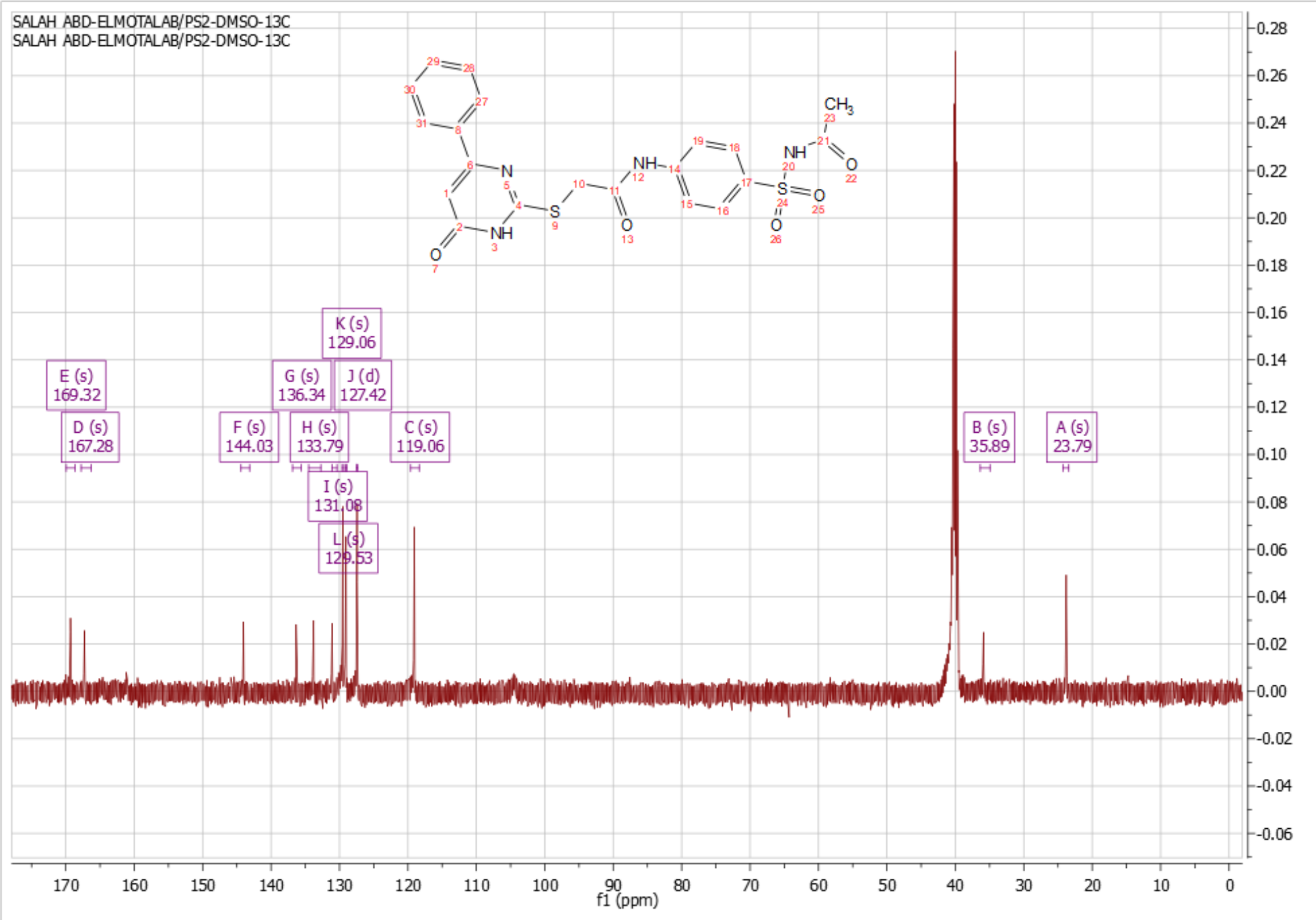

Figure S19: ¹H NMR of compound 4g

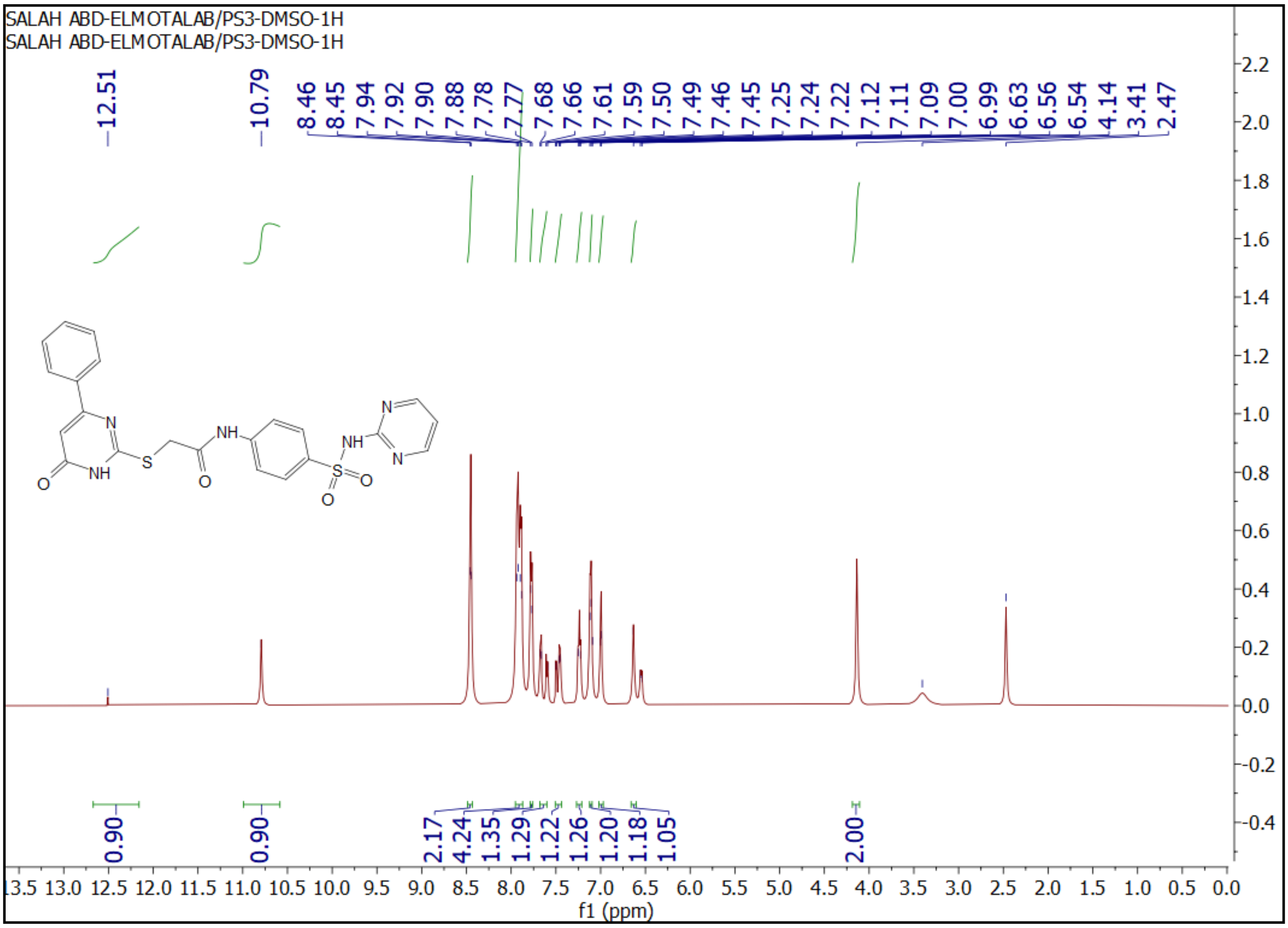

Figure S20: **Expanded ^{1}H NMR of compound 4g**

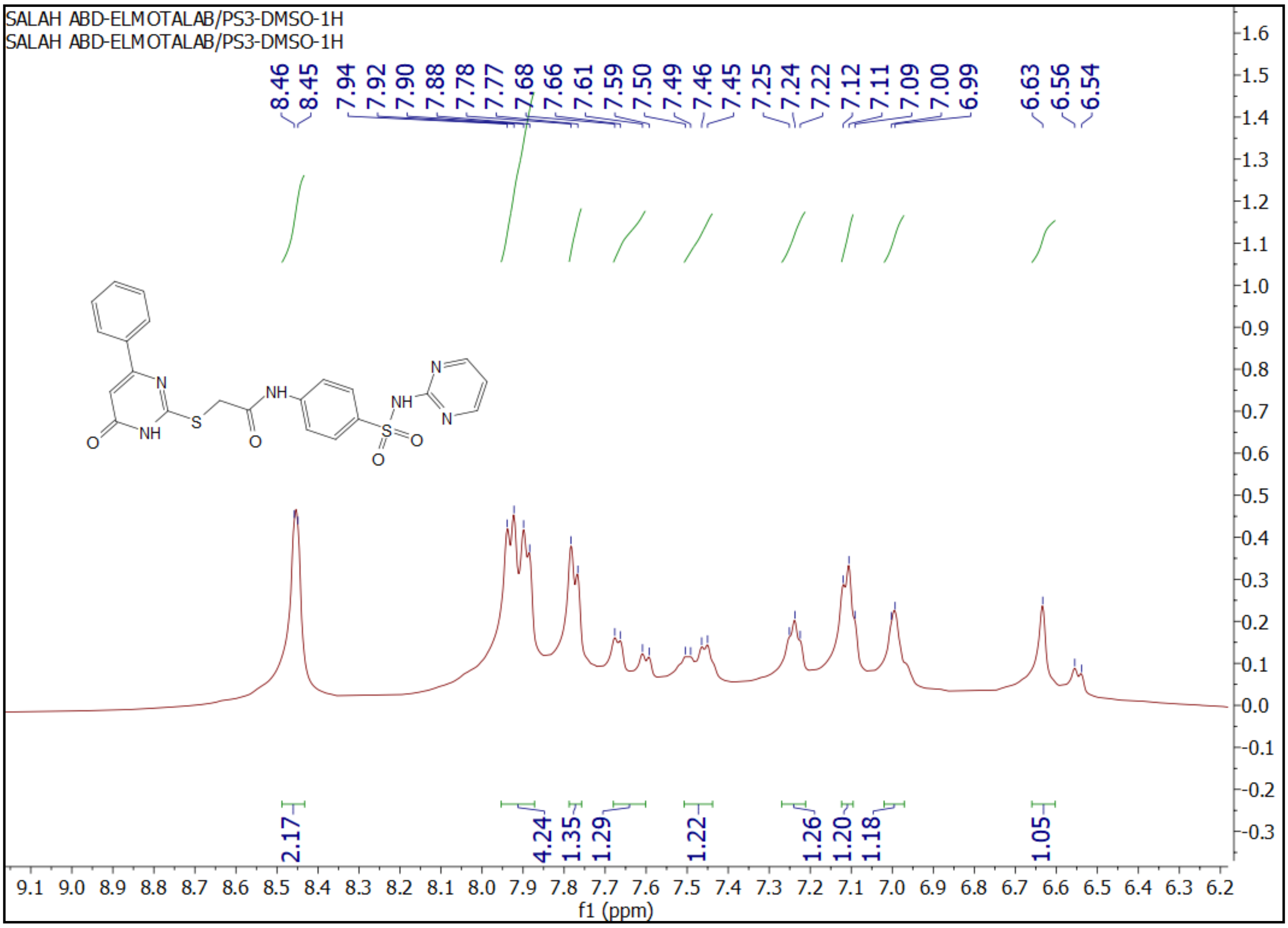

Figure S21: ^{13}C NMR of compound 4g

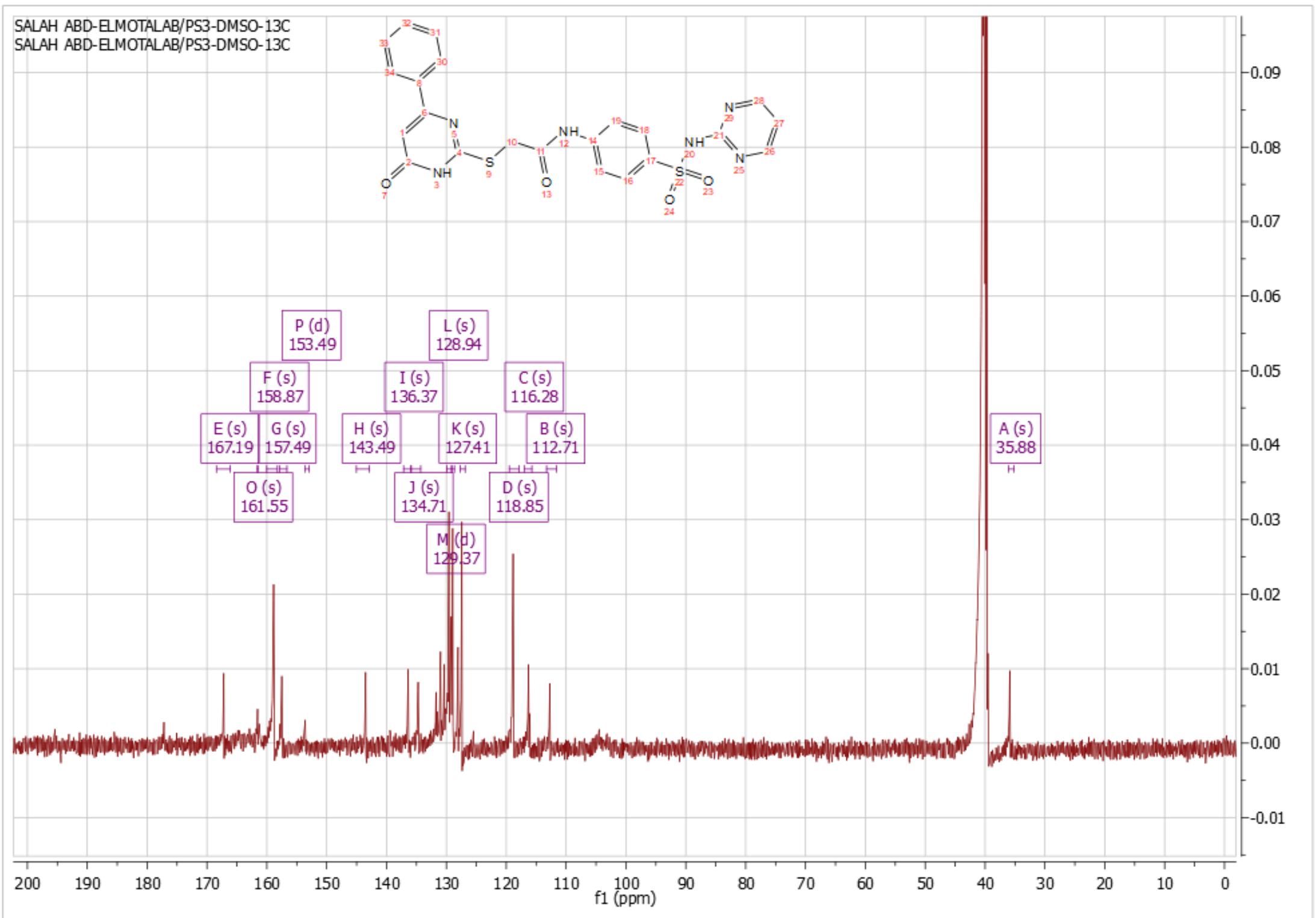

Figure S22: ^{1}H NMR of compound 4h

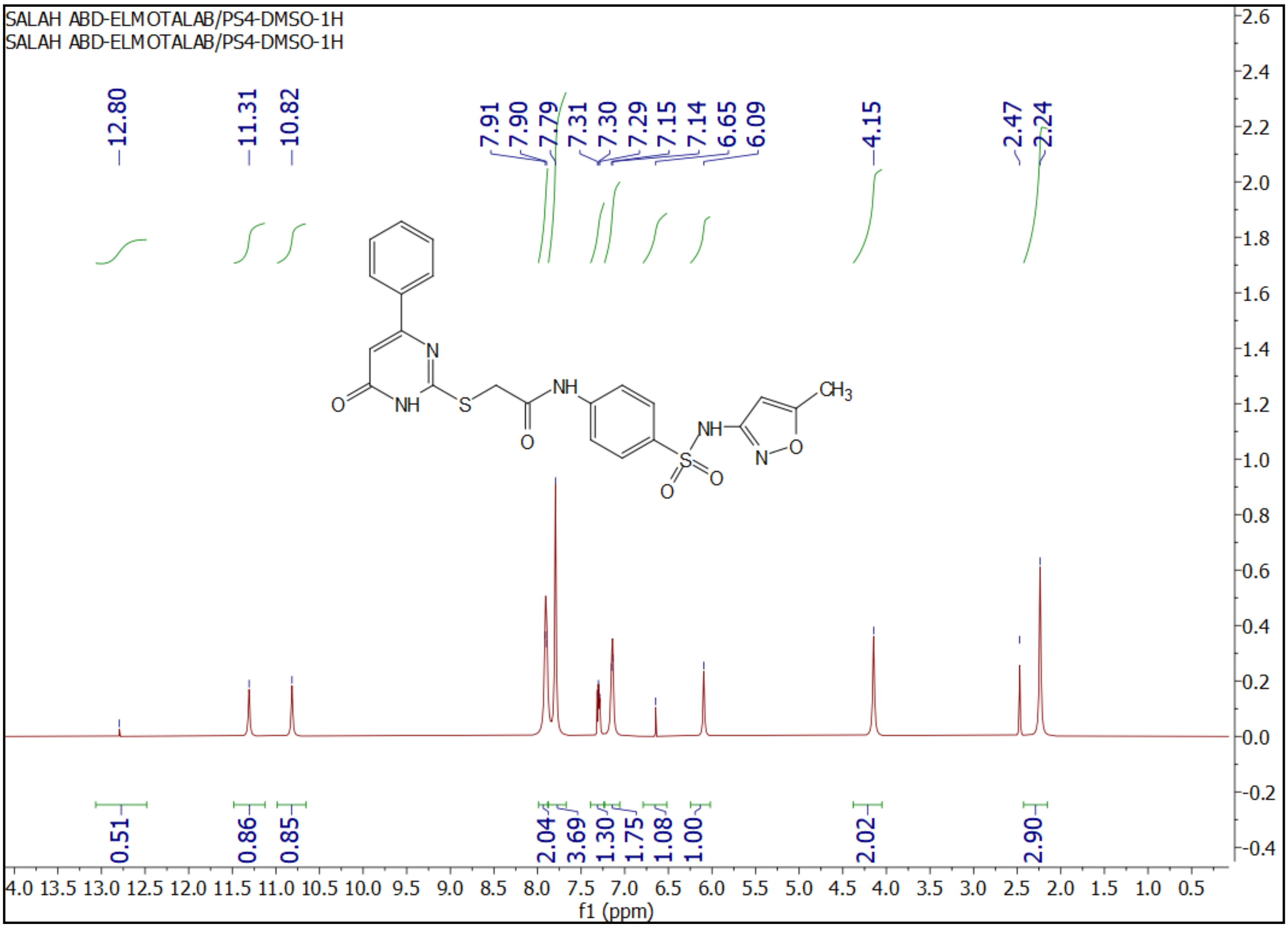

Figure S23: **Expanded ^{1}H NMR of compound 4h**

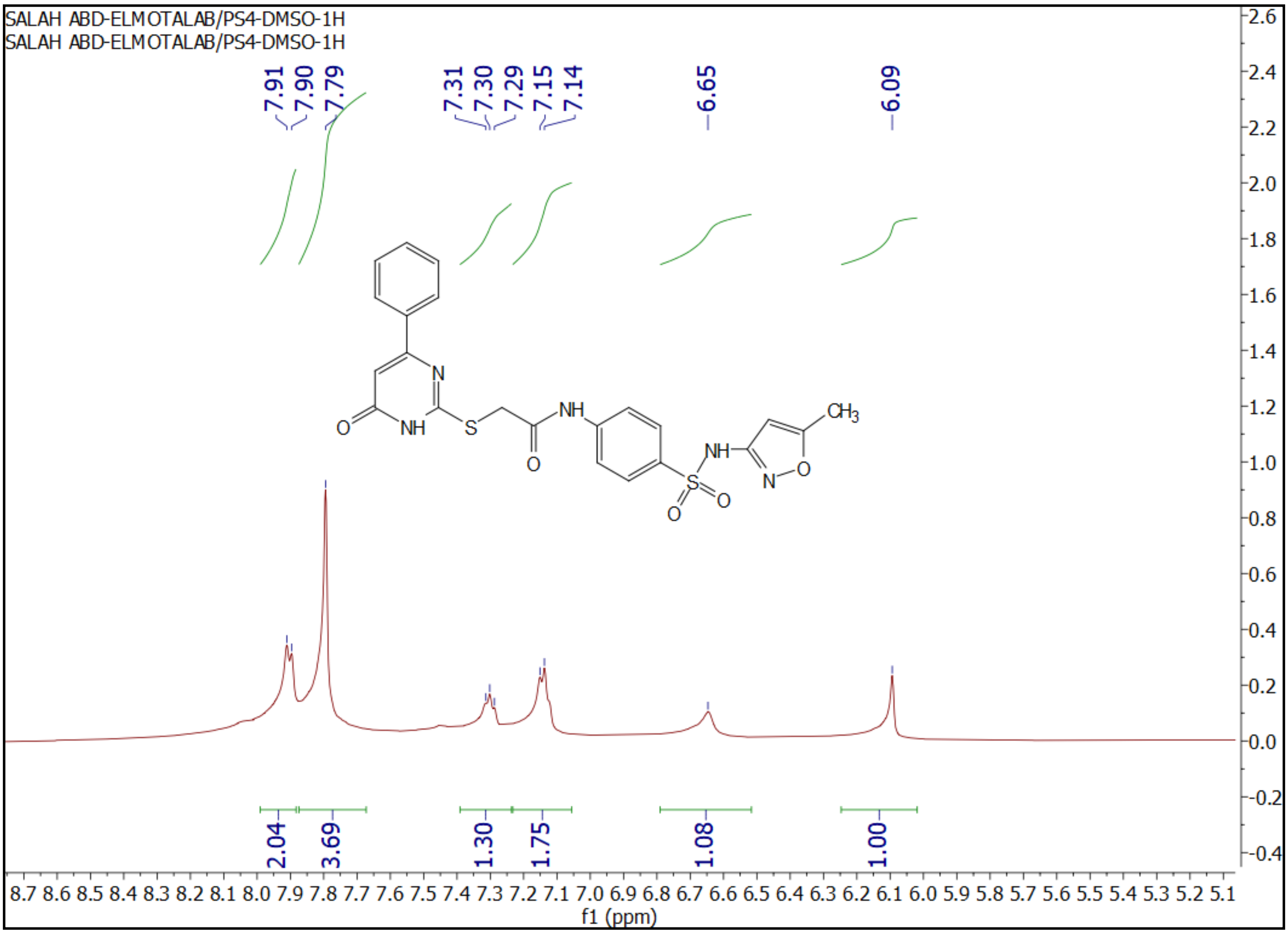

Figure S24: ^{13}C NMR of compound 4h

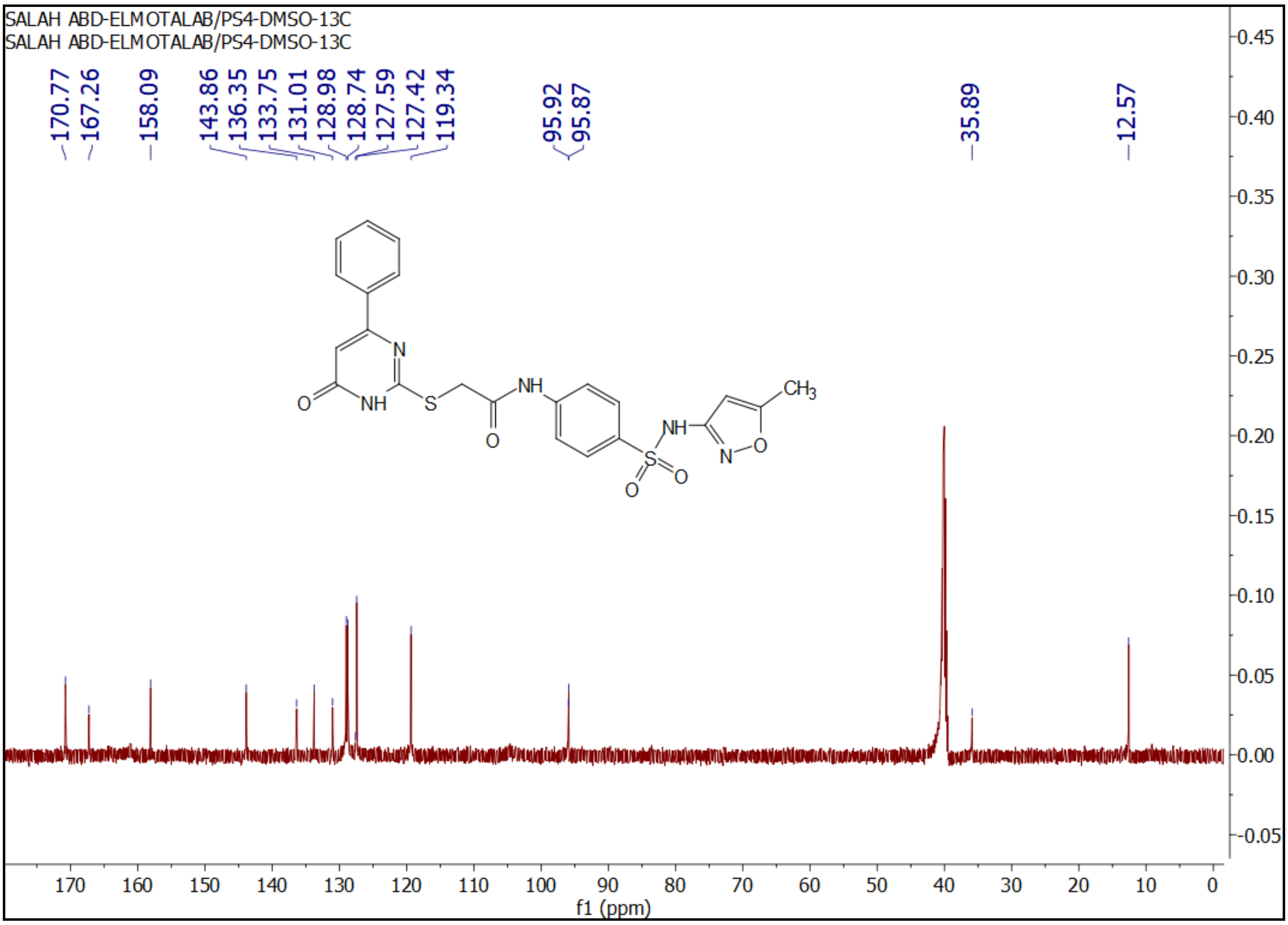

Figure S25: ^{1}H NMR of compound 4i

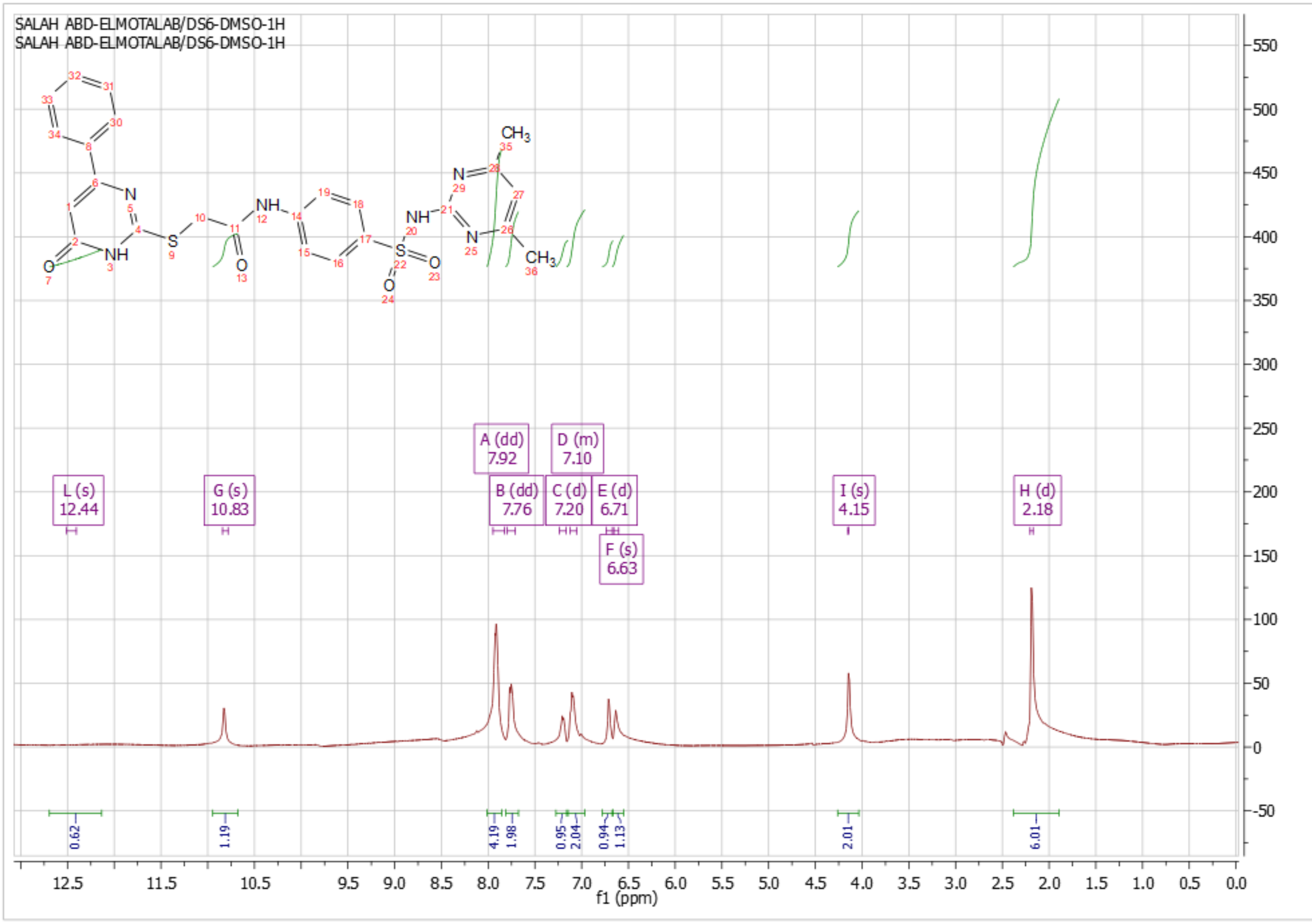

Figure S26: **Expanded ^{1}H NMR of compound 4i**

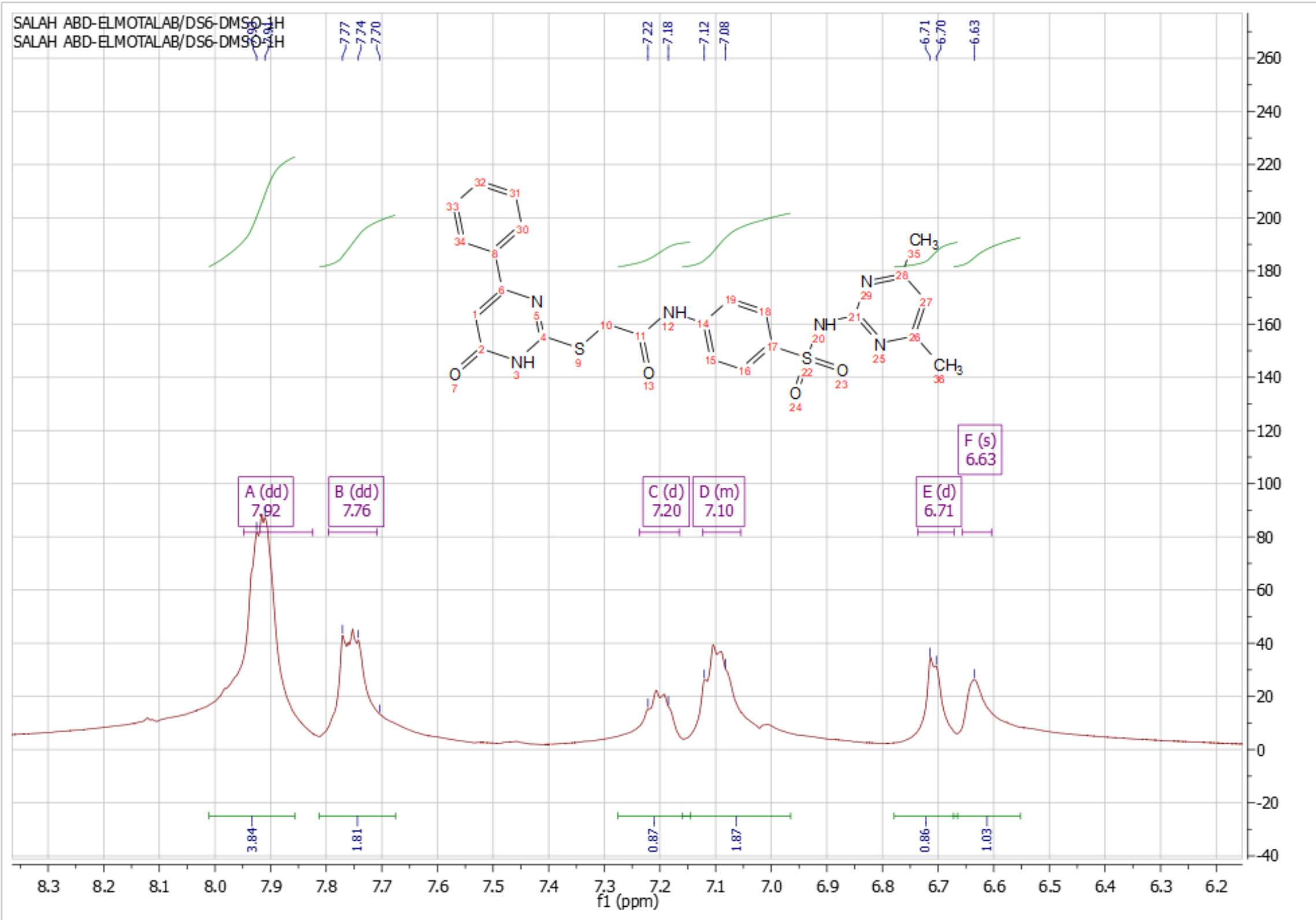

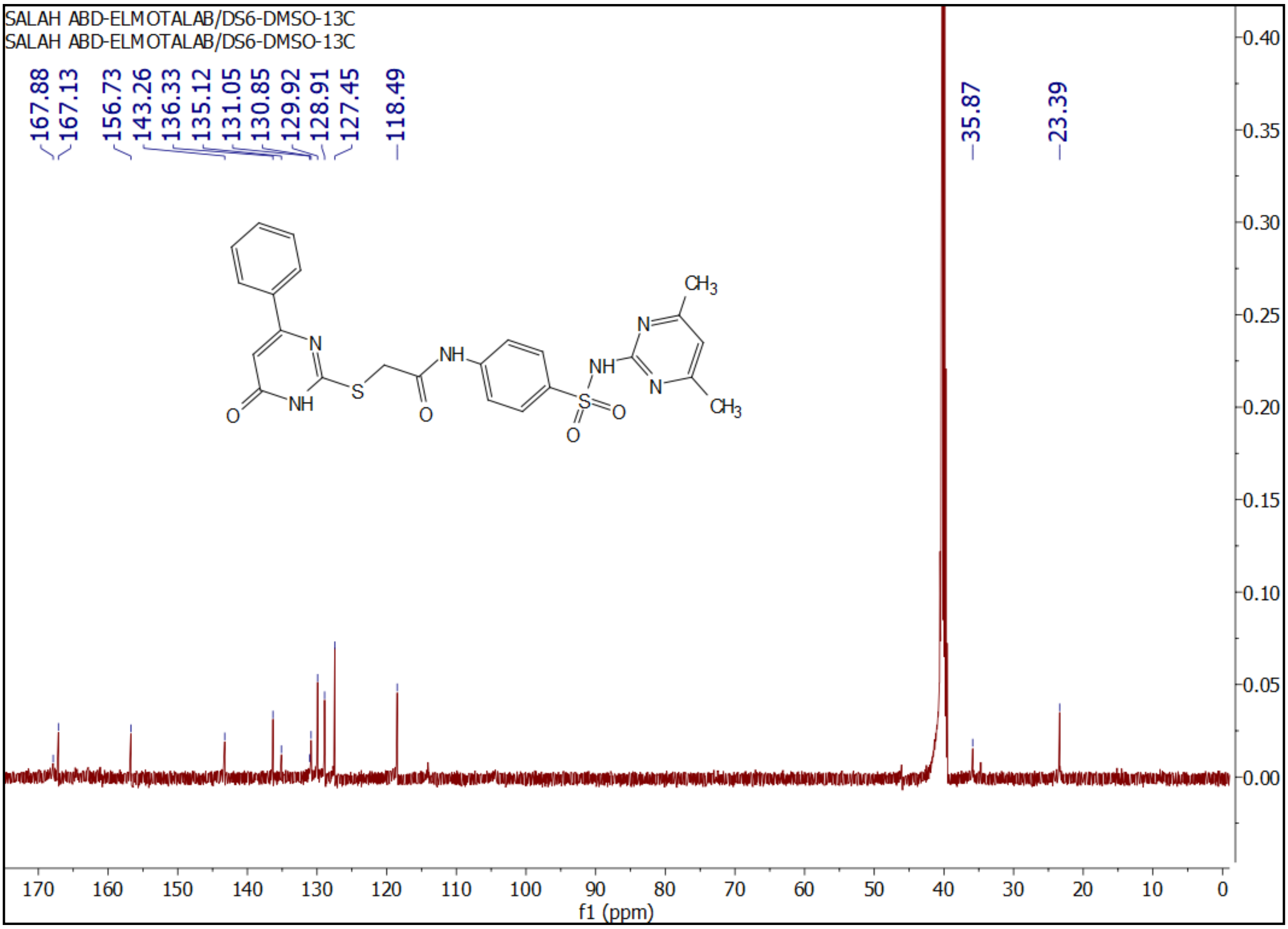

Figure S27: ^{13}C NMR of compound 4i

Figure S28: ^{1}H NMR of compound 4j

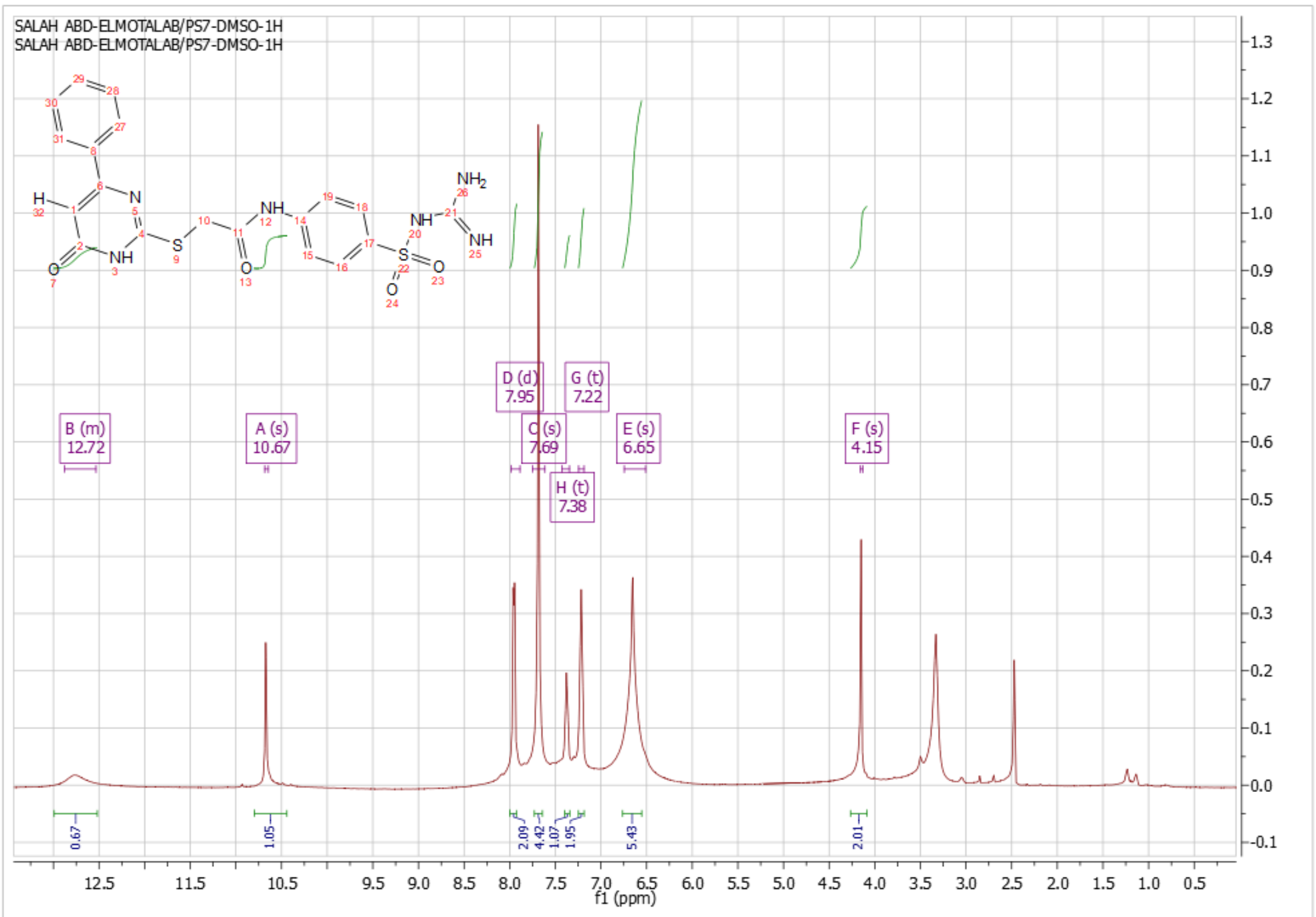

Figure S29: **Expanded ^{1}H NMR of compound 4j**

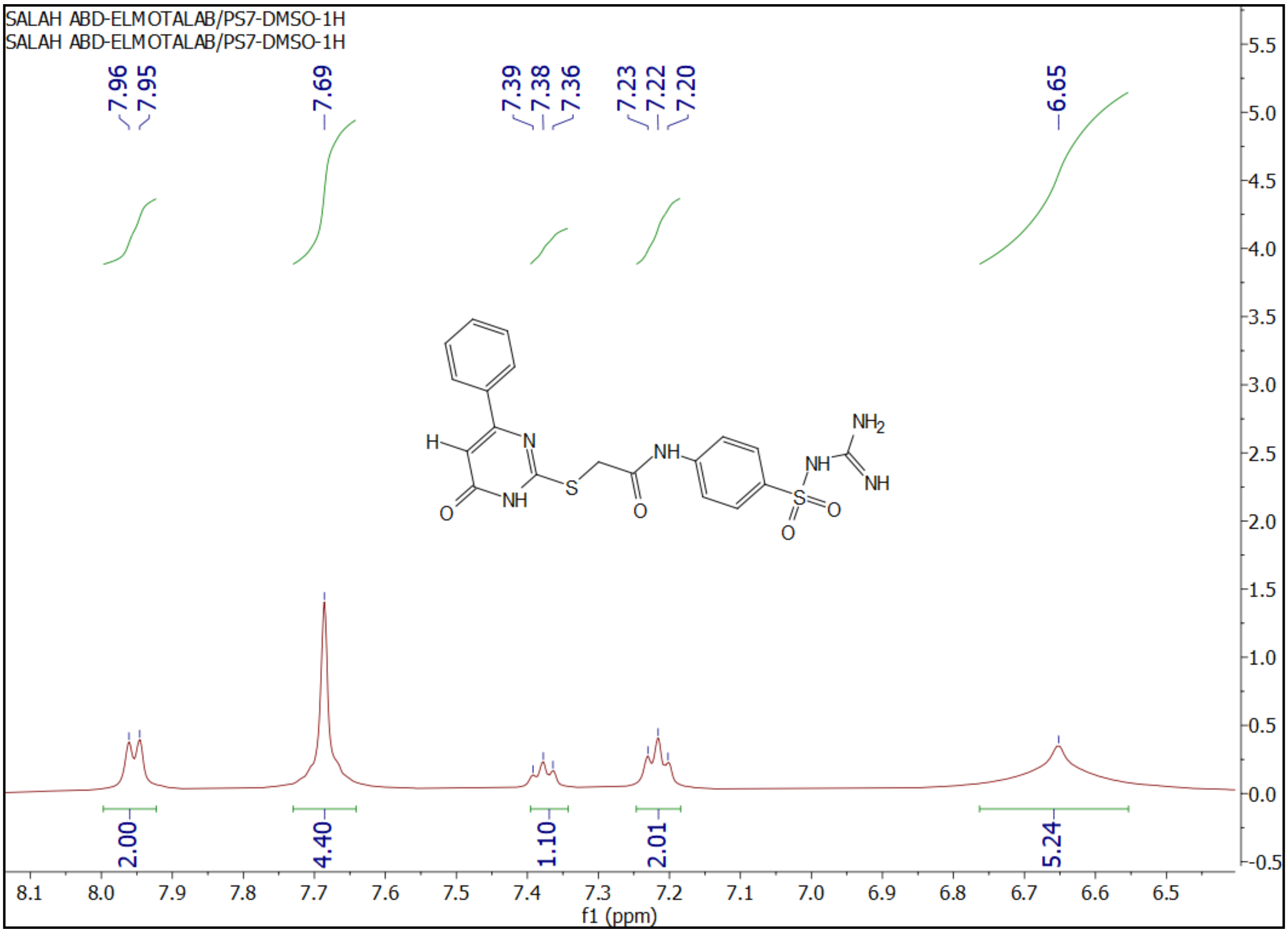

Figure S30: ^{13}C NMR of compound 4j

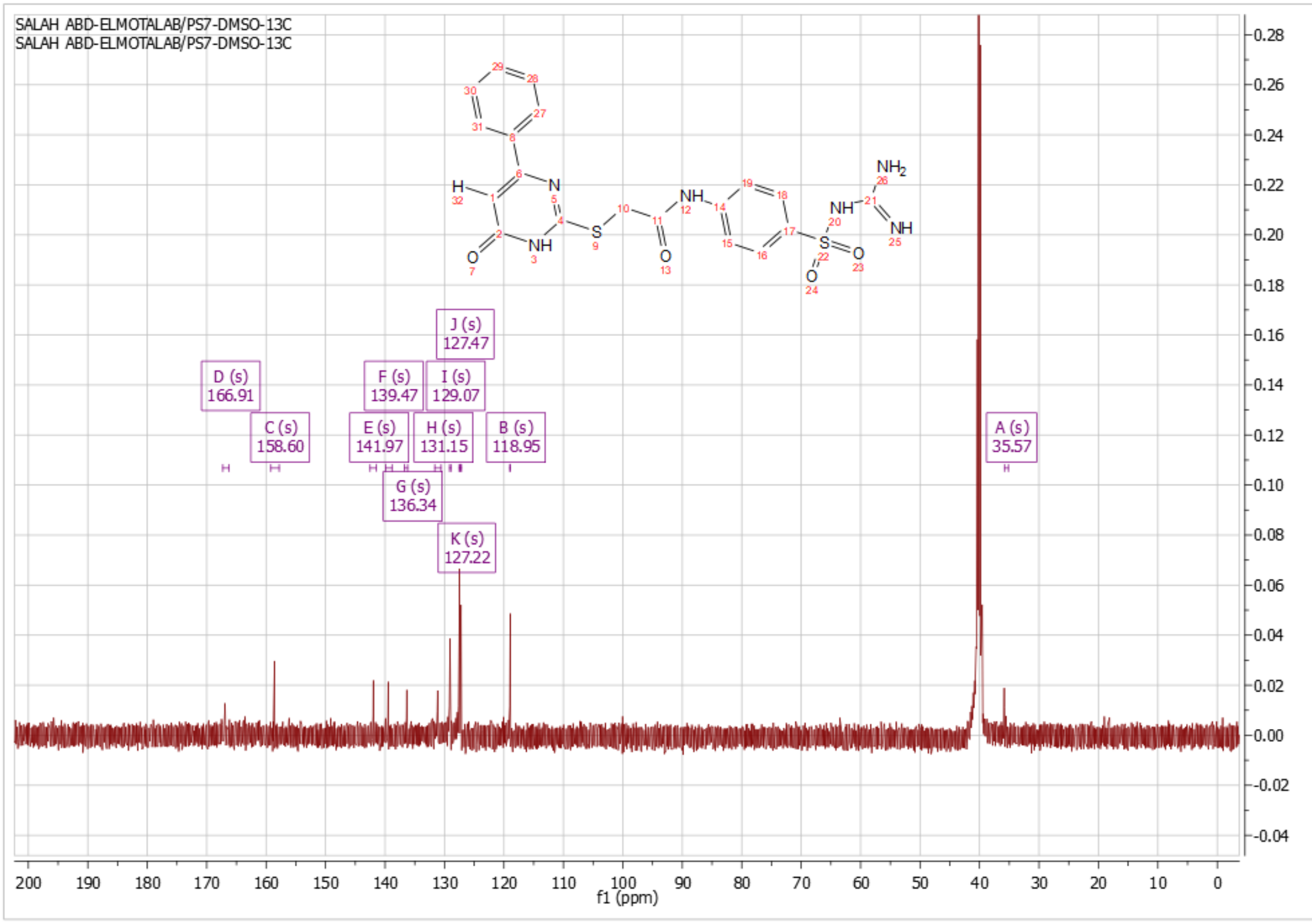

Figure S31: ¹H NMR of compound 4k

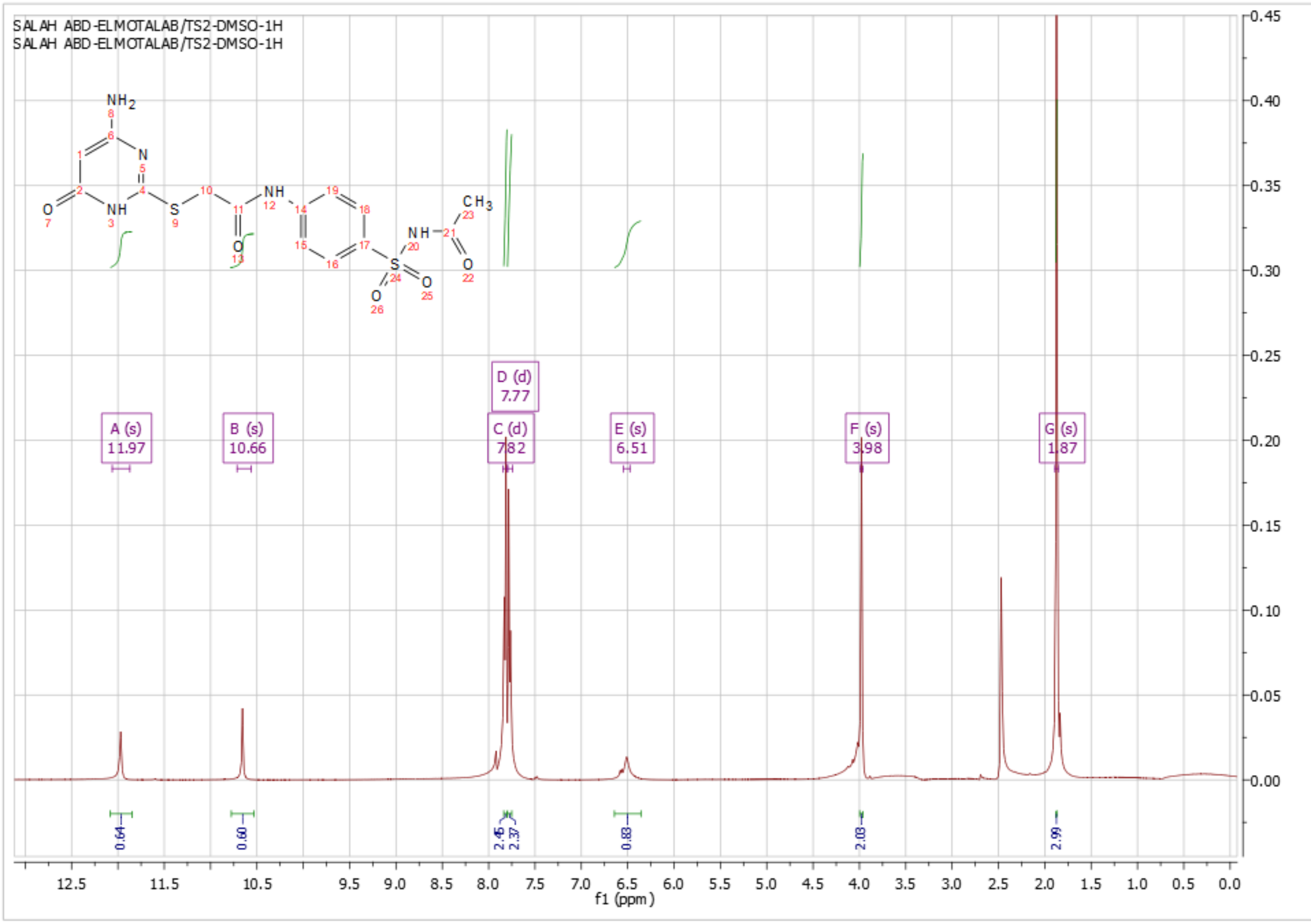

Figure S32: **Expanded ^{1}H NMR of compound 4k**

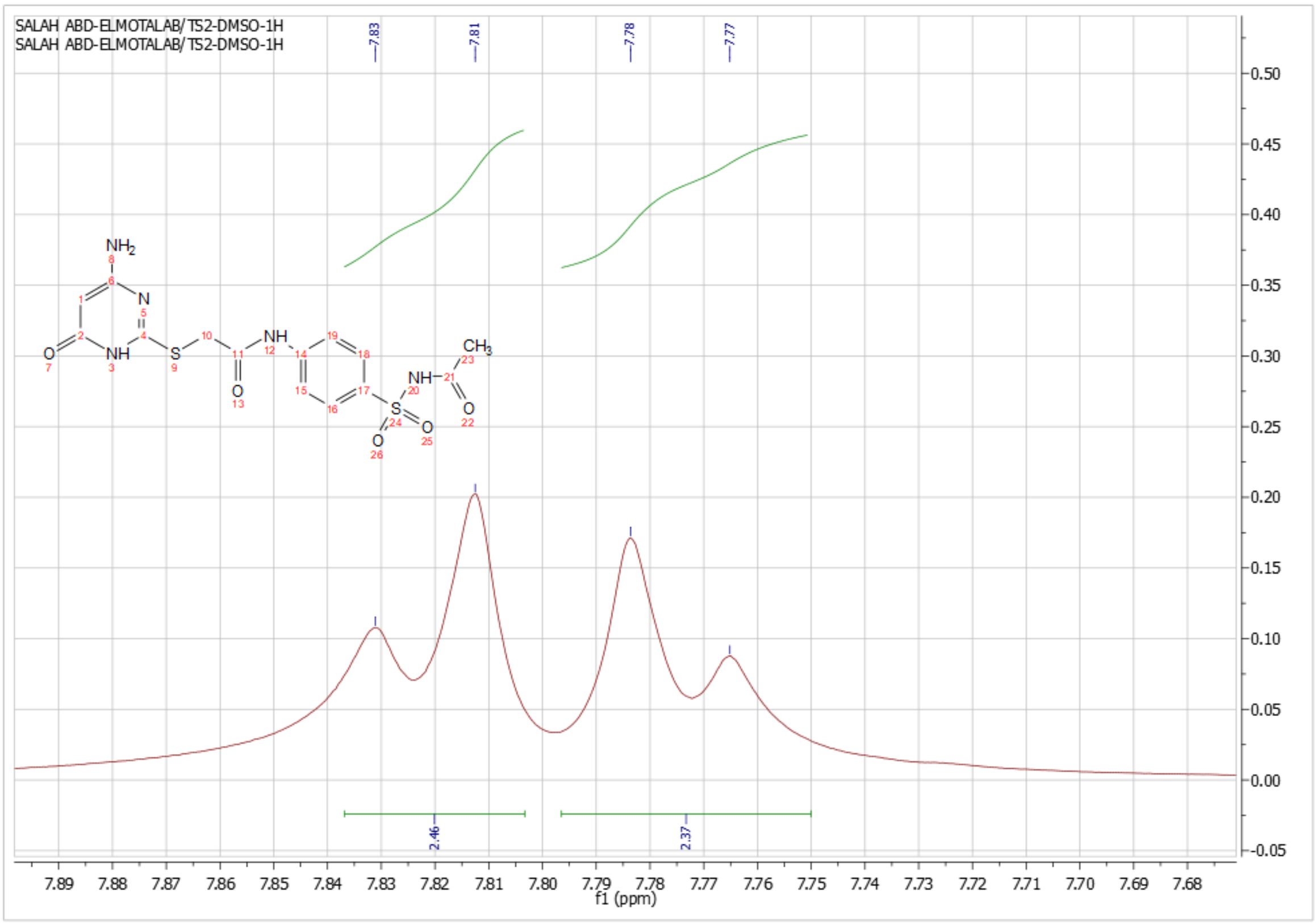

Figure S33: ^{13}C NMR of compound 4k

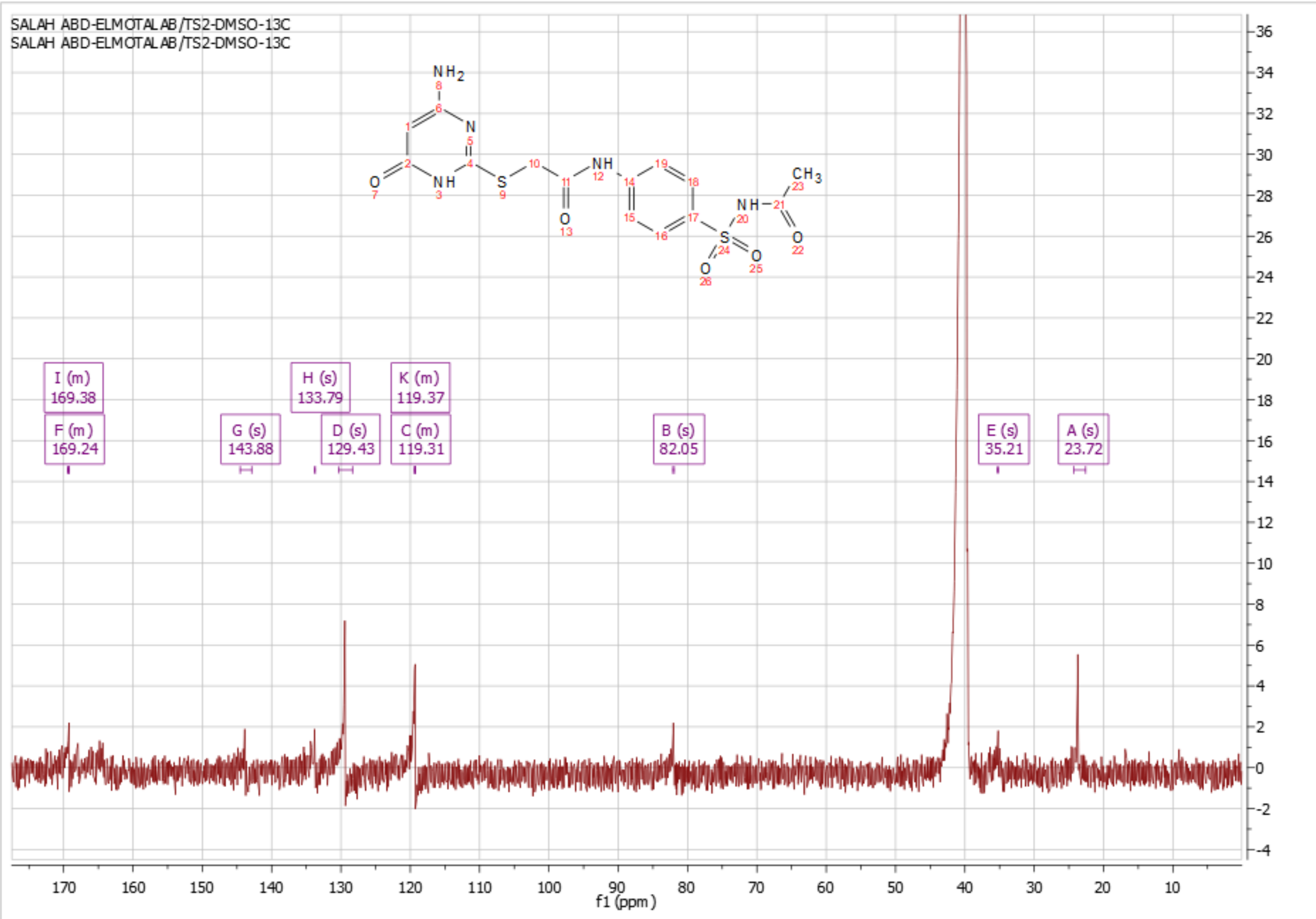

Figure S34: **^{1}H NMR of compound 4l**

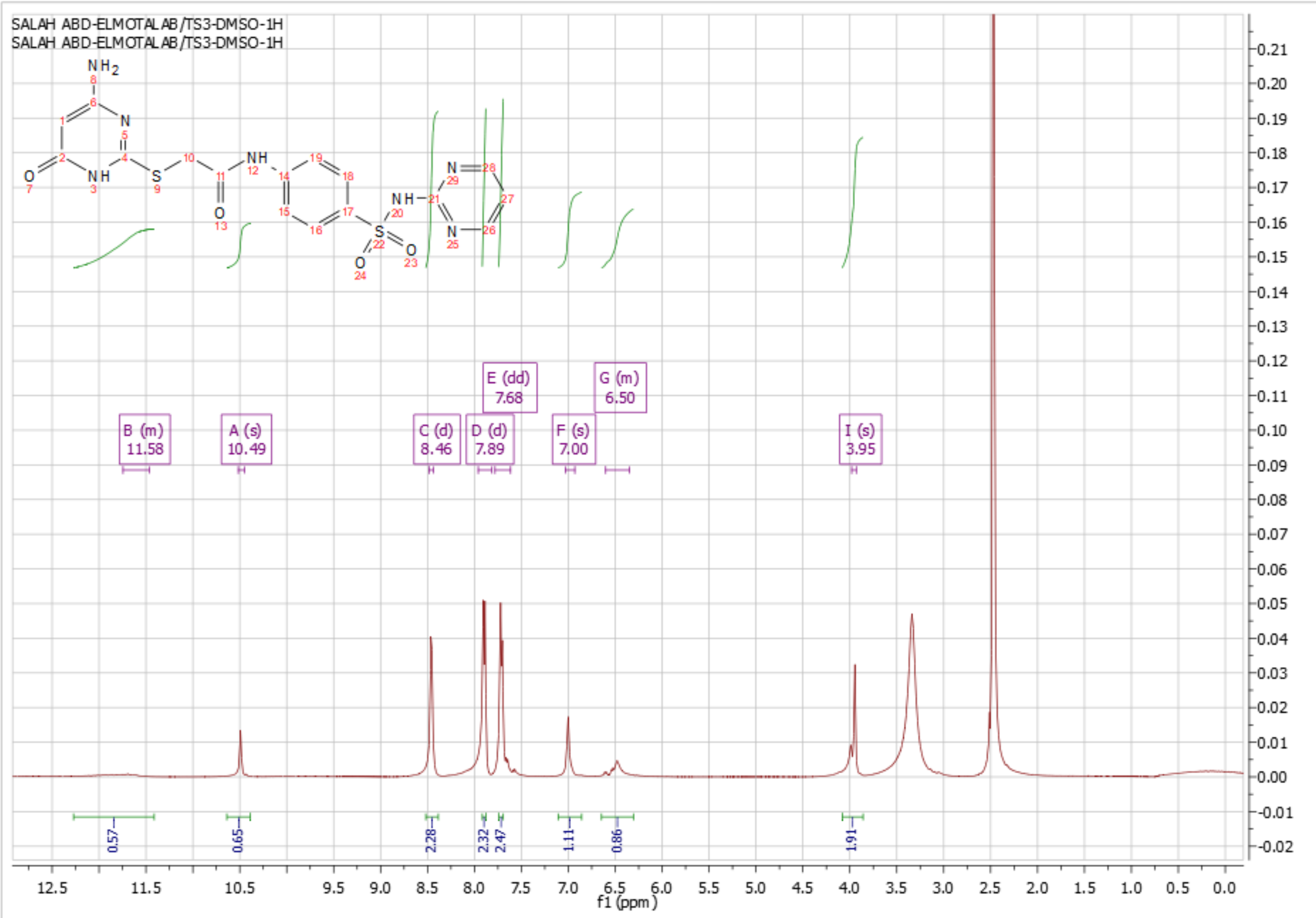

Figure S35: **Expanded ^{1}H NMR of compound 4l**

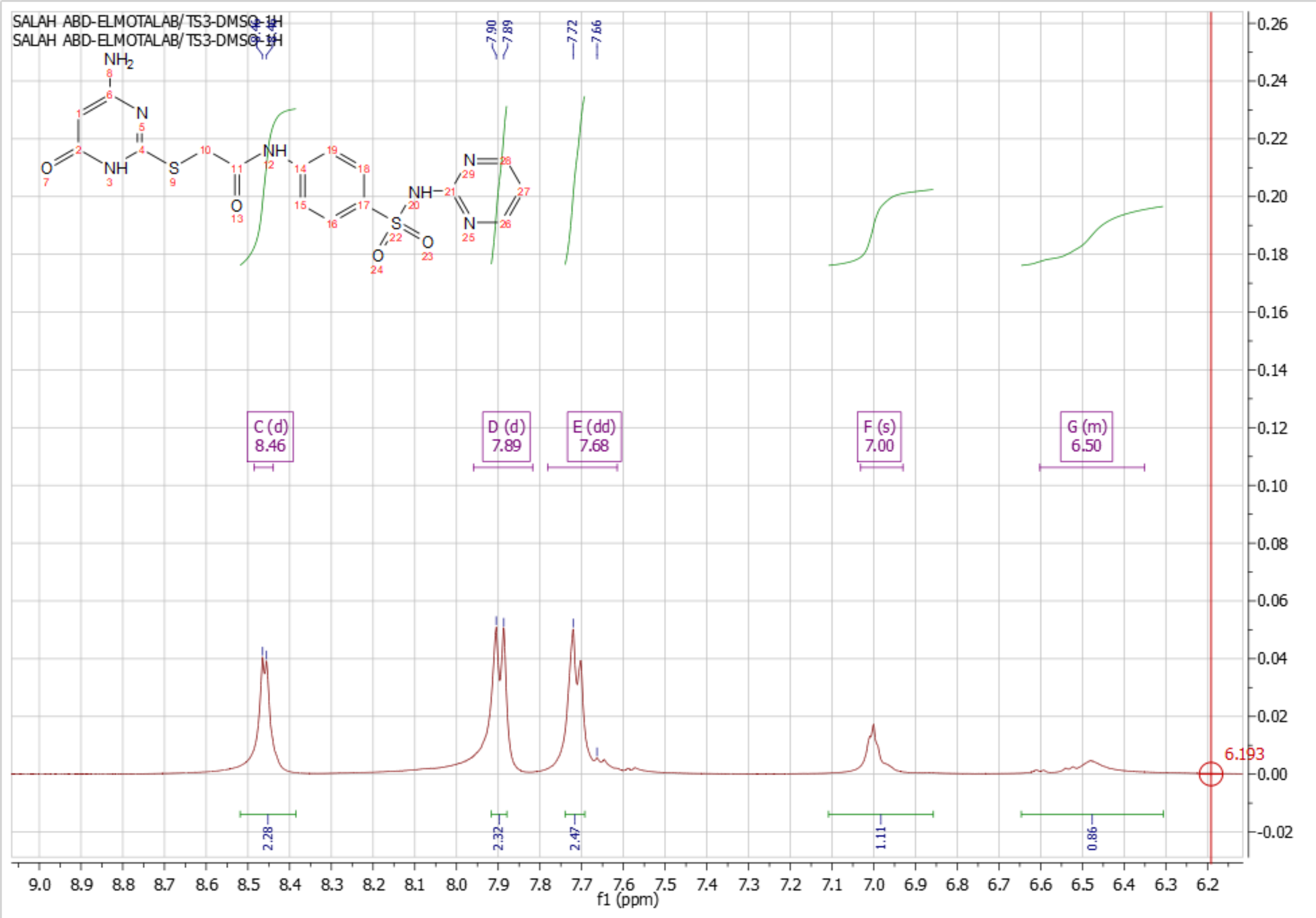

Figure S36: ^{13}C NMR of compound 4l

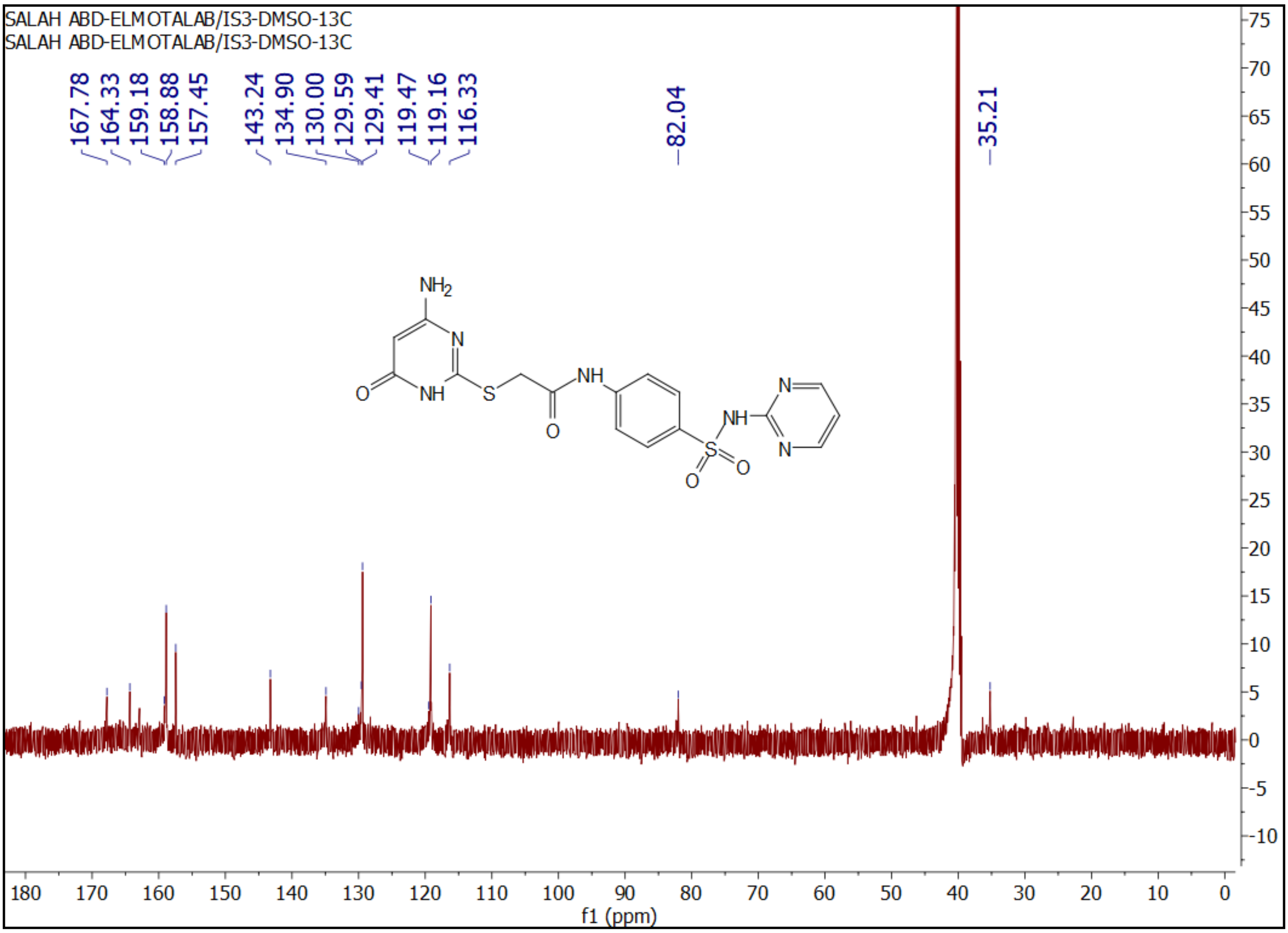

Figure S37: ^{1}H NMR of compound 4m

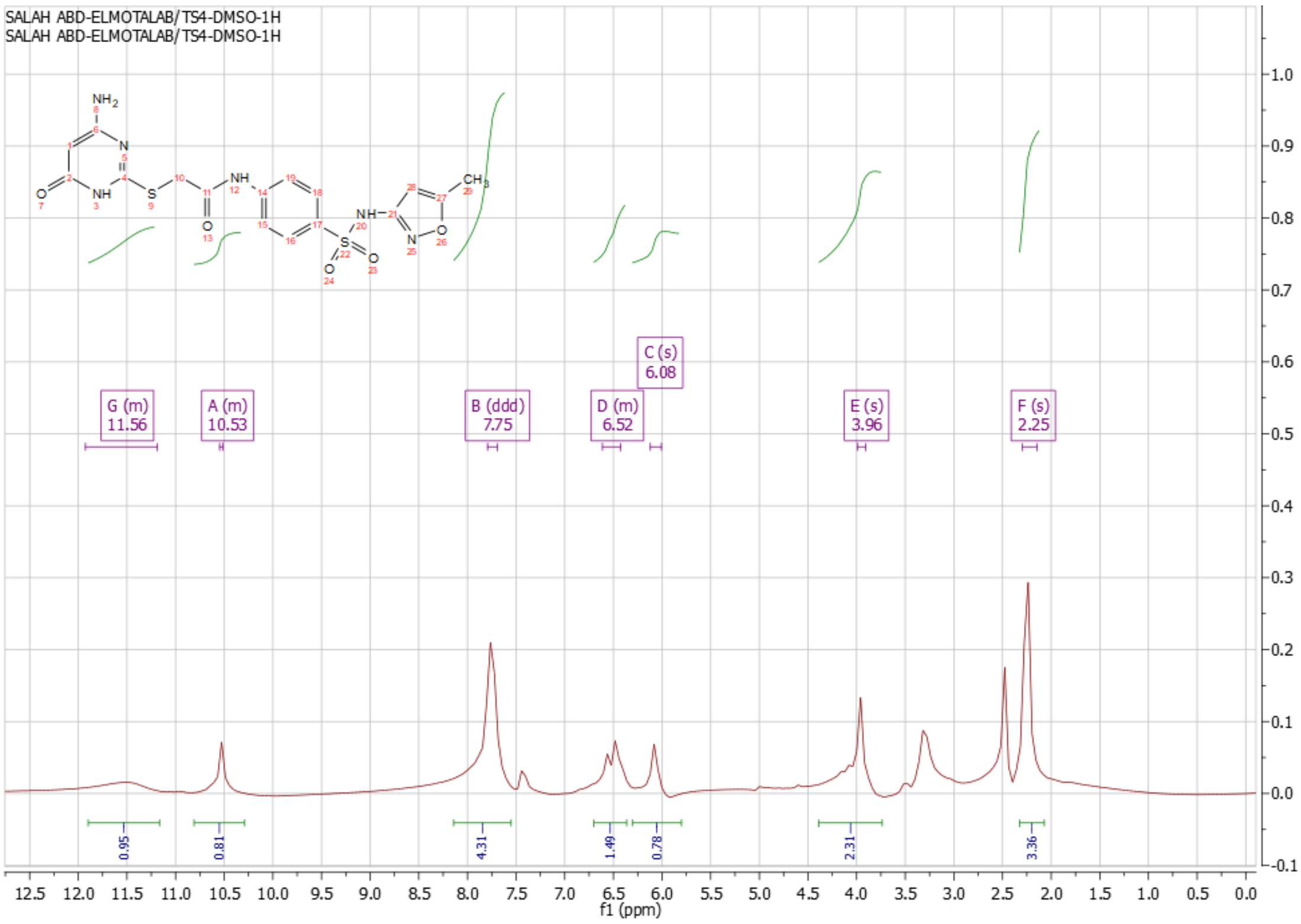

Figure S38: **Expanded ^{1}H NMR of compound 4m**

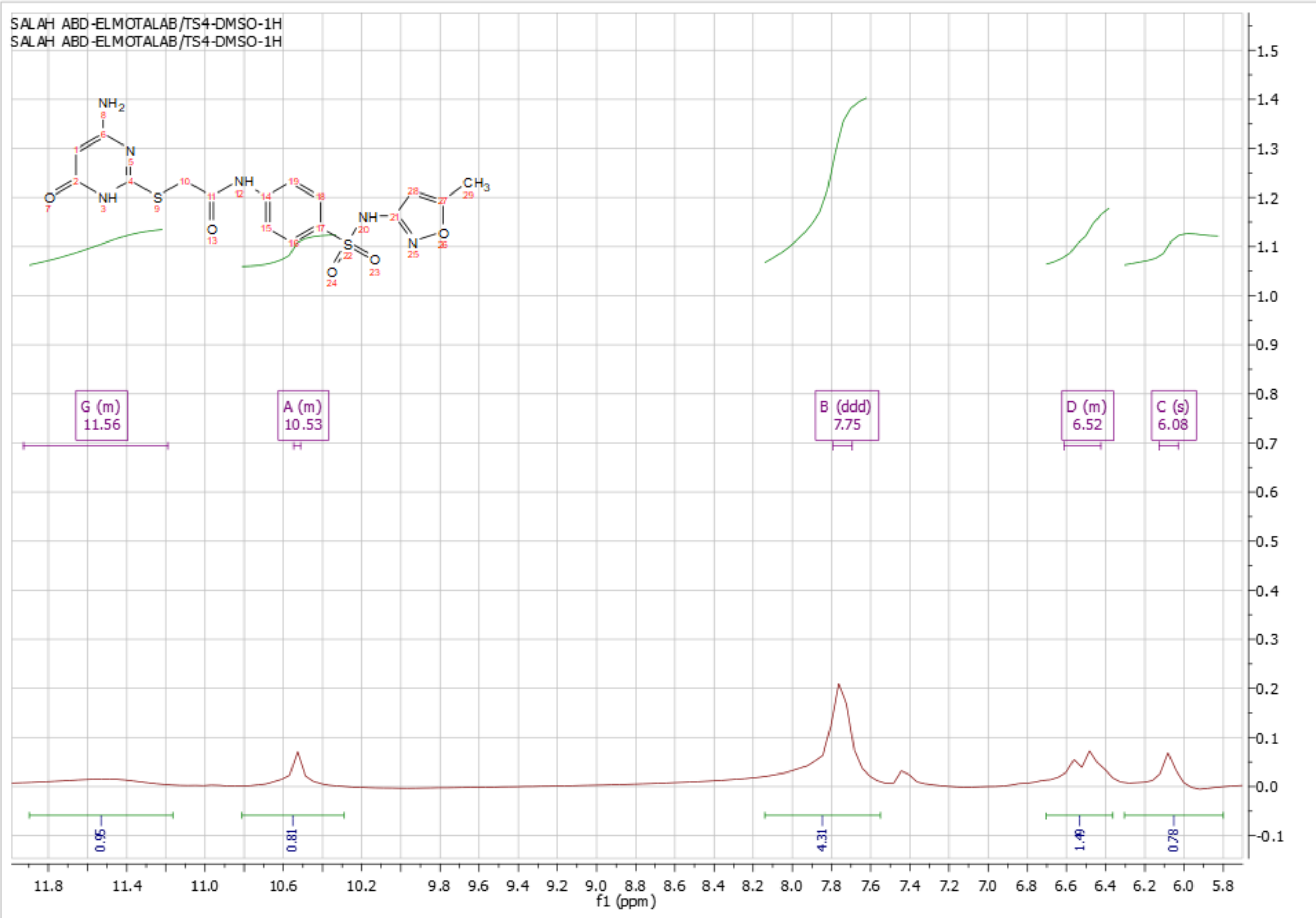

Figure S39: **^{13}C NMR of compound 4m**

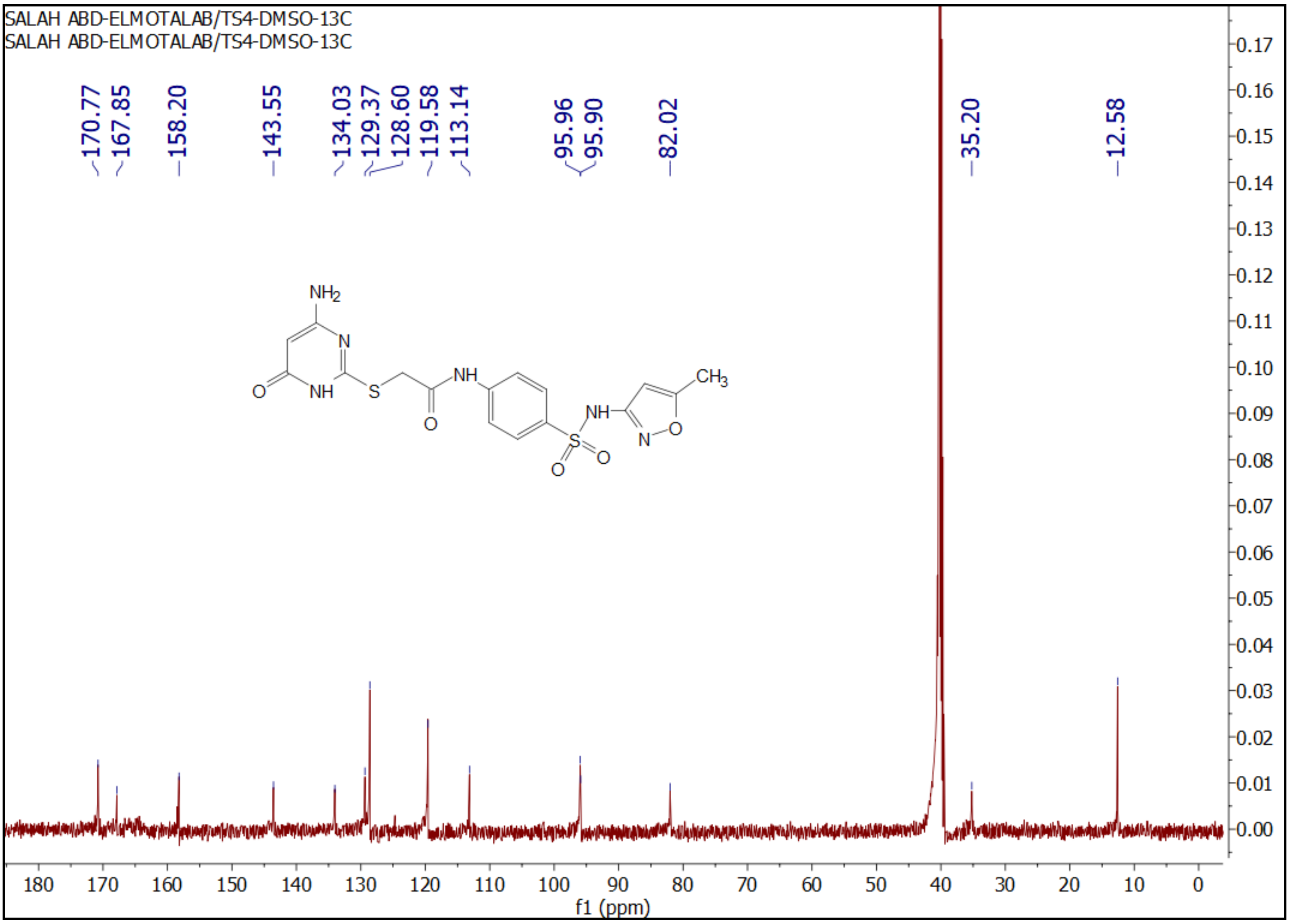

Figure S40: ^{1}H NMR of compound 4n

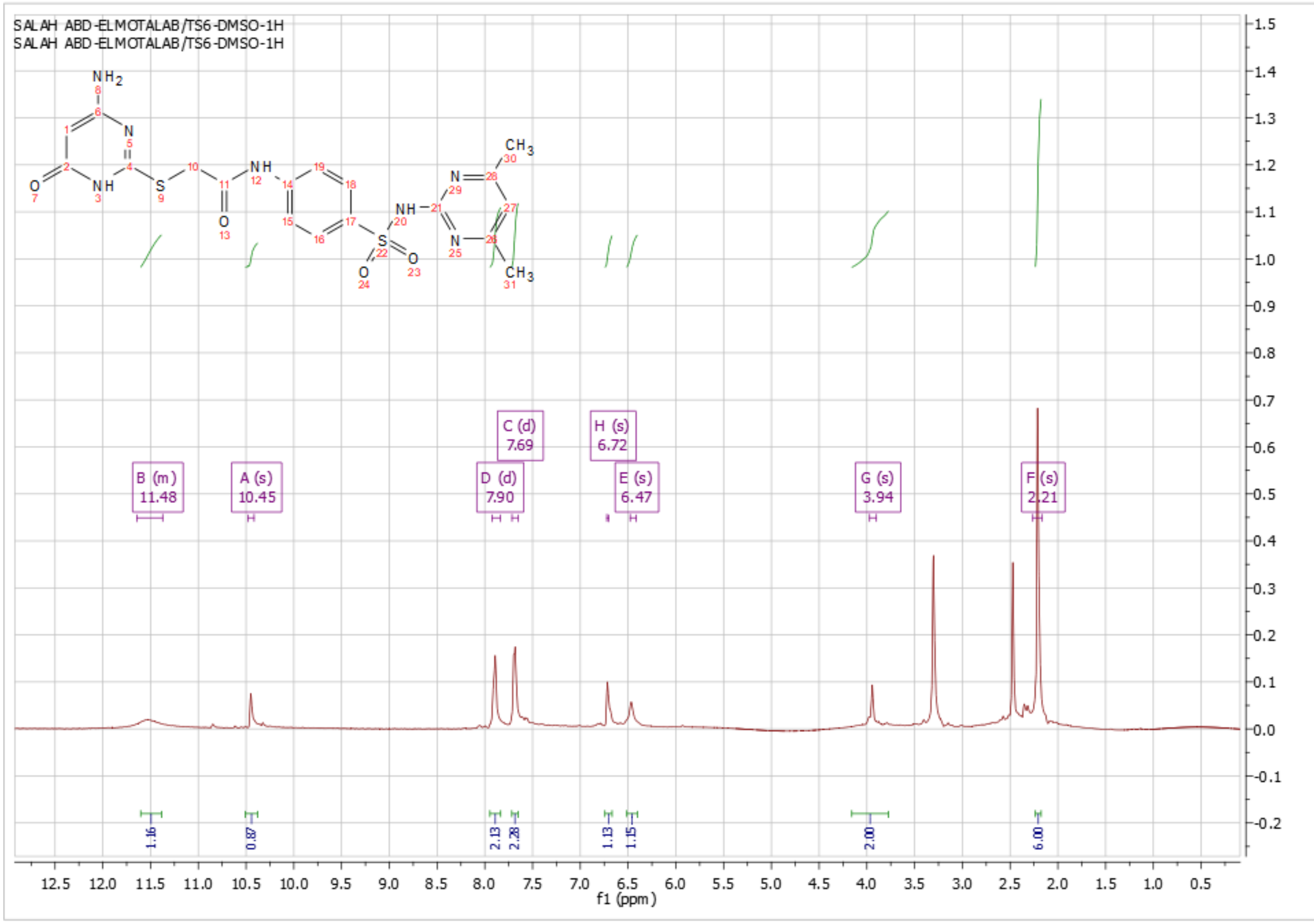

Figure S41: **Expanded ^{1}H NMR of compound 4n**

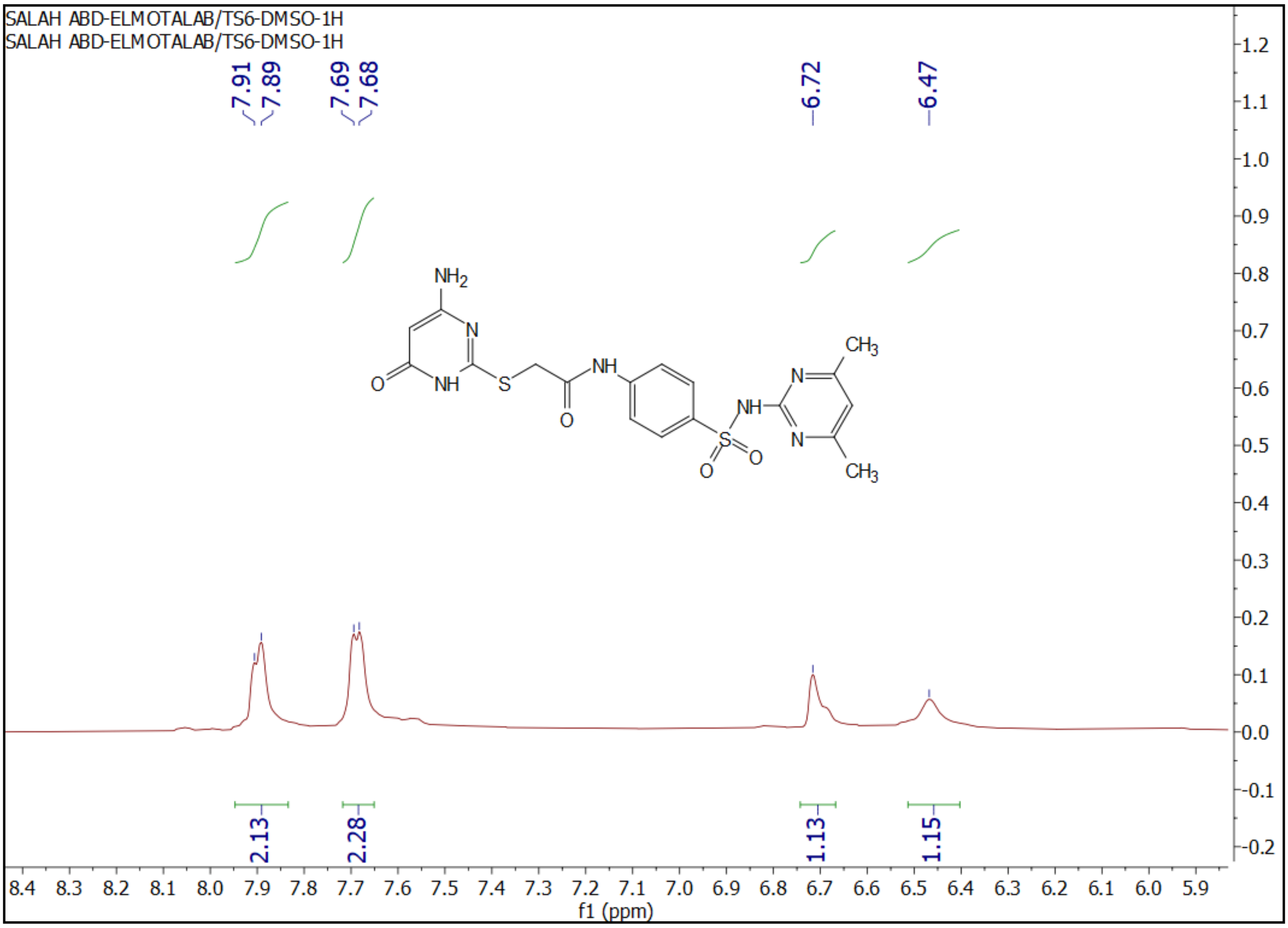

Figure S42: ^{13}C NMR of compound 4n

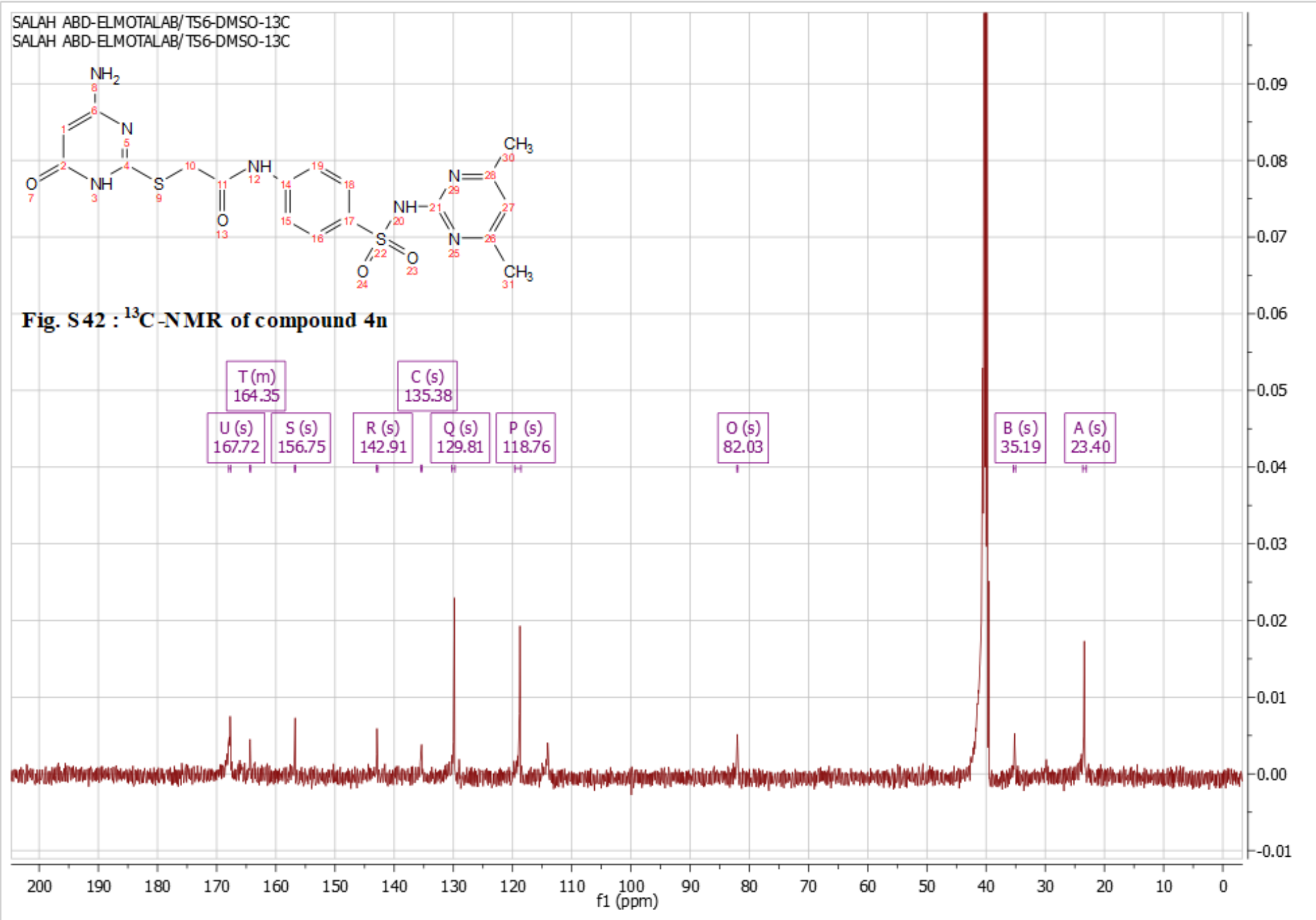

Figure S43: **^{1}H NMR of compound 4o**

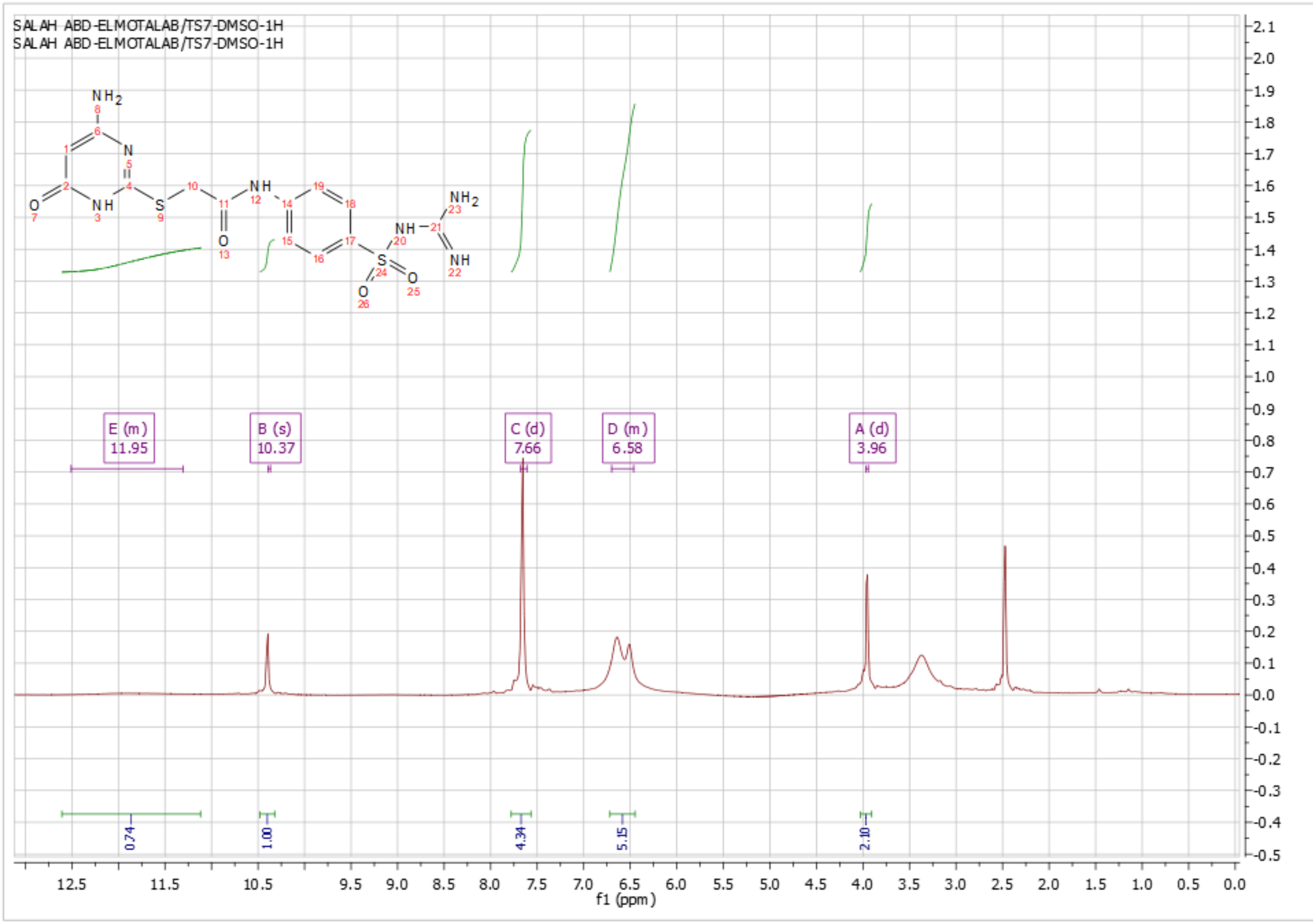

Figure S44: **Expanded ^{1}H NMR of compound 4o**

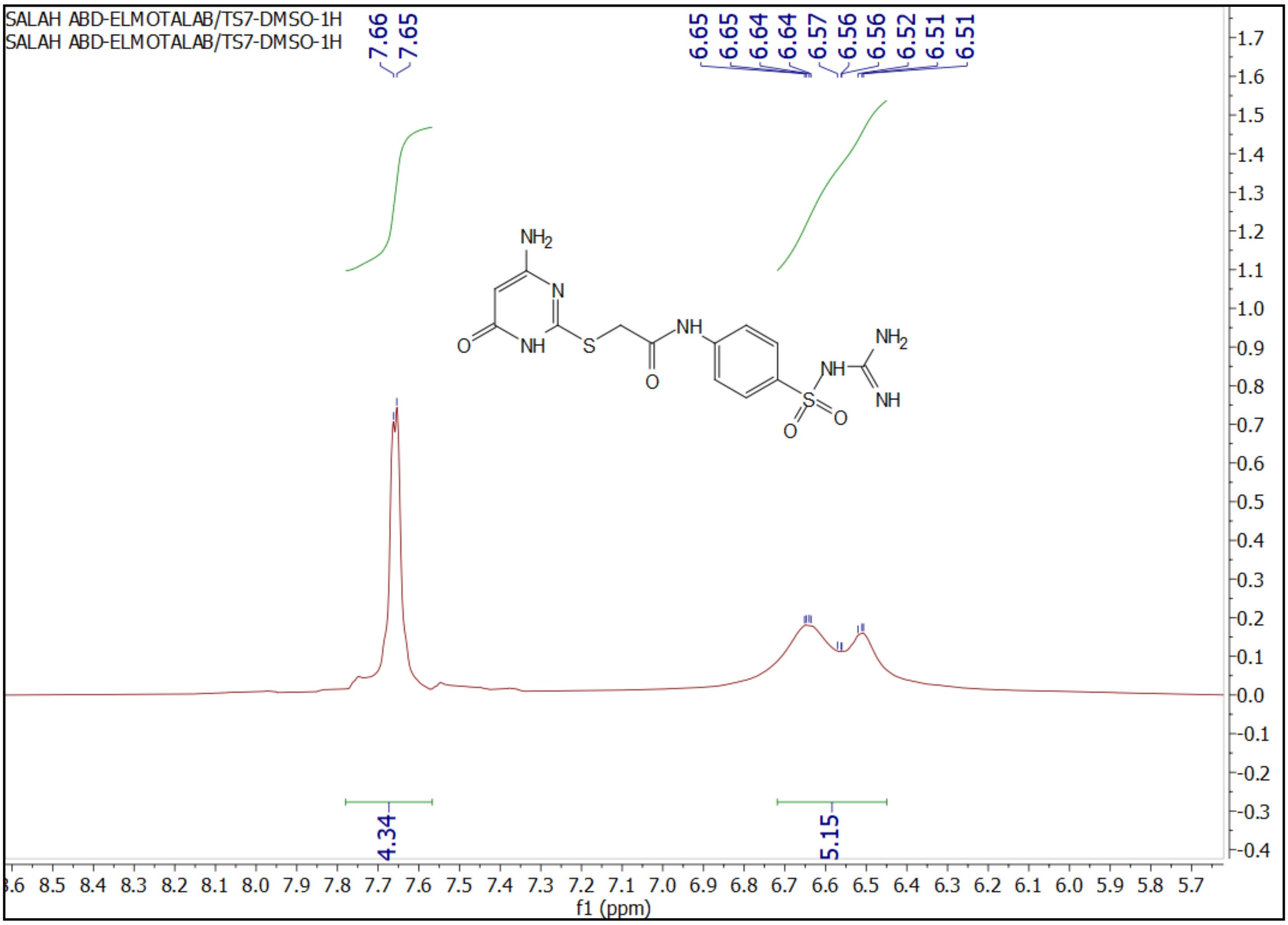

Figure S45: ^{13}C NMR of compound 4o

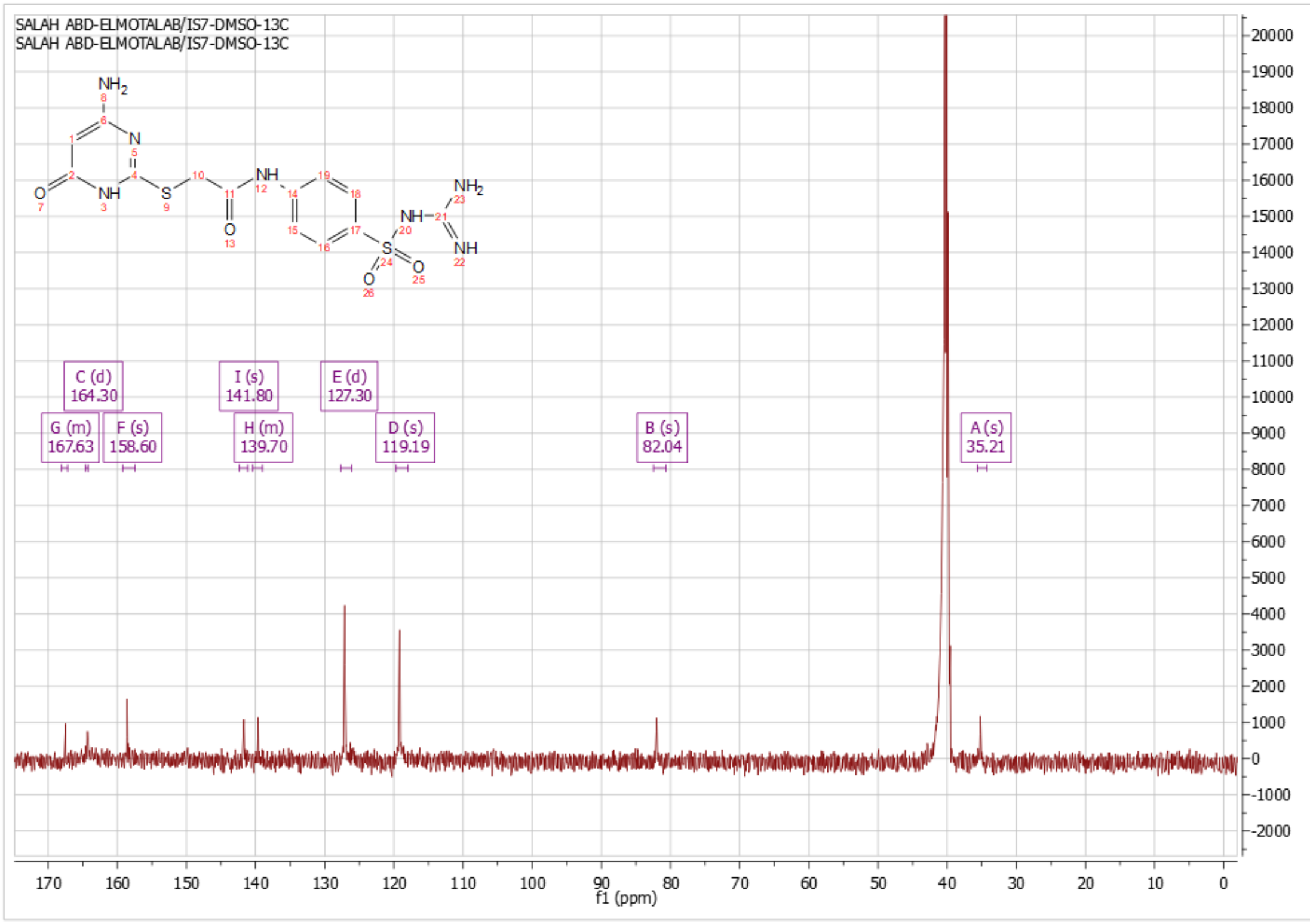

Figure S46: **EI-MS spectrum of compound 4a**

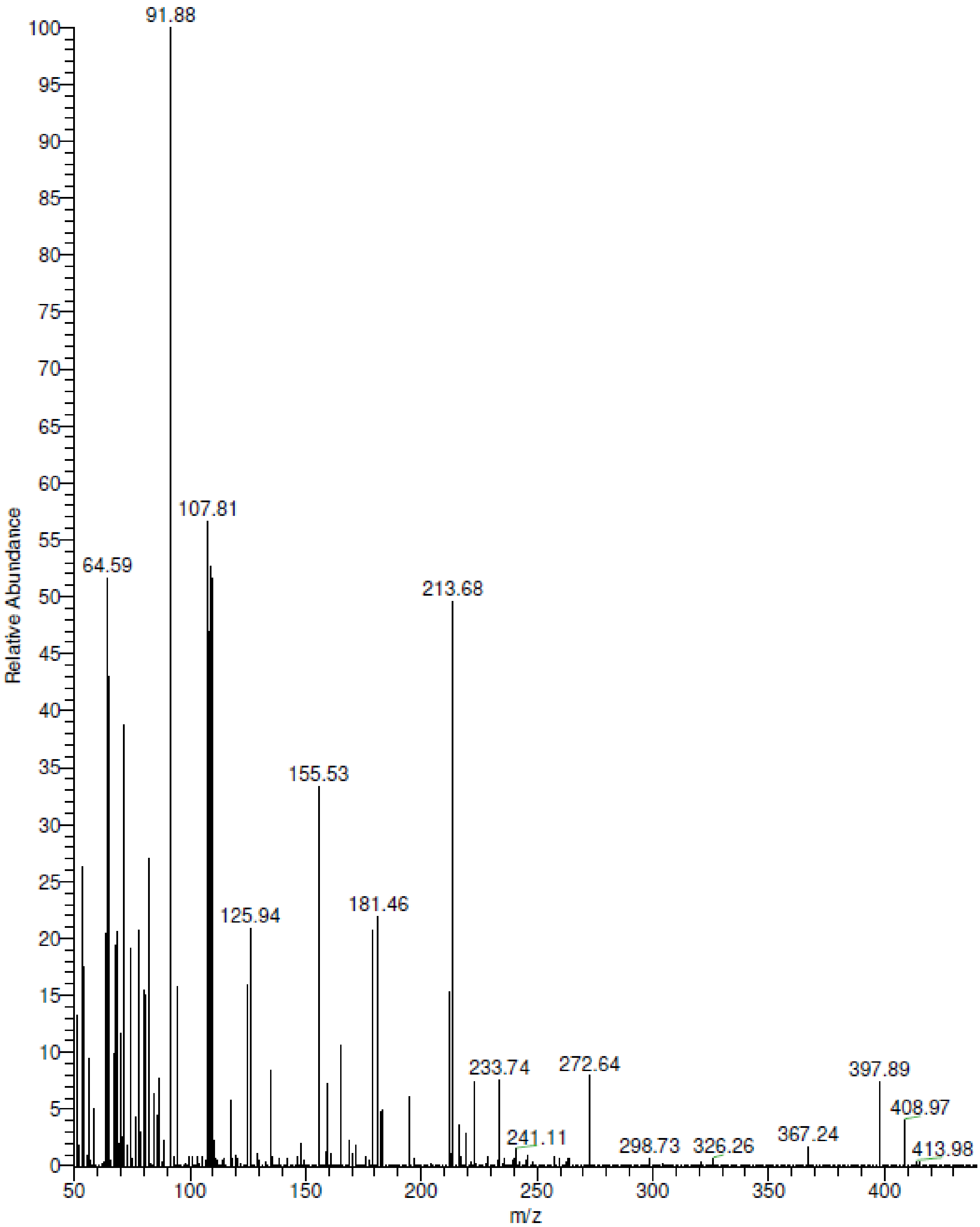

Figure S47: **EI-MS spectrum of compound 4b**

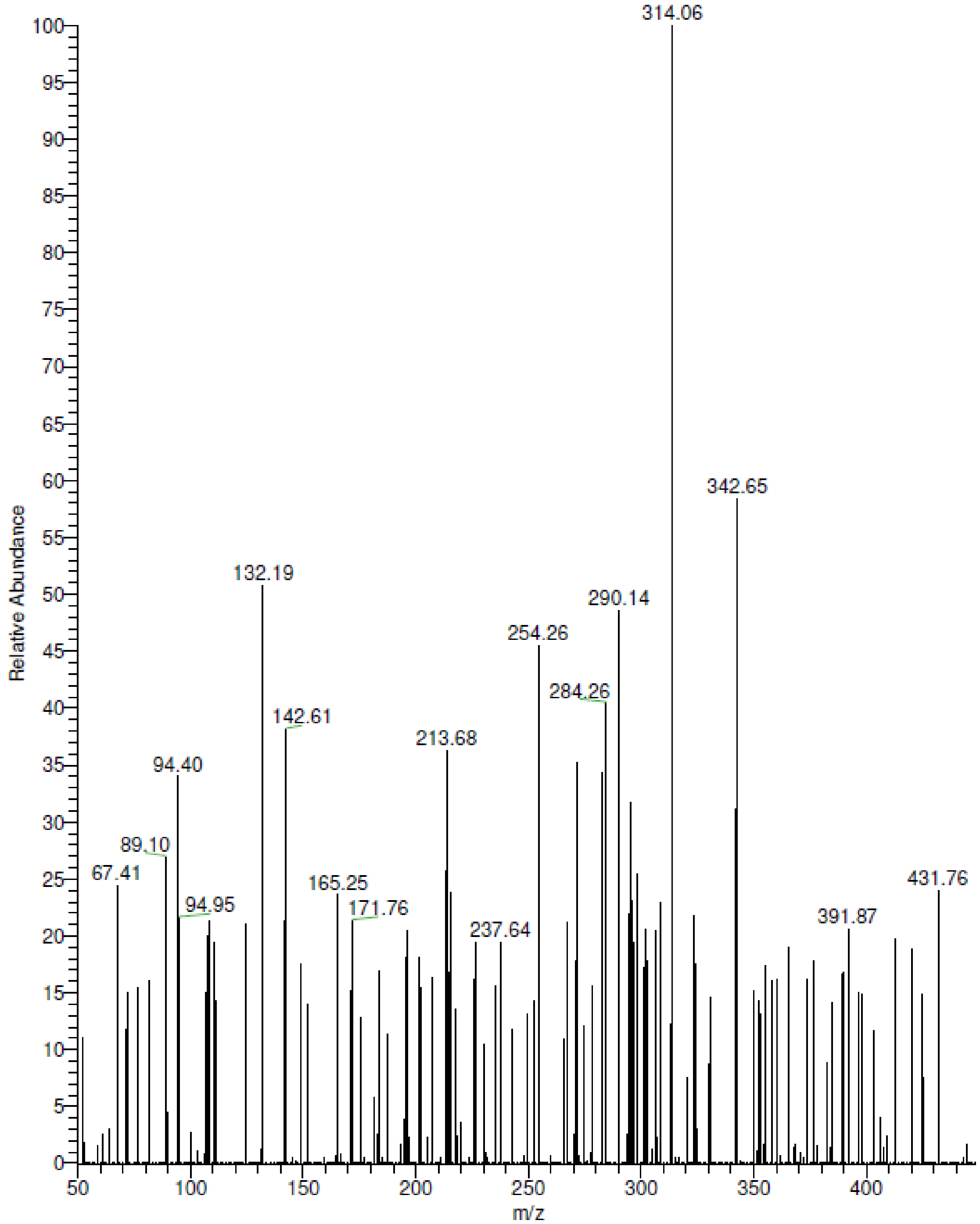

Figure S48: **EI-MS spectrum of compound 4d**

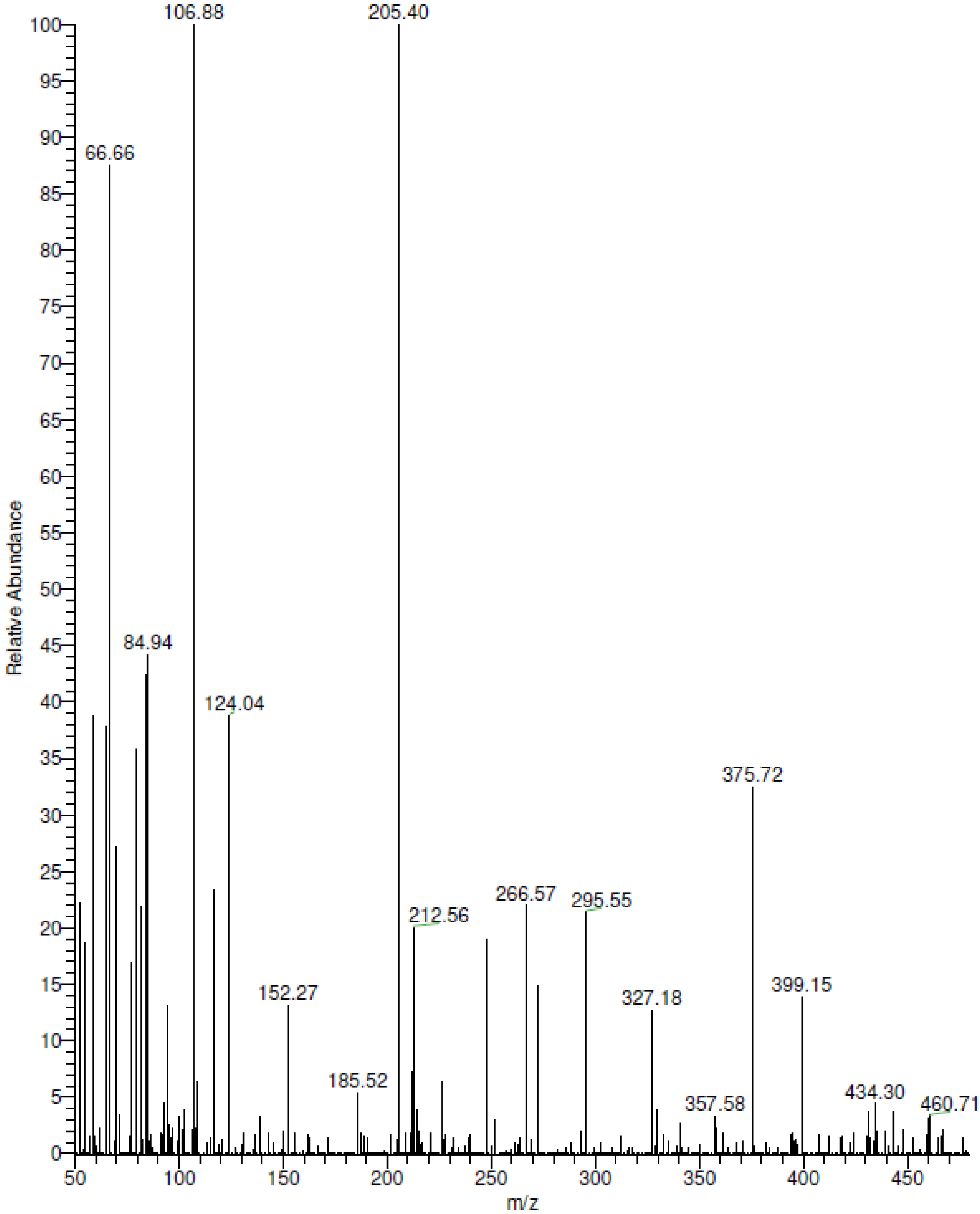

Figure S49: **EI-MS spectrum of compound 4g**

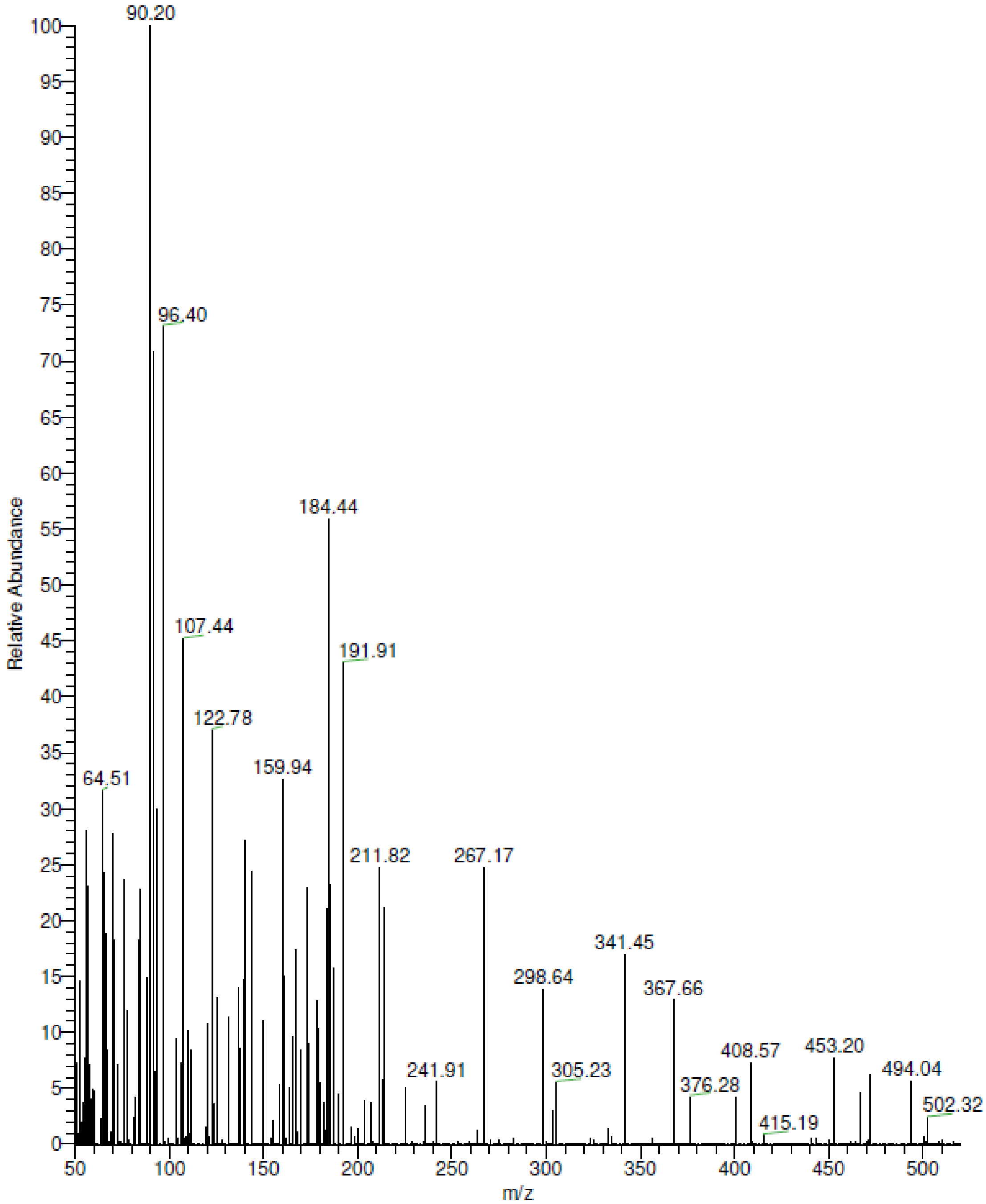

Figure S50: **EI-MS spectrum of compound 4i**

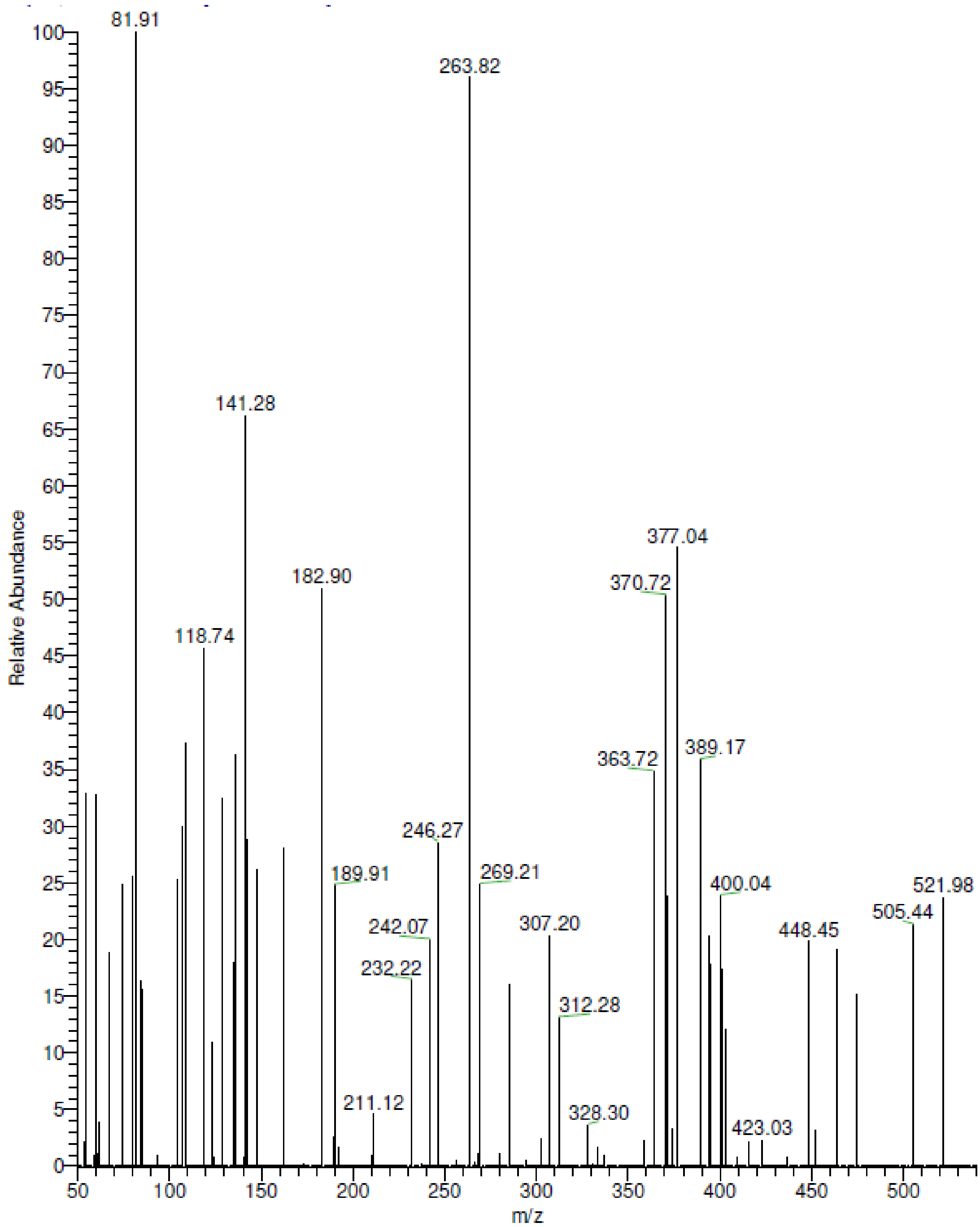

Figure S51: **EI-MS spectrum of compound 4j**

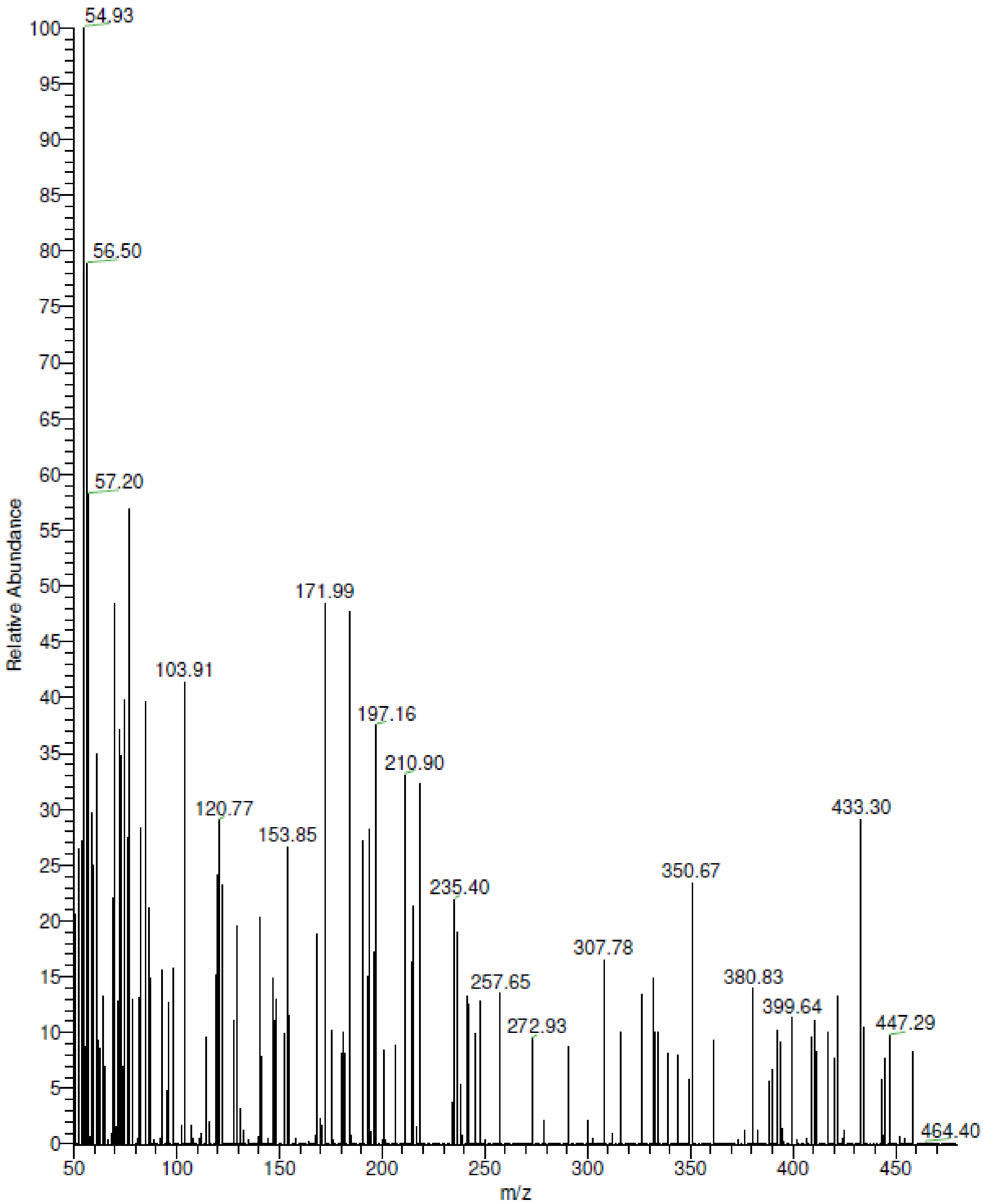

Figure S52: **EI-MS spectrum of compound 4k**

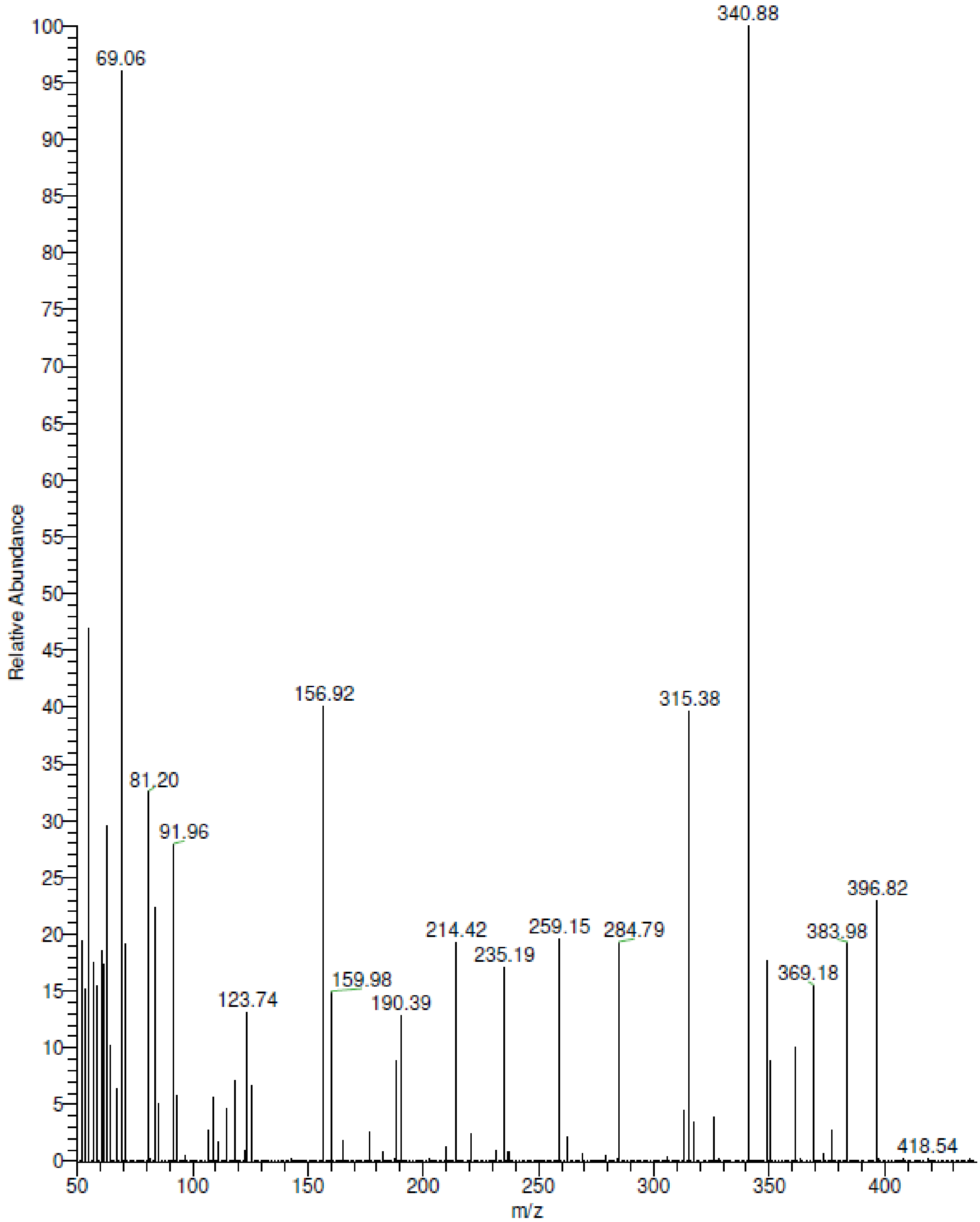

Figure S53: **EI-MS spectrum of compound 4l**

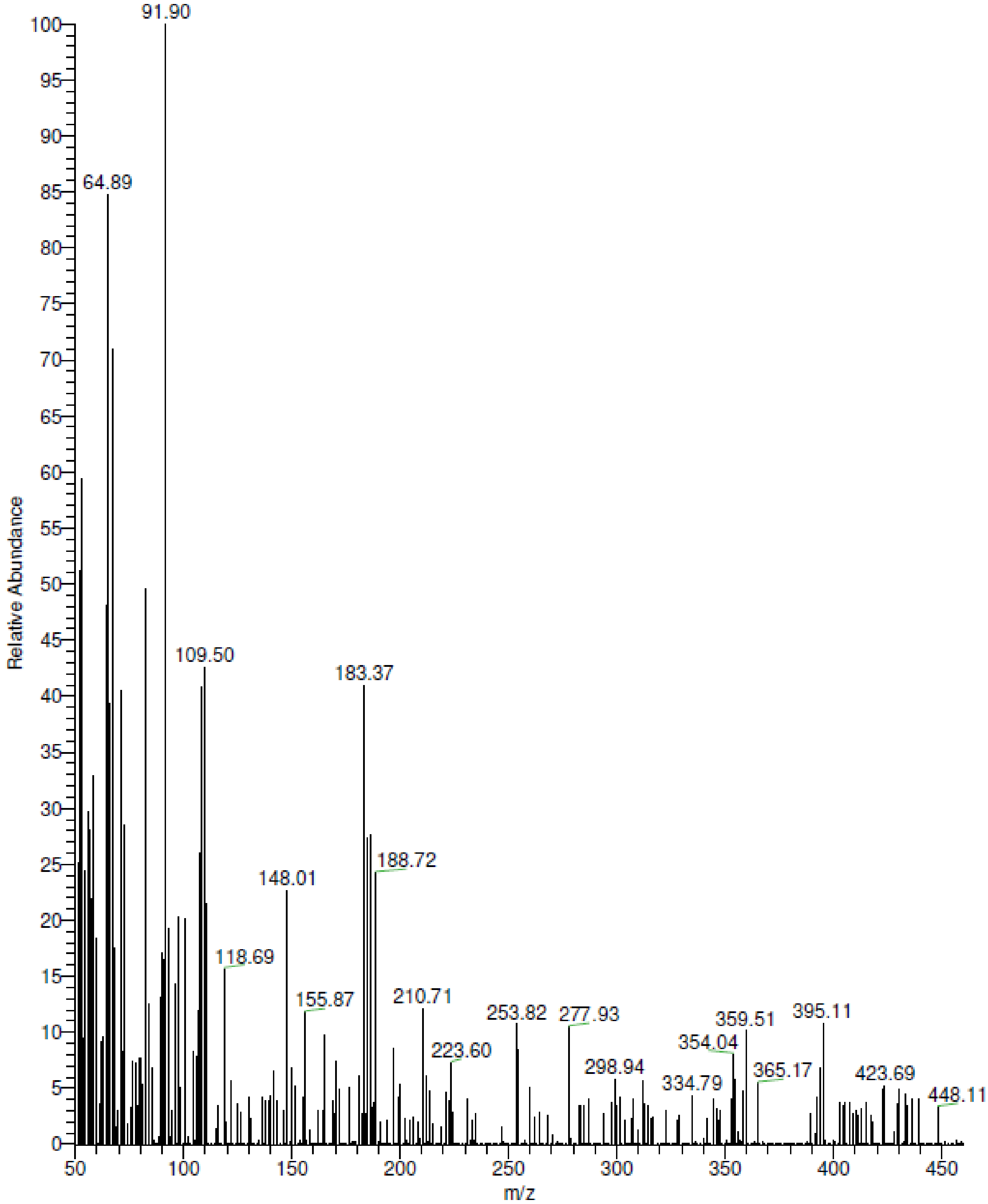

Figure S54: **EI-MS spectrum of compound 4m**

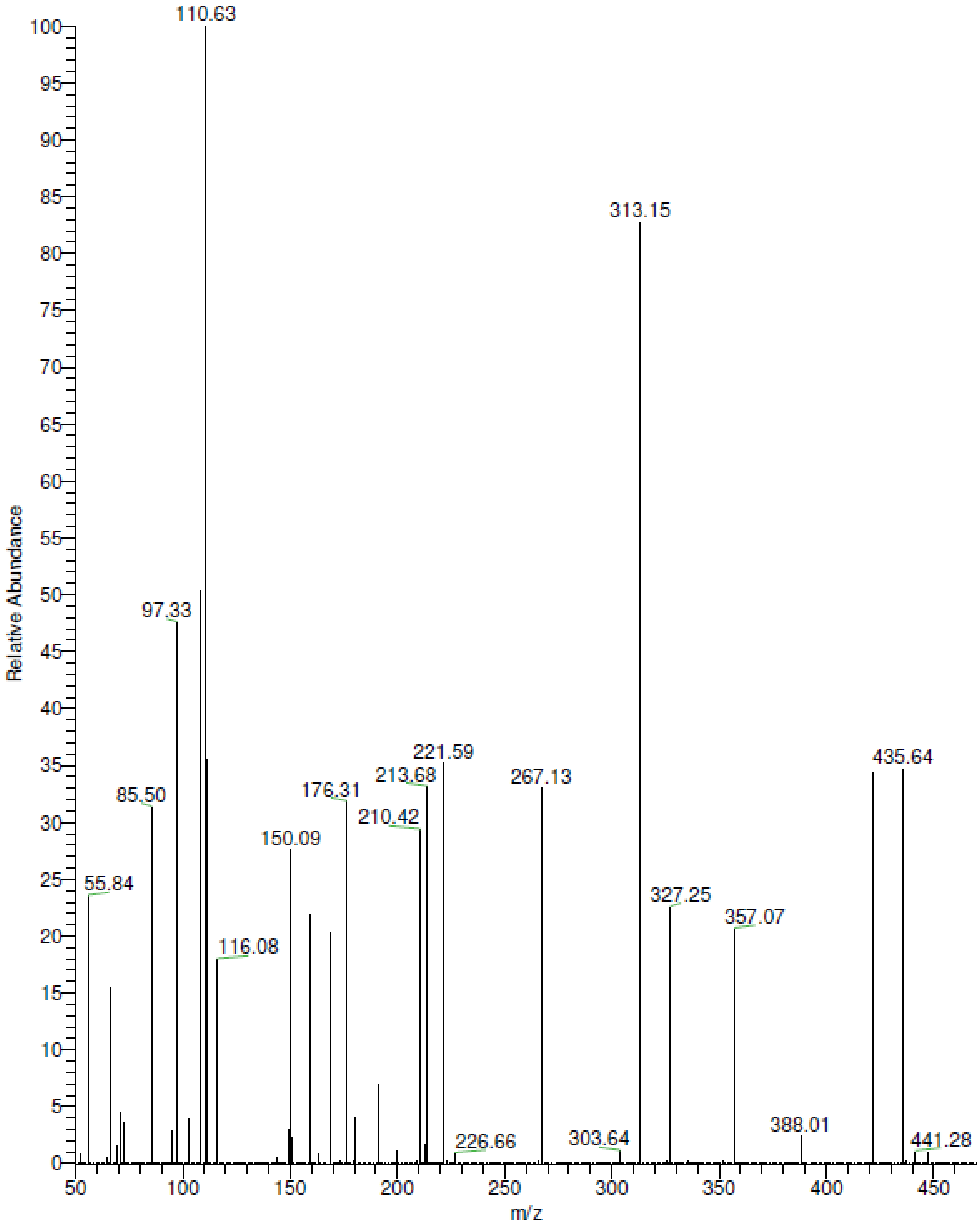

Figure S55: **EI-MS spectrum of compound 4o**

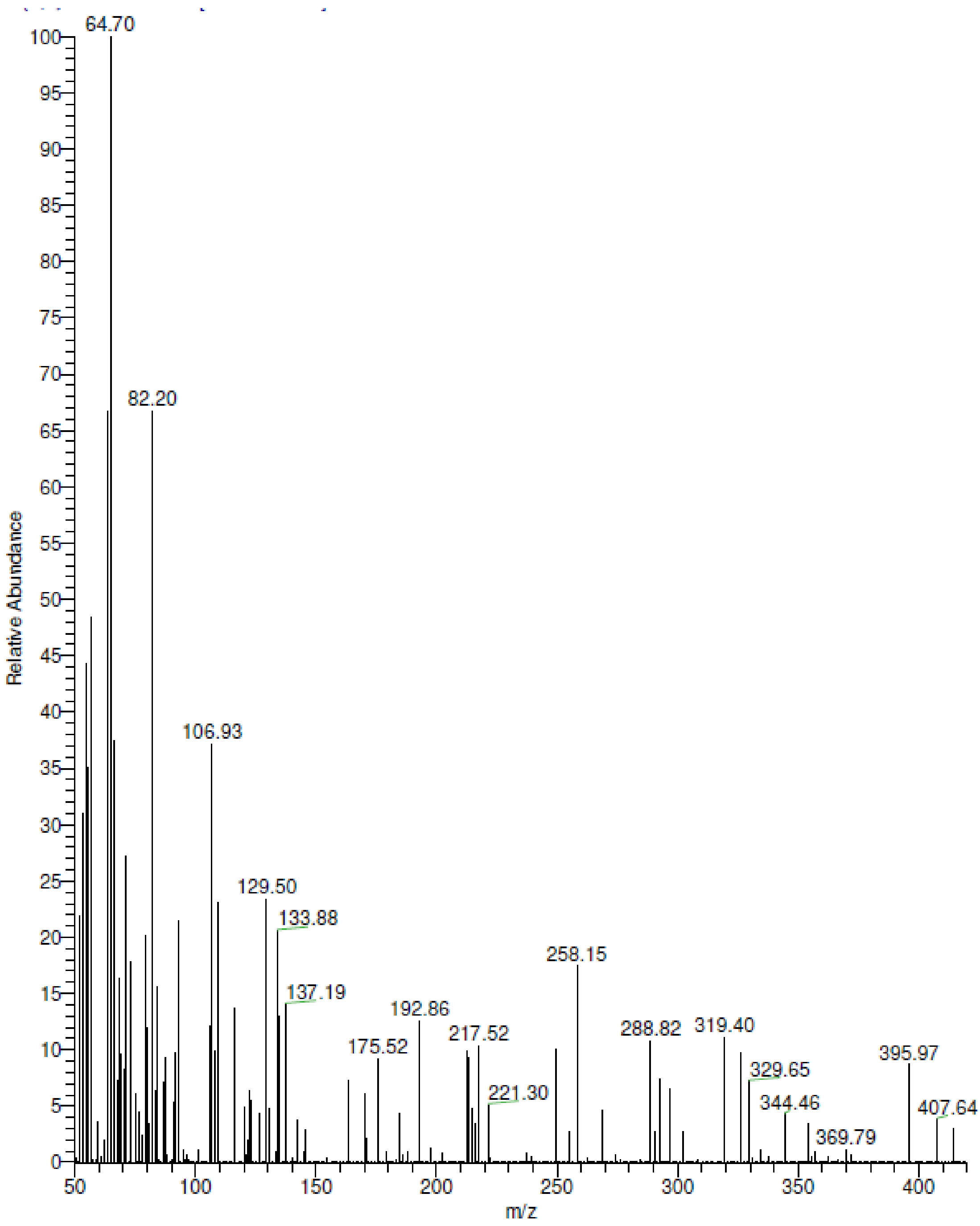

Appendix A

4. Experimental

4.1. Chemistry

General Information

All solvents were used as purchased from Merck (St. Louis, MO, USA). The progress of all reactions was monitored with thin-layer chromatography (TLC) on Merck alumina-backed TLC plates and visualized under UV light. Spectra were measured in DMSO-*d6* on a Bruker AV-500 spectrometer (500 MHz for ^{1}H and 126 MHz for ^{13}C), in the Chemistry Department, Faculty of Science, Cairo University, Egypt. Chemical shifts are expressed in δ (ppm) versus internal Tetramethylsilane (TMS) = 0 ppm for ^{1}H and ^{13}C and the coupling constants are stated in Hz. Splitting patterns are denoted as follows: singlet (s), doublet (d), multiplet (m), triplet (t), quartet (q), doublet of doublets (dd), doublet of triplets (dt), triplet of doublets (td), and doublet of quartet (dq). Melting points (mp) were determined with a Stuart melting point instrument in the Chemistry Department, Faculty of Science, Minia University, El Minia, 61519 Egypt, and are expressed in °C. Elemental analyses were carried out on a Perkin Elmer device at the Microanalytical center of Organic Chemistry, Al-Azhar university, Egypt

4.2. Biological evaluation

4.2.1. *In vitro* COX-1 and COX-2 inhibition assays

All the newly synthesized compounds were screened for their ability to inhibit COX-1 and COX-2 enzymes using ten folds serial dilutions (1, 0.1, 0.01, 0.001 mg/mL). This was carried out using the COX 1(human) Inhibitor Screening Assay Kit and COX 2 (human) Inhibitor Screening Assay Kit (supplied by Cayman chemicals (catalog no. 560131), Ann Arbor, MI, USA). The preparation of reagents and testing procedures were according to the instructions recommended by the supplier. COX catalyzes the first step in the biosynthesis of arachidonic acid to PGH2. The PGF2α produced from PGH2 by reduction with stannous chloride is measured by enzyme-linked immunosorbent assay (ELISA). In brief, the compounds were dissolved in dimethyl sulfoxide (DMSO). The enzyme COX-1 and COX-2 (10 mL), heme (10 mL), and samples (20 mL) were added to the supplied reaction buffer solution (160 mL, 0.1M Tris-HCl, pH 8 containing 5 mM ethylenediaminetetraacetate (EDTA), and 2 mM phenol) and pre-incubated for 10 min in a water bath (37 ^{o}C). After that, COX reactions were initiated by the addition of arachidonic acid (10 mL, final concentration in reaction mixture 100 mM). After 2 min, The COX reactions were stopped by the addition of saturated stannous chloride (30 mL) followed by incubation for 5 min at room temperature. The PGF2α formed in the samples by COX reactions was quantified by ELISA. Following transfer to a 96-well plate, the plate was incubated with samples for 18 h at room temperature. After incubation, the plate was washed to remove any unbound reagent, and then Ellman's reagent (200 mL), which contains substrate to

acetylcholinesterase, was added and incubated at room temperature for 60-90 min until the absorbance of Bo well is in the range 0.3e0.8 AU. at 410 nm. The plate was then read by an ELISA plate reader. The IC_{50} of inhibition of COX-1 and COX-2 was calculated by the comparison of the sample treated incubations to control incubations. Celecoxib was used as the reference standard drug in the study.

4.2.2. *In vitro* 5-LOX inhibitory assay

The newly synthesized compounds were screened for their ability to inhibit lipoxygenase enzymes. This was carried out using Abnova lipoxygenase inhibitor screening assay kit (Catalog No. 760700) according to reported method.

4.2.3. *In vitro* sEH assay

IC_{50} values were determined using a cell-based assay system of 96-well format [35]. Epoxy Flour 7, a sensitive fluorescent substrate, was utilized to monitor the activity of the enzyme through its hydrolysis by sEH to the fluorescent 6-methoxy-2-naphthaldehyde that could be monitored (λem = 330 nm, λex = 465 nm). Briefly, the assay was carried out through incubation of 10 mL of compounds buffer or 10 mL of the AUDA solution to appropriate wells. For positive control wells, 100 mL of the 10 ng/mL sEH positive control (substrate) was prepared for two wells. Then, 200 mL of 6-methoxy-2-Naphthaldehyde was added. Standards were prepared above to corresponding wells of the black plate. 100 mL of the substrate solution was added to each well, except the standards, and the plate was allowed to incubate at 37 ^{o}C for 30 min. The fluorescent intensity of each well (excitation = 330 nm; emission = 465 nm) was read.

4.2.4 Cell Viability assay (MTT assay)

MTT assay was performed to investigate the effect of the synthesized compounds on mammary epithelial cells (MCF-10A). The cells were propagated in medium consisting

of Ham's F-12 medium/ Dulbecco's modified Eagle's medium (DMEM) (1:1) supplemented with 10% foetal calf serum, 2 mM glutamine, insulin (10 µg/mL), hydrocortisone (500 ng/mL) and epidermal growth factor (20 ng/mL). Trypsin ethylenediamine tetra acetic acid (EDTA) was used to passage the cells after every 2-3 days. 96-well flat-bottomed cell culture plates were used to seed the cells at a density of 104 cells mL^{-1}. The medium was aspirated from all the wells of culture plates after 24 h followed by the addition of synthesized compounds (in 200 µL medium to yield a final concentration of 0.1% (v/v) dimethyl sulfoxide) into individual wells of the plates. Four wells were designated to a single compound. The plates were allowed to incubate at 37°C for 96 h. Afterwards, the medium was aspirated and 3-[4,5-dimethylthiazol-2-yl]-2,5-diphenyltetrazolium bromide (MTT) (0.4 mg/mL) in medium was added to each well and subsequently incubated for 3 h. The medium was aspirated and 150 µL dimethyl sulfoxide (DMSO) was added to each well. The plates were vortexed followed by the measurement of absorbance at 540 nm on a microplate reader. The results were presented as inhibition (%) of proliferation in contrast to controls comprising 0.1% DMSO.

4.2.5. Assay for antiproliferative effect

To explore the antiproliferative potential of compounds propidium iodide fluorescence assay was performed using different cell lines. To calculate the total nuclear DNA, a fluorescent dye (propidium iodide, PI) is used which can attach to the DNA, thus offering a quick and precise technique. PI cannot pass through the cell membrane, and its signal intensity can be considered as directly proportional to quantity of cellular DNA. Cells whose cell membranes are damaged or have changed permeability are counted as dead ones. The assay was performed by seeding the cells of different cell lines at a density of 3000-7500 cells/well (in200µl medium) in culture plates followed

by incubation for 24h at 37 °C in humidified 5%CO2/95% air atmospheric conditions. The medium was removed; the compounds were added to the plates at 10 µM concentrations (in 0.1% DMSO) in triplicates, followed by incubation for 48h. DMSO (0.1%) was used as control. After incubation, medium was removed followed by the addition of PI (25 µl, 50µg/mL in water/medium) to each well of the plates. At -80 °C, the plates were allowed to freeze for 24 h, followed by thawing at 25 °C. A fluorometer (Polar-Star BMG Tech) was used to record the readings at excitation and emission wavelengths of 530 and 620 nm for each well. The percentage cytotoxicity of compounds was calculated using the following formula:

$$\% \text{ Cytotoxicity} = \frac{A_C - A_{TC}}{A_C} \times 100$$

Where A*TC*= Absorbance of treated cells and AC= Absorbance of control.

Molecular docking

The examined enzymes were COX 2 (PDB ID 5KIR), human sEH (PDB ID: 3ANS), and 5-LOX (PDB ID code 6N2W) .The co-crystallized structures of the two enzymes bonded to the ligand inhibitors were downloaded from the Protein Data Bank, Chem Office tools (ChemDraw ultra 8.0 2D and 3D) were used to the energy-minimized chemical structures of the ligands; Open Babel GUI was used to convert the images of the ligands into their different file formats. The two enzymes were prepared by removing unnecessary co-crystallized ligands and adding polar hydrogen and charges using Autodock 4.2 and Discovery Studio 2024. For COX-2 enzyme, the depth of the XYZ input grid directions was fixed at a 84 × 84 × 84 Å with a spacing of 0.375 Å using an auto grid program, and the grid was placed in the center of the active site with xyz coordinates (20 × 20 × 20 Å^3) and was defined based on coordinates of the active

ligand provided for the target protein by the database. For human sEH enzyme, the depth of the XYZ input grid directions was fixed at a 60 × 60 × 60 Å with a spacing of 0.375 Å using an auto grid program, and the grid was placed in the center of the active site with xyz coordinates (22 × 0.467 × 53 $Å^3$) and was defined based on coordinates of the active ligand provided for the target protein by the database. In the case of 5-LOX, the depth of the XYZ input grid directions was fixed at a 48 × 44 × 72 Å with a spacing of 0.375 Å using an auto grid program, and the grid was placed in the center of the active site with xyz coordinates (35.9 × 65.3 × 38.3 $Å^3$) and was defined based on coordinates of the active ligand provided for the target protein by the database. Evaluation of the expected docked complexes was carried out on the basis of lowest binding energy complexes values. The 3D graphical depictions of the expected docked complexes was carried out using Discovery Studio Visualizer 2024 Software

Raw Data of MTT Assay

Conc. (µM)	HCT-116	MCF-7	MDAMB-231	PC-3
DOX				
100	7.1	6.2	6.3	8.8
50	13.9	10.9	11.2	16.3
25	18.7	14.3	14.1	21.7
12.5	31.4	26.9	28.3	38.9
6.25	47.9	41.5	45.8	59.2
3.125	60.5	58.4	57.6	73.6
1.56	73.8	69.1	71.2	95.3
SOR				
100	7.4	8.1	9.2	12.8
50	12.1	15.2	16.6	23.7
25	18.8	23.0	25.7	30.4
12.5	27.9	34.8	37.2	41.2
6.25	52.7	51.6	61.3	62.6
3.125	63.5	68.9	72.9	83.1
1.56	72.4	87.5	95.4	96.2
Celecoxib				
100	9.6	25.4	28.6	28.9
50	17.1	36.1	39.1	37.0
25	28.3	45.7	50.4	49.1
12.5	39.4	62.9	66.7	66.3
6.25	55.7	78.3	79.2	79.4
3.125	76.9	94.2	96.3	95.5
1.56	92.2	100	100	100
4m				
100	14.3	8.2	9.2	17.1
50	27.4	14.7	17.4	25.3
25	39.5	22.6	26.3	33.7
12.5	53.2	39.3	34.6	45.8
6.25	73.3	57.1	60.5	69.2
3.125	92.7	83.5	68.9	86.4
1.56	100	97.8	91.7	100
4g				
100	9.2	8.3	7.5	7.6
50	17.4	17.1	11.3	19.4
25	26.5	24.5	15.6	20.5

12.5	32.7	32.4	35.2	35.6
6.25	63.9	45.2	45.6	57.8
3.125	69.8	71.2	57.9	83.3
1.56	90.6	88.6	71.2	97.1

Conc.(µM)	HCT-116	MCF-7	MDAMB-231	PC-3
		4c		
100	7.8	7.2	4.5	14.3
50	15.2	11.9	10.2	23.7
25	22.7	20.3	17.6	31.6
12.5	24.5	29.7	26.7	48.2
6.25	51.6	45.1	37.3	61.1
3.125	66.5	61.6	47.1	87.4
1.56	89.1	73.4	61.2	99.3
		4j		
100	8.7	5.2	7.4	9.3
50	16.9	10.4	14.3	17.2
25	25.4	16.7	21.2	24.5
12.5	31.5	24.3	34.1	32.6
6.25	60.1	42.8	39.1	52.2
3.125	69.8	53.3	65.5	70.4
1.56	89.2	67.3	87.6	88.7
		4o		
100	22.4	8.1	13.9	18.7
50	29.7	20.4	16.9	27.9
25	41.9	22.6	25.8	36.4
12.5	50.2	39.2	31.6	52.3
6.25	73.6	57.3	60.4	69.6
3.125	91.5	84.1	69.3	86.5
1.56	100	97.5	89.2	100

Cox-2 activity assay

Compound KM-1 (4e)

IC_{50} = 4.248 µM

Concentration (µM)	Abs 1	Abs 2	Mean
0.05	0.6613	0.6628	0.6620
0.1	0.6481	0.6467	0.6474
0.2	0.6243	0.6226	0.6234
0.5	0.5792	0.5775	0.5783
1	0.5146	0.5159	0.5152
2	0.4398	0.4385	0.4392
5	0.3512	0.3497	0.3504
10	0.3198	0.3174	0.3186

$$100 \times \left(\frac{\text{sample}A_{\text{control}} - A}{\text{min}A_{\text{control}} - A} \right) \text{=\% of Inhibition}$$

Concentration (µM)	(Abs)	% Inhibition
0.05	0.6620	4.8673
0.1	0.6474	8.5223
0.2	0.6234	15.0956
0.5	0 .5783	26.8086
1	0.5152	43.2203
2	0.4392	58.2105
5	0.3504	67.1052
10	0.3186	67.3971

Log_{10}(conc)	% Inhibition
-1.3010	4.8673
-1.0000	8.5223
-0.6989	15.0956
-0.3010	26.8086
0.0000	43.2203
0.3010	58.2105
0.6989	67.1052
1.0000	67.3971

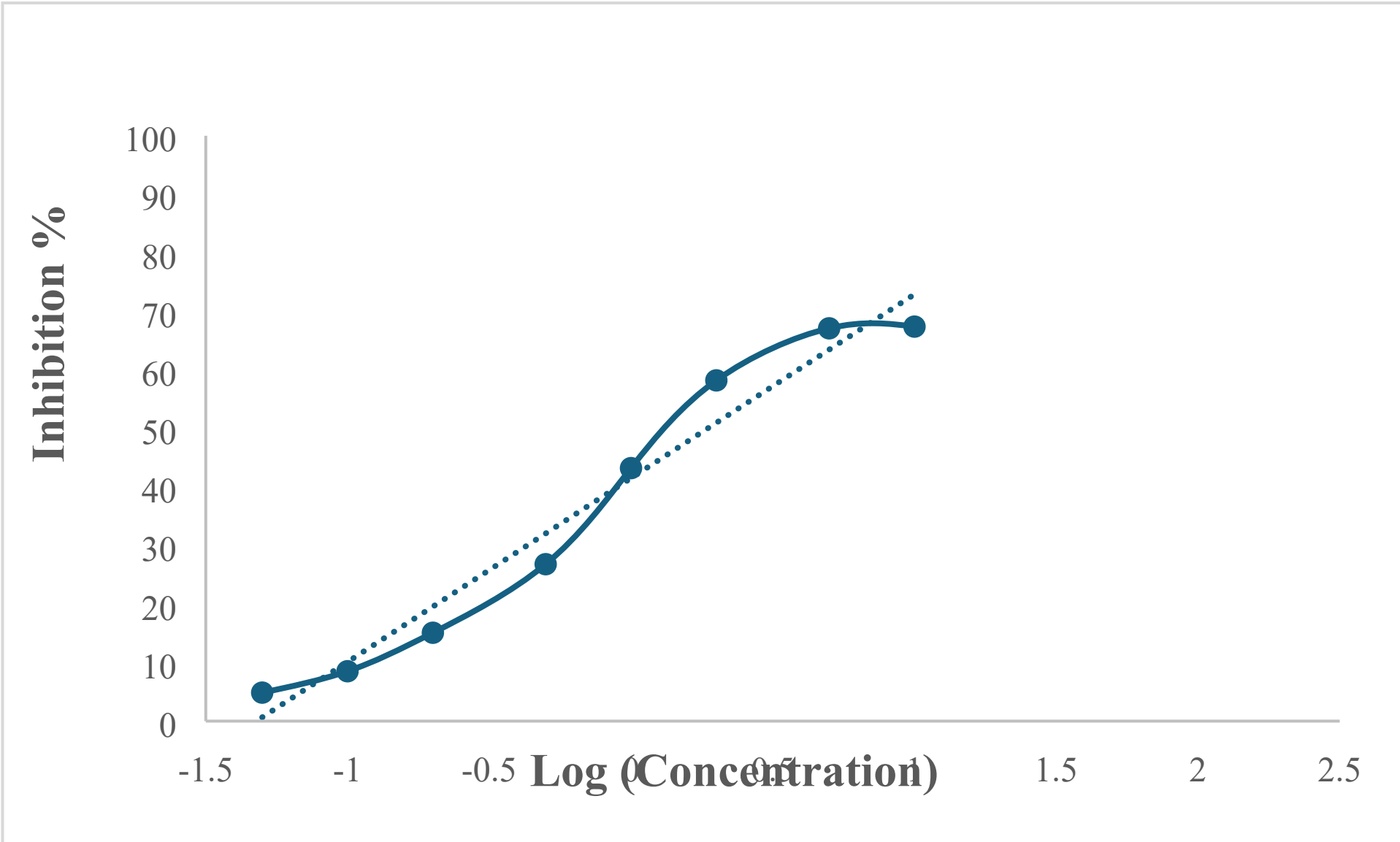

Compound KM-2 (4l)

IC_{50} = 2.425 µM

**Concentration (µM)**	**Abs 1**	**Abs 2**	**Mean**
0.05	0.6715	0.6732	0.6724
0.1	0.6591	0.6603	0.6597
0.2	0.6416	0.6398	0.6407
0.5	0.5912	0.5894	0.5903
1	0.5275	0.5283	0.5279
2	0.4336	0.4318	0.4327
5	0.3012	0.3027	0.3019
10	0.2863	0.2851	0.2857

$$100 \times \left(\frac{\text{sample}A_{\text{control}} - A}{\text{min}A_{\text{control}} - A} \right)$$ =% of Inhibition

**Concentration (µM)**	**(Abs)**	**% Inhibition**
0.05	0.6724	6.8732
0.1	0.6597	9.9296
0.2	0.6407	15.9922
0.5	0.5903	29.7801
1	0.5279	43.4744
2	0.4327	60.7941
5	0.3019	78.7038
10	0.2857	79.5923

**Log_{10}(conc)**	**% Inhibition**

Log$_{10}$(conc)	% Inhibition
-1.3010	6.8732
-1.0000	9.9296
-0.6989	15.9922
-0.3010	29.7801
0.0000	43.4744
0.3010	60.7941
0.6989	78.7038
1.0000	79.5923

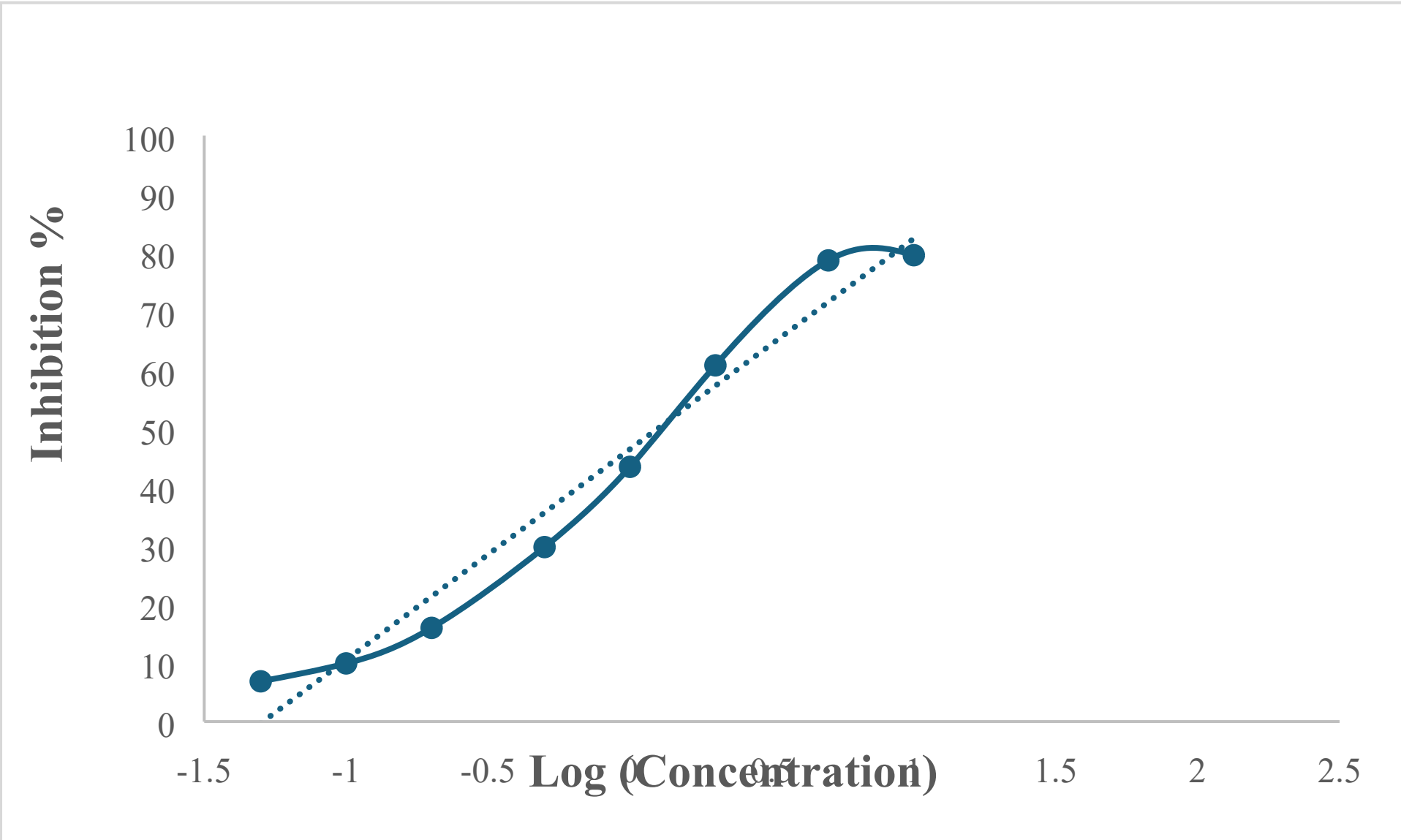

Compound KM-3 (4g)

IC$_{50}$ = 0.896 µM

Concentration (µM)	Abs 1	Abs 2	Mean
0.05	0.6657	0.6673	0.6665
0.1	0.6416	0.6439	0.6428
0.2	0.5984	0.5962	0.5973
0.5	0.5236	0.5211	0.5224
1	0.4192	0.4177	0.4184
2	0.3146	0.3163	0.3154
5	0.1927	0.1911	0.1919
10	0.1385	0.1362	0.1373

$$100 \times \left(\frac{{}_{\text{sample}}A_{\text{control}} - A}{{}_{\text{min}}A_{\text{control}} - A} \right)$$ =% of Inhibition

Concentration (µM)	(Abs)	% Inhibition
0.05	0.6665	10.8532

Concentration (µM)	(Abs)	% Inhibition
0.1	0.6428	16.1851
0.2	0.5973	24.1401
0.5	0.5224	28.2935
1	0.4184	47.3931
2	0.3154	66.4727
5	0.1919	88.2093
10	0.1373	89.7421

Log_{10}(conc)	% Inhibition
-1.3010	10.8532
-1.0000	16.1851
-0.6989	24.1401
-0.3010	28.2935
0.0000	47.3931
0.3010	66.4727
0.6989	88.2093
1.0000	89.7421

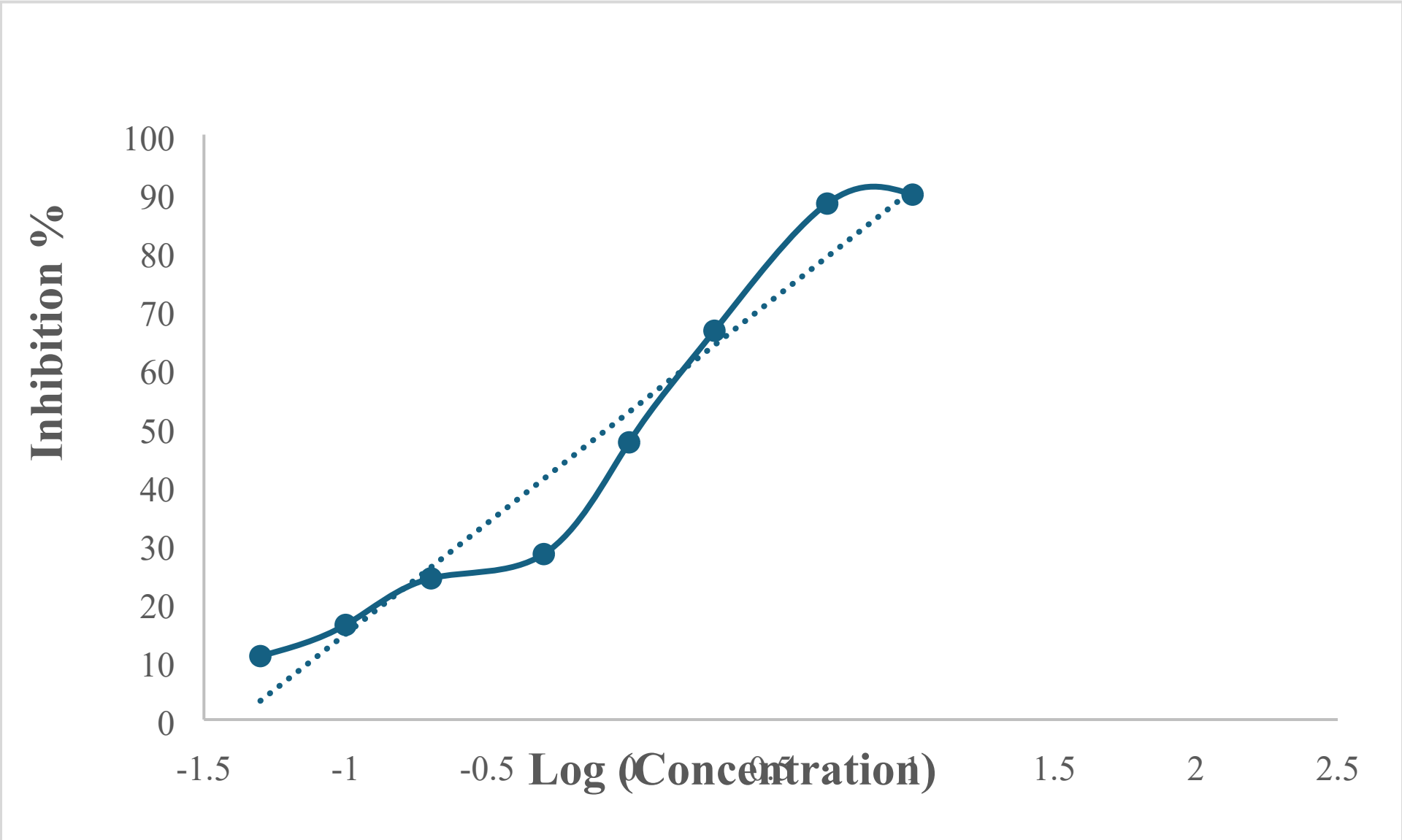

Compound KM-4 (4k)

IC_{50} = 2.752 µM

Concentration (µM)	Abs 1	Abs 2	Mean
0.05	0.6832	0.6816	0.6824

Concentration (µM)	Abs 1	Abs 2	Mean
0.1	0.6623	0.6641	0.6632
0.2	0.6318	0.6346	0.6332
0.5	0.5821	0.5798	0.5810
1	0.5174	0.5136	0.5155
2	0.4223	0.4192	0.4207
5	0.2742	0.2765	0.2753
10	0.1695	0.1717	0.1706

$100 \times \left(\frac{\text{sample}A_{\text{control}} - A}{\text{min}A_{\text{control}} - A} \right)$ =% of Inhibition

Concentration (µM)	(Abs)	% Inhibition
0.05	0.6824	6.0437
0.1	0.6632	10.3458
0.2	0.6332	17.5096
0.5	0.5810	26.7326
1	0.5155	33.5481
2	0.4207	51.1346
5	0.2753	77.2971
10	0.1706	77.1385

Log_{10}(conc)	% Inhibition
-1.3010	6.0437
-1.0000	10.3458
-0.6989	17.5096
-0.3010	26.7326
0.0000	33.5481
0.3010	51.1346
0.6989	77.2971
1.0000	77.4385

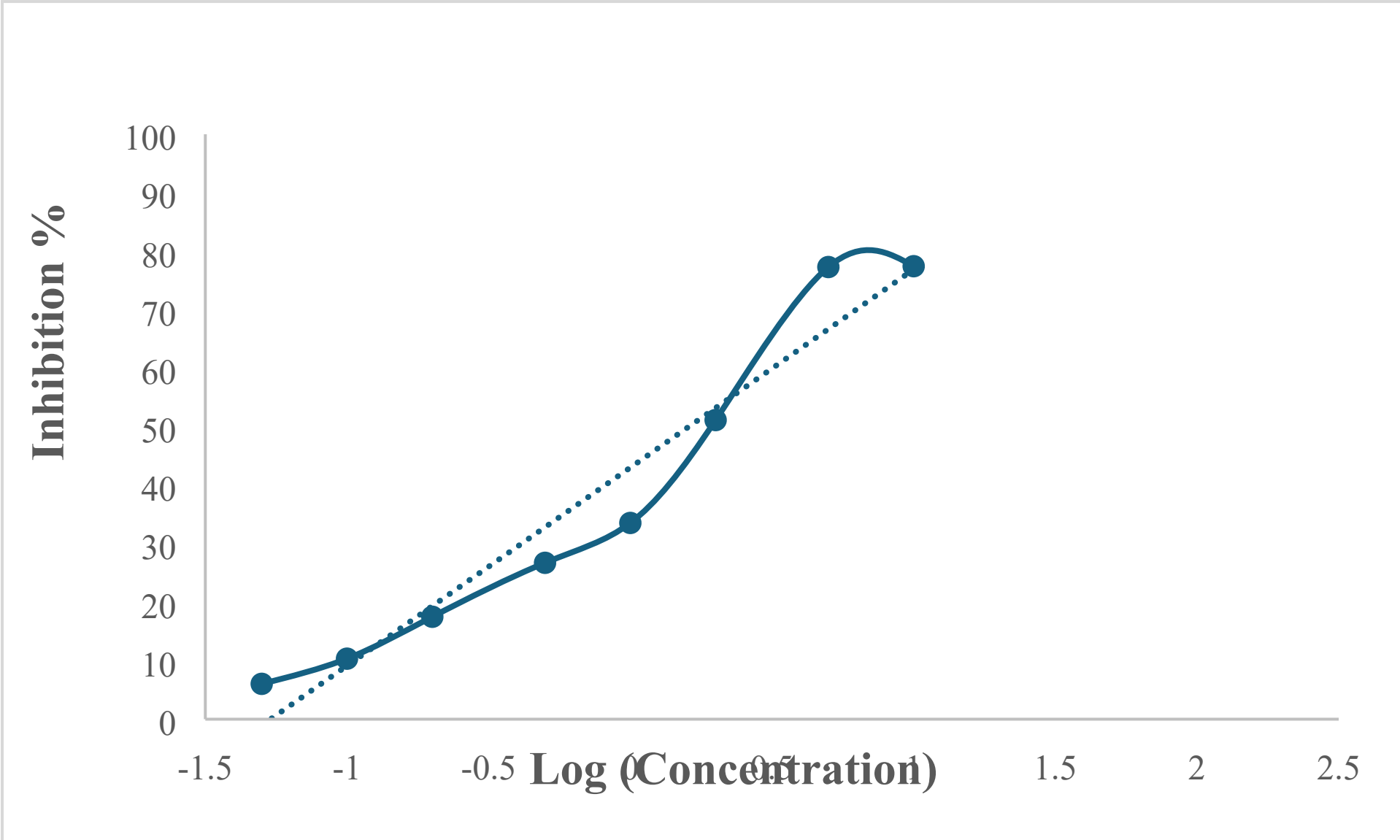

Compound KM-5 (4n)
IC_{50} = 2.139 **µM**

**Concentration (µM)**	**Abs 1**	**Abs 2**	**Mean**
0.05	0.6284	0.6257	0.6270
0.1	0.5983	0.6012	0.5997
0.2	0.5629	0.5606	0.5617
0.5	0.4951	0.4926	0.4938
1	0.4212	0.4187	0.4199
2	0.3585	0.3551	0.3568
5	0.2216	0.2238	0.2227
10	0.1361	0.1383	0.1372

$100 \times \left(\frac{{}_{\text{sample}}A_{\text{control}} - A}{{}_{\text{min}}A_{\text{control}} - A}\right)$ =% of Inhibition

**Concentration (µM)**	**(Abs)**	**% Inhibition**
0.05	0.6270	7.3228
0.1	0.5997	12.0586
0.2	0.5617	18.6590
0.5	0.4938	27.2336
1	0.4199	42.0292
2	0.3568	54.6418
5	0.2227	81.0782
10	0.1372	82.4118

**Log_{10}(conc)**	**% Inhibition**
-1.3010	7.3228
-1.0000	12.0586

Log$_{10}$(conc)	% Inhibition
-0.6989	18.6590
-0.3010	27.2336
0.0000	42.0292
0.3010	54.6418
0.6989	81.0782
1.0000	86.4118

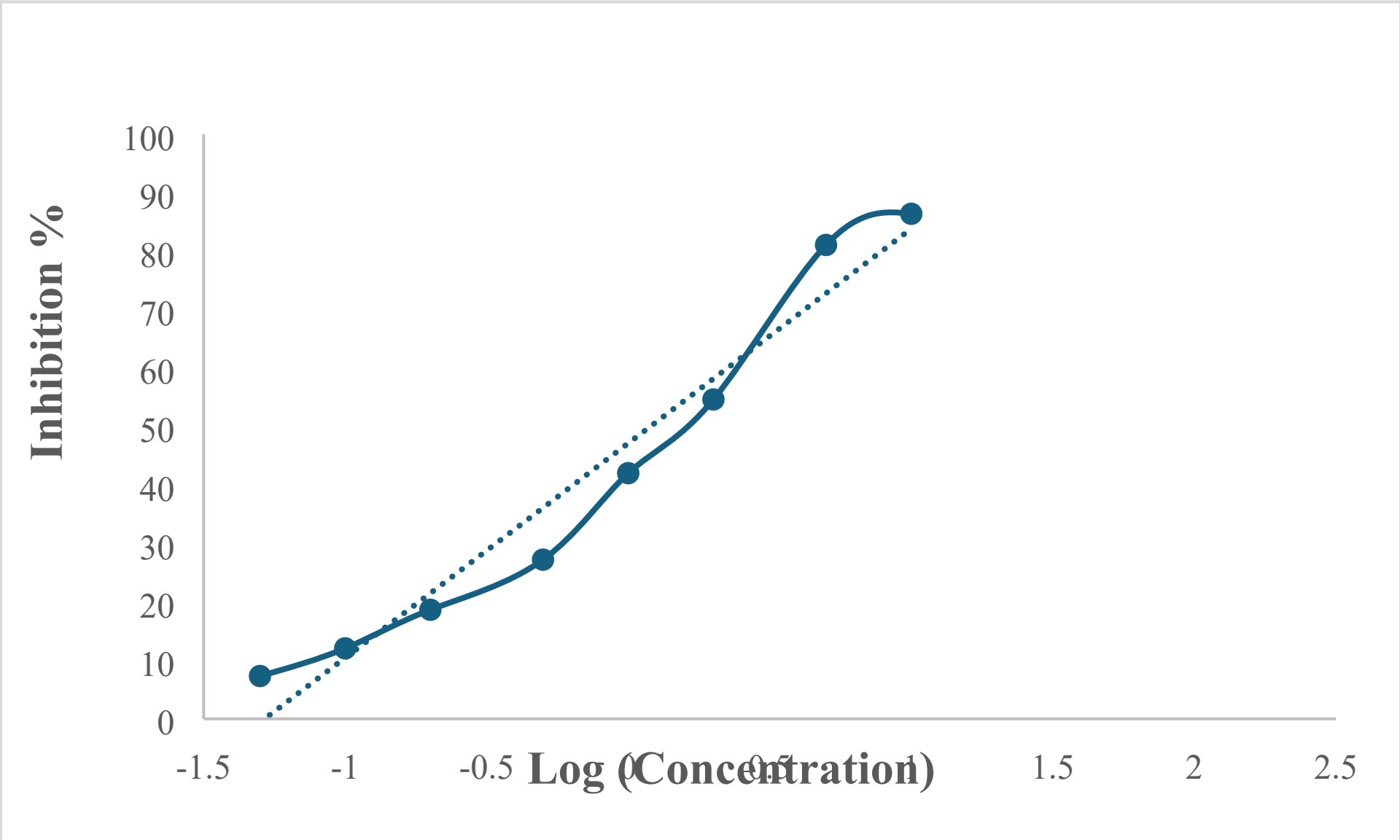

Compound KM-6 (4j)

IC$_{50}$ = 0.274 µM

Concentration (µM)	Abs 1	Abs 2	Mean
0.05	0.5873	0.5847	0.5860
0.1	0.5481	0.5456	0.5468
0.2	0.4965	0.4943	0.4954
0.5	0.3826	0.3794	0.3810
1	0.2671	0.2655	0.2663
2	0.1824	0.1851	0.1838
5	0.1152	0.1178	0.1165
10	0.0843	0.0817	0.0830

$$100 \times \left(\frac{{}_{\mathrm{sample}}A_{\mathrm{control}} - A}{{}_{\mathrm{min}}A_{\mathrm{control}} - A} \right)$$ =% of Inhibition

Concentration (µM)	(Abs)	% Inhibition
0.05	0.5860	11.3889
0.1	0.5468	16.1441
0.2	0.4954	24.7716
0.5	0.3810	44.4707

Concentration (μM)	(Abs)	% Inhibition
1	0.2663	65.5549
2	0.1838	79.4724
5	0.1165	89.2870
10	0.0830	91.4883

Log_{10}(conc)	% Inhibition
-1.3010	11.3889
-1.0000	16.1441
-0.6989	24.7716
-0.3010	44.4707
0.0000	65.5549
0.3010	79.4724
0.6989	89.2870
1.0000	91.4883

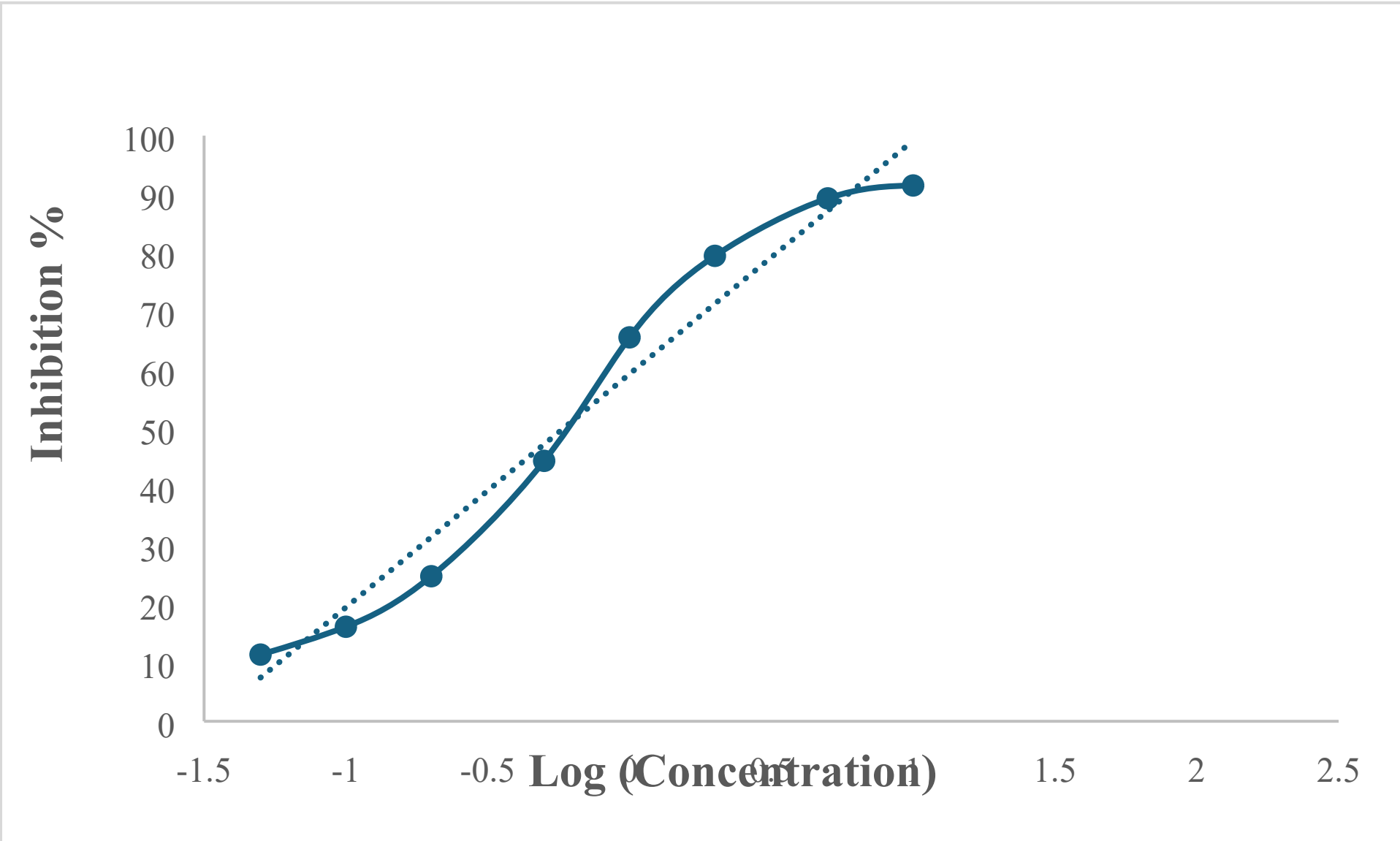

Compound KM-7 (4m)

IC_{50} = 1.091 μM

Concentration (μM)	Abs 1	Abs 2	Mean
0.05	0.5882	0.5867	0.5874
0.1	0.5439	0.5415	0.5427
0.2	0.4841	0.4826	0.4833

Concentration (µM)	Abs 1	Abs 2	Mean
0.5	0.3682	0.3659	0.3670
1	0.2943	0.2961	0.2952
2	0.2084	0.2067	0.2075
5	0.1359	0.1344	0.1352
10	0.0901	0.0873	0.0887

$$100 \times \left(\frac{{}_{\text{sample}}A_{\text{control}} - A}{{}_{\text{min}}A_{\text{control}} - A}\right)$$ =% of Inhibition

Concentration (µM)	(Abs)	% Inhibition
0.05	0.5874	10.1659
0.1	0.5427	16.1465
0.2	0.4833	25.5518
0.5	0.3670	43.9189
1	0.2952	56.8173
2	0.2075	71.3292
5	0.1352	82.6784
10	0.0887	86.2055

Log_{10}(conc)	% Inhibition
-1.3010	10.1659
-1.0000	16.1465
-0.6989	25.5518
-0.3010	43.9189
0.0000	56.8173
0.3010	71.3292
0.6989	82.6784
1.0000	87.2055

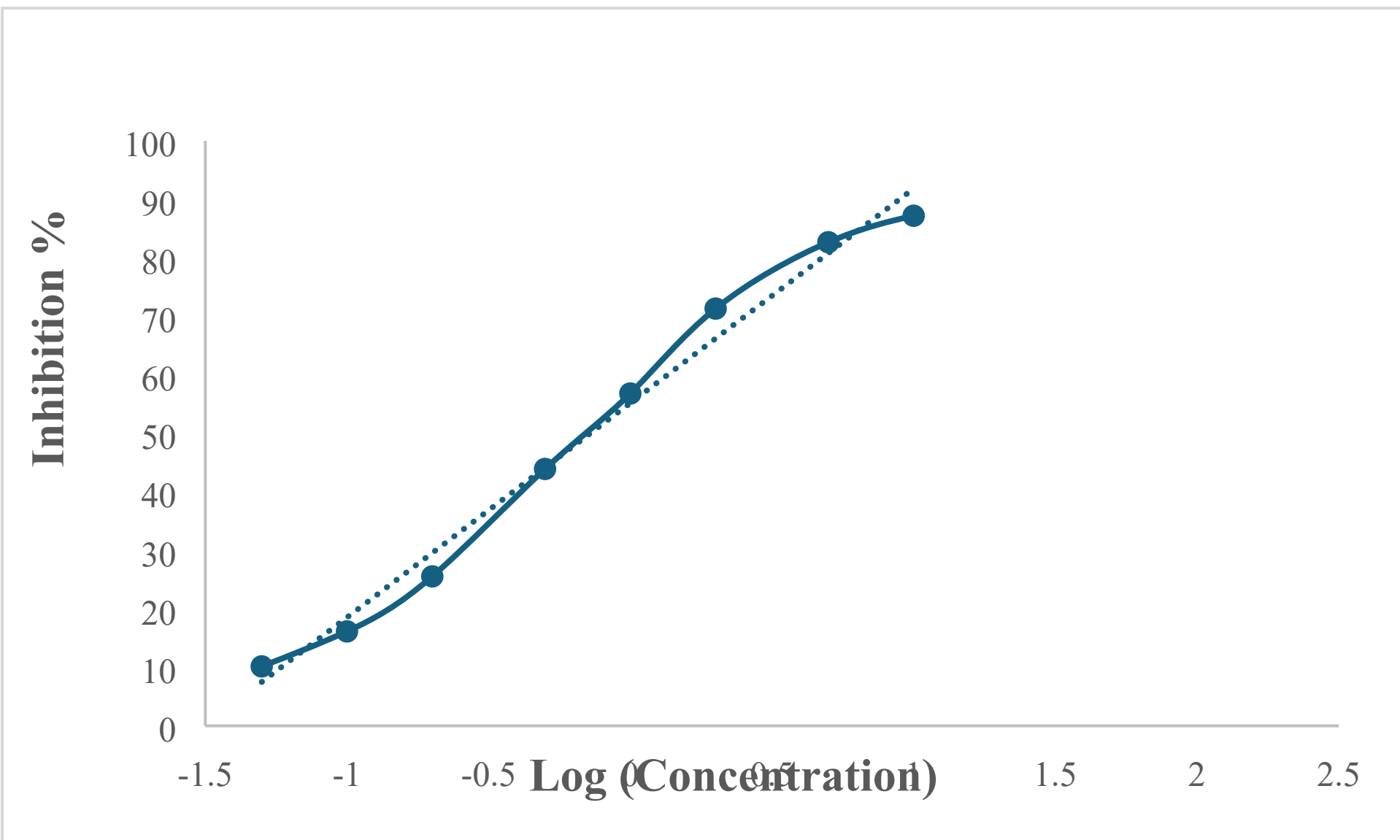

Compound KM-8 (4i)

IC_{50} = 5.603 µM

Concentration (µM)	Abs 1	Abs 2	Mean
0.05	0.6112	0.6095	0.6104
0.1	0.5821	0.5842	0.5832
0.2	0.5389	0.5371	0.5380
0.5	0.4894	0.4877	0.4885
1	0.4382	0.4369	0.4376
2	0.3815	0.3801	0.3808
5	0.3343	0.3359	0.3351
10	0.3125	0.3138	0.3131

$100 \times \left(\frac{{}_{sample}A_{control} - A}{{}_{min}A_{control} - A} \right)$ =% of Inhibition

Concentration (µM)	(Abs)	% Inhibition
0.05	0.6104	4.1077
0.1	0.5832	9.1245
0.2	0.5380	17.6801
0.5	0.4885	27.3566
1	0.4376	38.1334
2	0.3808	49.9864
5	0.3351	59.1452
10	0.3131	62.3119

Log_{10}(conc)	% Inhibition
-1.3010	4.1077
-1.0000	9.1245
-0.6989	17.6801
-0.3010	27.3566
0.0000	38.1334
0.3010	49.9864
0.6989	59.1452
1.0000	62.3119

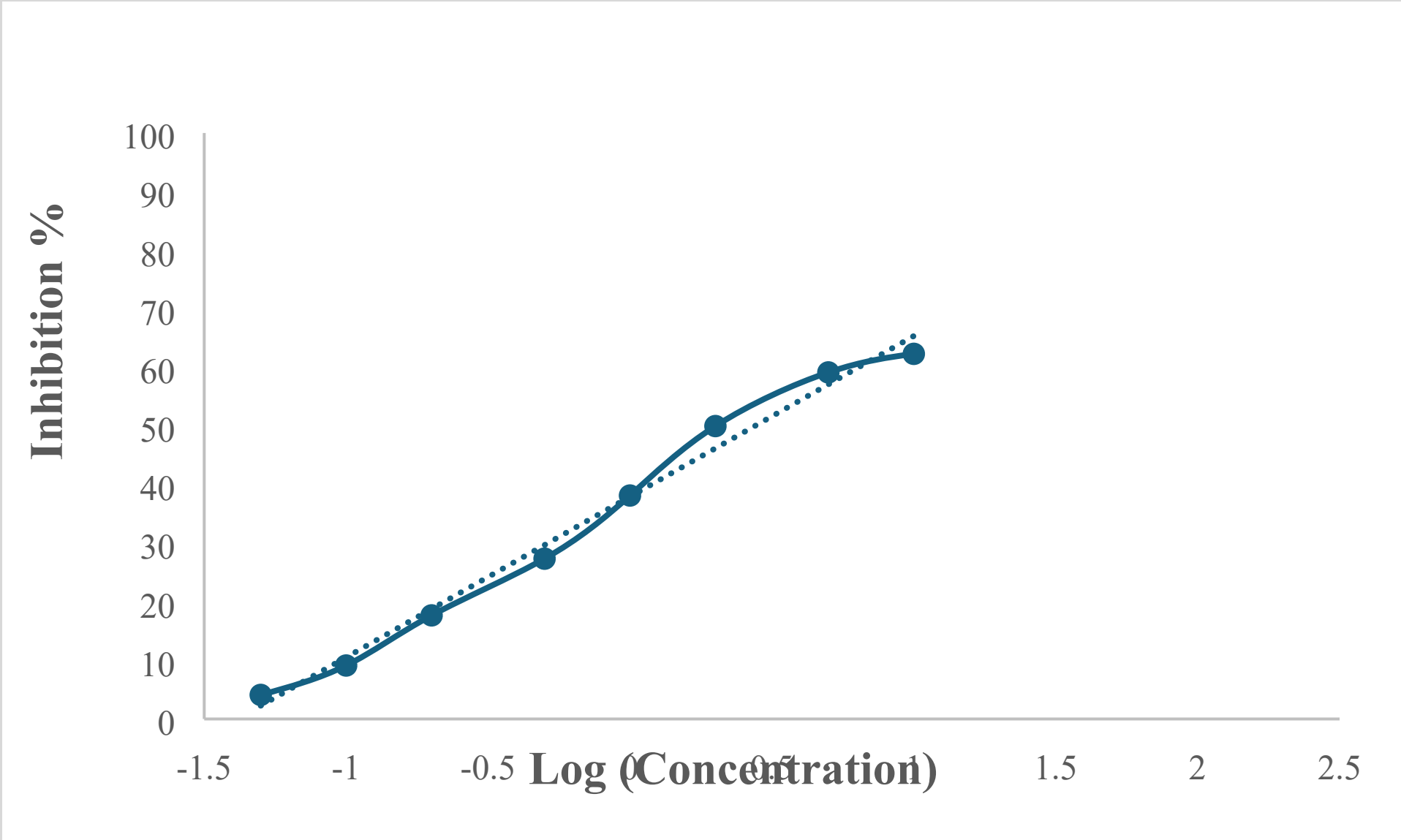

Compound KM-9 (4d)

IC_{50} = 16.467 µM

**Concentration (µM)**	**Abs 1**	**Abs 2**	**Mean**
0.05	0.6023	0.6041	0.6032
0.1	0.5917	0.5898	0.5908
0.2	0.5689	0.5665	0.5677
0.5	0.5246	0.5259	0.5253
1	0.4732	0.4758	0.4745
2	0.4215	0.4231	0.4223
5	0.3698	0.3681	0.3689
10	0.3457	0.3441	0.3449

$$100 \times \left(\frac{\text{sample} A_{\text{control}} - A}{\text{min} A_{\text{control}} - A} \right)$$ =% of Inhibition

**Concentration (µM)**	**Abs**	**% Inhibition**
0.05	0.6032	5.90332
0.1	0.5908	9.45873
0.2	0.5677	13.6954
0.5	0.5253	23.8547
1	0.4745	38.5071
2	0.4223	52.9984
5	0.3689	69.2433
10	0.3449	71.2889

**Log_{10}(conc)**	**% Inhibition**
-1.3010	5.9033

Log$_{10}$(conc)	% Inhibition
-1.0000	9.4587
-0.6989	13.6954
-0.3010	23.8547
0.0000	38.5071
0.3010	52.9984
0.6989	69.2433
1.0000	71.2889

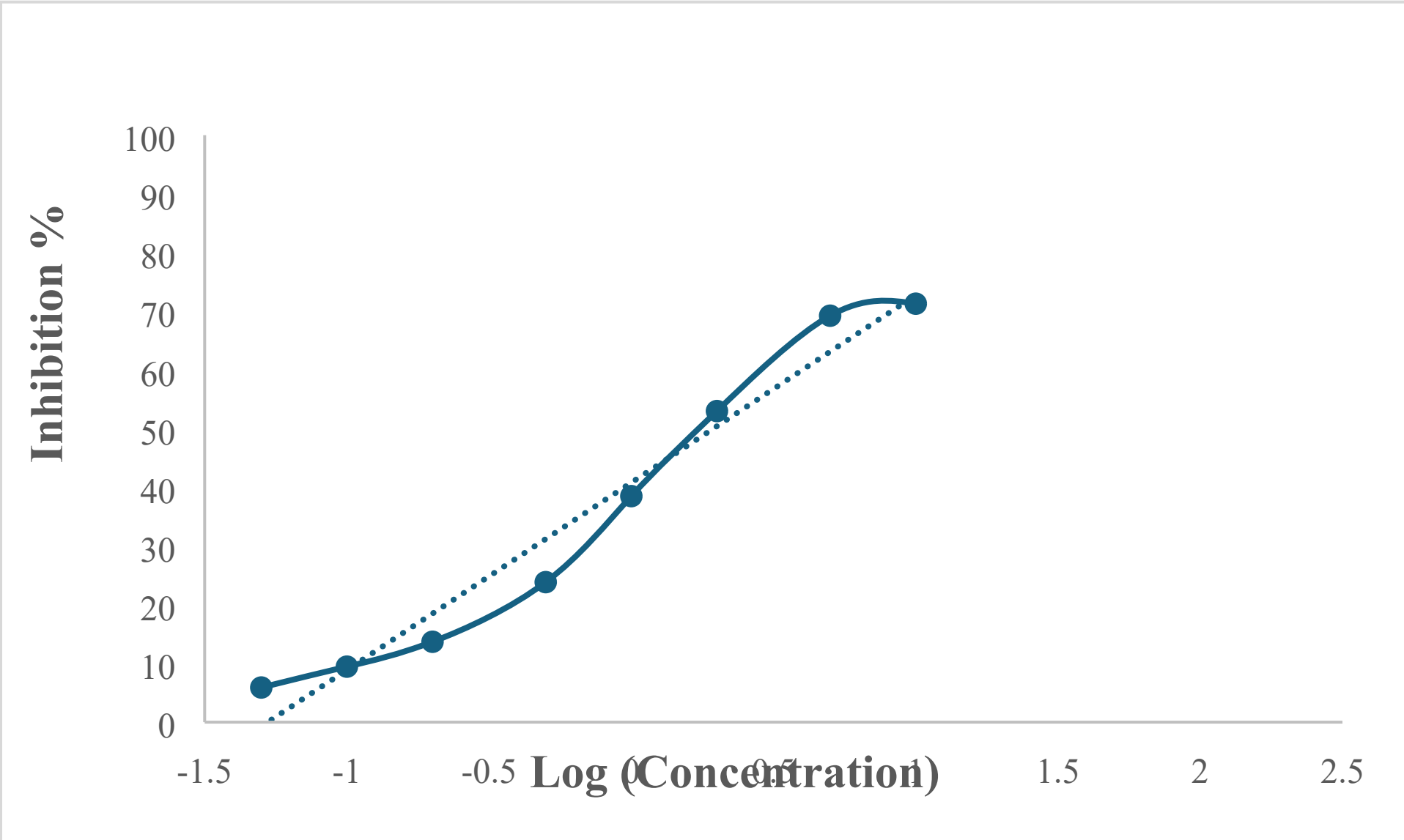

Compound KM-10 (4h)

IC$_{50}$ = 5.918 µM

Concentration (µM)	Abs 1	Abs 2	Mean
0.05	0.5923	0.5936	0.5930
0.1	0.5874	0.5891	0.5882
0.2	0.5788	0.5799	0.5793
0.5	0.5605	0.5612	0.5608
1	0.5311	0.5298	0.5305
2	0.4979	0.4986	0.4982
5	0.4581	0.4572	0.4576
10	0.4416	0.4425	0.4420

$100 \times \left(\frac{\text{sample}A\,\text{control} - A}{\text{min}A\,\text{control} - A} \right)$ =% of Inhibition

Concentration (µM)	Abs	% Inhibition
0.05	0.5930	2.7644
0.1	0.5882	3.1823
0.2	0.5793	4.5308
0.5	0.5608	8.5462
1	0.5305	13.9142
2	0.4982	20.5718
5	0.4576	35.9842
10	0.4420	58.2967

Log_{10}(conc)	% Inhibition
-1.3010	2.7644
-1.0000	3.1823
-0.6989	4.5308
-0.3010	8.5462
0.0000	13.9142
0.3010	20.5718
0.6989	35.9842
1.0000	58.2967

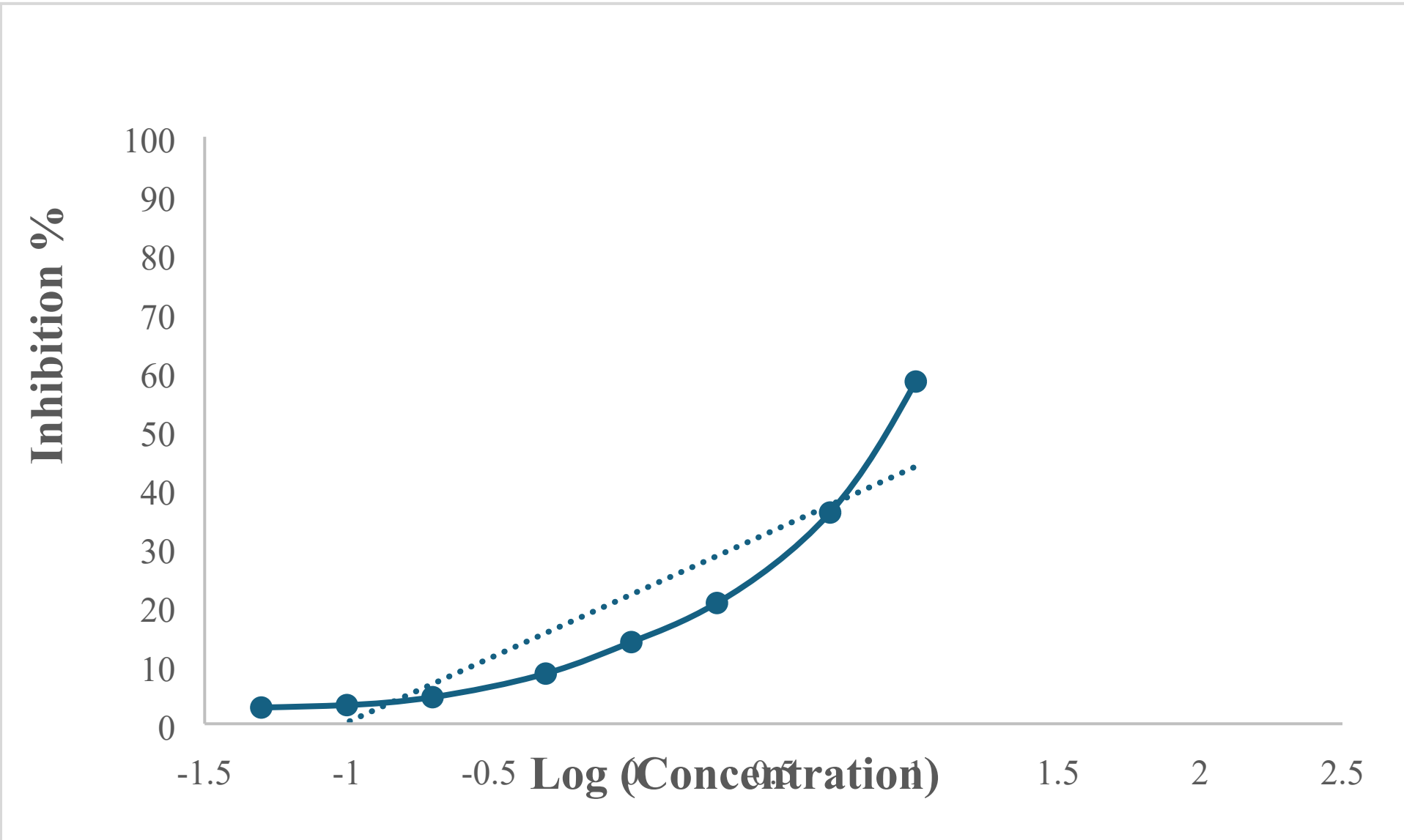

Compound KM-11 (4o)

IC_{50} = 1.375 µM

Concentration (µM)	Abs 1	Abs 2	Mean
0.05	0.7512	0.7520	0.7516
0.1	0.7358	0.7371	0.7365
0.2	0.7106	0.7099	0.7103
0.5	0.6531	0.6525	0.6528
1	0.5987	0.5993	0.5990

Concentration (µM)	Abs 1	Abs 2	Mean
2	0.4934	0.4941	0.4938
5	0.3791	0.3787	0.3789
10	0.2783	0.2787	0.2785

$100 \times \left(\frac{\text{sample}A_{\text{control}} - A}{\text{min}A_{\text{control}} - A}\right)$ =% of Inhibition

Concentration (µM)	Abs	% Inhibition
0.05	0.7516	8.3262
0.1	0.7365	11.2879
0.2	0.7103	17.4487
0.5	0.6528	29.0132
1	0.5990	38.1051
2	0.4938	58.6295
5	0.3789	79.4417
10	0.2785	85.1194

Log_{10} (Concentration)	% Inhibition
-1.3010	8.3262
-1.0000	11.2879
-0.6989	17.4487
-0.3010	29.0132
0.0000	38.1051
0.3010	58.6295
0.6989	79.4417
1.0000	85.1194

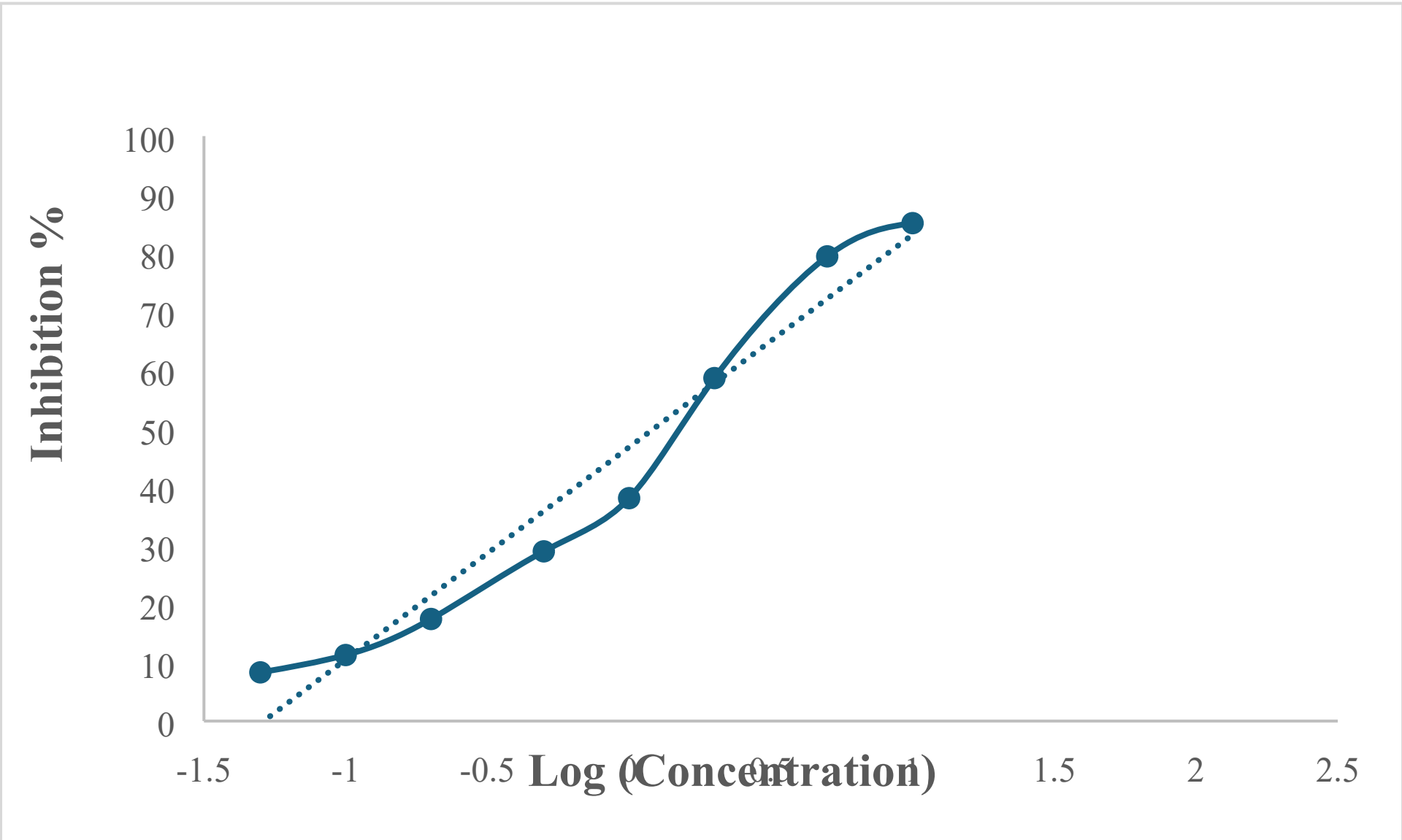

Compound KM-12 (4c)

IC_{50} = 0.108 µM

**Concentration (µM)**	**Abs 1**	**Abs 2**	**Mean**
0.05	0.8451	0.8462	0.8457
0.1	0.8324	0.8336	0.8330
0.2	0.7995	0.8008	0.8002
0.5	0.7403	0.7396	0.7399
1	0.6584	0.6578	0.6581
2	0.5207	0.5213	0.5210
5	0.3763	0.3757	0.3760
10	0.2702	0.2708	0.2705

$$100 \times \left(\frac{\text{sample}A_{\text{control}} - A}{\text{min}A_{\text{control}} - A} \right)$$ =% of Inhibition

**Concentration (µM)**	**Abs**	**% Inhibition**
0.05	0.8457	12.5322
0.1	0.8330	15.2198
0.2	0.8002	22.3137
0.5	0.7399	34.8629
1	0.6581	47.2785
2	0.5210	68.1581
5	0.3760	85.4038
10	0.2705	94.7308

**Log_{10} (Concentration)**	**% Inhibition**
-1.3010	12.5322
-1.0000	15.2198
-0.6989	22.3137
-0.3010	34.8629
0.0000	47.2785
0.3010	68.1581
0.6989	85.4038
1.0000	94.7308

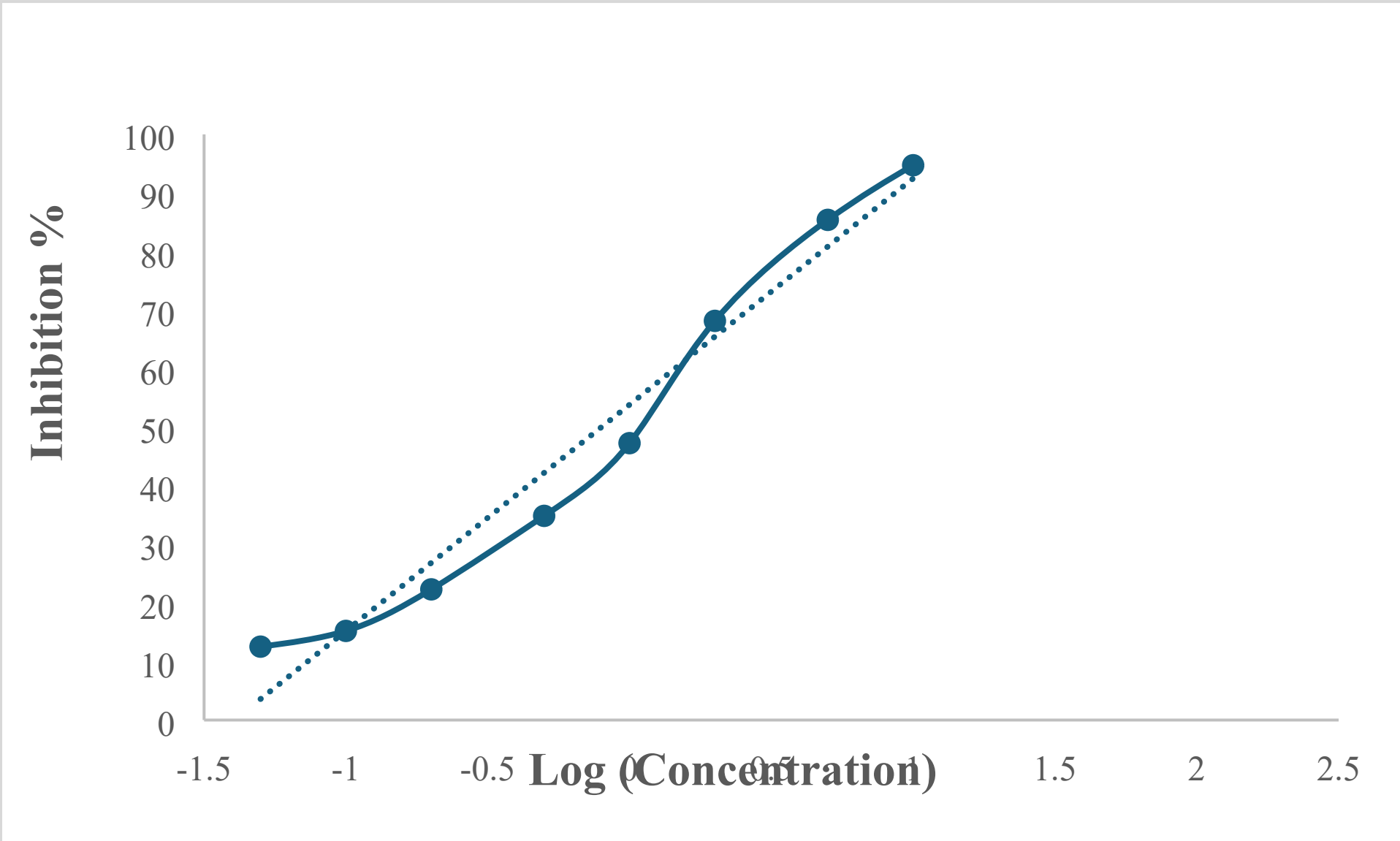

Compound Celecoxib

IC_{50} = 0.079 µM

Concentration (µM)	Abs 1	Abs 2	Mean
0.05	0.7812	0.7824	0.7818
0.1	0.7561	0.7557	0.7559
0.2	0.6934	0.6941	0.6937
0.5	0.5727	0.5736	0.5732
1	0.4133	0.4128	0.4131
2	0.2759	0.2767	0.2763
5	0.1237	0.1246	0.1242
10	0.0645	0.0648	0.0647

$100 \times \left(\frac{{}_{sample}A_{control} - A}{{}_{min}A_{control} - A}\right)$ =% of Inhibition

Concentration (µM)	ABS	% Inhibition
0.05	0.7818	16.2884
0.1	0.7559	18.4522
0.2	0.6937	29.6550
0.5	0.5732	46.6033
1	0.4131	72.3969
2	0.2763	85.5791
5	0.1242	92.1148
10	0.0647	97.4173

Log_{10} (Concentration)	% Inhibition
-1.3010	16.2884
-1.0000	18.4522
-0.6989	29.6550
-0.3010	46.6033
0.0000	72.3969
0.3010	85.5791
0.6989	92.1148
1.0000	97.4173

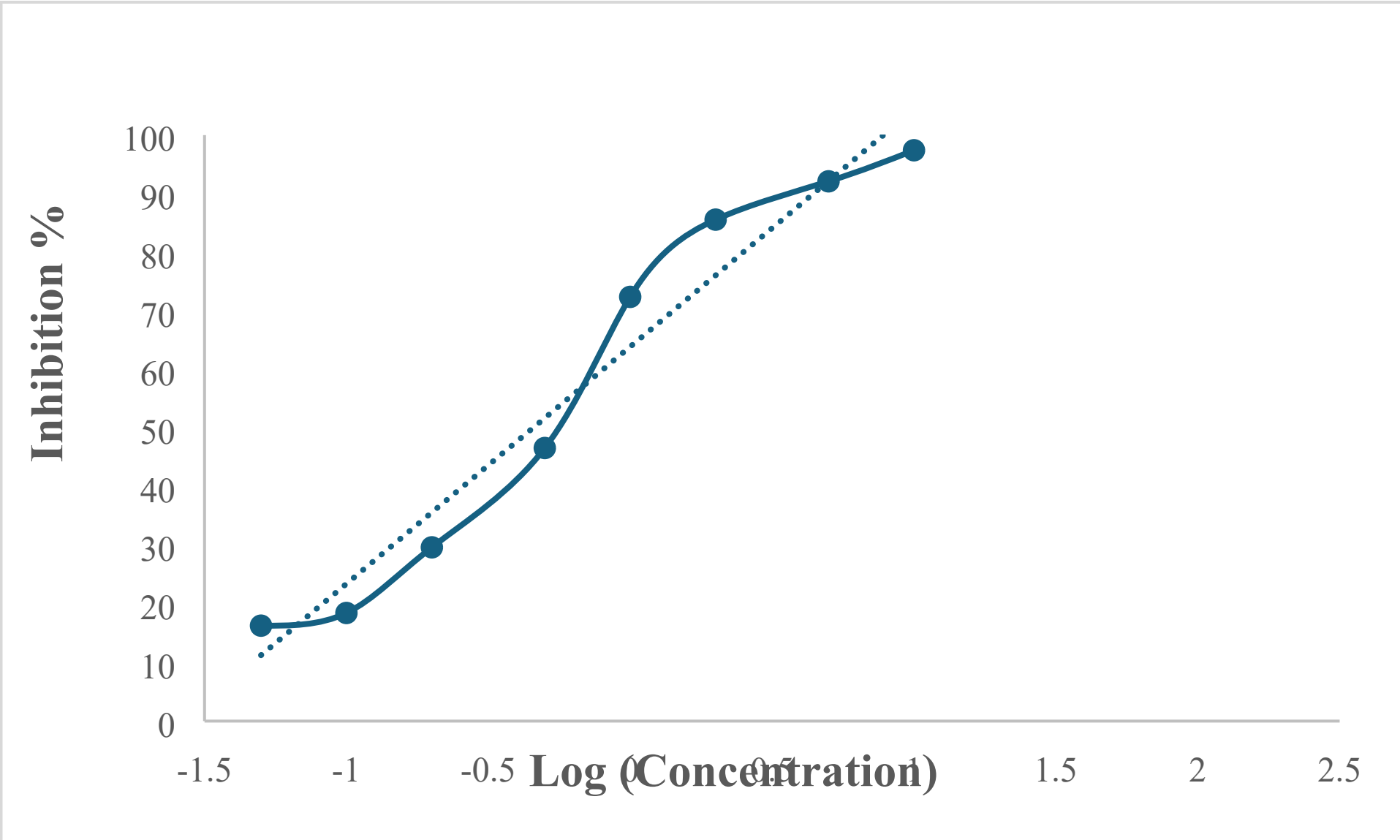

Cox-1 activity assay

Compound KM-1 (4e)

IC_{50} = 0.836 µM

Concentration (µM)	1 (Abs)	2 (Abs)	Mean (Abs)
0.1	0.6124	0.6142	0.6133
0.2	0.5847	0.5826	0.5837
0.5	0.5192	0.5209	0.5200
1	0.4476	0.4458	0.4467
2	0.3643	0.3662	0.3652
5	0.2395	0.2381	0.2388
10	0.1826	0.1814	0.1820
20	0.1341	0.1326	0.1333

$$100 \times \left(\frac{\text{sample}A_{\text{control}} - A}{\text{min}A_{\text{control}} - A} \right) \text{=\% of Inhibition}$$

Concentration (µM)	(Abs)	% Inhibition
0.1	0.6124	13.817
0.2	0.5847	18.361
0.5	0.5192	28.074
1	0.4476	40.824
2	0.3643	53.783
5	0.2395	72.964
10	0.1826	85.639
20	0.1341	98.482

Log_{10} (conc)	% Inhibition
-1.0000	13.817
-0.6989	18.361
-0.3010	28.074
0.0000	40.824
0.3010	53.783
0.6989	72.964
1.0000	85.639

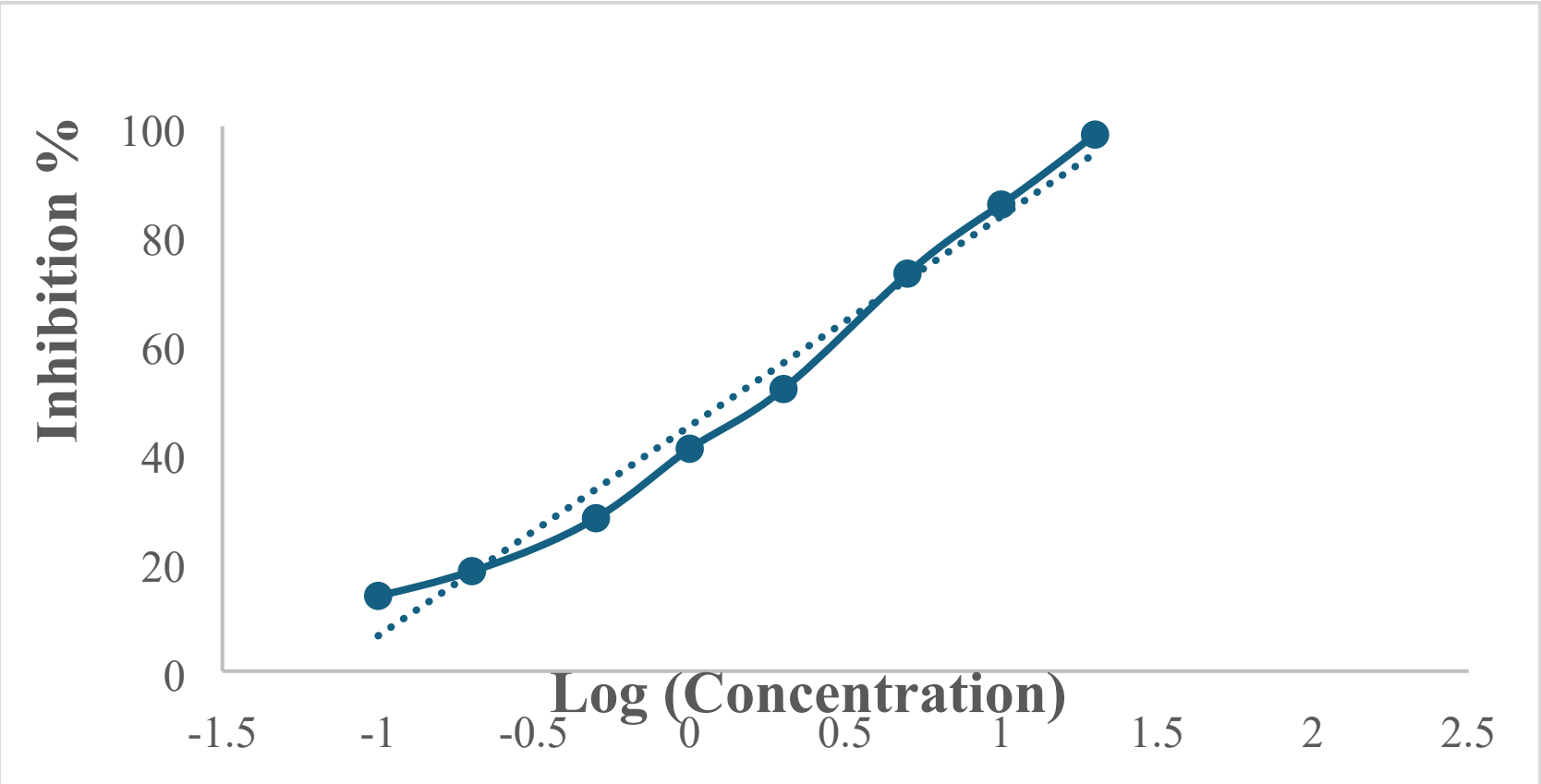

Log10 (conc)	% Inhibition
1.3010	98.482

Compound KM-2 (4l)

IC_{50} = 3.742 µM

Concentration (µM)	1 (Abs)	2 (Abs)	Mean (Abs)
0.1	0.6183	0.6169	0.6176
0.2	0.5836	0.5821	0.5828
0.5	0.5127	0.5142	0.5134
1	0.4375	0.4361	0.4368
2	0.3642	0.3628	0.3635
5	0.2481	0.2493	0.2487
10	0.1927	0.1941	0.1934
20	0.1462	0.1478	0.1470

$$100 \times \left(\frac{{}_{\text{sample}}A_{\text{control}} - A}{{}_{\text{min}}A_{\text{control}} - A} \right)$$ =% of Inhibition

Concentration (µM)	(Abs)	% Inhibition
0.1	0.6183	9.3281
0.2	0.5836	15.928
0.5	0.5127	28.507
1	0.4375	41.794
2	0.3642	54.706
5	0.2481	74.536
10	0.1927	83.752
20	0.1462	91.078

Log10(conc)	% Inhibition
-1.0000	9.3281
-0.6989	15.928
-0.3010	28.507
0.0000	41.794
0.3010	54.706
0.6989	74.536
1.0000	83.752
1.3010	91.078

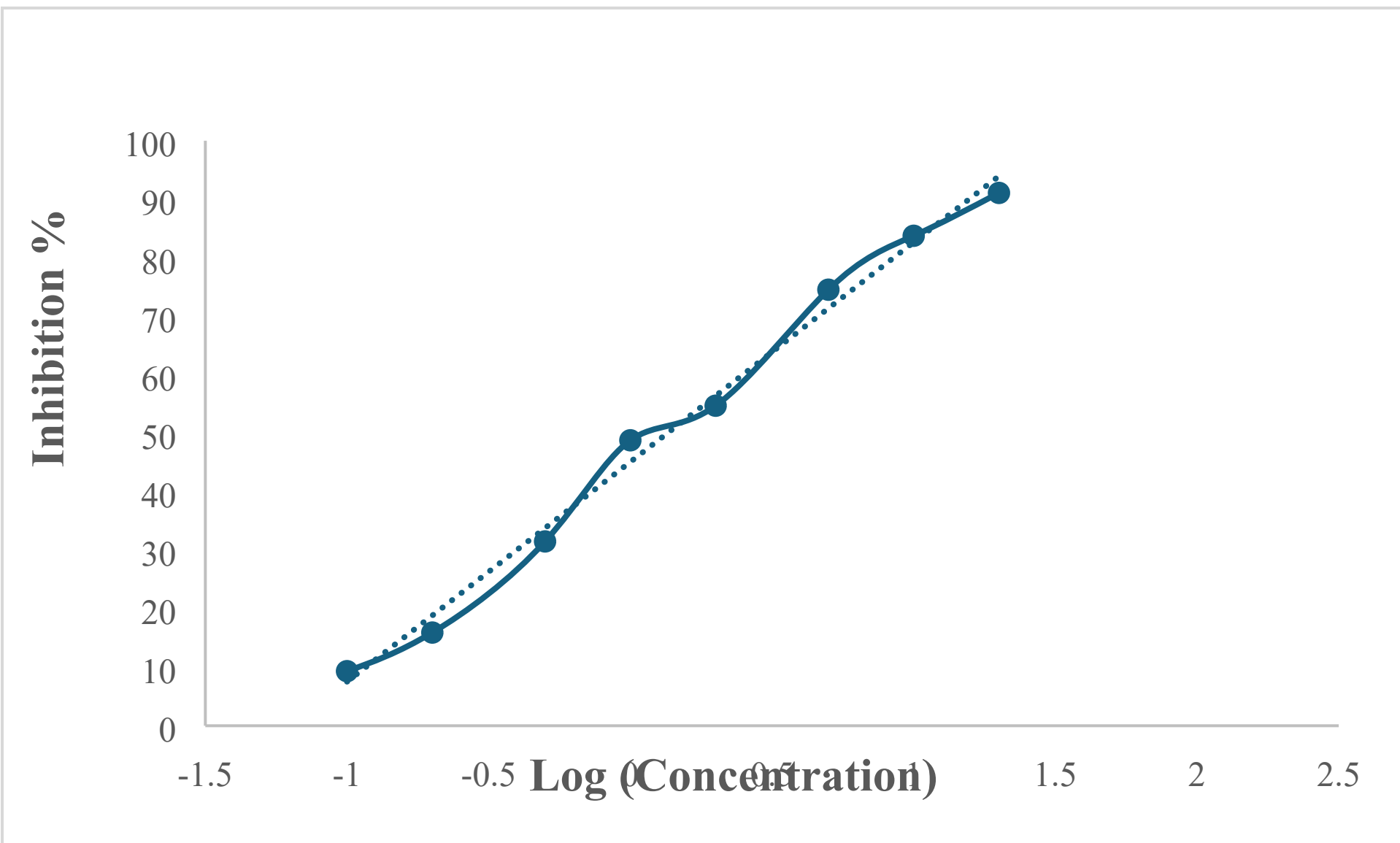

Compound KM-3 (4g)
IC_{50} = 1.763 µM

Concentration (µM)	1 (Abs)	2 (Abs)	Mean (Abs)
0.1	0.5746	0.5761	0.5754
0.2	0.5379	0.5362	0.5370
0.5	0.4625	0.4638	0.4631
1	0.3847	0.3865	0.3856
2	0.2964	0.2951	0.2957
5	0.1832	0.1845	0.1839
10	0.1276	0.1263	0.1270
20	0.0863	0.0849	0.0856

$100 \times \left(\frac{{}_{\text{sample}}A_{\text{control}} - A}{{}_{\text{min}}A_{\text{control}} - A} \right)$ =% of Inhibition

Concentration (µM)	(Abs)	% Inhibition
0.1	0.5746	11.782
0.2	0.5379	17.934
0.5	0.4625	30.598
1	0.3847	43.281
2	0.2964	56.924
5	0.1832	75.316
10	0.1276	86.409
20	0.0863	95.326

Log_{10}(conc)	% Inhibition
-1.0000	11.782
-0.6989	17.934
-0.3010	30.598
0.0000	43.281
0.3010	56.924
0.6989	75.316
1.0000	86.409
1.3010	95.326

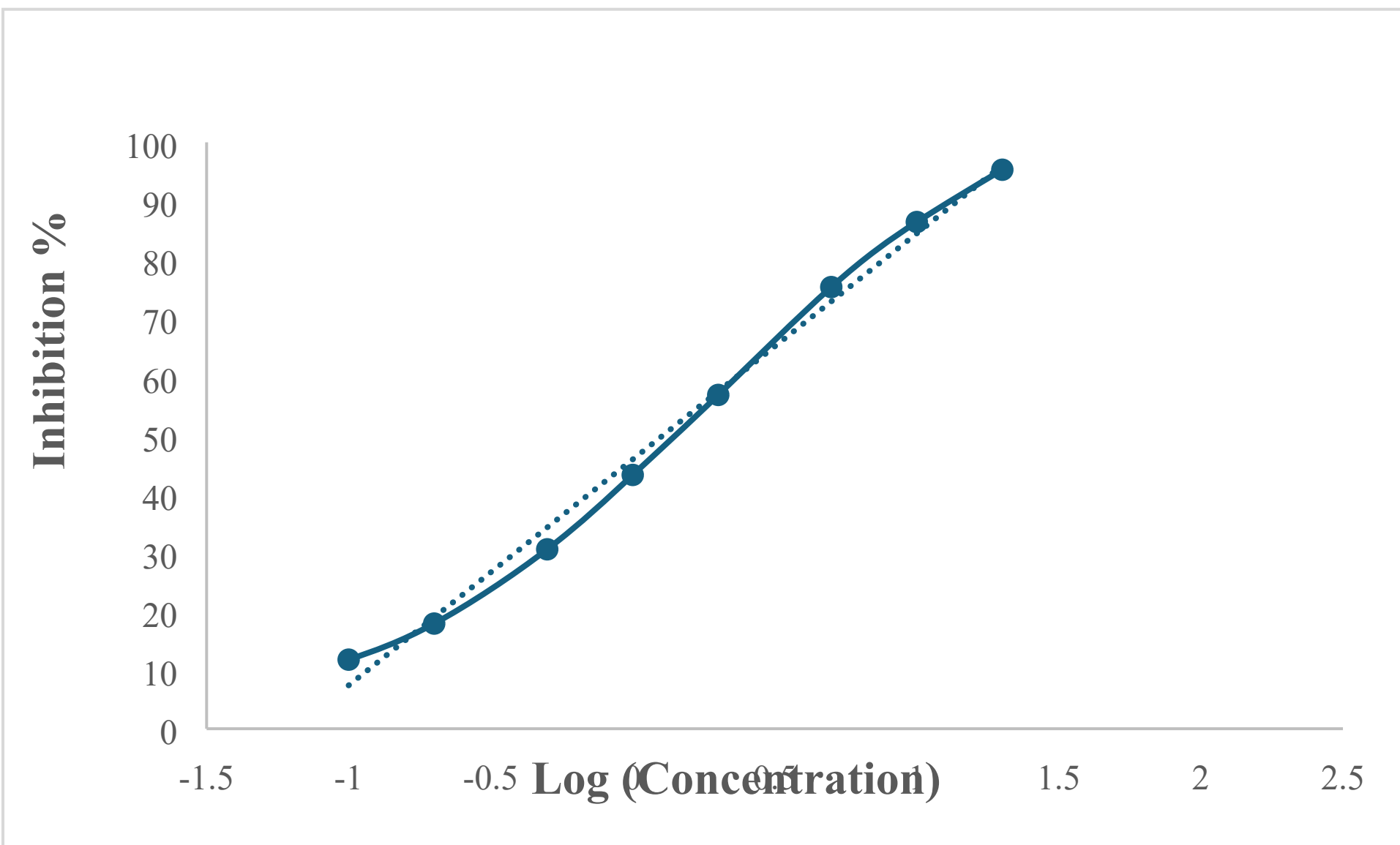

Compound KM-4 (4k)

IC_{50} = 4.973 µM

Concentration (µM)	1 (Abs)	2 (Abs)	Mean (Abs)
0.1	0.6379	0.6364	0.6371
0.2	0.6123	0.6137	0.6130
0.5	0.5516	0.5531	0.5523
1	0.4782	0.4768	0.4775
2	0.3926	0.3941	0.3933
5	0.2785	0.2771	0.2778
10	0.1843	0.1857	0.1850
20	0.1287	0.1272	0.1280

$$100 \times \left(\frac{\text{sample} A_{\text{control}} - A}{\text{min} A_{\text{control}} - A} \right) = \text{\% of Inhibition}$$

Concentration (µM)	(Abs)	% Inhibition
0.1	0.6379	9.582
0.2	0.6123	13.927
0.5	0.5516	22.750
1	0.4782	32.969
2	0.3926	45.649
5	0.2785	62.066
10	0.1843	76.478
20	0.1287	83.672

Log_{10}(conc)	% Inhibition
-1.0000	9.582
-0.6989	13.927
-0.3010	22.750
0.0000	32.969
0.3010	45.649
0.6989	62.066
1.0000	76.478
1.3010	83.672

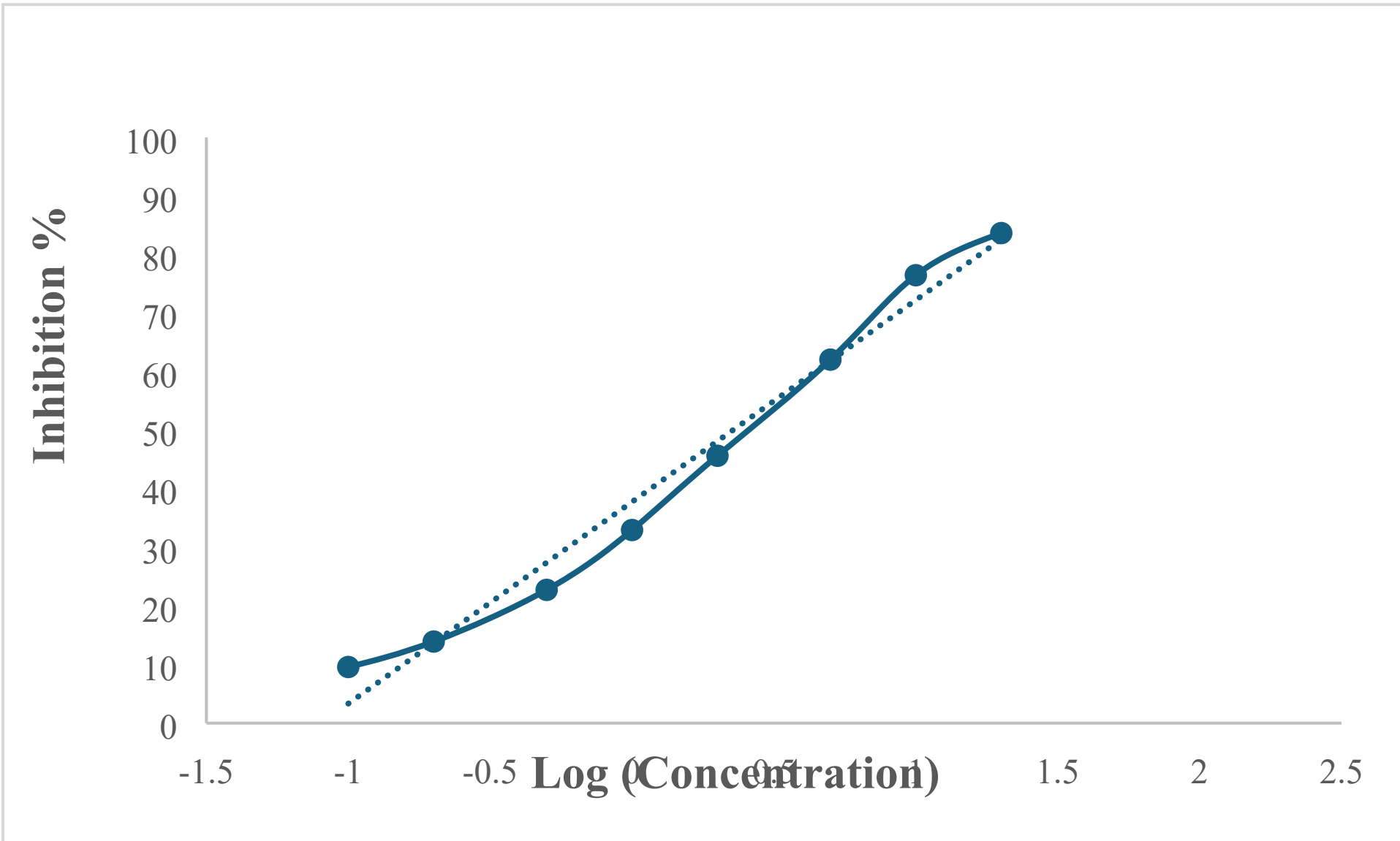

Compound KM-5 (4n)

IC_{50} = 12.628 µM

Concentration (µM)	1 (Abs)	2 (Abs)	Mean (Abs)
0.1	0.6423	0.6437	0.6430

Concentration (µM)	1 (Abs)	2 (Abs)	Mean (Abs)
0.2	0.6274	0.6285	0.6279
0.5	0.5987	0.6001	0.5994
1	0.5562	0.5549	0.5555
2	0.4891	0.4883	0.4887
5	0.3873	0.3859	0.3866
10	0.2927	0.2939	0.2933
20	0.2284	0.2269	0.2276

$100 \times \left(\frac{\text{sample}A_{\text{control}} - A}{\text{min}A_{\text{control}} - A}\right)$ =% of Inhibition

Concentration (µM)	(Abs)	% Inhibition
0.1	0.6423	2.8351
0.2	0.6274	5.5379
0.5	0.5987	10.934
1	0.5562	18.897
2	0.4891	30.961
5	0.3873	48.536
10	0.2927	62.183
20	0.2284	68.932

Log_{10}(conc)	% Inhibition
-1.0000	2.8351
-0.6989	5.5379
-0.3010	10.934
0.0000	18.897
0.3010	30.961
0.6989	48.536
1.0000	62.183
1.3010	68.932

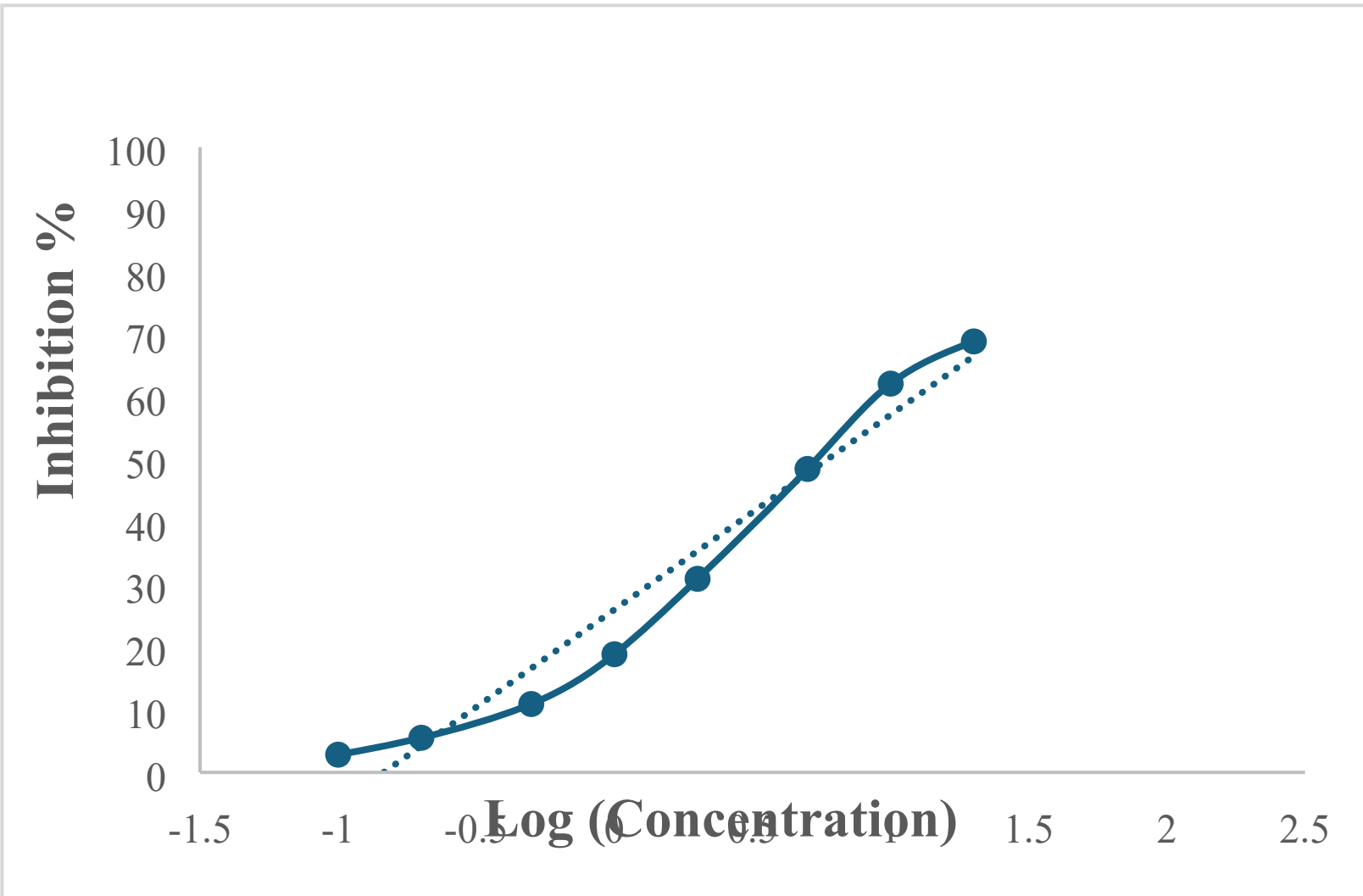

Compound KM-6 (4j)

IC_{50} = 9.084 µM

Concentration (µM)	1 (Abs)	2 (Abs)	Mean (Abs)
0.1	0.6741	0.6728	0.6735
0.2	0.6479	0.6465	0.6472
0.5	0.6037	0.6023	0.6030
1	0.5348	0.5361	0.5354

Concentration (µM)	1 (Abs)	2 (Abs)	Mean (Abs)
2	0.4473	0.4458	0.4465
5	0.3362	0.3376	0.3369
10	0.2487	0.2474	0.2480
20	0.1924	0.1938	0.1931

$$100 \times \left(\frac{{}_{sample}A_{control} - A}{{}_{min}A_{control} - A} \right) = \% \text{ of Inhibition}$$

Concentration (µM)	(Abs)	% Inhibition
0.1	0.6741	4.6234
0.2	0.6479	7.4659
0.5	0.6037	13.648
1	0.5348	23.915
2	0.4473	37.927
5	0.3362	54.267
10	0.2487	67.319
20	0.1924	74.328

Log_{10}(conc)	% Inhibition
-1.0000	4.6234
-0.6989	7.4659
-0.3010	13.648
0.0000	23.915
0.3010	37.927
0.6989	54.267
1.0000	67.319
1.3010	74.328

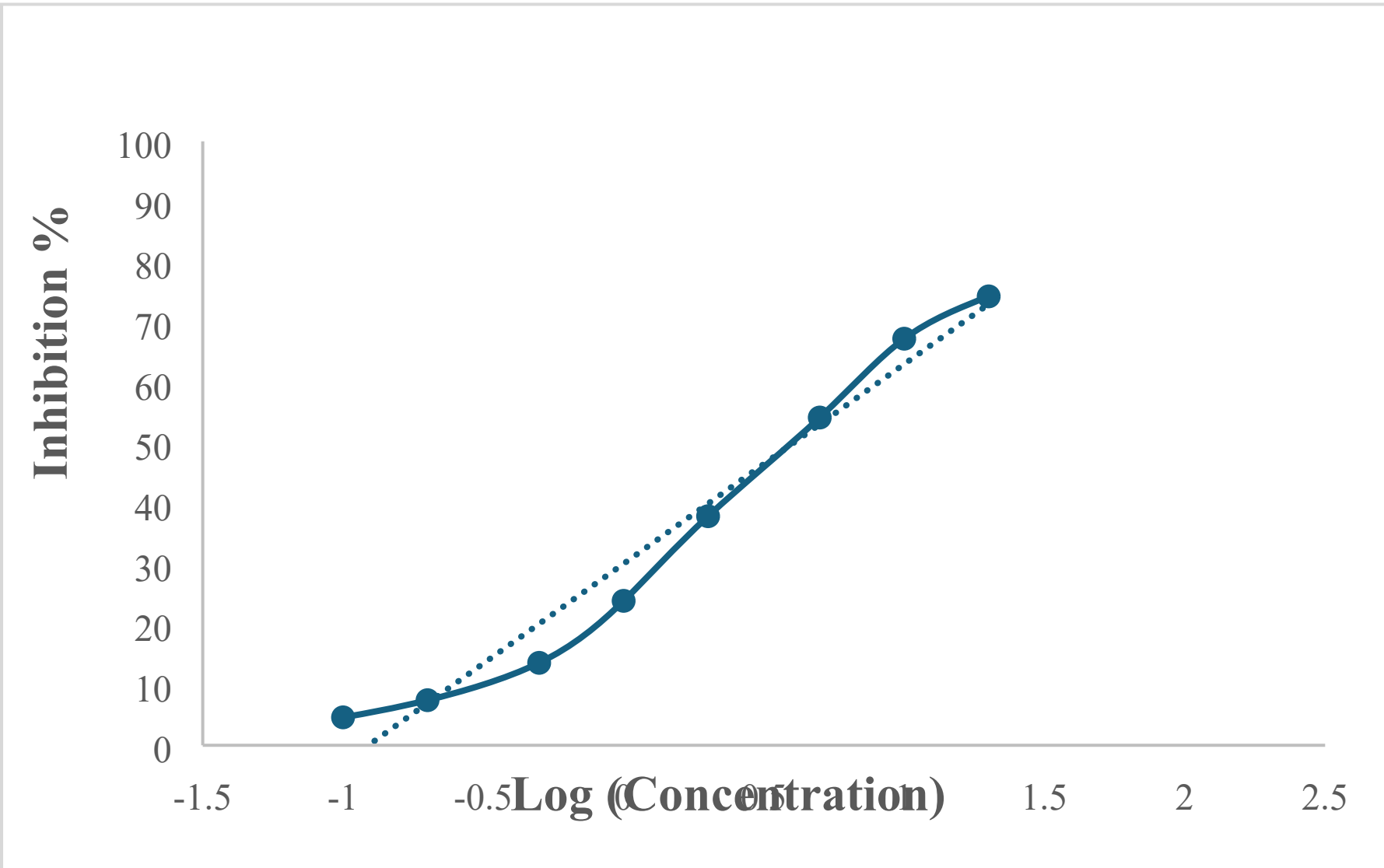

Compound KM-7 (4m)

IC_{50} = 1.217 µM

Concentration (µM)	1 (Abs)	2 (Abs)	Mean (Abs)
0.1	0.5972	0.5956	0.5964
0.2	0.5638	0.5651	0.5644
0.5	0.4816	0.4803	0.4810
1	0.3941	0.3954	0.3948
2	0.2873	0.2887	0.2880
5	0.1762	0.1776	0.1769
10	0.0934	0.0921	0.0927

Concentration (µM)	1 (Abs)	2 (Abs)	Mean (Abs)
20	0.0485	0.0498	0.0491

$$100 \times \left(\frac{\text{sample}A_{\text{control}} - A}{\text{min}A_{\text{control}} - A} \right)$$ =% of Inhibition

Concentration (µM)	(Abs)	% Inhibition
0.1	0.5972	12.399
0.2	0.5638	17.507
0.5	0.4816	28.832
1	0.3941	41.372
2	0.2873	56.285
5	0.1762	73.442
10	0.0934	87.879
20	0.0485	96.436

Log_{10}(conc)	% Inhibition
-1.0000	12.399
-0.6989	17.507
-0.3010	28.832
0.0000	41.372
0.3010	56.285
0.6989	73.442
1.0000	87.879
1.3010	96.436

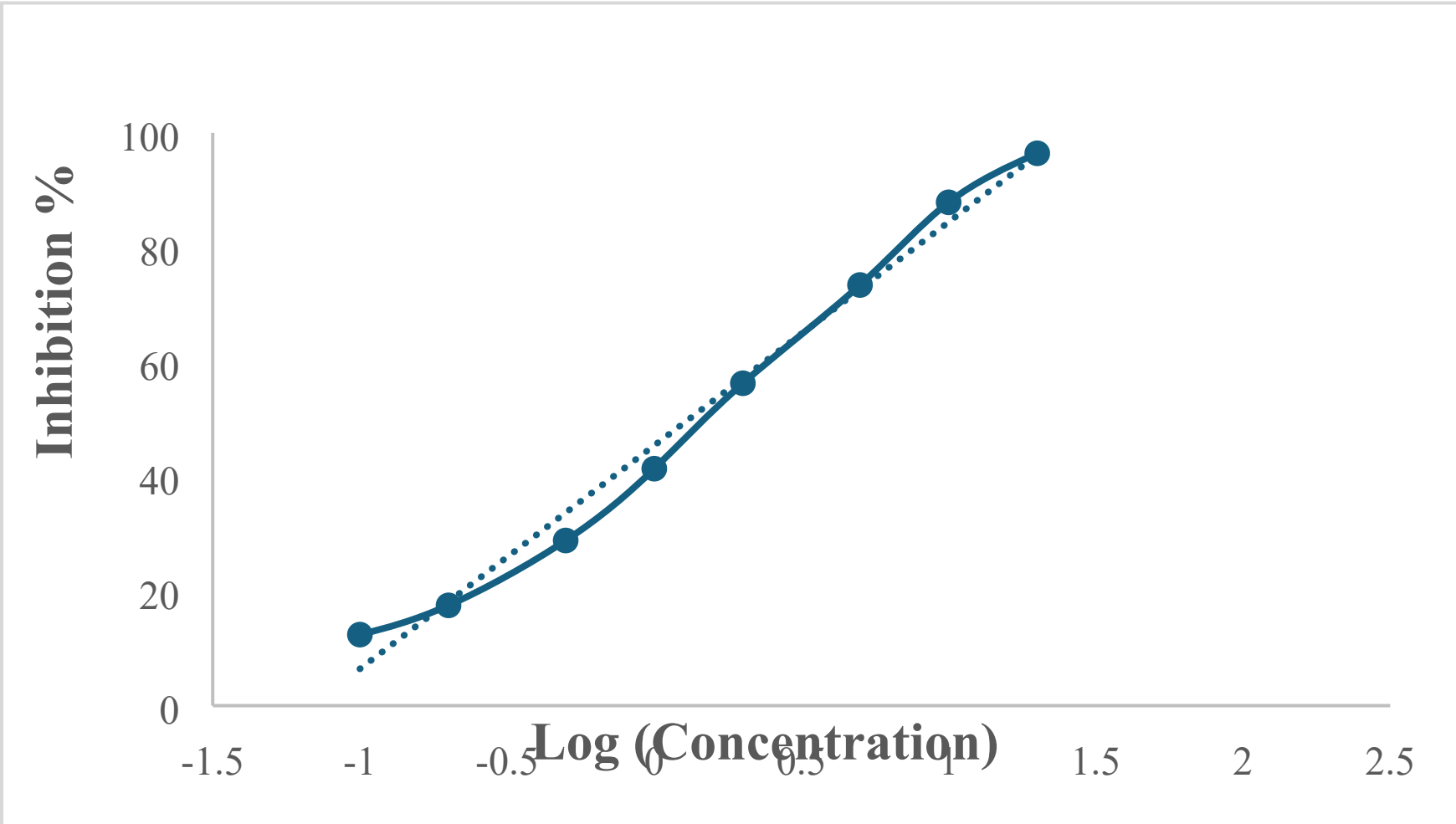

Compound KM-8 (4i)

IC_{50} = 11.295 µM

Concentration (µM)	1 (Abs)	2 (Abs)	Mean (Abs)
0.1	0.7124	0.7107	0.7116
0.2	0.6849	0.6835	0.6842
0.5	0.6437	0.6419	0.6428
1	0.5893	0.5881	0.5887
2	0.5096	0.5109	0.5102
5	0.4078	0.4065	0.4072
10	0.3262	0.3248	0.3255
20	0.2381	0.2394	0.2388

$$100 \times \left(\frac{\text{sample}A_{\text{control}} - A}{\text{min}A_{\text{control}} - A} \right)$$ =% of Inhibition

Concentration (μM)	(Abs)	% Inhibition
0.1	0.7124	3.8532
0.2	0.6849	7.8891
0.5	0.6437	15.596
1	0.5893	24.658
2	0.5096	35.872
5	0.4078	51.126
10	0.3262	63.489
20	0.2381	70.298

Log_{10}(conc)	% Inhibition
-1.0000	3.8532
-0.6989	7.8891
-0.3010	15.596
0.0000	24.658
0.3010	35.872
0.6989	51.126
1.0000	63.489
1.3010	70.298

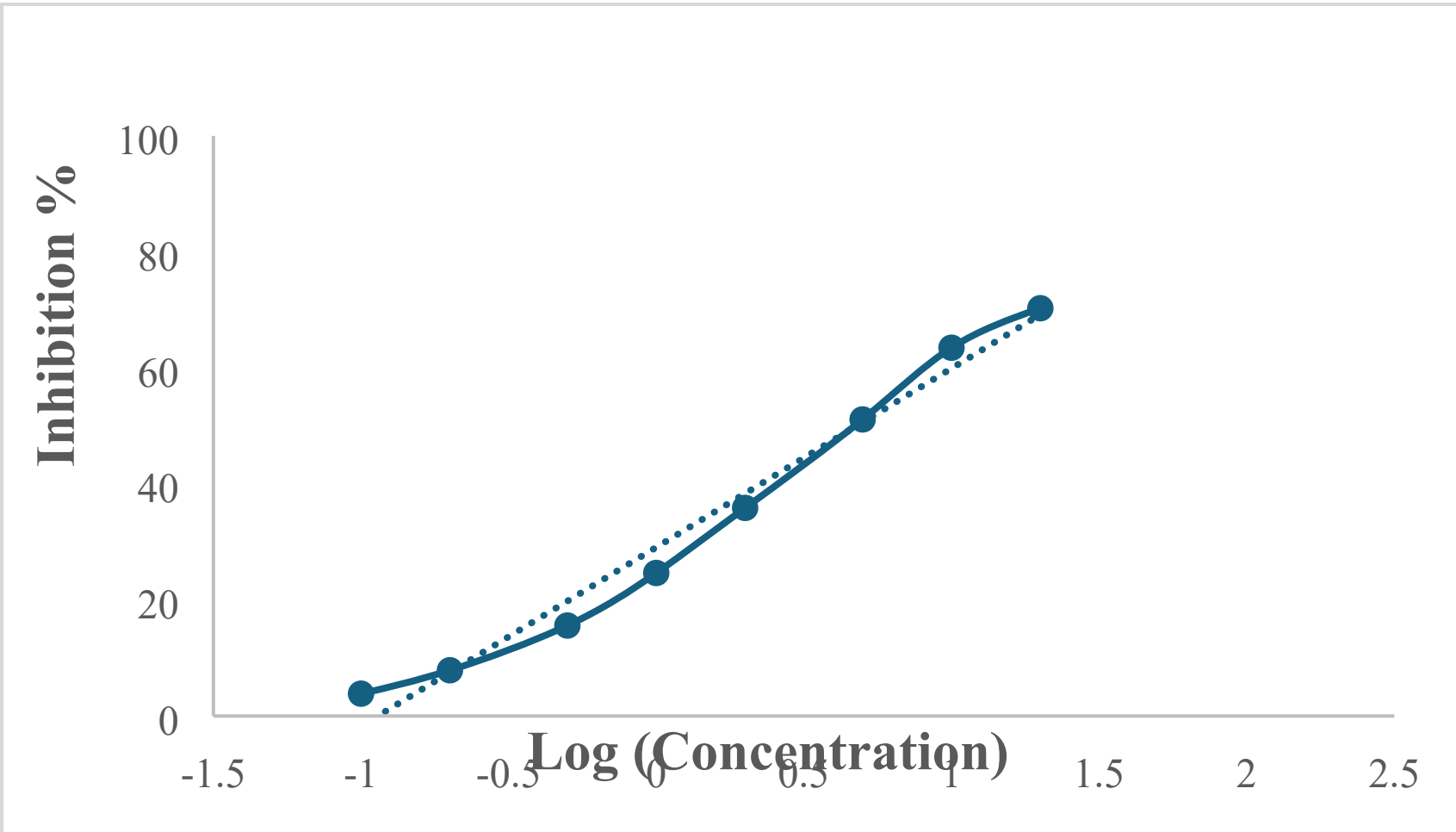

Compound KM-9 (4d)

IC_{50} = 16.467 μM

Concentration (μM)	1	2	Mean
0.1	0.7164	0.7149	0.7156
0.2	0.7028	0.7043	0.7035
0.5	0.6781	0.6768	0.6775
1	0.6387	0.6402	0.6395
2	0.5839	0.5826	0.5832
5	0.4932	0.4918	0.4925
10	0.3875	0.3861	0.3868
20	0.2936	0.2952	0.2944

$$100 \times \left(\frac{{}_{\mathrm{sample}}A_{\mathrm{control}} - A}{{}_{\mathrm{min}}A_{\mathrm{control}} - A} \right)$$ =% of Inhibition

Concentration (μM)	(Abs)	% Inhibition
0.1	0.7164	1.7832

Concentration (µM)	(Abs)	% Inhibition
0.2	0.7028	3.8054
0.5	0.6781	7.7229
1	0.6387	13.7635
2	0.5839	21.6201
5	0.4932	33.7317
10	0.3875	49.8389
20	0.2936	61.5703

Log_{10}(conc)	% Inhibition
-1.0000	1.7832
-0.6989	3.8054
-0.3010	7.7229
0.0000	13.7635
0.3010	21.6201
0.6989	33.7317
1.0000	49.8389
1.3010	61.5703

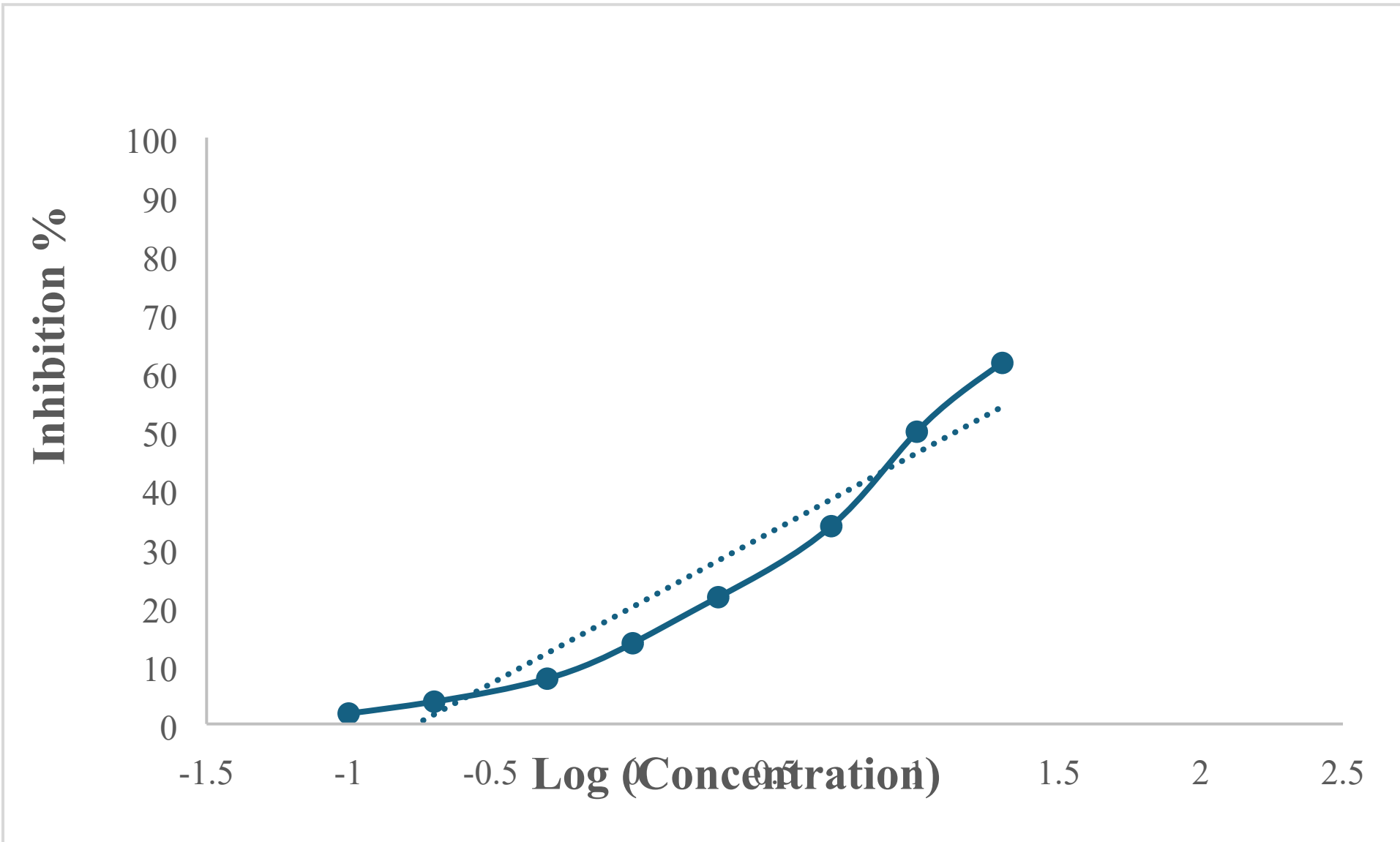

Compound KM-10 (4h)
IC_{50} = 7.178 µM

Concentration (µM)	1	2	Mean
0.1	0.6837	0.6821	0.6829
0.2	0.6594	0.6608	0.6601
0.5	0.6275	0.6289	0.6282
1	0.5823	0.5835	0.5829
2	0.4936	0.4921	0.4928
5	0.3764	0.3751	0.3758
10	0.2691	0.2676	0.2683
20	0.1957	0.1972	0.1965

$100 \times \left(\frac{A_{control} - A_{sample}}{A_{control} - A_{min}}\right)$ =% of Inhibition

Concentration (µM)	(Abs)	% Inhibition
0.1	0.6837	5.0884
0.2	0.6594	8.4243
0.5	0.6275	13.1961
1	0.5823	21.8726

Concentration (µM)	(Abs)	% Inhibition
2	0.4936	35.7549
5	0.3764	52.7967
10	0.2691	68.1693
20	0.1957	77.3392

Log_{10}(conc)	% Inhibition
-1.0000	5.0884
-0.6989	8.4243
-0.3010	13.1961
0.0000	21.8726
0.3010	35.7549
0.6989	52.7967
1.0000	68.1693
1.3010	77.3392

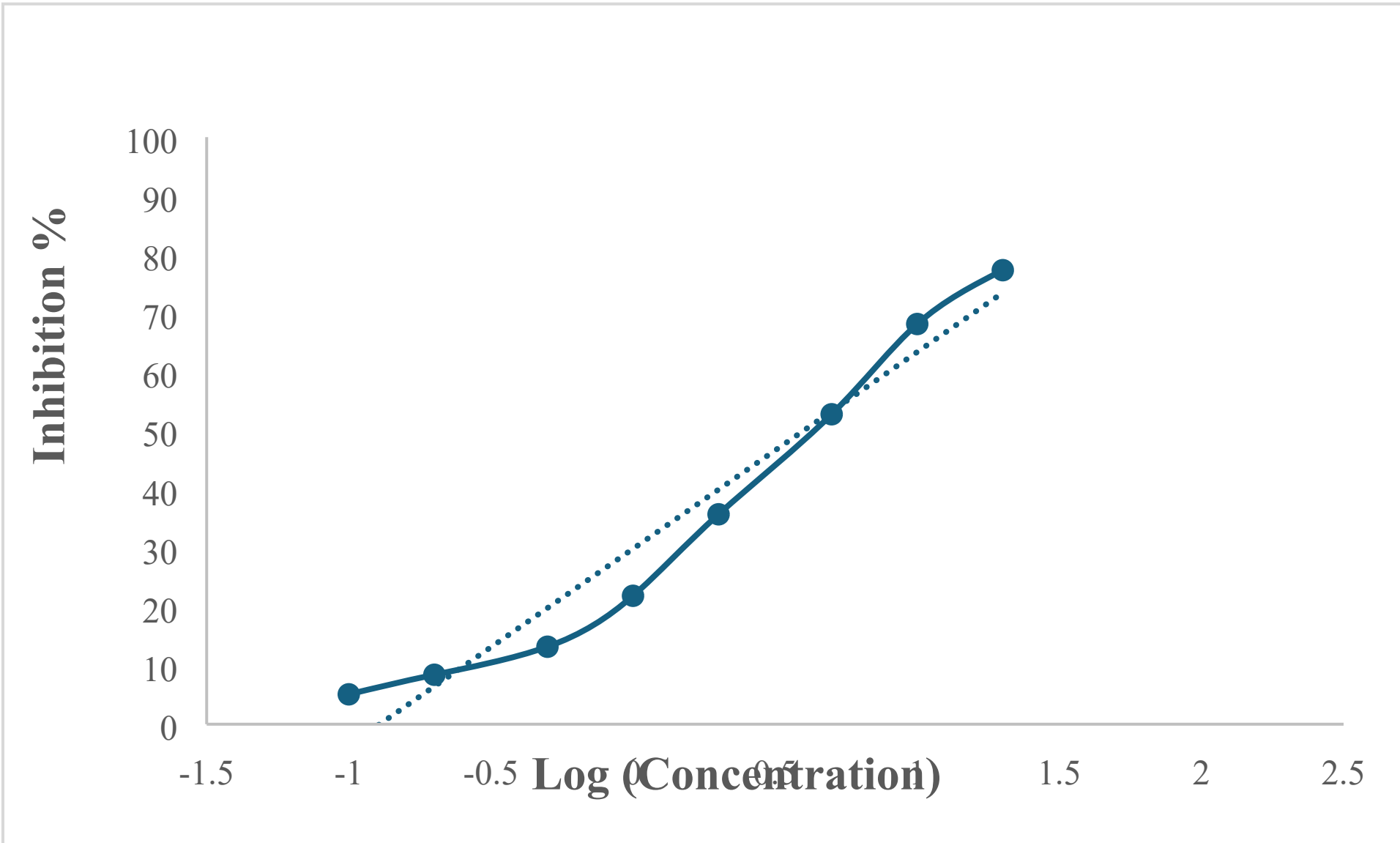

Compound KM-11 (4o)

IC_{50} = 9.699 µM

Concentration (µM)	1	2	Mean
0.1	0.7124	0.7138	0.7131
0.2	0.6897	0.6909	0.6903
0.5	0.6612	0.6625	0.6619
1	0.6238	0.6250	0.6244
2	0.5485	0.5472	0.5479
5	0.4376	0.4362	0.4369
10	0.3268	0.3279	0.3274
20	0.2805	0.2813	0.2809

$$100 \times \left(\frac{{}_{\text{sample}}A_{\text{control}} - A}{{}_{\text{min}}A_{\text{control}} - A} \right)$$ =% of Inhibition

Concentration (µM)	(Abs)	% Inhibition
0.1	0.7124	4.9733
0.2	0.6897	9.4121
0.5	0.6612	16.7334
1	0.6238	26.6127
2	0.5485	41.8829
5	0.4376	59.1145
10	0.3268	70.8144

Concentration (µM)	(Abs)	% Inhibition
20	0.2805	73.6933

Log$_{10}$(conc)	% Inhibition
-1.0000	4.9733
-0.6989	9.4121
-0.3010	16.7334
0.0000	26.6127
0.3010	41.8829
0.6989	59.1145
1.0000	70.8144
1.3010	73.6933

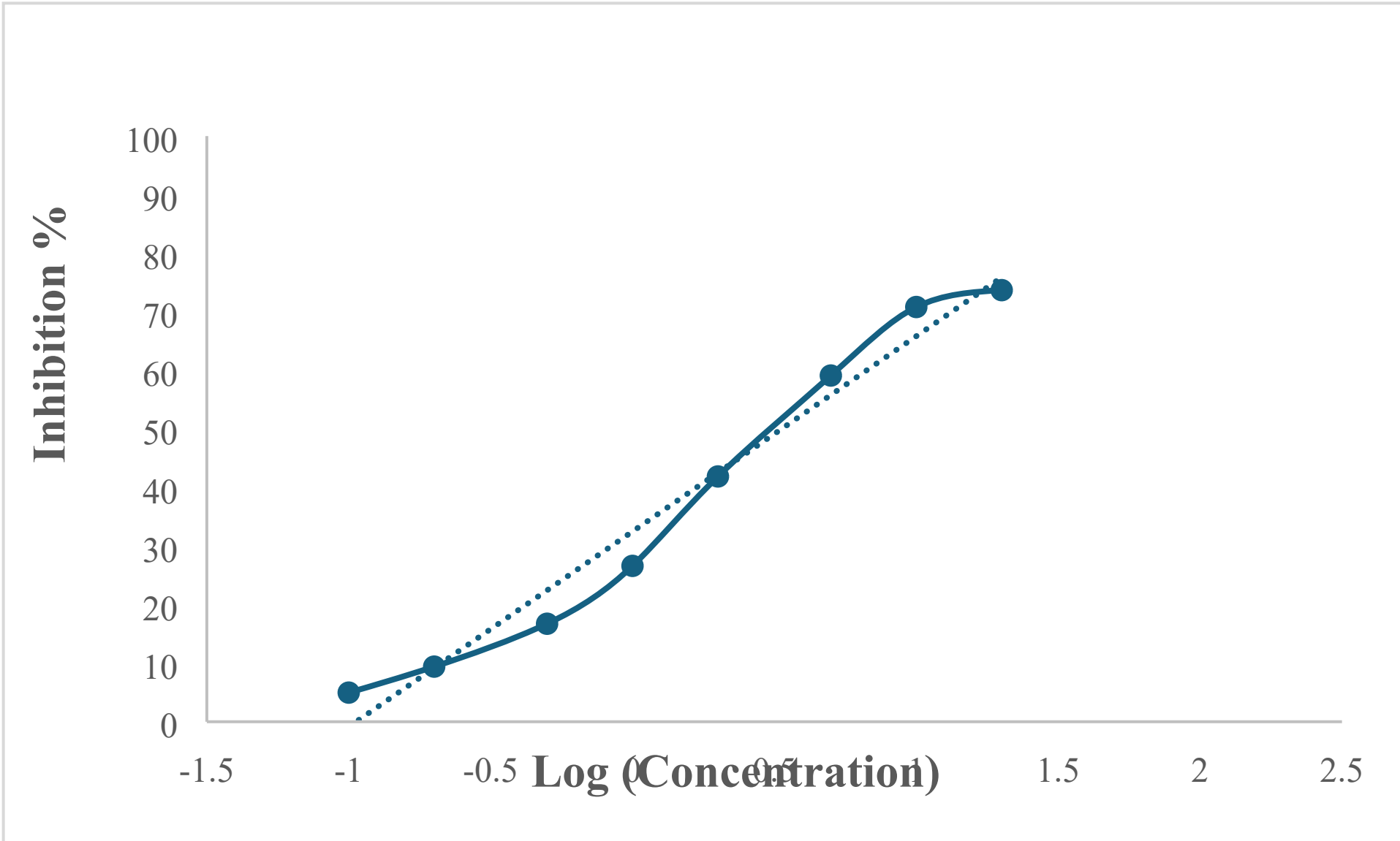

Compound KM-12 (4c)

IC$_{50}$ = 6.541 µM

Concentration (µM)	1	2	Mean
0.1	0.6982	0.6997	0.6990
0.2	0.6751	0.6765	0.6758
0.5	0.6339	0.6351	0.6345
1	0.5938	0.5949	0.5944
2	0.5217	0.5203	0.5210
5	0.4136	0.4145	0.4141
10	0.3198	0.3211	0.3204
20	0.2934	0.2942	0.2938

$$100 \times \left(\frac{{}_{\text{sample}}A_{\text{control}} - A}{{}_{\text{min}}A_{\text{control}} - A} \right)$$ =% of Inhibition

Concentration (µM)	(Abs)	% Inhibition
0.1	0.6982	5.9773
0.2	0.6751	12.4851
0.5	0.6339	24.8582
1	0.5938	36.1245
2	0.5217	54.9831
5	0.4136	69.2117
10	0.3198	77.1043
20	0.2934	79.8940

Log$_{10}$(conc)	% Inhibition

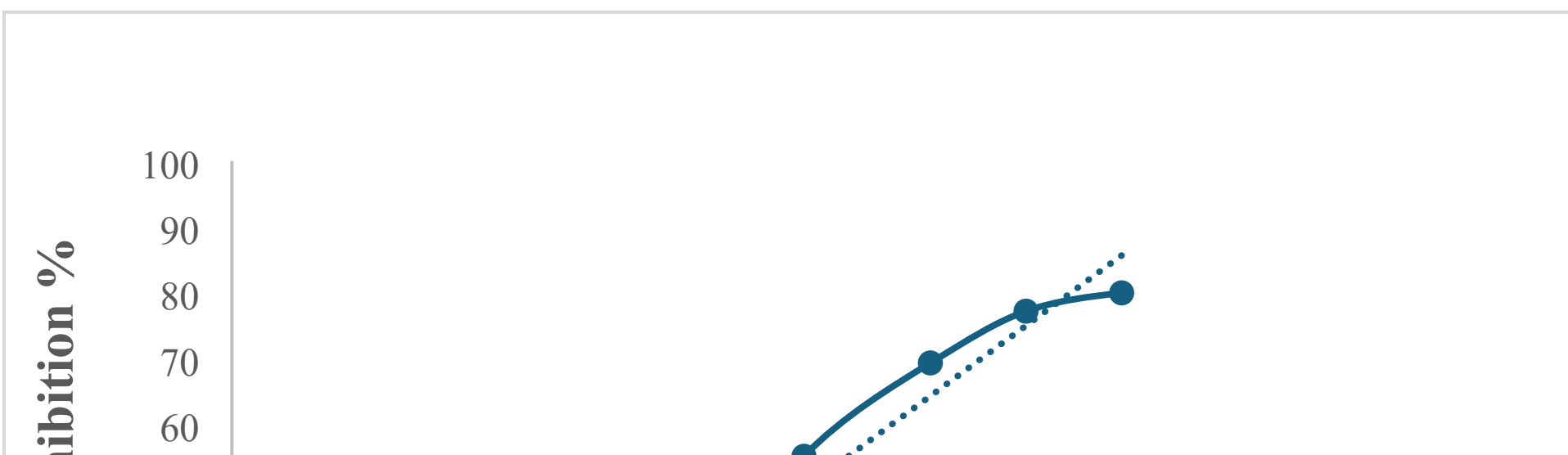

Log$_{10}$(conc)	% Inhibition
-1.0000	5.9773
-0.6989	12.4851
-0.3010	24.8582
0.0000	36.1245
0.3010	54.9831
0.6989	69.2117
1.0000	77.1043
1.3010	79.8940

Compound Indomethacin

IC$_{50}$ = 1.106 **µM**

Concentration (µM)	1	2	Mean
0.1	0.5824	0.5838	0.5831
0.2	0.5569	0.5581	0.5575
0.5	0.4981	0.4975	0.4978
1	0.4423	0.4417	0.4420
2	0.3287	0.3291	0.3289
5	0.2115	0.2109	0.2112
10	0.1754	0.1747	0.1750
20	0.1592	0.1587	0.1589

$$100 \times \left(\frac{{}_{\text{sample}}A_{\text{control}} - A}{{}_{\text{min}}A_{\text{control}} - A} \right) = \text{\% of Inhibition}$$

Concentration (µM)	(Abs)	% Inhibition
0.1	0.5824	13.042
0.2	0.5569	20.521
0.5	0.4981	34.788
1	0.4423	47.239
2	0.3287	72.436
5	0.2115	90.276
10	0.1754	95.814
20	0.1592	97.289

Log$_{10}$(conc)	% Inhibition
-1.0000	13.042
-0.6989	20.521
-0.3010	34.788

Log_{10}(conc)	% Inhibition
0.0000	47.239
0.3010	72.436
0.6989	90.276
1.0000	95.814
1.3010	97.289

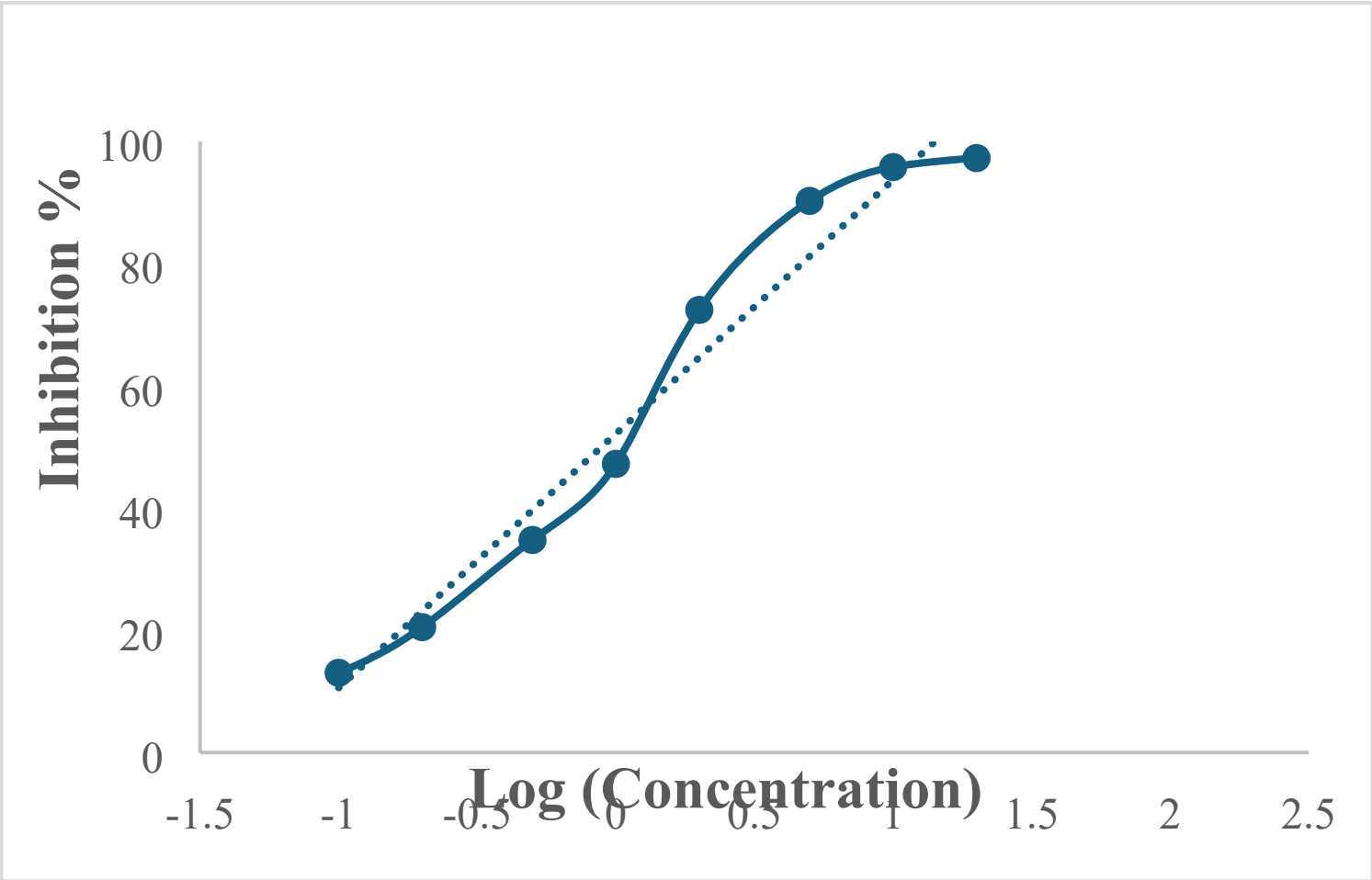

Soluble Epoxide Hydrolase (sEH) Raw Data

AUDA, IC_{50}= 2.674 µM

Concentration (µM)	Log10(Conc)	% Inhibition
50	1.69897	94.923
25	1.39794	90.337
12.5	1.09691	82.377
6.25	0.79588	70.035
3.125	0.49485	53.878
1.562	0.19382	36.874
0.781	−0.10734	22.604

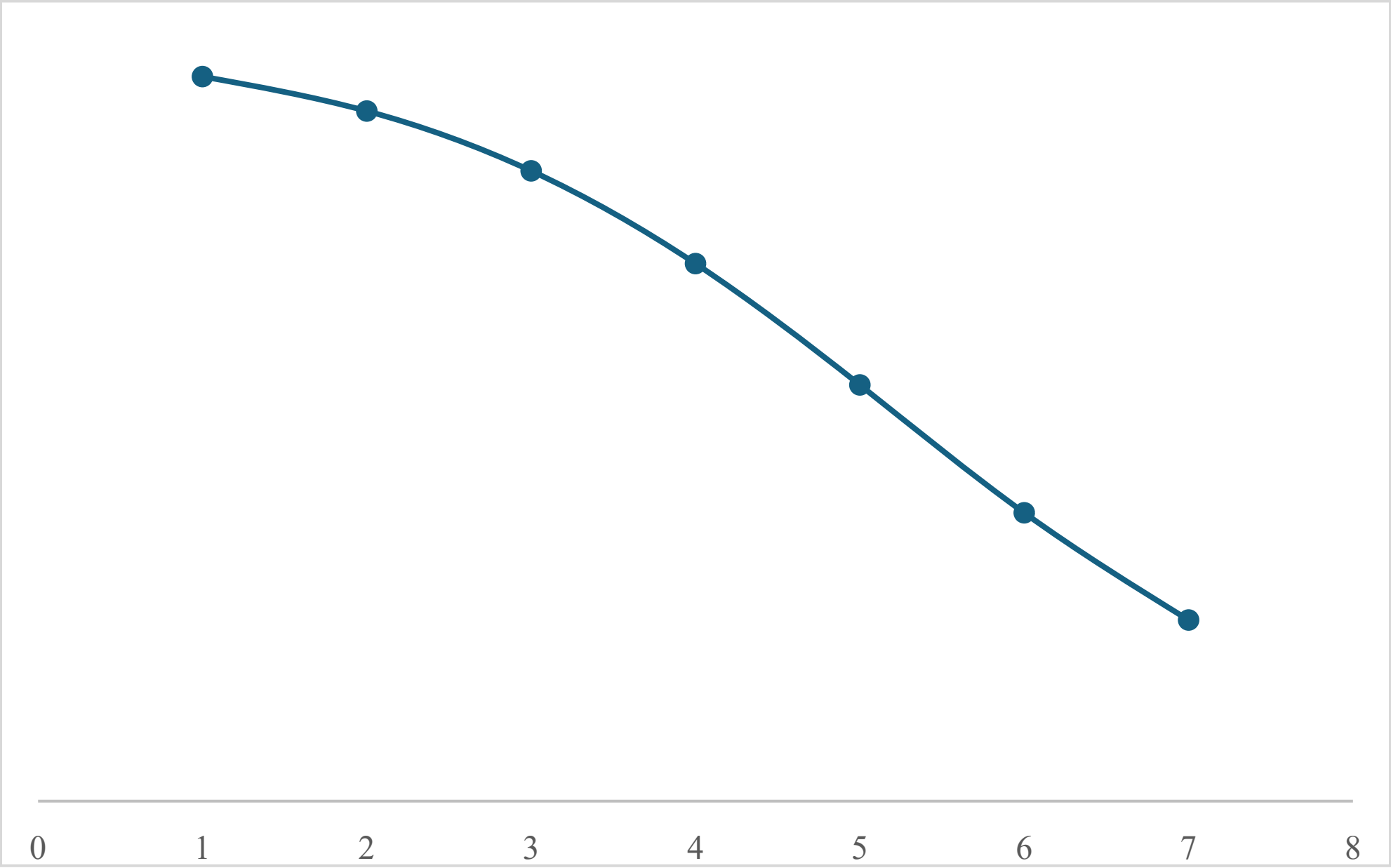

Compound 4c, IC_{50}= 4.329 µM

Concentration (µM)	Log10(Conc)	% Inhibition
50	1.69897	84.772
25	1.39794	73.459
12.5	1.09691	58.214
6.25	0.79588	39.861
3.125	0.49485	27.542
1.562	0.19382	19.721
0.781	−0.10735	16.983

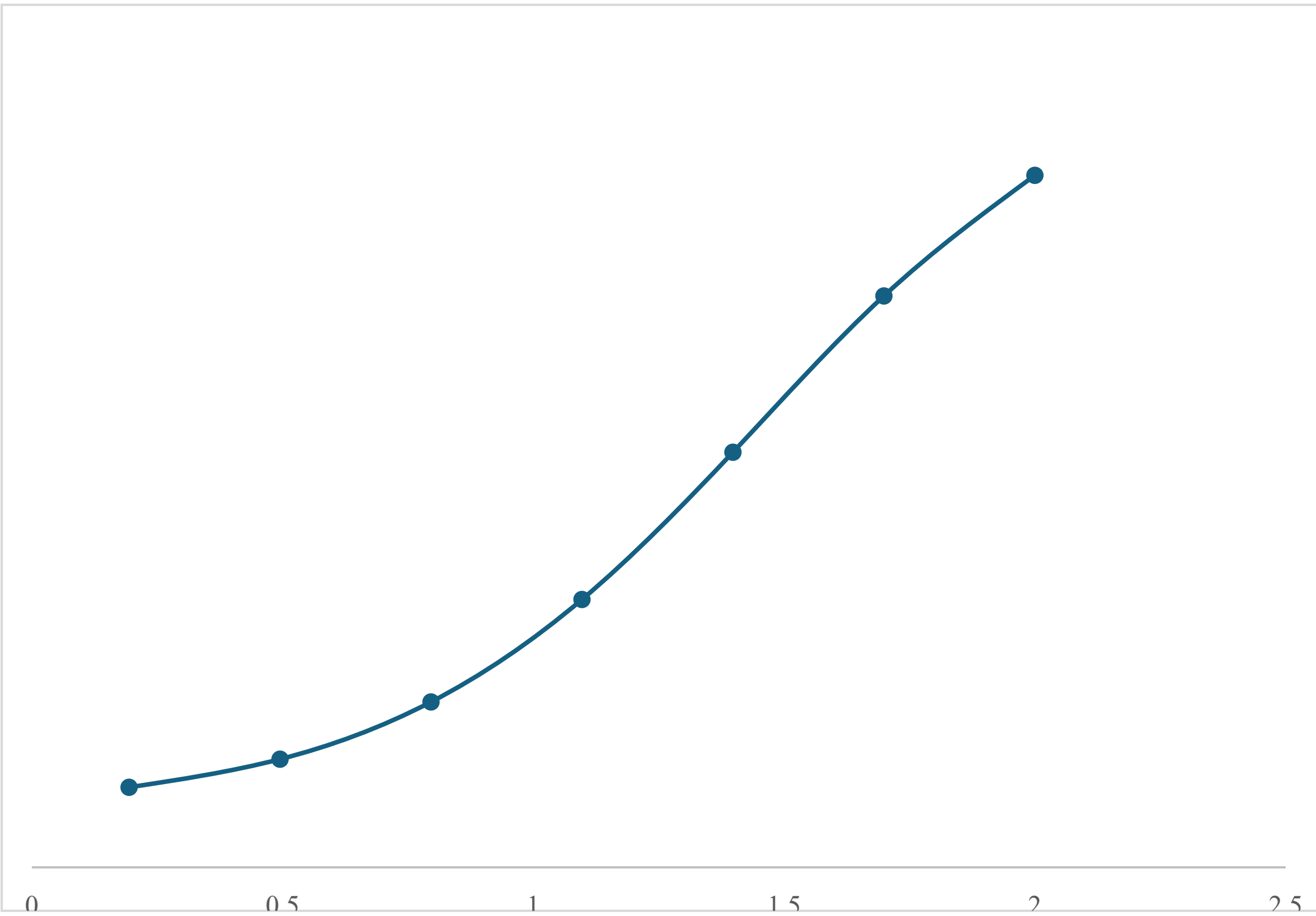

Concentration (µM)	OD1	OD2	OD_avg
50	0.018942	0.018801	0.018872
25	0.034201	0.034039	0.034120
12.5	0.052001	0.051935	0.051968
6.25	0.078900	0.079125	0.079012
3.125	0.096540	0.096359	0.096450
1.562	0.103121	0.102821	0.102971
0.781	0.108561	0.108403	0.108482

Concentration (µM)	A_0 (initial)	A_{20} (after 20 min)	$\Delta A = A_{20} - A_0$
50	0.009881	0.028623	0.018742
25	0.009743	0.043142	0.033399
12.5	0.009998	0.058223	0.048225
6.25	0.009921	0.077700	0.067779
3.125	0.010178	0.091892	0.081714
1.562	0.010049	0.099783	0.089734
0.781	0.010201	0.106882	0.096681

Concentration (µM)	OD1	OD2	OD_avg

Concentration (µM)	OD1	OD2	OD_avg
50	0.013020	0.013188	0.013104
25	0.026749	0.026813	0.026781
12.5	0.044397	0.044292	0.044344
6.25	0.074717	0.074755	0.074736
3.125	0.096553	0.096533	0.096543
1.562	0.108819	0.108760	0.108789
0.781	0.115650	0.115636	0.115643

Concentration (µM)	A_0 (initial)	A_{20} (after 20 min)	$\Delta A = A_{20} - A_0$
50	0.009611	0.018794	0.009183
25	0.010343	0.029138	0.018795
12.5	0.010276	0.041251	0.030975
6.25	0.009715	0.062005	0.052290
3.125	0.010055	0.077570	0.067515
1.562	0.009796	0.086026	0.076230
0.781	0.010315	0.091308	0.080993

Compound 4j, IC_{50}= 7.415 µM

Log10(Conc.)	% Inhibition
2	86.469
1.69897	73.158
1.39794	60.004
1.09691	41.732
0.79588	31.128
0.49485	22.947
0.19382	18.261

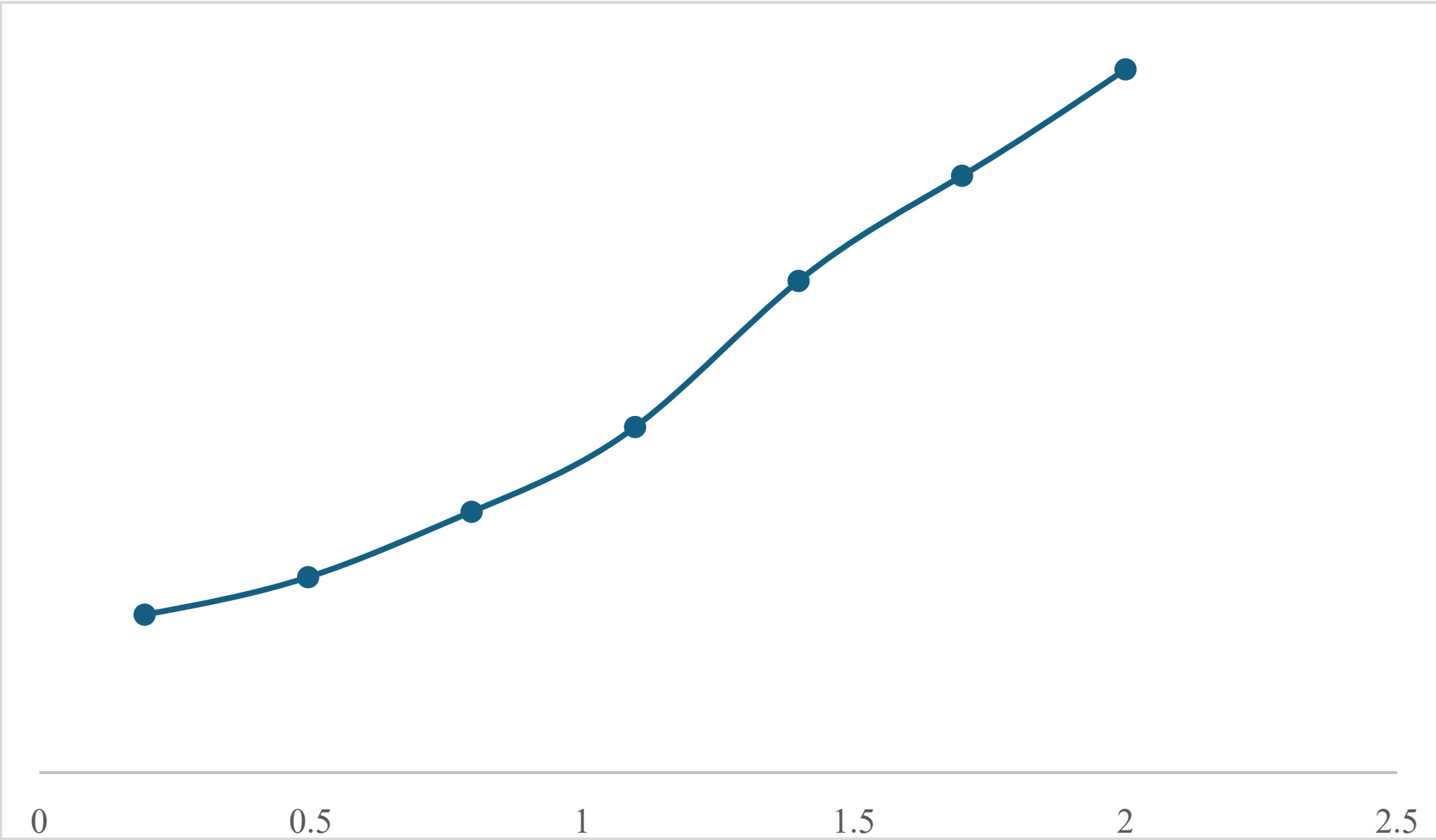

Concentration (μM)	RLU	RLU	Mean RLU
100	8449.511	8437.177	8443.344
50	16703.394	16795.422	16749.408

Concentration (µM)	RLU	RLU	Mean RLU
25	25062.008	24853	24957.504
12.500	36497.971	36220.493	36359.232
6.250	42963.418	42988.838	42976.128
3.125	48151.811	48010.333	48081.072
1.562	50874.089	51136.183	51005.136

Concentration (µM)	Mean RLU	% Inhibition
100	8443.344	86.469
50	16749.408	73.158
25	24957.504	60.004
12.500	36359.232	41.732
6.250	42976.128	31.128
3.125	48081.072	22.947
1.562	51005.136	18.261

Compound 4o, IC_{50}= 8.372

Concentration (µM)	log10(Conc)	A_initial	ΔA	A1 (A_final)	A2 (A_final)	µmole (B)	%Inhibition
0.625	-0.20412	0.1	0.2291406	0.3289406	0.3293406	5.7285144	20.4373

1.25	0.09691	0.1	0.2158623	0.3156623	0.3160623	5.3965584	25.0478
2.5	0.39794	0.1	0.1938856	0.2938856	0.2940856	4.8471408	32.6786
5	0.69897	0.1	0.1622586	0.2622586	0.2624586	4.0564656	43.6602
10	1. 0	0.1	0.1248267	0.2248267	0.2246267	3.1206672	56.6574
20	1.30103	0.1	0.0895427	0.1895427	0.1897427	2.2385664	68.9088
40	1.60206	0.1	0.0627405	0.1627405	0.1625405	1.5685128	78.2151
50	1.69897	0.1	0.0562268	0.1562268	0.1564268	1.4056704	80.4768

Celecoxib, IC_{50}= 19.703

Concentration (μM)	log10(Conc)	A initial	ΔA	A1 (A_final)	A2 (A_final)	μmole(B)	%Inhibition
0.625	-0.20412	0.11	0.2010376	0.311240	0.310836	5.433448	16.4085
1.25	0.09691	0.11	0.1970977	0.307298	0.306998	5.327163	18.0467
2.5	0.39794	0.11	0.1898474	0.350	0.299644	5.131010	21.0460
5	0.69897	0.11	0.1776823	0.287892	0.287472	4.802225	26.1350
10	1.0	0.11	0.1593409	0.259541	0.259141	4.306510	33.7460
20	1.30103	0.11	0.1365405	0.246741	0.246341	3.690285	43.2110
40	1.60206	0.11	0.1139008	0.224101	0.223701	3.078400	52.6400
50	1.69897	0.11	0.1074736	0.217674	0.217274	2.904720	55.3120

5-lipoxigenase activity assay

Compound 4o

IC_{50} = 1.094 μM

Concentration (μM)	Reading 1	Reading 2	Mean Absorbance
0.1	0.7273	0.7261	0.7267
0.2	0.6746	0.6758	0.6752

Concentration (µM)	Reading 1	Reading 2	Mean Absorbance
0.4	0.5971	0.5964	0.5968
1	0.4529	0.4535	0.4532
2	0.3227	0.3214	0.3221
5	0.2159	0.2163	0.2161
10	0.1485	0.1497	0.1491
15	0.1294	0.1281	0.1288

Concentration (µM)	Mean Absorbance	% Inhibition
0.1	0.7267	11.2435
0.2	0.6752	17.5566
0.4	0.5968	27.0799
1	0.4532	44.6541
2	0.3221	60.6577
5	0.2161	73.6107
10	0.1491	81.7932
15	0.1288	86.1840

Log[Conc.] (log10 µM)	% Inhibition
-1.000	11.2435
-0.698	17.5566
-0.398	27.0799
0.000	44.6541
0.301	60.6577
0.699	73.6107
1.000	81.7932
1.176	86.1840

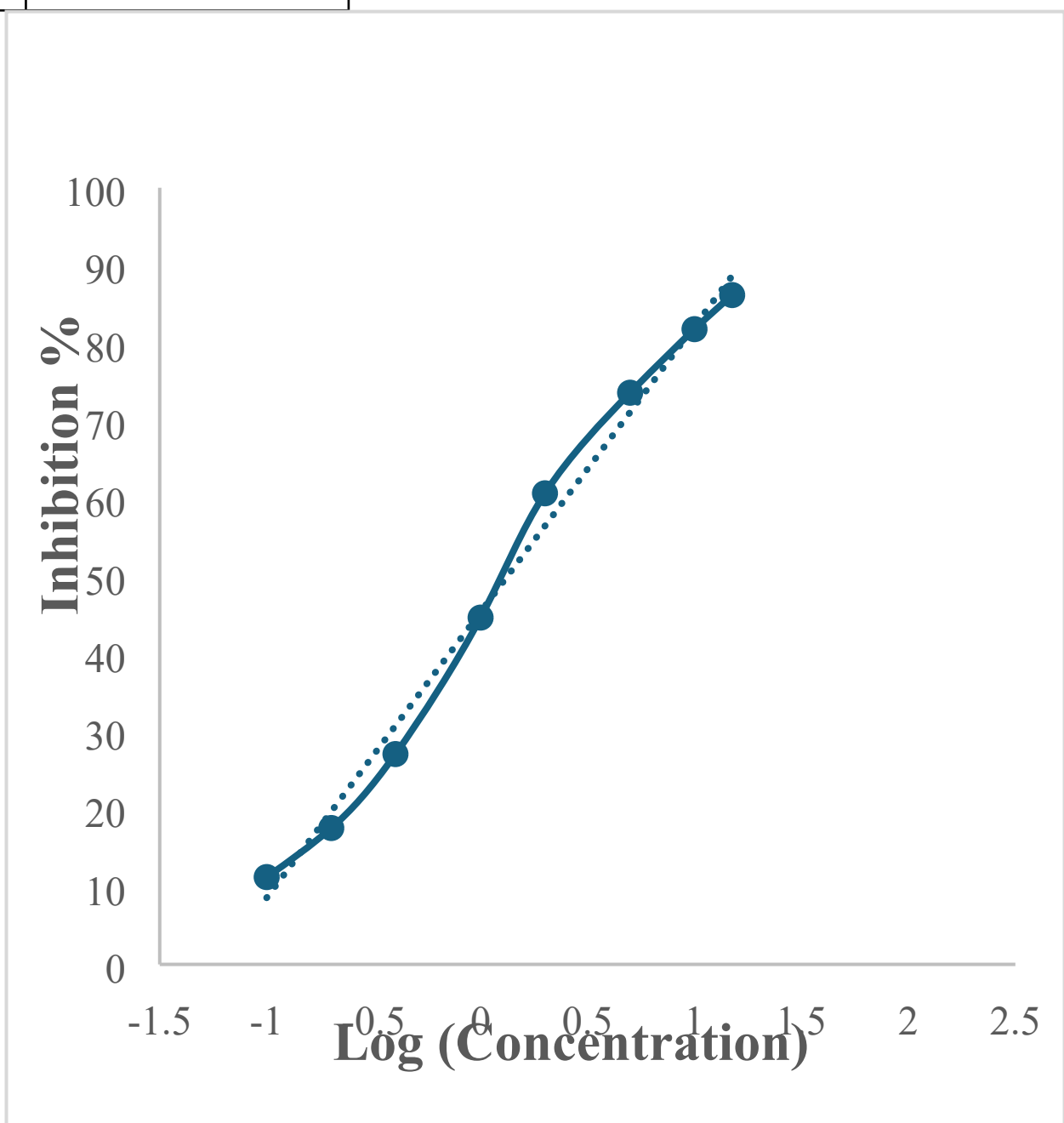

Compound 4m

IC_{50} = 0.927 µM

Concentration (µM)	Reading 1	Reading 2	Mean Absorbance
0.1	0.7238	0.7225	0.7231
0.2	0.6937	0.6949	0.6943
0.4	0.6154	0.6167	0.6160
1	0.4385	0.4373	0.4379
2	0.2918	0.2929	0.2924
5	0.1697	0.1682	0.1690

Concentration (µM)	Reading 1	Reading 2	Mean Absorbance
10	0.1091	0.1103	0.1097
15	0.0937	0.0924	0.0930

Concentration (µM)	Mean Absorbance	% Inhibition
0.1	0.7231	12.42191
0.2	0.6943	15.8247
0.4	0.6160	25.3374
1	0.4379	46.9290
2	0.2924	64.5619
5	0.1690	79.5159
10	0.1097	86.7070
15	0.0930	88.3955

Log[Conc.] (log10 µM)	% Inhibition
-1.000	12.42191
-0.698	15.8247
-0.398	25.3374
0.000	46.9290
0.301	64.5619
0.699	79.5159
1.000	86.7070
1.176	88.3955

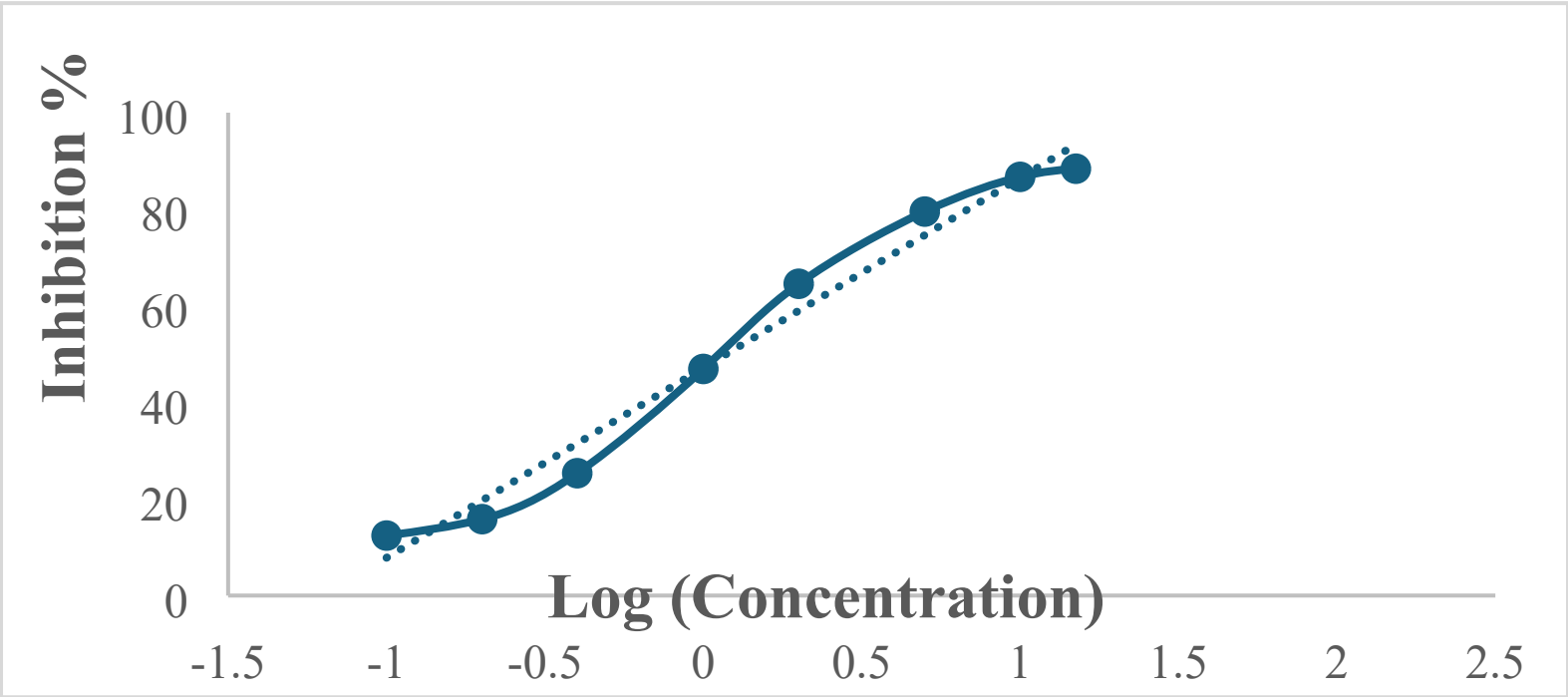

Compound 4j

IC_{50} = 0.802 µM

Concentration (µM)	Reading 1	Reading 2	Mean Absorbance
0.1	0.7451	0.7439	0.7445
0.2	0.7113	0.7097	0.7105
0.4	0.6338	0.6351	0.6345
1	0.4587	0.4574	0.4580
2	0.3212	0.3199	0.3206
5	0.1946	0.1958	0.1952
10	0.1069	0.1083	0.1076
15	0.0629	0.0638	0.0634

Concentration (µM)	Mean Absorbance	% Inhibition
0.1	0.7445	13.1563
0.2	0.7105	17.2984
0.4	0.6345	26.1789
1	0.4580	46.7146
2	0.3206	62.6926
5	0.1952	77.2939
10	0.1076	87.4817
15	0.0634	92.7088

Log[Conc.] (log10 µM)	% Inhibition
-1.000	13.1563
-0.698	17.2984
-0.398	26.1789
0.000	46.7146
0.301	62.6926
0.699	77.2939
1.000	87.4817
1.176	92.7088

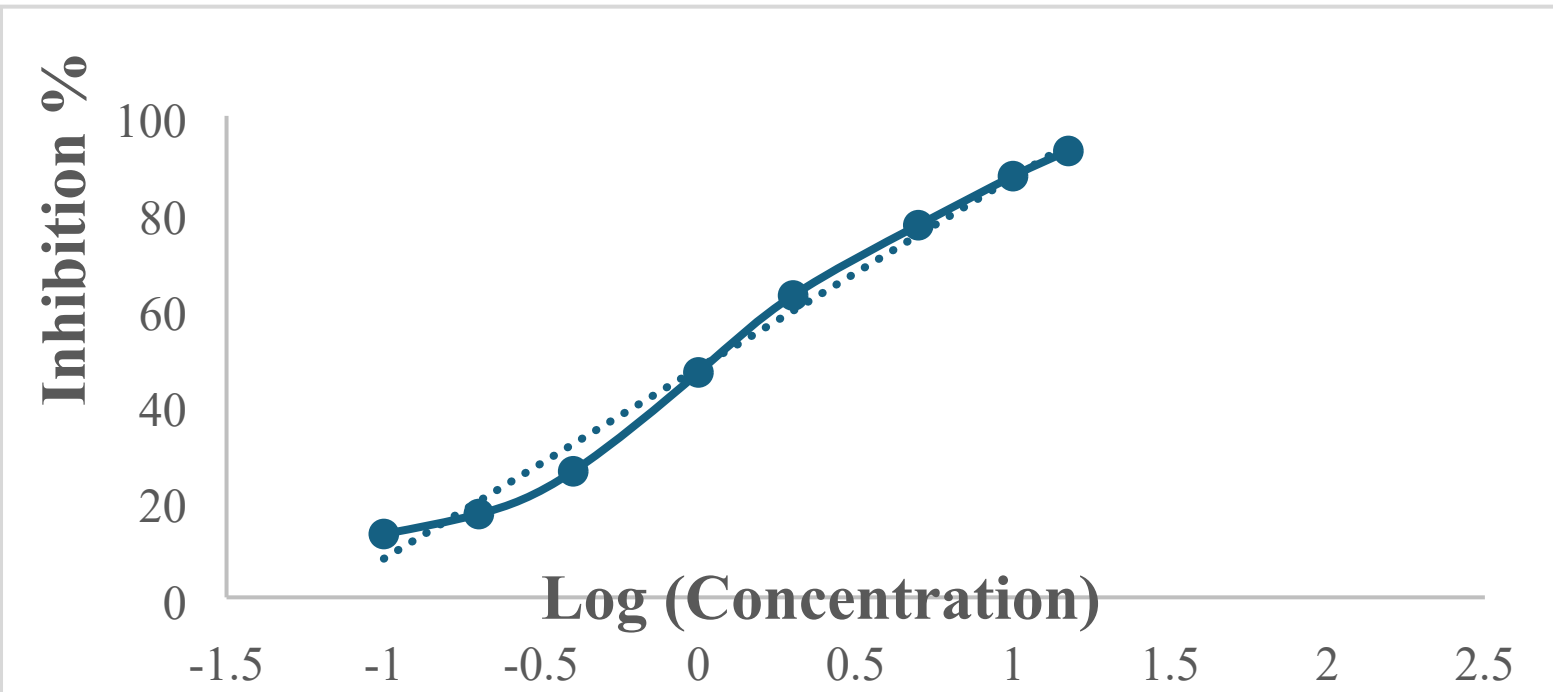

Compound 4g

IC_{50} = 0.874 µM

Concentration (µM)	Reading 1	Reading 2	Mean Absorbance
0.1	0.7486	0.7498	0.7492
0.2	0.6952	0.6941	0.6946
0.4	0.6154	0.6172	0.6163
1	0.4838	0.4859	0.4849
2	0.3712	0.3724	0.3718
5	0.2975	0.2989	0.2982
10	0.2286	0.2298	0.2292
15	0.0713	0.0731	0.0722

Concentration (µM)	Mean Absorbance	% Inhibition
0.1	0.7492	13.0443
0.2	0.6946	19.3582
0.4	0.6163	28.4568
1	0.4849	43.7025
2	0.3718	56.8273
5	0.2982	65.3681
10	0.2292	73.3889
15	0.0722	91.7203

Log[Conc.] (log10 µM)	% Inhibition
-1.000	13.0443
-0.698	19.3582
-0.398	28.4568
0.000	43.7025
0.301	56.8273
0.699	65.3681
1.000	73.3889
1.176	91.7203

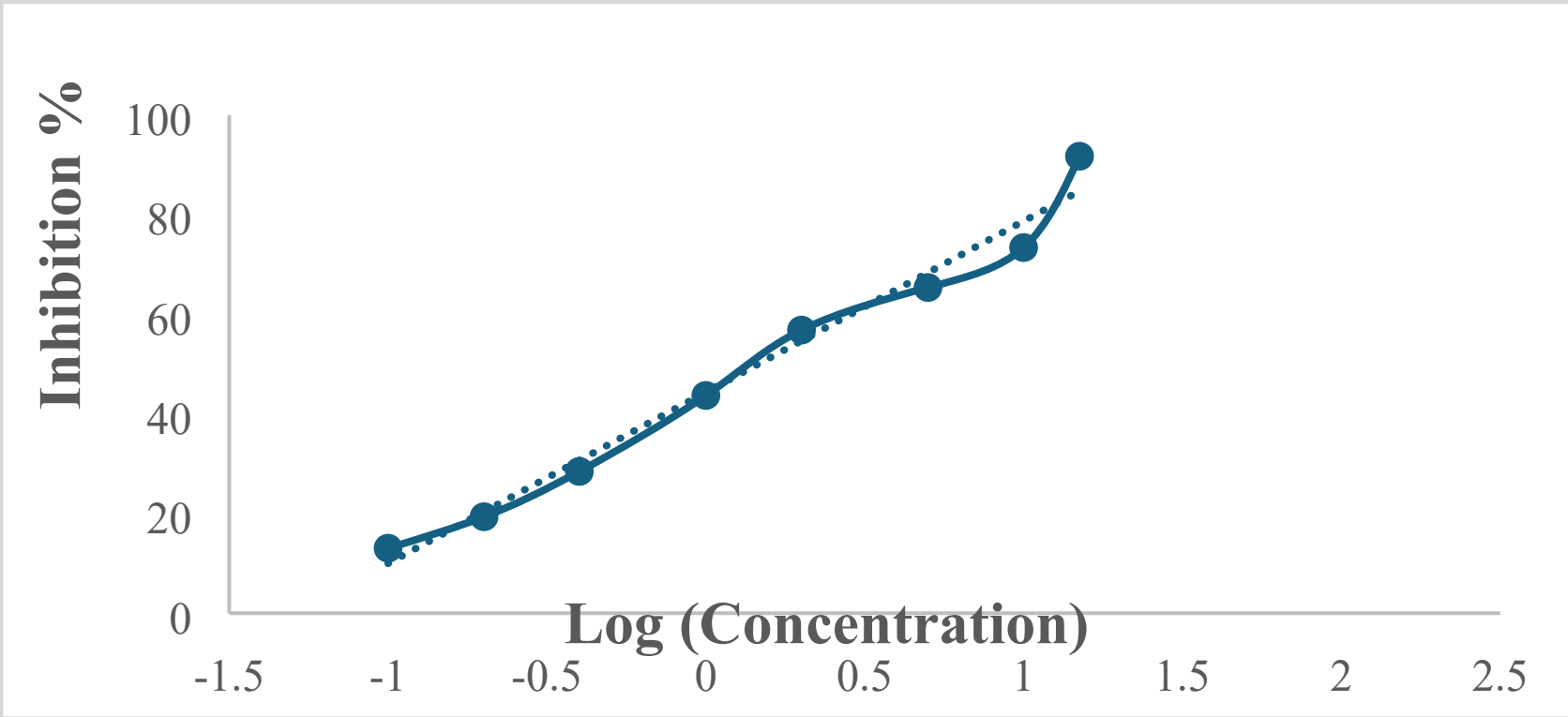

Compound 4c

IC_{50} = 0.462 **µM**

Concentration (µM)	Reading 1	Reading 2	Mean Absorbance
0.1	0.7359	0.7341	0.7350
0.2	0.6926	0.6938	0.6932
0.4	0.5824	0.5839	0.5832
1	0.3872	0.3887	0.3879
2	0.2926	0.2915	0.2921
5	0.1683	0.1672	0.1678
10	0.0946	0.0934	0.0940
15	0.0163	0.0171	0.0167

Concentration (µM)	Mean Absorbance	% Inhibition
0.1	0.7350	17.4883
0.2	0.6932	22.2427
0.4	0.5832	34.5914
1	0.3879	56.4985
2	0.2921	67.2332
5	0.1678	81.1786
10	0.0940	89.4549
15	0.0167	98.2066

Log[Conc.] (log10 µM)	% Inhibition
-1.000	17.4883
-0.698	22.2427
-0.398	34.5914
0.000	56.4985
0.301	67.2332
0.699	81.1786
1.000	89.4549
1.176	98.2066

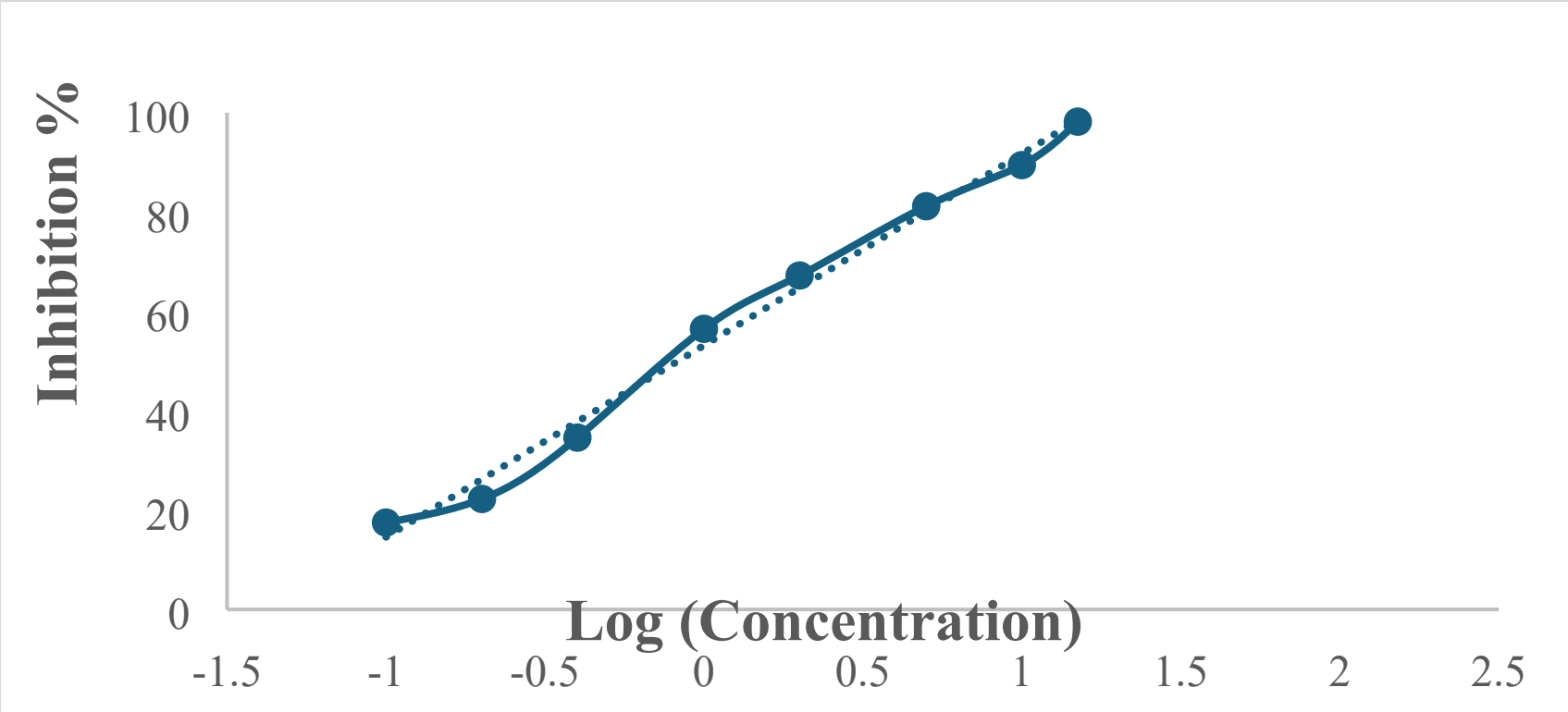

Compound Zileuton

IC_{50} = 0.689 µM

Concentration (µM)	Reading 1	Reading 2	Mean Absorbance
0.1	0.7362	0.7350	0.7356
0.2	0.6945	0.6927	0.6936
0.4	0.6113	0.6098	0.6106
1	0.4721	0.4704	0.4712
2	0.3326	0.3339	0.3333
5	0.2085	0.2071	0.2078
10	0.1167	0.1179	0.1173
15	0.0410	0.0422	0.0416

Concentration (µM)	Mean Absorbance	% Inhibition
0.1	0.7356	15.8404
0.2	0.6936	20.6584
0.4	0.6106	30.1287
1	0.4712	46.1017
2	0.3333	61.8763
5	0.2078	76.2296
10	0.1173	86.5740
15	0.0416	95.3966

Log[Conc.] (log10 µM)	% Inhibition
-1.000	15.8404
-0.698	20.6584
-0.398	30.1287
0.000	46.1017
0.301	61.8763
0.699	76.2296
1.000	86.5740
1.176	95.3966

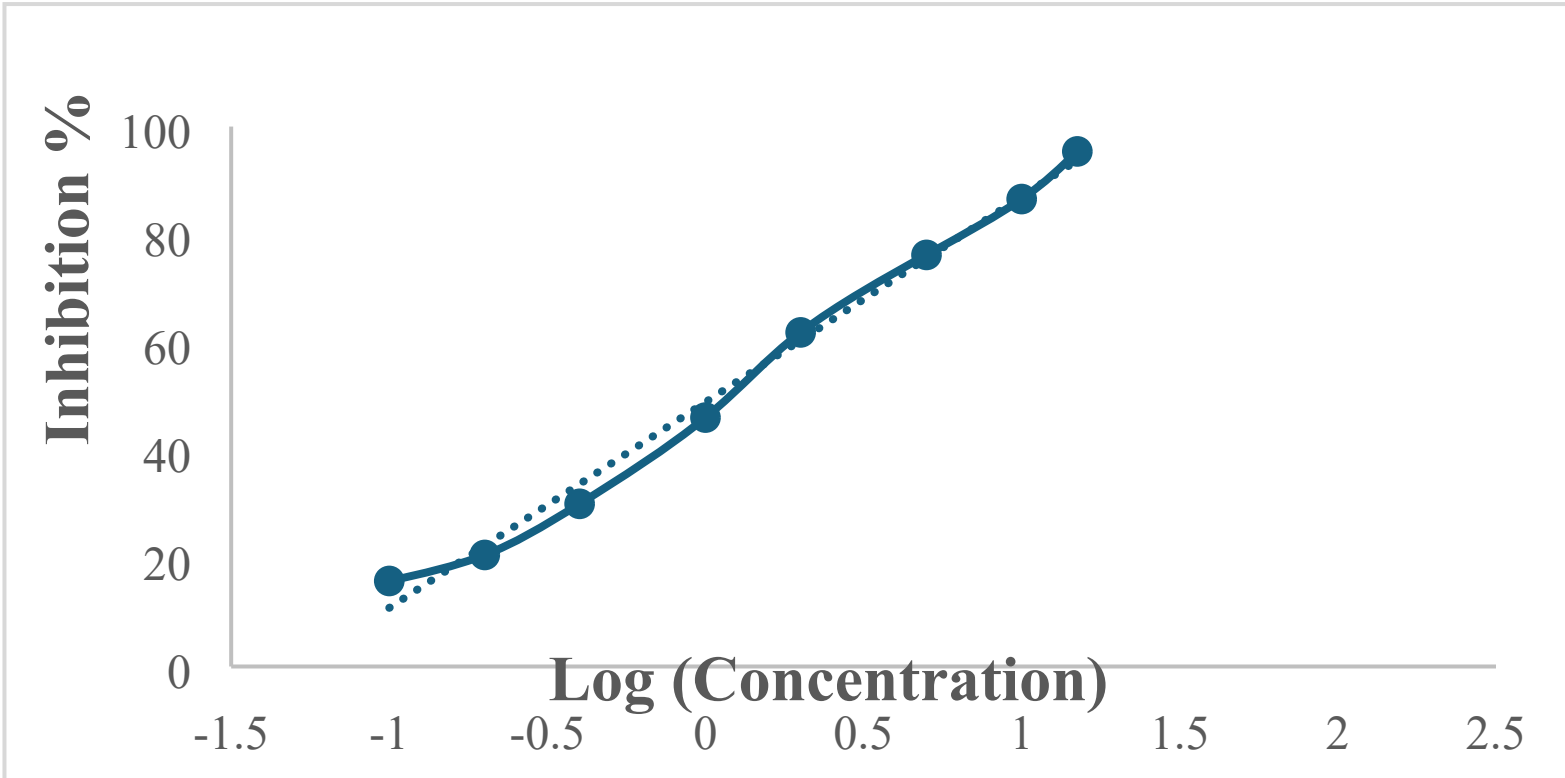
Inhibition %
100
80
60
40
20
0
Log (Concentration)
-1.5
-1
-0.5
0
0.5
1
1.5
2
2.5
